# A Unified Strategy for Arylsulfur(VI) Fluorides from Aryl Halides: Access to Ar‐SOF_3_ Compounds

**DOI:** 10.1002/anie.202009699

**Published:** 2020-10-26

**Authors:** Lin Wang, Josep Cornella

**Affiliations:** ^1^ Max-Planck-Institut für Kohlenforschung Kaiser-Wilhelm-Platz 1 Mülheim an der Ruhr 45470 Germany

**Keywords:** heterocycles, sulfinyl trifluorides, sulfonimidoyl fluorides, sulfonyl fluorides, tetrafluorosulfanyl chlorides

## Abstract

A convenient protocol to selectively access various arylsulfur(VI) fluorides from commercially available aryl halides in a divergent fashion is presented. Firstly, a novel sulfenylation reaction with the electrophilic *N*‐(chlorothio)phthalimide (Cl‐S‐Phth) and arylzinc reagents afforded the corresponding Ar‐S‐Phth compounds. Subsequently, the S(II) atom was selectively oxidized to distinct fluorinated sulfur(VI) compounds under mild conditions. Slight modifications on the oxidation protocol permit the chemoselective installation of 1, 3, or 4 fluorine atoms at the S(VI) center, affording the corresponding Ar‐SO_2_F, Ar‐SOF_3_, and Ar‐SF_4_Cl. Of notice, this strategy enables the effective introduction of the rare and underexplored ‐SOF_3_ moiety into various (hetero)aryl groups. Reactivity studies demonstrate that such elusive Ar‐SOF_3_ can be utilized as a linchpin for the synthesis of highly coveted aryl sulfonimidoyl fluorides (Ar‐SO(NR)F).

## Graphical abstract

A divergent protocol for the synthesis of various (hetero)aryl S(VI) fluorides from the same starting material is reported. The protocol proceeds through a novel metal‐free Ar−S bond formation, followed by a mild oxidation. The careful tuning of the oxidation conditions dictates the chemoselectivity of the oxidation step.

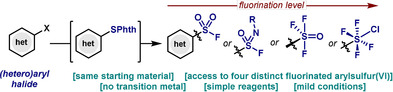

Arylsulfur compounds with the S atom in the oxidation state (VI) represent a large portion of the molecular functionalities encountered in organic chemistry. From the venerable electrophilic properties of sulfonyl chlorides^[1]^ to the antibacterial activity of sulfonamides,^[2]^ arylsulfur(VI) compounds have occupied a privileged place in the history of organic synthesis. Despite the wealth of literature on this topic,^[3]^ arylsulfur(VI) compounds where the S atom is directly attached to a fluorine atom have only recently received attention owing to their potential application as electrophiles.^[4]^ (Figure 1 A). The most persuasive example deals with the use of aryl sulfonyl fluorides (Ar‐SO_2_F), which have been shown to be extremely powerful reagents in sulfur‐fluoride exchange reactions (SuFEx).^[5]^ Similarly, aryl sulfonimidoyl fluorides (Ar‐S(O)(NR)F) have been explored due to their rapid S‐F substitution by various incoming nucleophiles (amines, alcohols, organolithium reagents).^[6]^ From the molecular point of view, both Ar‐SO_2_F and Ar‐S(O)(NR)F represent the lowest level of fluorination for an aryl S(VI) compound, with only one fluorine attached to the S atom (level 1). Tetrafluoro‐λ^6^‐sulfanyl chlorides (Ar‐SF_4_Cl) represent a class of compounds of high fluorination level (level 4), with 4 fluorines at the same S(VI) atom.^[7]^ These compounds have been demonstrated to be good precursors *en route* to the highest level of fluorination (level 5): Ar‐SF_5_ compounds.[^7c^, ^8^] Yet, compounds with mid‐levels of fluorination (level 2 and 3) are much rarer.^[9]^ A unique example of level 3 fluorination reported is the (trifluorosulfinyl)benzene (Ar‐SOF_3_).^[10]^ In 1980, Ruppert disclosed the reaction of diphenyl sulfoxide with F_2_ and NaF at −115 °C in CFCl_3_, obtaining a remarkable 20 % of Ph‐SOF_3_ (Figure 1 B). This compound was observed to be an extremely good electrophile at S, which anticipates its tremendous potential for organic synthesis. Yet, the uncommon starting material, necessity of a dangerous gas, in combination with the low yield of product, prevented diversity of Ar‐SOF_3_.

**Figure 1:**
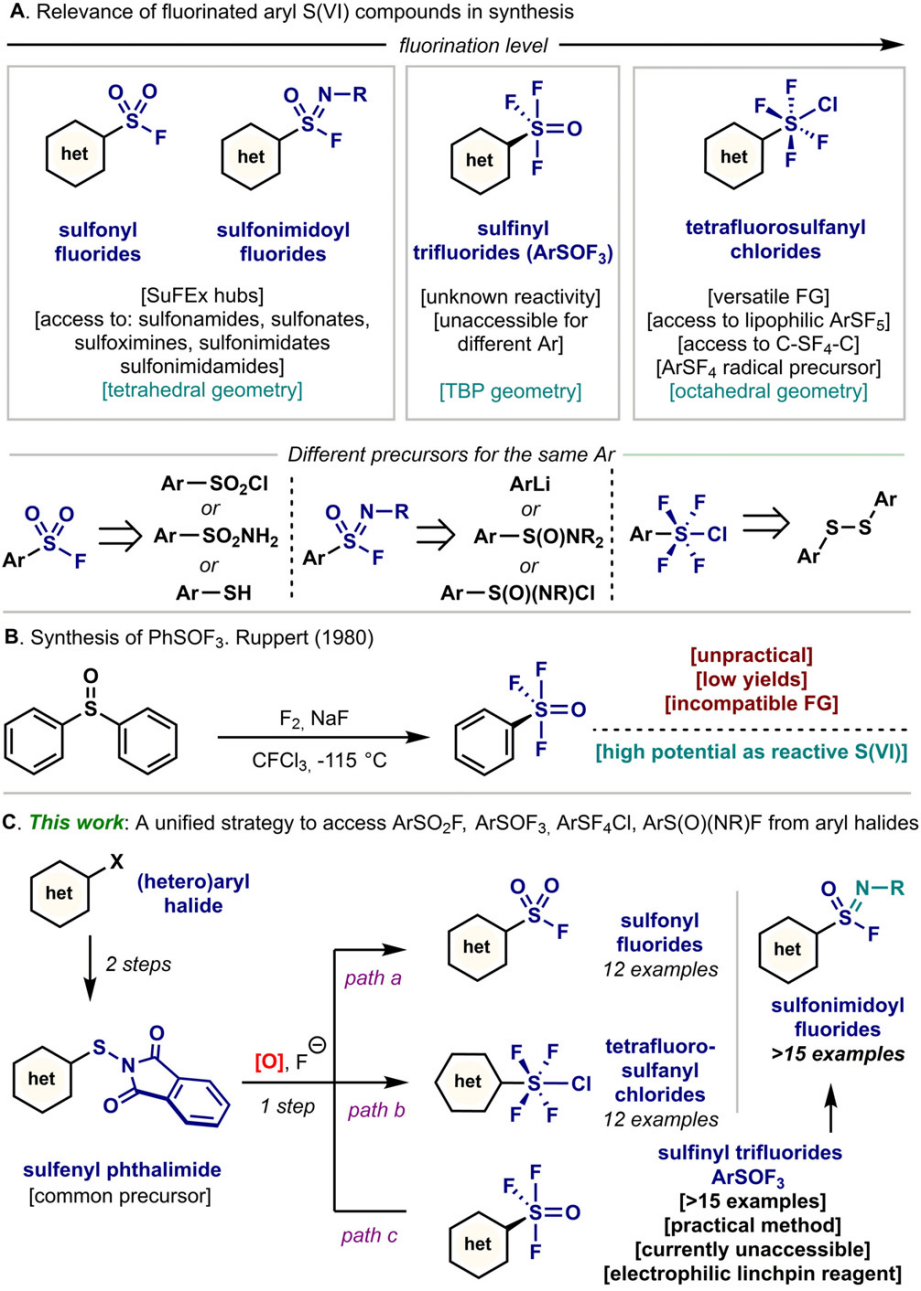
A) Various fluorinated arylsulfur(VI) compounds with different levels of fluorination and their precursors for synthesis. B) Previous work for synthesis of Ph‐SOF_3_. C) Our unified strategy for selective synthesis of Ar‐SO_2_F, Ar‐SOF_3_, Ar‐SF_4_Cl, and Ar‐S(O)(NR)F from aryl halides.

One of the main drawbacks associated to the synthesis of fluorinated arylsulfur(VI) compounds is the limited number of unified strategies using the same aryl synthon, that lead to different fluorination levels. For example, synthesis of aryl sulfonyl fluorides has mainly relied on the anion exchange from the parent arylsulfonyl chlorides.[^5a^, ^11^] Recent examples also disclosed the possibility of obtaining sulfonyl fluorides from the corresponding thiophenols (Ar‐SH),^[12]^ aryl halides (Ar‐X)^[13]^ or arylsulfonamides (Ar‐SO_2_NH_2_).^[14]^ Access to aryl sulfonimidoyl fluorides is currently possible by halogen exchange from sulfonimidoyl chlorides,^[15]^ oxidation of sulfinamide salts^[16]^ or aryl lithium addition to S(O)(NR)F_2_.^[6b]^ Finally, state‐of‐the‐art synthesis of Ar‐SF_4_Cl mainly relies on the oxidation of the corresponding aryl disulfides.^[7]^

With the aim of providing a unified approach to synthesize various fluorinated arylsulfur(VI) compounds *from the same starting material*, herein we present a strategy that enables access to Ar‐SOF_3_, Ar‐SO_2_F, Ar‐SF_4_Cl and Ar‐S(O)(NR)F, from the corresponding commercially available aryl halides (Figure 1 C). The methodology capitalizes in the formation of an arylsulfenyl phthalimide precursor by virtue of the reaction of the ArZnCl with the common Cl‐S‐Phth reagent. A subsequent selective oxidation‐fluorination affords the corresponding Ar‐SO_2_F (*path a*), Ar‐SF_4_Cl (*path b*) and the underexplored Ar‐SOF_3_ (*path c*) in high chemoselectivity and yields. This protocol benefits from a high functional group tolerance, accommodating a variety of *N*‐containing heterocycles. Furthermore, addition of a primary amine into the Ar‐SOF_3_ in the presence of base affords the corresponding Ar‐S(O)(NR)F.

At the onset of this project, we envisaged that a divergent access to fluorinated arylsulfur(VI) compounds from aryl halides should rely on the construction of a common precursor: an “active” arylsulfur(II). Moreover, the method should be facile, robust and with a high degree of functional group tolerance. Inspired by previous work on electrophilic sulfur reagents^[17]^ and after a survey of various candidates (e.g. Bunte salts,^[18]^ Ar‐S‐S(O)_2_Ph^[19]^ or Ar‐S‐CN^[20]^), *N*‐(arylsulfenyl)phthalimides (Ar‐S‐Phth) attracted our attention due to their thermal stability upon storage despite the weak S−N bond.^[21]^ Typically, Ph‐S‐Phth is prepared from the reaction of thiophenol with phthalimide in the presence of Br_2_ or SO_2_Cl_2_.^[22]^ However, the synthesis of such compounds from the largely more available aryl halides is virtually unknown. After scanning different sulfur(II) electrophilic reagents and reaction conditions (see Supporting Information), we found that a protocol based upon simple ArZnX formation^[23]^ followed by nucleophilic addition to *N*‐(chlorothio)phthalimide^[24]^ (Cl‐S‐Phth, **2**, Table 1 A), led to a straightforward synthesis of Ar‐S‐Phth precursors (**3**) in good yields. It is interesting to note the chemoselectivity in favor of the displacement of the Cl over the good leaving group phthalimide. These compounds are indeed bench‐stable, easy to handle and the S atom is attached to a good leaving group (Phth), which is crucial in the forthcoming modification (vide infra).

**Table 1:** A) Sulfenylation of aryl zinc reagents. See Supporting Information for reaction conditions. B) Optimization of the reaction conditions for the divergent synthesis of arylsulfur(VI) oxyfluoride compounds. 
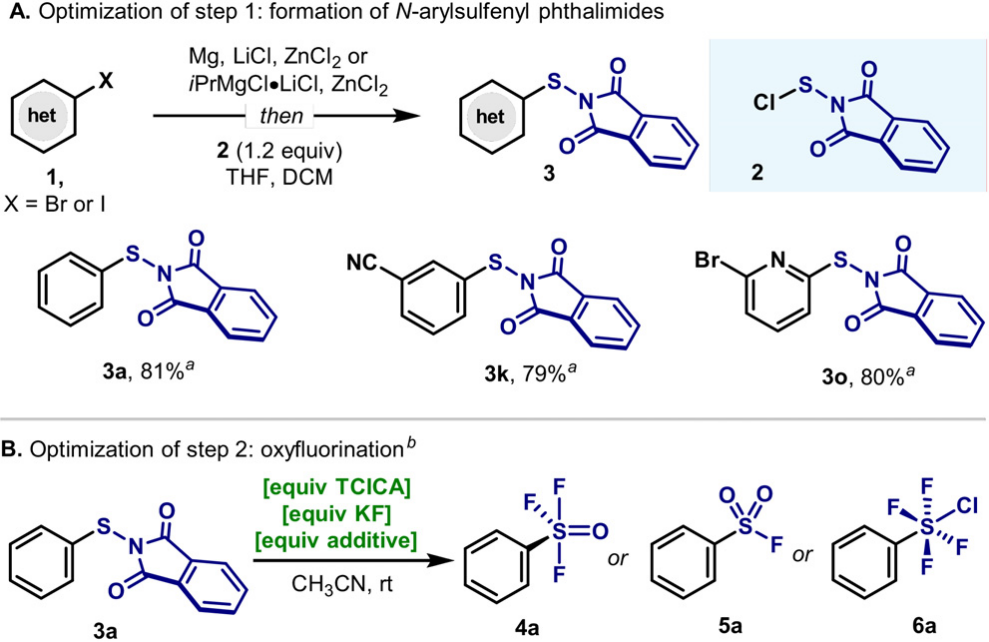

Entry	**TCICA** [equiv]	**KF** [equiv]	**Additive** [equiv]	Yield [%]^[c]^ (**4 a**+**5 a**+**6 a**)	Ratio^[c]^ (**4 a**:**5 a**:**6 a**)
*1*	*9*	*16*	*TFA (2)*	*>95*	*96:4:<0.1*
2	9	16	TFA (1)	71	65:28:17
3	9	16	TFA (0.5)	58	34:28:38
4	9	16	AcOH (2)	64	<0.1:99:<0.1
5	9	16	H_2_O (2)	56	<0.1:99:<0.1
*6*	*9*	*16*	*MeOH (2)*	*>95*	*2:98:<0.1*
7	9	16	TFA (0.1)	72	21:<0.1:79
8^[d]^	9	16	TFA (0.1)	82	26:<0.1:74
*9* ^[e]^	*18*	*32*	*TFA (0.1)*	*>95*	*12:2:85*

[a] Yields of isolated products, the detailed procedure of preparation see Supporting Information. [b] Reaction conditions: **3 a** (0.1 mmol), TCICA, KF, and additives in 1 mL MeCN at room temperature for 24 h. [c] ^19^F NMR yields, α,α,α‐trifluorotoluene as the internal standard. [d] Reaction time 48 h. [e] 0.2 mmol **3 a** as the starting material.

With the Ar‐S‐Phth precursors (**3**) in hand, we set out to explore the second step of the synthetic route. Inspired by the remarkable findings recently reported by Pitts, Santschi and Togni *en route* to fluorinated aryl‐S(VI),^[7b]^ ‐Te^IV^ ^[25]^ and ‐Se^IV^ ^[26]^ compounds from ArCh‐ChAr, we attempted the oxyfluorination of Ph‐S‐Phth with a mixture of trichloroisocyanuric acid (TCICA) and potassium fluoride (KF), in the presence of additives. During our initial oxidation experiments, we were pleased to observe the formation of the rare Ph‐SOF_3_ (**4 a**),^[10]^ accompanied by large amounts of PhSO_2_F (**5 a**) and PhSF_4_Cl (**6 a**). The formation of **4 a** together with unselective formation of **5 a** and **6 a**, suggested that access to different levels of fluorination at S(VI) could be within reach. A short optimization revealed that TCICA (9 equiv), KF (16 equiv) and TFA (trifluoroacetic acid, 2 equiv) afforded **4 a** (Table 1 B, entry 1) in excellent yield (95 %) and chemoselectivity (**4 a**:**5 a**=96:4). In the ^19^F NMR, a typical AX_2_ pattern appears at 64.5 ppm (t, *J(F_ax_,F_eq_)*=157.9 Hz) and 100.6 ppm (d, *J(F_ax_*,*F_eq_)*=157.9 Hz) with an integral ratio of 1:2, which is in agreement with a trigonal bipyramidal geometry (TBP) of the S(VI) where two fluorine atoms are placed in the apical positions.^[27]^ The remaining fluorine, the phenyl and the O atom occupy the vertexes of the trigonal plane perpendicular to the main axis. This result is in line with Ruppert's analysis about the geometry of this functionality.^[10]^ The differences in chemical shift for both F signals (axial vs. equatorial) resemble those in fluorinated S(VI) compounds containing axial and equatorial F atoms, such as the octahedral Ar‐SF_5_.^[7a]^ However, such differences are minimal compared to other TBP geometries of Ar−S compounds in lower oxidation state such as Ar‐SF_3_.^[7c]^

The high yields obtained for **4 a** from a simple starting material represents a tremendous step forward for the chemistry of S(VI) and also provides a safer and synthetically useful platform to produce a variety of Ar‐SOF_3_ compounds (vide infra). Yet, such compounds exhibit extremely high reactivity and rapidly evolve into Ph‐SO_2_F upon exposure to traces of H_2_O, which made isolation attempts and crystallographic analysis completely unsuccessful.

Interestingly, this methodology is significantly sensitive to the amount and type of additives. When the loading of TFA was decreased, both yield and selectivity of **4 a** significantly decreased (Table 1 B, entries 2 and 3). Moreover, when TFA was replaced by AcOH, H_2_O or MeOH, a complete switch of chemoselectivity was observed, favouring the formation of Ph‐SO_2_F (**5 a**) as the main product (Table 1 B, entries 4–6). Refinement of the reaction parameters led to the optimized conditions shown in entry 6: in the presence of 2 equiv MeOH, Ph‐SO_2_F (**5 a**) was obtained in almost quantitative yield (95 %) and selectivity (**4 a**:**5 a**=2:98). When the amount of oxidant and fluoride was increased, (18 equiv TCICA, 32 equiv KF) and catalytic amount of TFA was used, the corresponding Ph‐SF_4_Cl (**6 a**) was obtained in high yield (Table 1 B, entry 9). This is in complete agreement with Togni's results, and provides an alternative synthetic method which departs from the use of disulfides as starting materials.^[7a]^ The results in Table 1 permit the practitioner to select between three levels of fluorination (Ar‐SOF_3_, Ar‐SO_2_F or Ar‐SF_4_Cl) commencing from the same starting material by simply tuning the oxidation conditions.

Under the optimized conditions in Table 1, the scope on the aryl moiety for the synthesis of various Ar‐SOF_3_ was explored (Scheme 1). Using aryl halides containing other halogen atoms (F, Cl and Br), oxidative fluorination to Ar‐SOF_3_ was observed in good yields (**4 b**–**4 d**, 81–90 %). On the contrary, the presence of electron‐donating groups such as methyl (**4 e**–**4 f**) and methoxy (**4 g**) significantly affected the reactivity, especially when at the *ortho*‐position (**4 f**, 41 %). This is mainly due to the low conversion of the Ar‐S‐Phth to Ar‐SOF_3_. With the aim of having Ar‐SOF_3_ as potential scaffolds for organic synthesis, it is essential that the oxidative fluorination tolerates other functional groups. Indeed, this protocol permits the presence of nitro (**4 h**), nitrile (**4 k**), ester (**4 l**) and trifluoromethyl (**4 i**–**4 j**) as well as other aromatic (**4 m** and **4 n**) groups. Remarkably, some heterocyclic halides were able to be transformed to the corresponding sulfinyl trifluoride. For example, unsubstituted pyridine (**4 o**), halogenated pyridines (**4 p**–**4 q**) and a pyridine bearing a trifluoromethyl group (**4 r**) were obtained in moderate to good yields. Of notice, the sulfinyl trifluoride could also be obtained for the challenging 2‐pyrimidine (**4 s**) albeit in low yields (15 %). Unfortunately, other heterocycles with pyrimidinyl‐ or thienyl groups resulted in no conversion toward **4 t** or **4 u**, respectively. When low‐yield or no conversion is obtained for heterocyclic substrates, higher amounts of Ar‐SF_4_Cl and Ar‐SO_2_F were observed. Monitoring the formation of **4 d** by ^19^F NMR, indicated that Ar‐SOF is formed very rapidly at the initial stages of the reaction in the presence of 2 equiv of TFA. This intermediate is then oxidized further and after halogen exchange it affords **4 d** (SI for details).

**Scheme 1:**
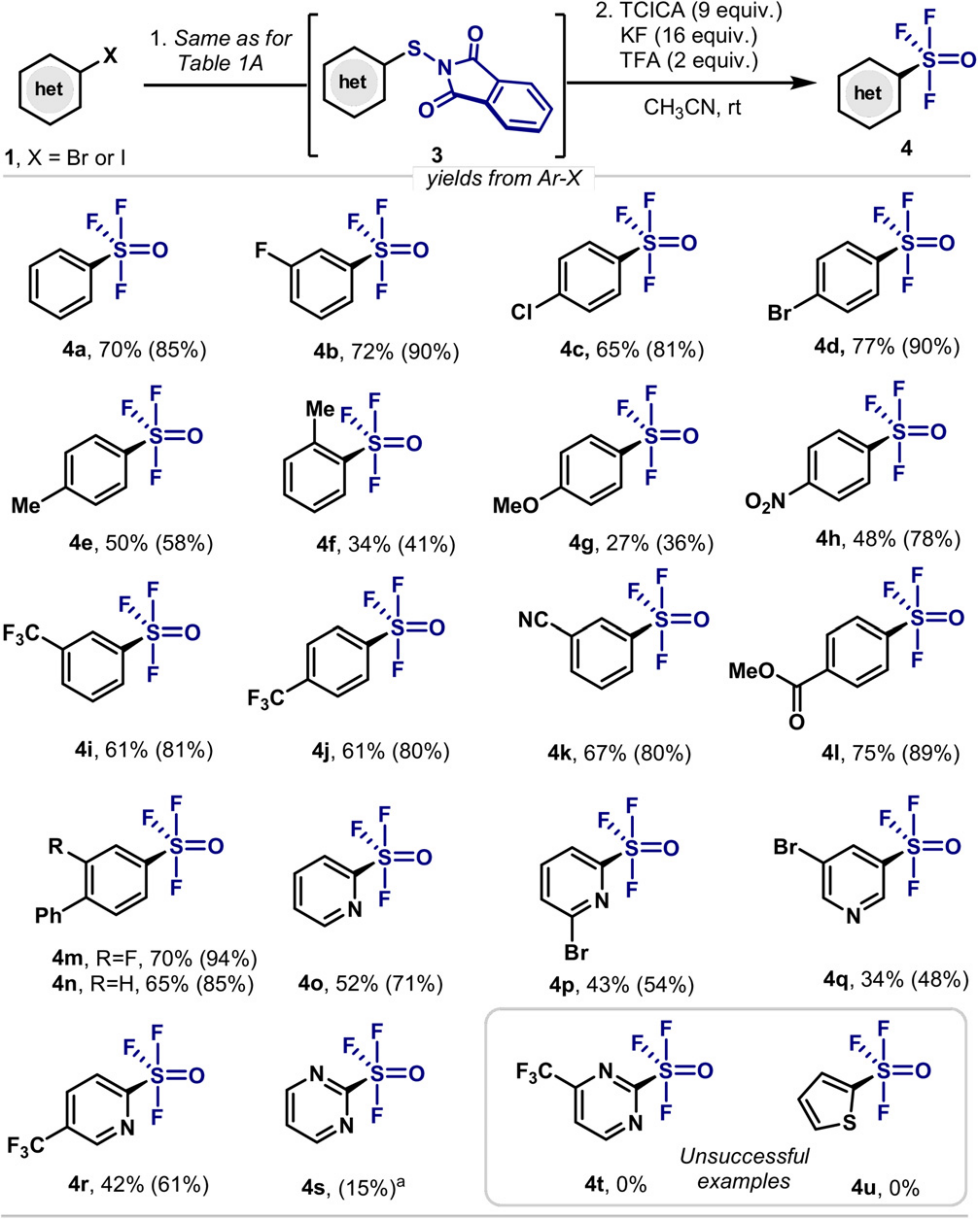
Synthesis of Ar‐SOF_3_ from aryl halides. Reaction conditions: The Ar‐S‐Phth **3** was firstly synthesized based on the general methods from corresponding aryl halides. After purification through column chromatography, **3** (0.1 mmol), TCICA (0.9 mmol), KF (0.16 mmol), TFA (0.2 mmol) were stirred in MeCN (1 mL), RT for 24 h. Yields in parentheses correspond to those from Ar‐S‐Phth (based on ^19^F NMR spectra, PhCF_3_ as internal standard); yields outside the parenthesis correspond to those from **1**. [a] Reaction conditions: **3 s** was prepared from the reaction of *N*‐chlorophthalimide with 2‐pyrimidinethiol.

As shown in Table 1B, replacement of TFA by MeOH afforded the corresponding (hetero)arylsulfonyl fluorides. Scheme 2 explores the generality of these conditions for the synthesis of various Ar‐SO_2_F compounds (**5**). Of importance is that either electron‐withdrawing (**5 b**–**5 c**, **5 f**–**5 j**) or electron‐donating groups (**5 d**–**5 e**) presented similar reactivity, with yields ranging from 53 % to 81 %. Furthermore, aryl groups having the reactive site between two bulky methyl‐ groups did not affect the reactivity, as shown by the 58 % yield of **5 e**.^[28]^ It is important to mention that also in this case, the presence of some *N*‐basic functionalities did not affect the outcome of the reaction as highlighted by the high yields obtained by pyridines **5 k** and **5 l**.

**Scheme 2:**
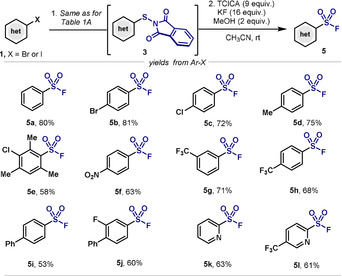
Synthesis of Ar‐SO_2_F from aryl halides. Reaction Conditions: The Ar‐S‐Phth **3** was firstly synthesized based on the general methods from corresponding aryl halides. After purification through column chromatography, **3** (0.1 mmol), TCICA (0.9 mmol), KF (0.16 mmol), MeOH (0.2 mmol) were stirred in MeCN (1 mL), RT for 24 h. Overall yields from **1**.

When increasing the loading of TCICA and KF in combination with catalytic amounts of TFA, ArSF_4_Cl (**6**) was obtained selectively from (hetero)aryl halides (Table 1B, entry 9). When exploring the generality of this protocol, similar electronic tendencies to the synthesis of Ar‐SOF_3_ were obtained (Scheme 3). Electron‐withdrawing substituents present in the aryl unit resulted in higher yields (**6 b**, **6 e**–**6 h**) than aryl halides bearing electron‐donating groups (**6 c**–**d**). More importantly, heterocyclic substrates were smoothly converted, as exemplified by the good yields obtained using various pyridines (**6 i**, **6 k**–**6 l**) and pyrimidines (**6 j**).

**Scheme 3:**
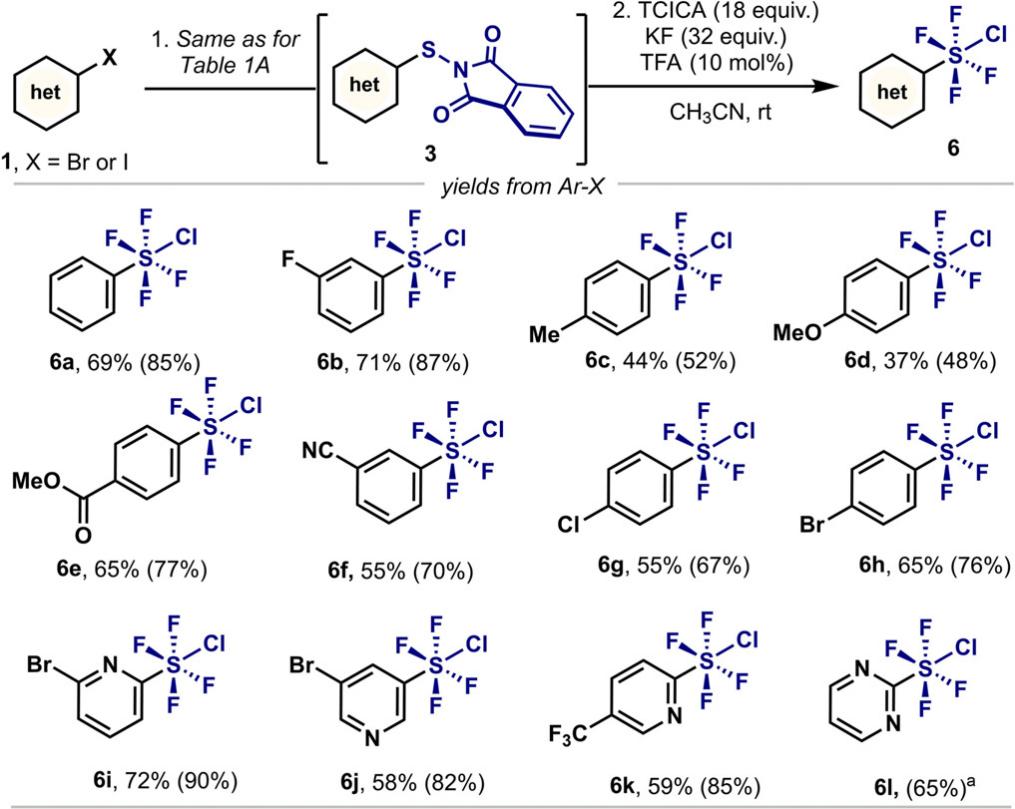
Synthesis of Ar‐SF_4_Cl from aryl halides. Reaction Conditions: The Ar‐S‐Phth **3** was firstly synthesized based on the general methods from corresponding aryl halides. After purification through column chromatography, **3** (0.2 mmol), TCICA (3.6 mmol), KF (6.4 mmol), TFA (0.02 mmol) were stirred in MeCN (2 mL), RT for 24 h. Yields in parentheses correspond to those from Ar‐S‐Phth (based on ^19^F NMR spectra, PhCF_3_ as internal standard); yields outside the parenthesis correspond to those from the parent Ar‐X. [a] The Ar‐S‐Phth precursor was prepared from the reaction of *N*‐chlorophthalimide with 2‐pyrimidinethiol.

Based on the high electrophilicity exhibited by Ar‐SOF_3_ compounds and their ability to rapidly engage with traces of H_2_O to forge Ar‐SO_2_F, we envisaged that synthesis of the important sulfonimidoyl fluorides (ArS(O)(NR)F) should be within reach when primary amines are used. Indeed, when **4** was reacted with a primary amine (**7**) in the presence of Et_3_N, smooth conversion to the corresponding sulfonimidoyl fluorides (**8**) was observed (Scheme 4). For example, the reaction of **4 d** with different anilines led to **8 a**–**8 c** in high yields (63–70 %). Of particular importance is the tolerance of the styrenyl moiety in **8 c**. Benzylamines or aliphatic primary amines also boded well with this protocol, affording good yields of the corresponding sulfonimidoyl fluorides (**8 d**–**8 f**).

**Scheme 4:**
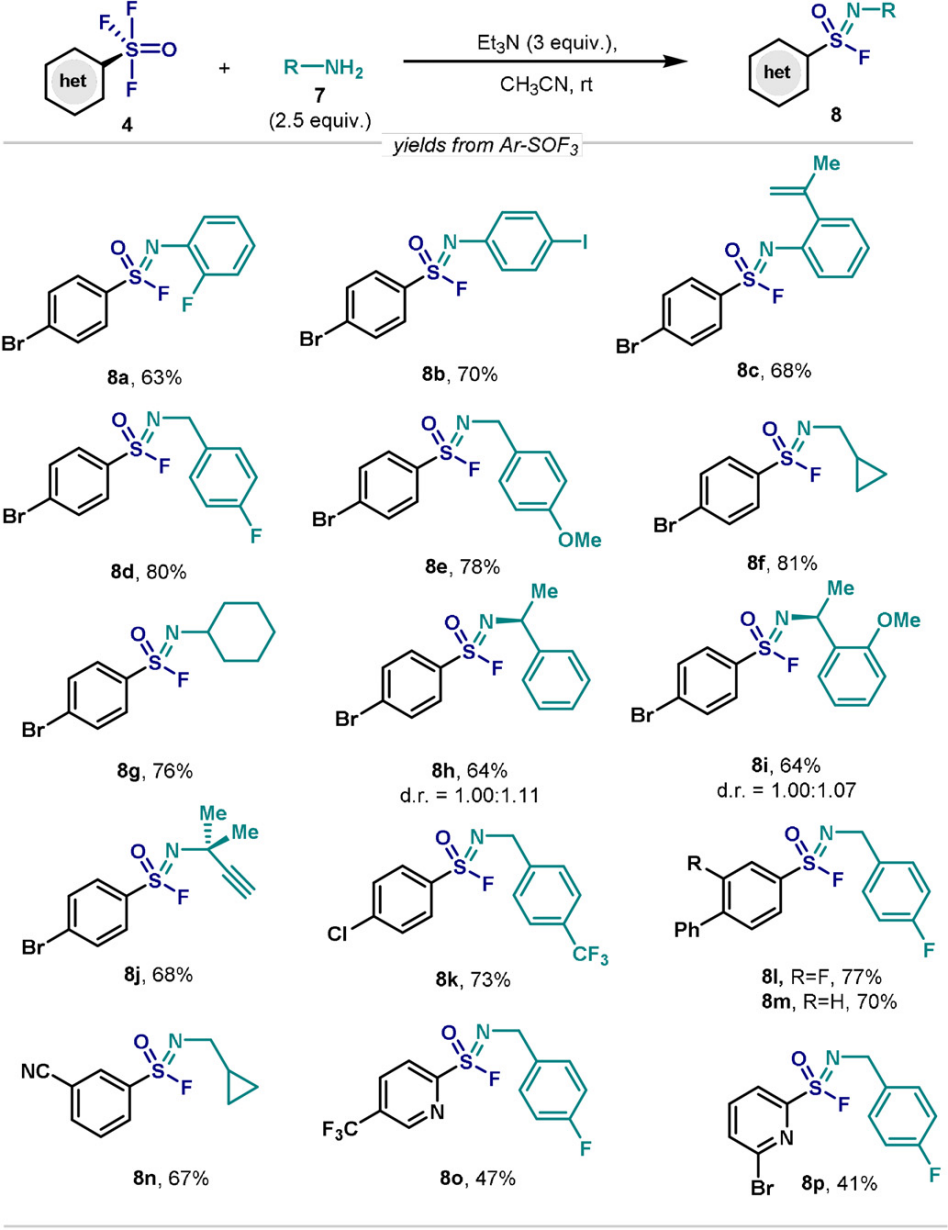
Exploration of reactivity of Ar‐SOF_3_ with amines. Reaction conditions: ArSOF_3_ (0.1 mmol), amine (0.25 mmol), NEt_3_ (0.3 mmol) in MeCN (1 mL) at RT for 18 h. Yields of isolated products.

Additionally, the steric hindrance on the amine partner was also explored. Gratifyingly, di‐ or tri‐ substituted amines did not pose any difficulties, affording the desired compounds in good yields (**8 g**–**8 j**). Due to the chiral nature of the S(VI) center in **8**, compounds **8 h** and **8 i** were obtained as a mixture of diastereoisomers, with a d.r. of approximately 1:1, judging by the two different ^19^F NMR signal for each isomer.^[6b]^ Modification of the aryl moiety in **4** did not affect the reactivity dramatically, as judged by the good yields of **8** with different aryl (**8 k**–**8 n**) and heteroaryl (**8 o**–**8 p**) groups.

In conclusion, we have developed a protocol that enables access to four different fluorinated aryl S(VI) compounds from the same aryl precursor. The first step introduces the S atom selectively into the (hetero)aryl group by virtue of a novel reaction between an ArZnX and the electrophilic Cl‐S‐Phth. The second step consists in a chemoselective oxidation of the S(II) atom. Slight modification of the oxidation step leads to chemoselective synthesis of various fluorinated S(VI) compounds: Ar‐SOF_3_, Ar‐SO_2_F and Ar‐SF_4_Cl. The potential of this bottom‐up approach is highlighted in the access to the unusual Ar‐SOF_3_, which up until now, only forcing conditions allowed its synthesis in low yields. Further examination on the electrophilic properties of Ar‐SOF_3_ leads to its potential use as linchpin functionality to access another synthetically useful S(VI) compound, namely the Ar‐SO(NR)F. Finally, the synthetic versatility of this protocol is represented by the >50 examples provided overall, including Lewis‐basic containing heterocycles. The operational simplicity of the protocol might become appealing for practitioners, and is envisaged to find applications in the rapid assembly of aryl S(VI) compounds.

## Conflict of interest

The authors declare no conflict of interest.

## Supporting information

As a service to our authors and readers, this journal provides supporting information supplied by the authors. Such materials are peer reviewed and may be re‐organized for online delivery, but are not copy‐edited or typeset. Technical support issues arising from supporting information (other than missing files) should be addressed to the authors.

Supplementary
