# Supplementary material for: A Unified Strategy for Arylsulfur(VI) Fluorides from Aryl Halides: Access to Ar‐SOF3 Compounds
Source: Angew Chem Int Ed Engl. 2020 Oct 26;59(52):23510–5. doi: 10.1002/anie.202009699 (PMC7756513; doi:10.1002/anie.202009699)

Angewandte Chemie
Eine Zeitschrift der Gesellschaft Deutscher Chemiker

Supporting Information

A Unified Strategy for Arylsulfur(VI) Fluorides from Aryl Halides: Access to Ar-SOF_3 Compounds

*Lin Wang and Josep Cornella**

anie_202009699_sm_miscellaneous_information.pdf

Table of Contents

1. General experimental considerations 4

2. Arylsulfur(II) precursors candidates 5

3. Oxyfluorination reactivity of arylsulfur(II) precursor candidates 5

4. Optimization of Ph-S-Phth from phenyl halides 6

5. Preparation of Ar-S-Phth 7

5.1 Preparation of 1M THF solution of $ZnCl_2$ 7

5.2 General method for preparation of organozinc halides from aryl halides 7

5.3 General method for synthesis of Phth-S-Cl 7

5.4 General method for the synthesis of Ar-S-Phth 8

6. Optimization of oxyfluorination step 17

7. General method for the synthesis of $ArSOF_3$ 18

8. Scope of $ArSOF_3$ 19

9. Analysis of the structure of $ArSOF_3$ 39

10. General method for the synthesis of $ArSO_2F$ 45

11. Scope of $ArSO_2F$ 46

12. General method for the synthesis of $ArSF_4Cl$ 52

13. Scope of $ArSF_4Cl$ 53

14. Study on the mechanism for the oxidation step toward $ArSOF_3$, $ArSO_2F$ and $ArSF_4Cl$ 66

14.1 ^{19}F NMR monitor of the reaction for $ArSOF_3$ 66

14.2 The reaction for the formation of $ArSO_2F$ in the presence of $H_2^{18}O$ 68

14.3 ^{19}F NMR monitoring of the reaction for $ArSF_4Cl$ 69

14.4 Possible mechanism for the divergent formation of $ArSOF_3$, $ArSO_2F$ and $ArSF_4Cl$ 70

15. General method on synthesis of ArS(O)(NR)F 71

16. Scope for synthesis of ArS(O)(NR)F 72

17. References 81

18. NMR Spectra 83

1. General experimental considerations

Unless otherwise stated, all manipulations were performed using standard Schlenk techniques under dry argon in flame-dried glassware. Anhydrous solvents were distilled from appropriate drying agents and were transferred under Argon: THF (Mg/anthracene), CH_2Cl_2 (CaH_2), CH_3CN (CaH_2), hexane (Na/K), EtOH, MeOH (Mg), Et_3N (MS). Aryl halides, amines and phthalimide were purchased from Sigma-Aldrich, Alpha Aesar and TCI and used without prior drying or purification. Mg powder was activated with diluted hydrochloric acid followed by washing with water (3 times) and hexane (3 times). The active Mg powder was dried under vacuum (10^{-3} mbar) and stored under argon. Turbo Grignard reagent (*i*PrMgCl·LiCl, 1.3 M in THF) was purchased from Sigma-Aldrich. Trichloroisocyanuric acid (TCICA, powder) was purchased from Alpha Aesar and transferred in an argon-filled glovebox before usage. Potassium fluoride (KF, powder) was purchased from Sigma-Aldrich, rigorously dried under high vacuum (10^{-6} mbar) at 120 °C for 24 h and stored under argon. Flash chromatography: Merck silica gel 60 (40-63 µm). GC-MS (FID): GC-MS-QP2010 equipped (Shimadzu Europe Analytical Instriuments). MS (EI): Finnigan MAT 8200 (70 eV), ESI-MS: ESQ 3000 (Bruker). Accurate mass determinations: Bruker APEX III FT-MS (7 T magnet) or MAT 95 (Finnigan). NMR spectra were recorded using a Bruker Avance VIII-300, Bruker Avance III HD 400 MHz spectrometer, Bruker Avance III 500MHz spectrometer equipped with a 5mm BBFO probe. ^{1}H NMR spectra (300.13 MHz, 400.1 MHz, or 500 MHz) were referenced to the residual protons of the deuterated solvent used. All ^{19}F NMR spectra were acquired on either a 300, 400, or 500 MHz spectrometer. For ^{19}F NMR yield determination, α, α, α-trifluorotoluene was used as internal standard (^{19}F, d = –63.10 in CD_3CN). $^{13}C\{^{1}H\}$ NMR spectra (75.47 MHz. 101 MHz, or 125 MHz) were referenced internally to the D-coupled ^{13}C resonances of the NMR solvent. All compounds purified by column chromatography are shown with the isomerized product in the same spectra. Chemical shifts (δ) are given in ppm, relative to deuterated solvent residual peak, and coupling constants (J) are provided in Hz.

2. Arylsulfur(II) precursors candidates

SP-1 to **SP-5** were prepared according to the literature procedures:

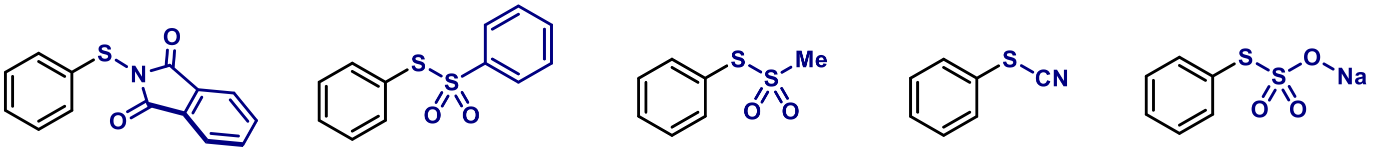

SP-1 (3a) [1] **SP-2** [2] **SP-3** [3] **SP-4** [4] **SP-5** [5] *'Bunte Salts'*

3. Oxyfluorination reactivity of arylsulfur(II) precursor candidates

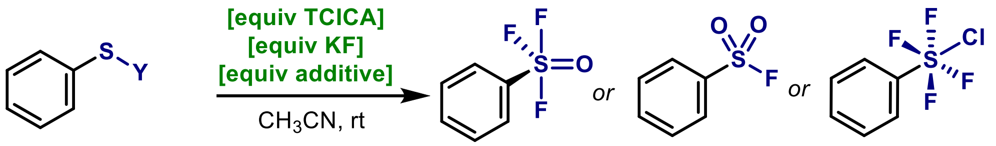

SP1 - SP5 **4a** **5a** **6a**

Conditions C: To produce Ph-SF_4Cl, TCICA (18 eq.), KF (32 eq.), TFA (10 mol%) in CH_3CN at r.t. 24 h

Inspired by the recent developments reported from Togni *en route* to fluorinated aryl–S(VI), –Se(VI) and –Te(IV) compounds from ArCh–ChAr [6], we explored the oxidative fluorination of 5 phenylsulfur(II) precursors (**SP1** – **SP5**) with trichloroisocyanuric (TCICA) / potassium fluorides (KF). The results are outlined below:

Table S1. Compare of the oxyfluorination reactivity of 5 S(II) precursors (**S1** – **S5**)

S(II) Precursor	**SP1**	**SP2**	**SP3**	**SP4**	**SP5**
Yield (**4a**+**5a**+**6a**, %)	>95	>100 (?)	14	<5	17
Ratio (**4a**:**5a**:**6a**)	14:<0.1<85	<0.1:78:26	<0.1:99:<0.1	-	<0.1:99<0.1

Table S1 indicated that the Ph-S-Phth (**SP-1**, **3a**) is the most appropriate precursor for the oxyfluorination reaction, which afforded the highest yield and excellent selectivity towards **6a**. When **SP-3, SP-4** and **SP-5** were used as precursors, the conversion is low and no **6a** was produced. As for **SP2**, $PhSO_2$- played the role as the leaving group, which could be conversed to $PhSO_2F$ under the condition (It is the reason why the yield is beyond 100%). Low selectivity and too much side-product make **SP2** inferior to **SP1**.

4. Optimization of Ph-S-Phth from phenyl halides

Typically, Ph–S–Phth is prepared from the reaction of thiophenol with phthalimide in the presence of Br_2 or SO_2Cl_2.[1] Yet, the synthesis of such compounds from the readily available aryl halides is virtually unknown. In order to synthesize these compounds from aryl halides, N,N'-Thiobisphthalimide (Phth-S-Phth, **Reagent A**), N,N'-Dithiobis(phthalimide) (Phth-S-S-Phth, **Reagent B**) and Phthalimidesulfenyl chloride (Phth-S-Cl, **Reagent C**) were chosen as coupling partners. The preparation of these three reagents were based on the literature (**Reagent A**[7], **Reagent B**[8]**, Reagent C**[9]). The optimal conditions are shown in table S2.

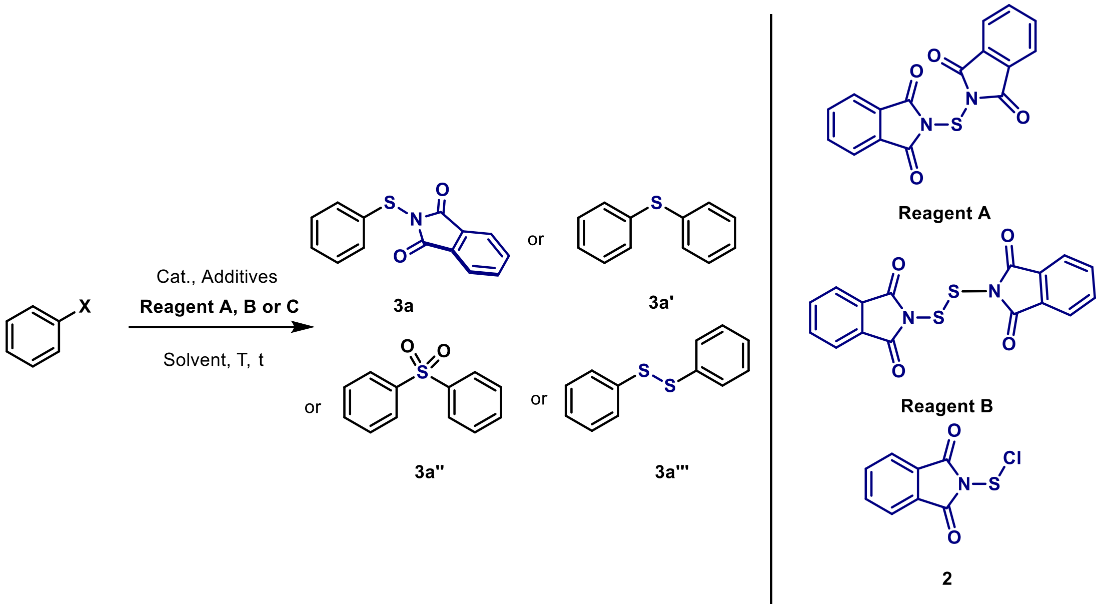

Table S2. Optimization for synthesis of Ph-S-Phth from aryl halides.

Entry	Aryl-X	Reagent	Cat. (%)	Add. (eq.)	Solvent	T (°C)	t (h)	Conv. (%)	Product ratio
1	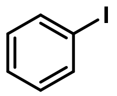	A	$CuSO_4$ (20)	$NaHCO_3$	DCM	60	24	trace	-
2		B	$CuCl_2$ (20)	Cs_2CO_3	DMF	80	24	trace	-
3[a]		A	CuI (20)	Bpy (20%)	DMSO/H_2O	80	24	54	**3a'':3a'** = 82:18
4[a]		B	CuCl (20)	Bpy (20%)	DMF	80	24	43	**3a'':3a'** = 65:35
5[b]	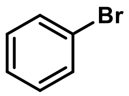	A	$Cu(OAc)_2$ (5)	-	THF/DCM	r.t.	24	43	**3a'':3a'** = 3:97
6[b]		B	$Cu(OAc)_2$ (5)	-	THF/DCM	r.t.	24	51	Only **3a'**
7[c]		**2**	$Cu(OAc)_2$ (5)	-	THF	r.t.	12	trace	-
8[c]		**2**	-	-	Et_2O	r.t.	2	90	Only **3a'**
9[b]		**2**	$Cu(OAc)_2$ (5)		THF	r.t.	2	84	**3a:3a':3a'''** = 30:54:16
10[b]		**2**	-	-	THF/DCM	0 - r.t.	1	>95	**3a:3a'''**=95:5

Conditions: [a] Open in the air; [b] bromobenzene was transformed to zinc reagent firstly and then mixed with S(II) reagent and catalyst; [c] Bromobenzene was transformed to Grignard reagent first and then mixed with S(II) reagent and catalyst.

5. Preparation of Ar-S-Phth

5.1 Preparation of 1M THF solution of $ZnCl_2$

The 1M THF solution of $ZnCl_2$ was prepared according to the literature[10]. $ZnCl_2$ powder (20 mmol) was dried at 140 °C under vacuum (10^{-3} mbar) for 6 h. After cooling to room temperature, 20 mL THF were added to dissolve the dry $ZnCl_2$. The suspension was kept stirring for 24 h until a clear colorless solution was obtained. The fresh-prepared $ZnCl_2$ should be kept over molecular sieves (4Å).

5.2 General method for preparation of organozinc halides from aryl halides

Method A: Under argon, activated Mg powder (12 mmol) and dry LiCl (10 mmol) were charged in a flame-dried 50 mL three-necked flask, which was equipped with a reflux condenser and a dropping funnel. After addition of a small amount (c.a. 2 mL) of aryl bromide solution (10 mmol in 8 mL THF), a few grains of iodine were added to initiate the reaction. The rest of aryl bromide solution was added dropwise over 0.5 h. Upon complete addition, the mixture was heated at 55 °C for 2 h. Subsequently, the $ZnCl_2$ (1M in THF, 12 mL) was added slowly into the mixture at 0 °C. Afterwards, the mixture was allowed to warm up to 25 ℃ and kept stirring for 1 h. A clear solution was obtained via filtration and the organozinc halides was titrated against iodine/LiCl before use. [11]

Method B: Under argon, aryl halides (3 mmol) was added into a flame-dried Schlenk flask and dissolved in dry THF (2 mL). When the reaction mixture was set to the appropriate temperature, *i*PrMgCl·LiCl (1.3 M in THF, 3.15 mmol) was added dropwise. The reaction was monitored by GC-MS (the reaction aliquots were quenched with saturated NH_4Cl aq. solution before analysis). Upon completion of the reaction, the $ZnCl_2$ (1M in THF, 3.6 mL) was added slowly into the mixture at 0 °C. Afterwards, the mixture was allowed to warm up to 25 ℃ and kept stirring for 1 h. The organozinc chlorides were titrated against iodine/LiCl before use. [10]

5.3 General method for synthesis of Phth-S-Cl

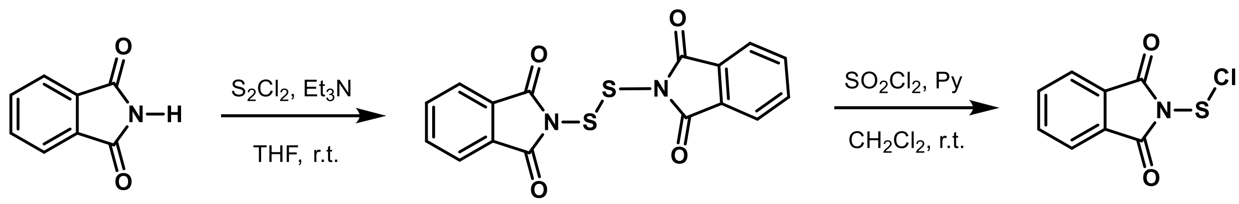

2

Phth-S-Cl (**2**) was prepared according to the literature[9]. Phthalimide (20 mmol) was dissolved in 40 mL THF followed by addition of trimethylamine (30 mmol). The mixture was then cooled to 0 ℃ and into that sulfur monochloride (S_2Cl_2, 10 mmol) was added slowly. The mixture was stirred for 2 h and quenched with water. Upon the completion of the reaction, the resulting precipitate was filtered and washed with diethyl ether (10 mL × 3). In order to purify the product, the resulting solid was re-dissolved in the solvent of $CHCl_3$/MeOH (v/v = 2:1,

25 mL) and refluxed for 1 h. After removal of the insoluble solid, the filtrate was concentrated under vacuum. The product di(1-phthalimidyl)disulfane was subsequently used for next step.

A flame-dried Schlenk flask was charged with 10 mL DCM solution of di(1-phthalimidyl)disulfane (2 mmol) and anhydrous pyridine (1.2 mmol) under argon. To the solution, fresh distilled sulfuryl chloride (SO_2Cl_2, 31 mmol) was added dropwise at room temperature. Then the mixture was stirred at the same temperature for 48 h. Upon the completion of the reaction, the solvent and excess SO_2Cl_2 were removed under vacuum, the resulting solid was collected, which is the desired Phth-S-Cl (two-step total yield: 63%). This reagent should be stored under argon.

5.4 General method for the synthesis of Ar-S-Phth

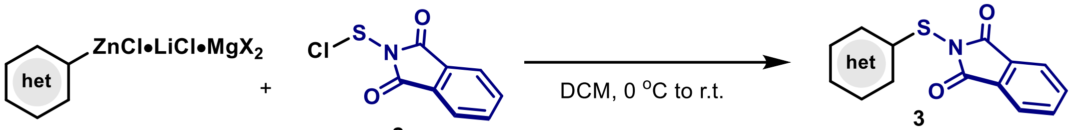

A flame-dried Schlenk flask was charged with Phth-S-Cl (102.2 mg, 0.48 mmol) and 2.5 mL DCM under argon. The mixture was subsequently cooled to 0 °C followed by addition of the THF solution of arylzinc chloride (0.4 mmol) over 10 min. The reaction was stirred at the same temperature for another 10 min and then allowed to warm up to 25 °C. The mixture was stirred for 30 min. During that time, the reaction was monitored by TLC. Upon the completion of reaction, the solvent was removed and the corresponding Ar-S-Phth was purified through a short silica column chromatography (Pentane/EtOAc: 3:1).

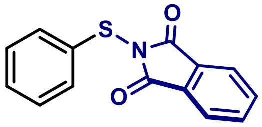

3a

Synthesis of N-(benzenesulfenyl)phthalimide (3a). The phenyl zinc chloride was prepared according to the **Method A** from bromobenzene **1a**. The title compound was prepared according to the general method for synthesis of Ar-S-Phth using 0.4 mmol phenyl zinc chloride (solution in THF) and 0.48 mmol Phth-S-Cl in DCM. After the reaction, purification through a column chromatography afforded **3a** as a white solid (82.6 mg, two-step yield 81%). NMR data are in accordance with the literature report [12].

^{1}H NMR (300 MHz, $CDCl_3$) δ 7.93 (dd, J = 5.5, 3.1 Hz, 2H), 7.78 (dd, J = 5.5, 3.1 Hz, 2H), 7.59 – 7.62 (m, 2H), 7.30 – 7.35 (m, 3H).

^{13}C NMR (75 MHz, $CDCl_3$) δ 167.7, 135.0, 134.7, 132.0, 130.9, 129.3, 124.1, 123.6.

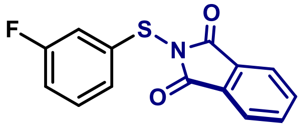

3b

Synthesis of N-(3-flourophenylsulfenyl)phthalimide (3b). The 3-fluorophenyl zinc chloride was prepared according to the **Method B** (the halogen-magnesium exchange underwent at r.t.) from 1-bromo-3-fluorobenzene **1b**. The title compound was prepared according to the general method for synthesis of Ar-S-Phth using 0.4 mmol 3-fluorophenyl zinc chloride (solution in THF) and 0.48 mmol Phth-S-Cl in DCM. After the reaction, purification through a column chromatography afforded **3b** as a white solid (87.3 mg, two-step yield 80%).

^{1}H NMR (300 MHz, $CDCl_3$) δ 7.96 (dd, *J* = 5.5, 3.1 Hz, 2H), 7.81 (dd, *J* = 5.5, 3.1 Hz, 2H), 7.28 – 7.31 (m, 2H), 7.2 (d, *J* = 7.5 Hz, 1H), 6.95 – 7.03 (m, 1H).
^{19}F{^{1}H} NMR (282 MHz, $CDCl_3$) δ -110.8 (s, 1F)
^{13}C NMR (75 MHz, $CDCl_3$) δ 167.4, 162.7 (d, *J* = 250.4 Hz), 137.3 (d, *J* = 7.5 Hz), 134.9, 131.9, 130.7 (d, *J* = 8.3 Hz), 125.0 (d, *J* = 3.2 Hz), 124.2, 116.4 (d, *J* = 23.2 Hz), 116.0 (d, *J* = 21.2 Hz).
HRMS (ESI): Calc'd for $C_{14}H_8FNO_2S^+$ $[M]^+$ 273.02543; found 273.02582

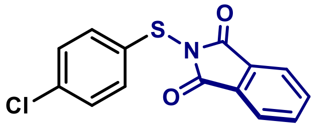

3c

Synthesis of N-(4-Cl-phenylsulfenyl)phthalimide (3c). The 4-chlorophenyl zinc chloride was prepared according to the **Method A** from 1-bromo-4-chlorobenzene **1c**. The title compound was prepared according to the general method for synthesis of Ar-S-Phth using 0.4 mmol 4-chlorophenyl zinc chloride (solution in THF) and 0.48 mmol Phth-S-Cl in DCM. After the reaction, purification through a column chromatography afforded **3c** as a white solid (93.2 mg, two-step yield 81%). NMR data are in accordance with the literature report [10].

^{1}H NMR (300 MHz, $CDCl_3$) δ 7.93 (dd, *J* = 5.5, 3.0 Hz, 2H), 7.79 (dd, *J* = 5.5, 3.0 Hz, 2H), 7.57 (d, *J* = 8.5 Hz, 2H), 7.30 (d, *J* = 8.5 Hz, 2H).
^{13}C NMR (75 MHz, $CDCl_3$) δ 167.5, 135.8, 134.8, 133.3, 132.8, 131.9, 129.5, 124.1.

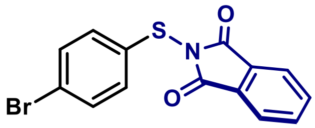

3d

Synthesis of N-(4-Br-phenylsulfenyl)phthalimide (3d). The 4-bromophenyl zinc chloride was prepared according to the **Method A** (the addition of $ZnCl_2$ solution at –20 °C) from 1-bromo-4-bromobenzene **1d**. The title compound was prepared according to the general method for synthesis of Ar-S-Phth using 0.4 mmol 4-bromophenyl zinc chloride (solution in THF) and 0.48 mmol Phth-S-Cl in DCM. After the reaction, purification through a column chromatography afforded **3d** as a white solid (113.2 mg, two-step yield 85%). NMR data are in accordance with the literature report [10].

^{1}H NMR (300 MHz, $CDCl_3$) δ 7.94 (dd, *J* = 5.5, 3.0 Hz, 2H), 7.79 (dd, *J* = 5.5, 3.0 Hz, 2H), 7.43 – 7.51 (m, 4H).
^{13}C NMR (75 MHz, $CDCl_3$) δ 167.5, 134.8, 134.0, 132.8, 132.5, 131.9, 124.1, 124.0.

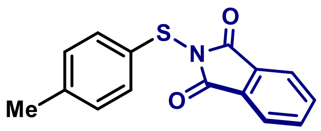

3e

Synthesis of N-(4-methylphenylsulfenyl)phthalimide (3e). The 4-methylphenyl zinc chloride was prepared according to the **Method A** from 4-bromotoluene **1e.** The title compound was prepared according to the general method for synthesis of Ar-S-Phth using 0.4 mmol 4-methylphenyl zinc chloride (solution in THF) and 0.48 mmol Phth-S-Cl in DCM. After the reaction, purification through a column chromatography afforded **3e** as a pale yellow solid (92.5 mg, two-step yield 86%). NMR data are in accordance with the literature report [13].

^{1}H NMR (300 MHz, $CDCl_3$) δ 7.83 (dd, *J* = 5.5, 3.1 Hz, 2H), 7.68 (dd, *J* = 5.5, 3.1 Hz, 2H), 7.52 (d, *J* = 7.9 Hz, 2H), 7.06 (d, *J* = 7.9 Hz, 2H), 3.24 (s, 3H).
^{13}C NMR (75 MHz, $CDCl_3$) δ 167.8, 140.3, 134.5, 132.6, 132.0, 131.4, 130.0, 123.9, 21.2.

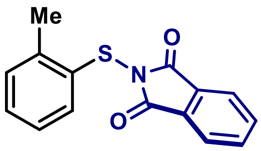

3f

Synthesis of N-(2-methylphenylsulfenyl)phthalimide (3f). The 2-methylphenyl zinc chloride was prepared according to the **Method A** from 2-bromotoluene **1f.** The title compound was prepared according to the general method for synthesis of Ar-S-Phth using 0.4 mmol 2-methylphenyl zinc chloride (solution in THF) and 0.48 mmol Phth-S-Cl in DCM. After the reaction, purification through a column chromatography afforded **3f** as a pale yellow solid (90.3 mg, two-step yield 83%). NMR data are in accordance with the literature report [14].

^{1}H NMR (300 MHz, $CDCl_3$) δ 7.93 (dd, *J* = 5.5, 3.1 Hz, 2H), 7.78 (dd, *J* = 5.5, 3.1 Hz, 2H), 7.42 (d, *J* = 7.6 Hz, 1H), 7.19 – 7.21 (m, 2H), 7.10 – 7.14 (m, 1H), 2.62 (s, 3H).

^{13}C NMR (75 MHz, $CDCl_3$) δ 166.8, 137.3, 133.6, 133.1, 131.0, 129.7, 129.6, 128.0, 125.8, 123.0, 19.1.

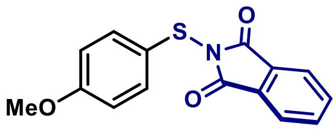

3g

Synthesis of N-(4-methoxyphenylsulfenyl)phthalimide (3g). The 4-methoxyphenyl zinc chloride was prepared according to the **Method A** from 4-bromoanisole **1g.** The title compound was prepared according to the general method for synthesis of Ar-S-Phth using 0.4 mmol 4-methoxyphenyl zinc chloride (solution in THF) and 0.48 mmol Phth-S-Cl in DCM. After the reaction, purification through a column chromatography afforded **3g** as a white solid (86.6 mg, two-step yield 76%). NMR data are in accordance with the literature report [13].

^{1}H NMR (300 MHz, $CDCl_3$) δ 7.89 (dd, *J* = 5.5, 3.0 Hz, 2H), 7.76 – 7.79 (m, 2H), 7.74 (dd, *J* = 5.5, 3.0 Hz, 2H), 6.85 (d, *J* = 8.9 Hz, 1H), 3.78 (s, 3H).

^{13}C NMR (75 MHz, $CDCl_3$) δ 167.8, 161.5, 136.6, 134.5, 132.1, 125.4, 123.9, 114.7, 55.4.

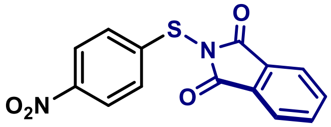

3h

Synthesis of N-(4-nitrophenylsulfenyl)phthalimide 3h. The 4-nitrophenyl zinc chloride was prepared according to the **Method A** from 1-bromonitrobenzene **1h.** The title compound was prepared according to the general method for synthesis of Ar-S-Phth using 0.4 mmol 4-nitrophenyl zinc chloride (solution in THF) and 0.48 mmol Phth-S-Cl in DCM. After the reaction, purification through a column chromatography afforded **3h** as a pale brown solid (73.2 mg, two-step yield 61%). NMR data are in accordance with the literature report [13].

^{1}H NMR (300 MHz, $CDCl_3$) δ 8.14 – 8.19 (m, 2H), 8.01 (dd, *J* = 5.5, 3.0 Hz, 2H), 7.87 (dd, *J* = 5.5, 3.0 Hz, 2H), 7.39 – 7.43 (m, 2H).

^{13}C NMR (75 MHz, $CDCl_3$) δ 167.0, 147.0, 144.6, 135.3, 131.7, 125.9, 124.5, 124.4.

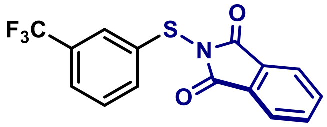

3i

Synthesis of N-(3-trifluoromethylphenylsulfenyl)phthalimide (3i). The 3-CF_3-phenyl zinc chloride was prepared according to the **Method B** (the halogen-magnesium exchange and addition of $ZnCl_2$ solution underwent at – 20 °C) from 1-iodo-3-trifluoromethylbenzene **1i.** The title compound was prepared according to the general method for synthesis of Ar-S-Phth using 0.4 mmol 3-CF_3-phenyl zinc chloride (solution in THF) and 0.48 mmol Phth-S-Cl in DCM. After the reaction, purification through a column chromatography afforded **3i** as a pale yellow solid (96.9 mg, two-step yield 75%).

^{1}H NMR (300 MHz, $CDCl_3$) δ 7.96 (br, 2H), 7.81 (br, 3H), 7.72 (d, J = 7.3 Hz, 1H), 7.56 (d, J = 7.3 Hz, 1H), 7.46 (t, J = 7.3 Hz, 1H).
^{19}F{^{1}H} NMR (282 MHz, $CDCl_3$) δ -62.85 (s, 3F)
^{13}C NMR (75 MHz, $CDCl_3$) δ 167.3, 136.5, 134.9, 133.1, 131.8, 131.7 (d, J_{C-F}= 32.2 Hz), 129.8, 126.6 (q, J_{C-F}= 3.5 Hz), 125.8 (q, J_{C-F}= 3.5 Hz), 124.2, 123.3 (d, J_{C-F}= 271.5 Hz).
HRMS (ESI): Calc'd for $C_{15}H_8F_3NO_2SNa^+$ $[M+Na]^+$ 346.01201; found 346.01188

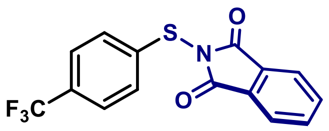

3j

Synthesis of N-(3-trifluoromethylphenylsulfenyl)phthalimide (3j). The 4-CF_3-phenyl zinc chloride was prepared according to the **Method B** (the halogen-magnesium exchange underwent at r.t.) from 1-bromo-4-trifluoromethylbenzene **1j.** The title compound was prepared according to the general method for synthesis of Ar-S-Phth using 0.4 mmol 4-CF_3-phenyl zinc chloride (solution in THF) and 0.48 mmol Phth-S-Cl in DCM. After the reaction, purification through a column chromatography afforded **3j** as a white solid (98.2 mg, two-step yield 76%). NMR data are in accordance with the literature report [14].

^{1}H NMR (300 MHz, $CDCl_3$) δ 7.97 (dd, J = 5.5, 3.1 Hz, 2H), 7.83 (dd, J = 5.5, 3.1 Hz, 2H), 7.50 – 7.58 (m, 4H).
^{19}F{^{1}H} NMR (282 MHz, $CDCl_3$) δ -62.83 (s, 3F)
^{13}C NMR (75 MHz, $CDCl_3$) δ 167.3, 140.2 (d, J_{C-F}= 1.5 Hz), 135.0, 131.8, 130.4 (d, J_{C-F}= 32.8 Hz), 128.0, 126.2 (q, J_{C-F}= 3.7 Hz), 124.3, 123.6 (d, J_{C-F}= 270.8 Hz).

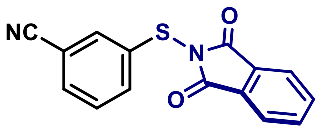

3k

Synthesis of N-(3-nitrilphenylsulfenyl)phthalimide (3k). The 3-CN-phenyl zinc chloride was prepared according to the **Method B** (the halogen-magnesium exchange underwent at 0 °C) from 3-bromobenzonitrile **1k.** The title compound was prepared according to the general method for synthesis of Ar-S-Phth using 0.4 mmol 4-

CN-phenyl zinc chloride (solution in THF) and 0.48 mmol Phth-S-Cl in DCM. After the reaction, purification through a column chromatography afforded **3k** as a white solid (88.5 mg, two-step yield 79%).

^{1}H NMR (300 MHz, $CDCl_3$) δ 7.98 (dd, *J* = 5.5, 3.1 Hz, 2H), 7.84 (dd, *J* = 5.5, 3.1 Hz, 2H), 7.72 – 7.75 (m, 2H), 7.57 (dt, *J* = 7.8 Hz, 1.3 Hz, 1H), 7.45 (t, *J* = 8.5, 1H).
^{19}F{^{1}H} NMR (282 MHz, $CDCl_3$) δ -62.83 (s, 3F)
^{13}C NMR (75 MHz, $CDCl_3$) δ 167.2, 137.4, 135.1, 133.2, 132.12, 132.09, 131.8, 130.0, 124.4, 117.7, 113.8.
HRMS (EI): Calc'd for $C_{15}H_8N_2O_2S^+$ $[M]^+$ (m/z) 280.03010; found 280.03053

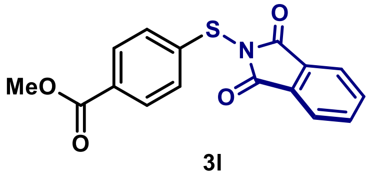

3l

Synthesis of N-((methyl4-benzoate)sulfenyl)phthalimide (3l). The 4-COOMe-phenyl zinc chloride was prepared according to the **Method B** (the halogen-magnesium exchange and addition of $ZnCl_2$ solution underwent at -30 °C) from 4-(carbomethoxy)iodobenzene **1l.** The title compound was prepared according to the general method for synthesis of Ar-S-Phth using 0.4 mmol 4-COOMe-phenyl zinc chloride (solution in THF) and 0.48 mmol Phth-S-Cl in DCM. After the reaction, purification through a column chromatography afforded **3l** as a white solid (100.1 mg, two-step yield 80%).

^{1}H NMR (300 MHz, $CDCl_3$) δ 7.95 – 8.00 (m, 4H), 7.83 (dd, *J* = 5.5, 3.1 Hz, 2H), 7.38 – 7.42 (m, 2H), 3.89 (s, 3H).
^{13}C NMR (75 MHz, $CDCl_3$) δ 167.3, 166.2, 141.4, 135.0, 131.8, 130.4, 129.6, 126.5, 124.3, 52.2.
HRMS (ESI): Calc'd for $C_{16}H_{11}NO_4SNa^+$ $[M+Na]^+$ 336.03010; found 336.03016

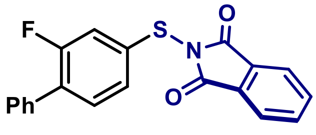

3m

Synthesis of N-((2-fluoro[1,1'-biphenyl]-4-yl)sulfenyl)phthalimide (3m). The 2-fluoro[1,1'-biphenyl]-4-yl zinc chloride was prepared according to the **Method A** from 4-bromo-2-fluoro1,1'-biphenyl **1m.** The title compound was prepared according to the general method for synthesis of Ar-S-Phth using 0.4 mmol 2-fluoro[1, 1'-biphenyl]-4-yl zinc chloride (solution in THF) and 0.48 mmol Phth-S-Cl in DCM. After the reaction, purification through a column chromatography afforded **3m** as a white solid (101.9 mg, two-step yield 73%).

^{1}H NMR (300 MHz, $CDCl_3$) δ 7.89 (dd, *J* = 5.5, 3.1 Hz, 2H), 7.73 (dd, *J* = 5.5, 3.1 Hz, 2H), 7.24 – 7.41 (m, 8H).

^{19}**F{^{1}H} NMR** (282 MHz, $CDCl_3$) δ -115.5 (s, 1F)

^{13}**C NMR** (75 MHz, $CDCl_3$) δ 167.4, 159.5 (d, $J_{C\text{-}F}$= 252.4 Hz), 135.8 (d, $J_{C\text{-}F}$= 7.8 Hz), 134.8, 134.3, 131.9, 131.4 (d, $J_{C\text{-}F}$= 4.0 Hz), 130.0 (d, $J_{C\text{-}F}$= 13.7 Hz), 128.9 (d, $J_{C\text{-}F}$= 3.0 Hz), 128.5, 128.1, 125.8 (d, $J_{C\text{-}F}$= 3.6 Hz), 124.2, 117.6 (d, $J_{C\text{-}F}$= 24.9 Hz).

HRMS (EI): Calc'd for $C_{20}H_{12}FNO_2S^+$ $[M]^+$ 349.05728; found 349.05718

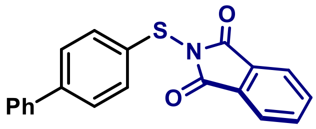

3n

Synthesis of N-(([1,1'-biphenyl]-4-yl)sulfenyl)phthalimide (3n). The [1,1'-biphenyl]-4-yl zinc chloride was prepared according to the **Method A** from 4-bromo-1,1'-biphenyl **1n.** The title compound was prepared according to the general method for synthesis of Ar-S-Phth using 0.4 mmol 1,1'-biphenyl]-4-yl zinc chloride (solution in THF) and 0.48 mmol Phth-S-Cl in DCM. After the reaction, purification through a column chromatography afforded **3n** as a white solid (100.7 mg, two-step yield 77%).

^{1}**H NMR** (300 MHz, $CDCl_3$) δ 7.94 (dd, J = 5.5, 3.1 Hz, 2H), 7.78 (dd, J = 5.5, 3.1 Hz, 2H), 7.70 (d, J = 8.4 Hz, 2H), 7.51 – 7.55 (m, 4H), 7.32 – 7.44 (m, 3H).

^{13}**C NMR** (75 MHz, $CDCl_3$) δ 167.7, 142.5, 139.9, 134.7, 133.8, 132.0, 131.8, 128.8, 128.0, 127.8, 127.1, 124.0.

HRMS (ESI): Calc'd for $C_{20}H_{13}NO_2SNa^+$ $[M+Na]^+$ 354.05592; found 354.05575

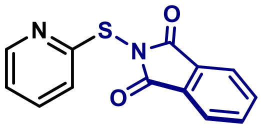

3o

Synthesis of N-(2-pyridinylsulfenyl)phthalimide (3o). The 2-pyridinyl zinc chloride was prepared according to the **Method B** (the halogen-magnesium exchange underwent at r.t.) from 2-bromopyridine **1o.** The title compound was prepared according to the general method for synthesis of Ar-S-Phth using 0.4 mmol 2-pyridinyl zinc chloride (solution in THF) and 0.48 mmol Phth-S-Cl in DCM. After the reaction, purification through a column chromatography afforded **3o** as a white solid (74.7 mg, two-step yield 73%). NMR data are in accordance with the literature report [15].

^{1}**H NMR** (300 MHz, $CDCl_3$) δ 8.30 (d, J = 4.8 Hz, 1H), 8.00 (dd, J = 5.4, 3.1 Hz, 2H), 7.84 (dd, J = 5.4, 3.1 Hz, 2H), 7.56 (t, J = 8.4 Hz, 1H), 7.13 (d, J = 8.4 Hz, 1H), 7.02 (dd, J = 7.2, 4.8 Hz, 1H). ^{13}**C NMR** (75 MHz, $CDCl_3$) δ 167.6, 156.4, 150.0, 136.8, 134.7, 132.2, 124.2, 121.0, 118.1.

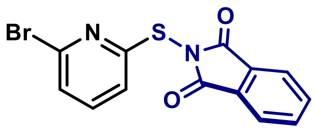

3p

Synthesis of N-(6-bromo-2-pyridinylsulfenyl)phthalimide (3p). The 6-bromo-2-pyridinyl zinc chloride was prepared according to the **Method B** (the halogen-magnesium exchange underwent at 0 °C) from 2,6-dibromopyridine **1p.** The title compound was prepared according to the general method for synthesis of Ar-S-Phth using 0.4 mmol 6-bromo-2-pyridinyl zinc chloride (solution in THF) and 0.48 mmol Phth-S-Cl in DCM. After the reaction, purification through a column chromatography afforded **3p** as a pale yellow solid (106.5 mg, two-step yield 80%).

^{1}H NMR (300 MHz, $CDCl_3$) δ 8.02 (dd, *J* = 5.4, 3.0 Hz, 2H), 7.85 (dd, *J* = 5.4, 3.0 Hz, 2H), 7.40 (t, *J* = 7.8 Hz, 1H), 7.20 (d, *J* = 7.8 Hz, 1H), 7.05 (d, *J* = 7.8 Hz, 1H).

^{13}C NMR (75 MHz, $CDCl_3$) δ 167.3, 157.6, 142.0, 139.0, 135.0, 132.1, 125.2, 124.3, 116.5.

HRMS (ESI): Calc'd for $C_{13}H_7BrN_2O_2SNa^+$ $[M+Na]^+$ 356.93039; found 356.93032

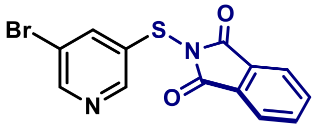

3q

Synthesis of N-(5-bromo-3-pyridinylsulfenyl)phthalimide (3q). The 5-bromo-3-pyridinyl zinc chloride was prepared according to the **Method B** (the halogen-magnesium exchange underwent at 0 °C) from 3,5-dibromopyridine **1q.** The title compound was prepared according to the general method for synthesis of Ar-S-Phth using 0.4 mmol 5-bromo-3-pyridinyl zinc chloride (solution in THF) and 0.48 mmol Phth-S-Cl in DCM. After the reaction, purification through a column chromatography afforded **3q** as a pale yellow solid (94.5 mg, two-step yield 71%).

^{1}H NMR (300 MHz, $CDCl_3$) δ 8.68 (d, *J* = 2.0 Hz, 1H), 8.55 (d, *J* = 2.0 Hz, 1H), 8.01 (t, *J* = 2.0 Hz, 1H), 7.89 (dd, *J* = 5.5, 3.1 Hz, 2H), 7.75 (dd, *J* = 5.5, 3.1 Hz, 2H).

^{13}C NMR (75 MHz, $CDCl_3$) δ 167.1, 156.7, 151.5, 149.4, 140.9, 135.0, 133.4, 131.8, 124.3.

HRMS (ESI): Calc'd for $C_{13}H_8BrN_2O_2S^+$ $[M+H]^+$ 334.94845; found 334.94873

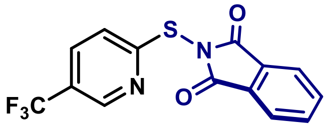

3r

Synthesis of N-(5-trifluoromethyl-2-pyridinylsulfenyl)phthalimide (3r). The 5-trifluoromethyl-2-pyridinyl zinc chloride was prepared according to the **Method B** (the halogen-magnesium exchange underwent at - 60 °C) from 2-iodo-5-(trifluoromethyl)pyridine **1r.** The title compound was prepared according to the general method for synthesis of Ar-S-Phth using 0.4 mmol 5-trifluoromethyl-2-pyridinyl zinc chloride (solution in THF) and 0.48 mmol Phth-S-Cl in DCM. After the reaction, purification through a column chromatography afforded **3r** as a white solid (89.4 mg, two-step yield 69%). NMR data are in accordance with the literature report [13].

^{1}H NMR (300 MHz, $CDCl_3$) δ 8.55 (dt, *J* = 2.3, 0.9 Hz, 1H), 8.02 (dd, *J* = 5.5, 3.0 Hz, 2H), 7.86 (dd, *J* = 5.5, 3.0 Hz, 2H), 7.79 (ddd, *J* = 8.4, 2.3, 0.9 Hz, 1H), 7.27 (dt, *J* =8.4, 0.9 Hz, 1H).
^{19}F{^{1}H} NMR (282 MHz, $CDCl_3$) δ -62.4 (s, 3F)
^{13}C NMR (75 MHz, $CDCl_3$) δ 167.2, 161.1, 146.9 (q, *J* = 4.2 Hz), 134.9, 133.8 (q, *J* = 3.4 Hz), 132.1, 124.3, 124.2 (d, *J* = 33 Hz), 123.3 (d, *J* = 270 Hz), 117.6.

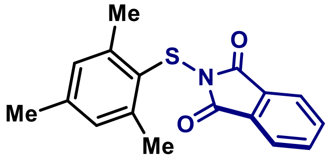

3v

Synthesis of N-(2,4,6-trimethyl-phenylsulfenyl)phthalimide (3v). The 2,4,6-trimethyl-phenyl zinc chloride was prepared according to the **Method A** from 2-bromo-1,3,5-trimethylbenzene **1v.** The title compound was prepared according to the general method for synthesis of Ar-S-Phth using 0.4 mmol 2,4,6-trimethyl-phenyl zinc chloride (solution in THF) and 0.48 mmol Phth-S-Cl in DCM. After the reaction, purification through a column chromatography afforded **3v** as a white solid (103.3 mg, two-step yield 87%).

^{1}H NMR (300 MHz, $CDCl_3$) δ 7.83 (dd, *J* = 5.5, 3.1 Hz, 2H), 7.72 (dd, *J* = 5.5, 3.1 Hz, 2H), 6.94 (s, 2H), 2.72 (s, 6H), 2.25 (s, 3H).
^{13}C NMR (75 MHz, $CDCl_3$) δ 168.0, 144.2, 141.1, 134.4, 132.1, 129.4, 128.6, 123.6, 22.2, 21.1.
HRMS (GC-EI): Calc'd for $C_{17}H_{15}NO_2S^+$ $[M]^+$ (m/z): 297.08180; found 297.08199

6. Optimization of oxyfluorination step

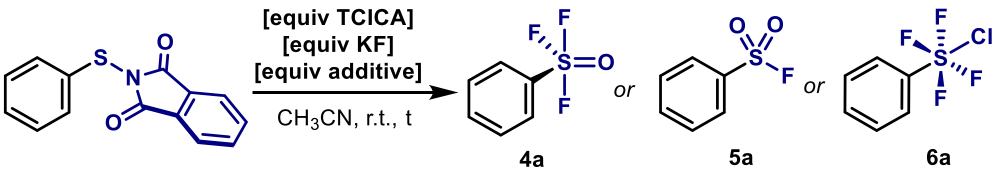

Entry	TCICA (eq.)	KF (eq.)	Additive (eq.)	Time (h)	Yield (%) (4a+5a+6a)	Ratio (4a:5a:6a)	Note
1	9	16	TFA (2)	24	>95	96:4:<0.1	Optimized condition for synthesis of $ArSOF_3$
2	9	16	TFA (1)	24	71	65:28:17	
3	9	16	TFA (0.5)	24	58	34:28:38	
4	9	16	TFA (0.2)	24	60	35:<0.1:65	
5	9	16	TFA (0.1)	24	72	21:<0.1:79	
6	9	16	-	24	35	44:3:53	18% $PhSF_3$ formed
7	9	16	-	48	51	43:1:56	12% $PhSF_3$ formed
8	9	16	AcOH (2)	24	64	<0.1:99:<0.1	
9	9	16	H_2O (2)	24	56	<0.1:99:<0.1	
10	9	16	MeOH (2)	24	>95	2:98:<0.1	Optimized condition for synthesis of $ArSO_2F$
11	9	16	EtOH (2)	24	>95	2:98:<0.1	
12	9	16	$ZnCl_2$ (0.1)	24	44	50:6:44	42% $PhSF_3$ formed
13	4.5	8	TFA (0.1)	9	24	32:<0.1:68	15% $PhSF_3$ formed
14	18	32	TFA (0.1)	24	87	18:<0.1:82	
15	18	32	TFA (0.1)	24	>95	12:2:85	With 0.2 mmol SM. Optimized condition for synthesis of $ArSF_4Cl$
16	18	32	TFA (2)	24	76	92:8:<0.1	
17	9*	16	TFA (0.1)	24	0	-	*Cyanuryl chloride instead of TCICA
18	-	-	TFA (0.1)	24	0	-	2 bar SF_6 gas was used
19	9	16	TFA (2)	24	24	14:86:<0.1	DCM was used as solvent
20	9	16	TFA(2)	24	0	-	THF was used as solvent

Reaction conditions: **3a** (0.1 mmol), TCICA, KF and additives in 1 mL MeCN at room temperature; ^{19}F NMR yields, α, α, α-trifluorotoluene as the internal standard.

7. General method for the synthesis of $ArSOF_3$

In a glovebox under Argon, the Ar-S-Phth precursor (**3**, 0.1 mmol, 1 equiv.), trichloroisocyanuric acid (TCICA, 210 mg, 0.9 mmol, 9 equiv.) and rigorously dried KF (93 mg, 16 mmol, 16 equiv.) was added to an oven-dried 12 mL round-bottom reaction vial equipped with a stir bar. Under vigorous stirring, 1 mL dry and degassed MeCN was added to the mixture followed by addition of TFA solution (1M in MeCN, 0.2 mL). Then the vial was sealed with a tight cap and the reaction was stirred at the room temperature in the glovebox for 24 h. Upon the completion of reaction, the atmosphere in the vial was vented carefully and the internal standard α,α,α-trifluorotoluene was added into the mixture. After 10 min stirring, an aliquot of the resulting solution was filtered under argon. The NMR sample was prepared with 0.4 mL of the filtered aliquot and 0.1 mL CD_3CN for ^{19}F NMR yield determination.

Please note, the $ArSOF_3$ is extremely sensitive to the moisture and even glass. All reaction vials, flasks and NMR tubes used for the reaction and work-up process should be dried rigorously. Solvent, including deuterated solvent, should be ultra-dry (water content should be at least below 15 ppm). The reaction and work-up process should be handled always under argon. Interestingly, the $ArSOF_3$ can remain stable in the resulting solution after reaction for at least 4 days.

8. Scope of $ArSOF_3$

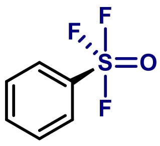

4a

Phenylsulfur(VI) trifluoridoxide **4a** was prepared according to the general method in section 7.

^{19}F{^{1}H} NMR (282 MHz, CH_3CN/CD_3CN) δ 100.6 (d, J = 157.9 Hz, 2F), 64.5 (t, J = 157.9 Hz, 1F) (due to the (pseudo) trigonal-bipyramidal coordination, ^{19}F NMR spectrum shows AB_2 pattern, which will be discussed in detail in section 9.)

^{19}F NMR spectra (282 MHz)

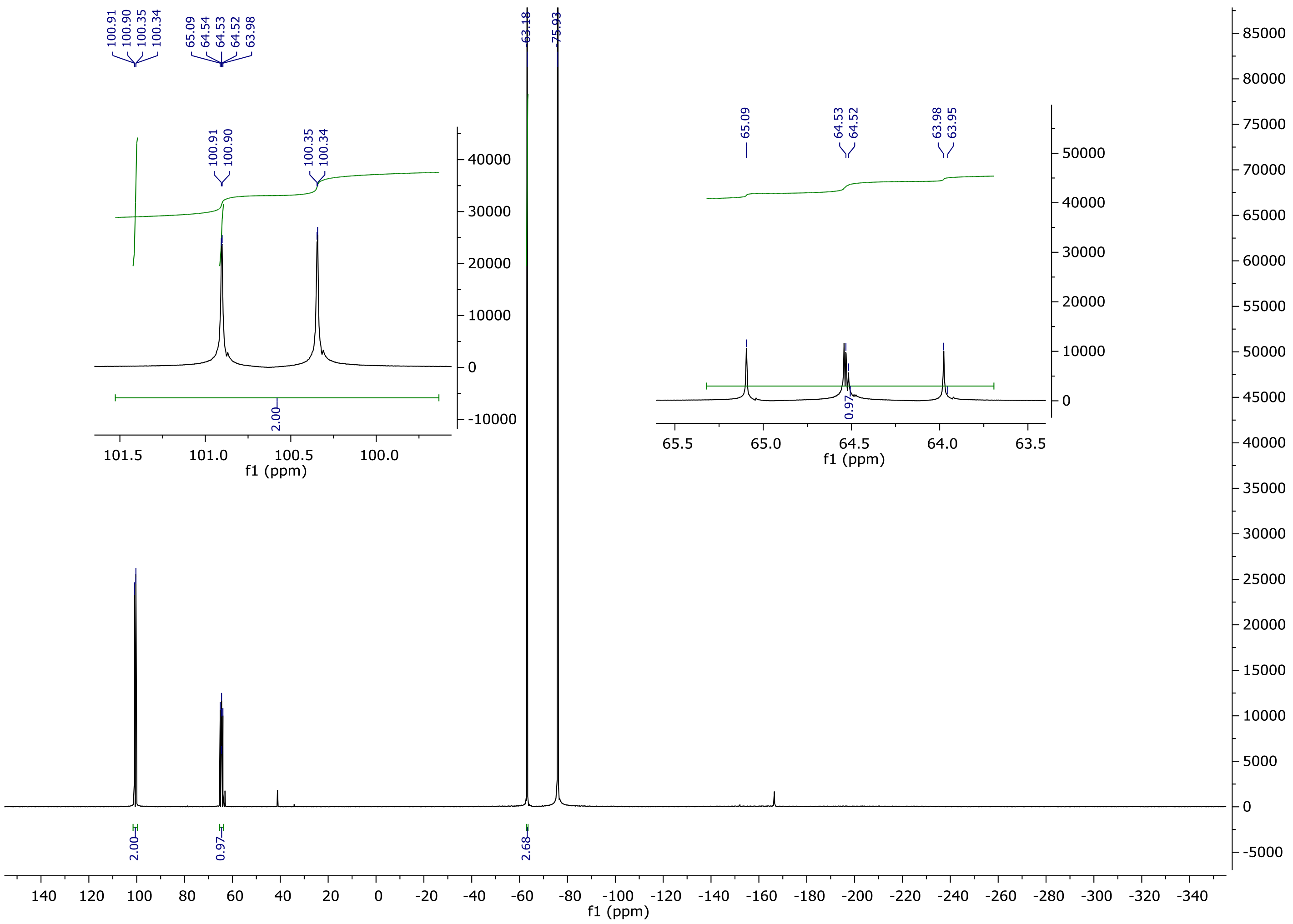

Note. Based on internal standard α,α,α-trifluorotoluene (at –63.18 ppm, 11.1 mg, 0.076 mmol), ^{19}F NMR yield: 85%. In combination with the yield for **3a**, the three-step total yield: 70%. Singlet at 64.52 ppm is the signal of $PhSO_2F$ (**5a**); Singlet at –75.93 ppm is the signal of excess TFA.

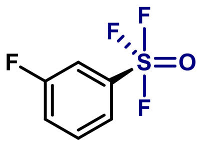

4b

3-Flourophenylsulfur(VI) trifluoridoxide **4b** was prepared according to the general method in section 7.

^{19}F{^{1}H} NMR (282 MHz, CH_3CN/CD_3CN) δ 101.6 (d, *J* = 160.5 Hz, 2F), 65.4 (t, *J* = 160.5 Hz, 1F), -109.5 (s, 1F) (due to the (pseudo) trigonal-bipyramidal coordination, ^{19}F NMR spectrum shows AB_2 pattern, which will be discussed in detail in section 9).

^{19}F NMR spectra (282 MHz)

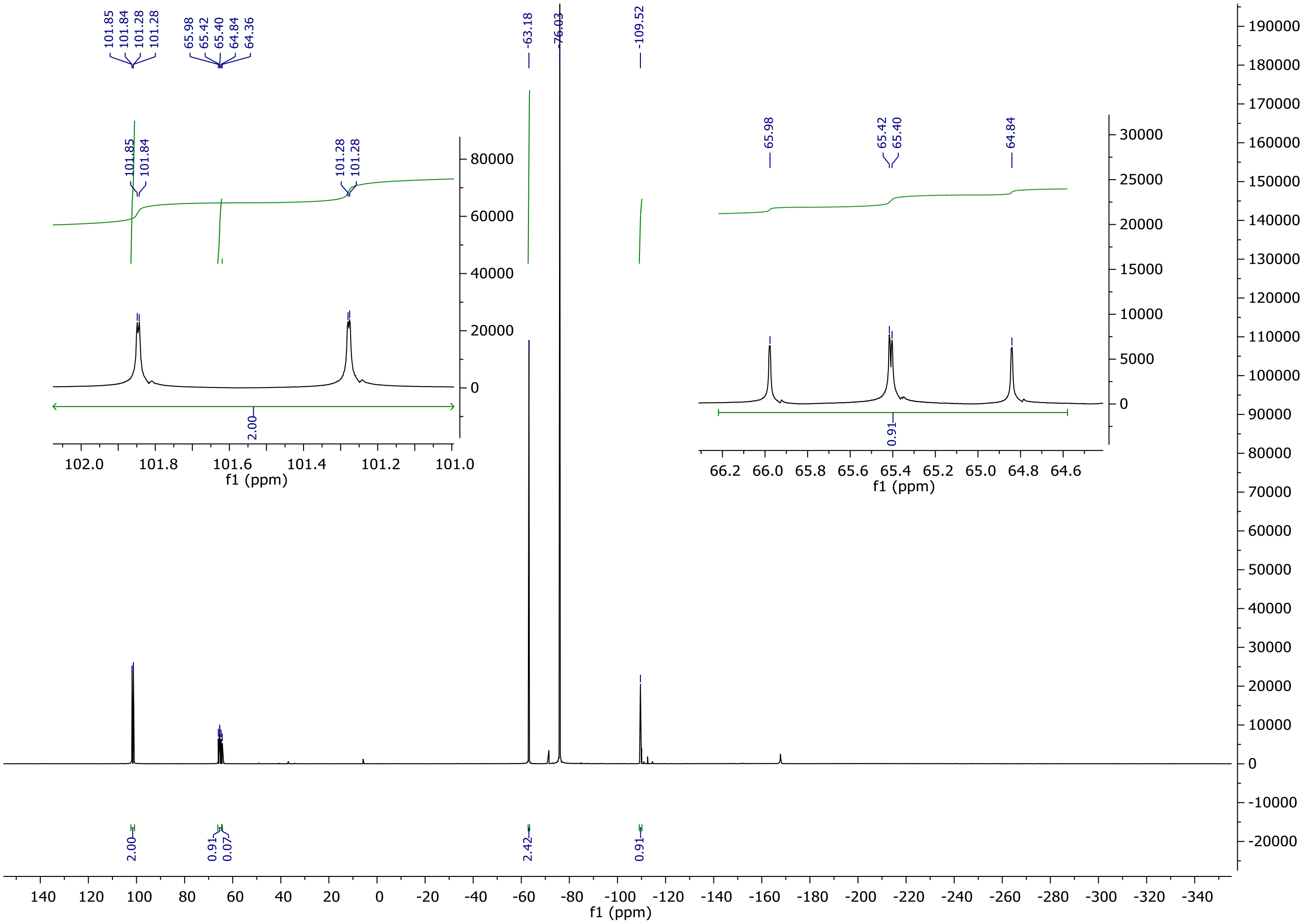

Note. Based on internal standard α,α,α-trifluorotoluene (at –63.18 ppm, 10.6 mg, 0.073 mmol), ^{19}F NMR yield: 90%. In combination with the yield for **3b**, the three-step total yield: 72%. Singlet at 64.36 ppm is the signal of 3-F-$PhSO_2F$; Singlet at –76.03 ppm is the signal of excess TFA.

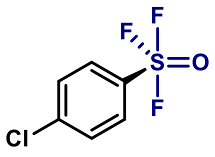

4c

4-chlorophenylsulfur(VI) trifluoridoxide **4c** was prepared according to the general method in section 7.

^{19}F{^{1}H} NMR (282 MHz, CH_3CN/CD_3CN) δ 100.5 (d, *J* = 159.5 Hz, 2F), 66.2 (t, *J* = 159.5 Hz, 1F) (due to the (pseudo) trigonal-bipyramidal coordination, ^{19}F NMR spectrum shows AB_2 pattern, which will be discussed in detail in section 9).

^{19}F NMR spectra (282 MHz)

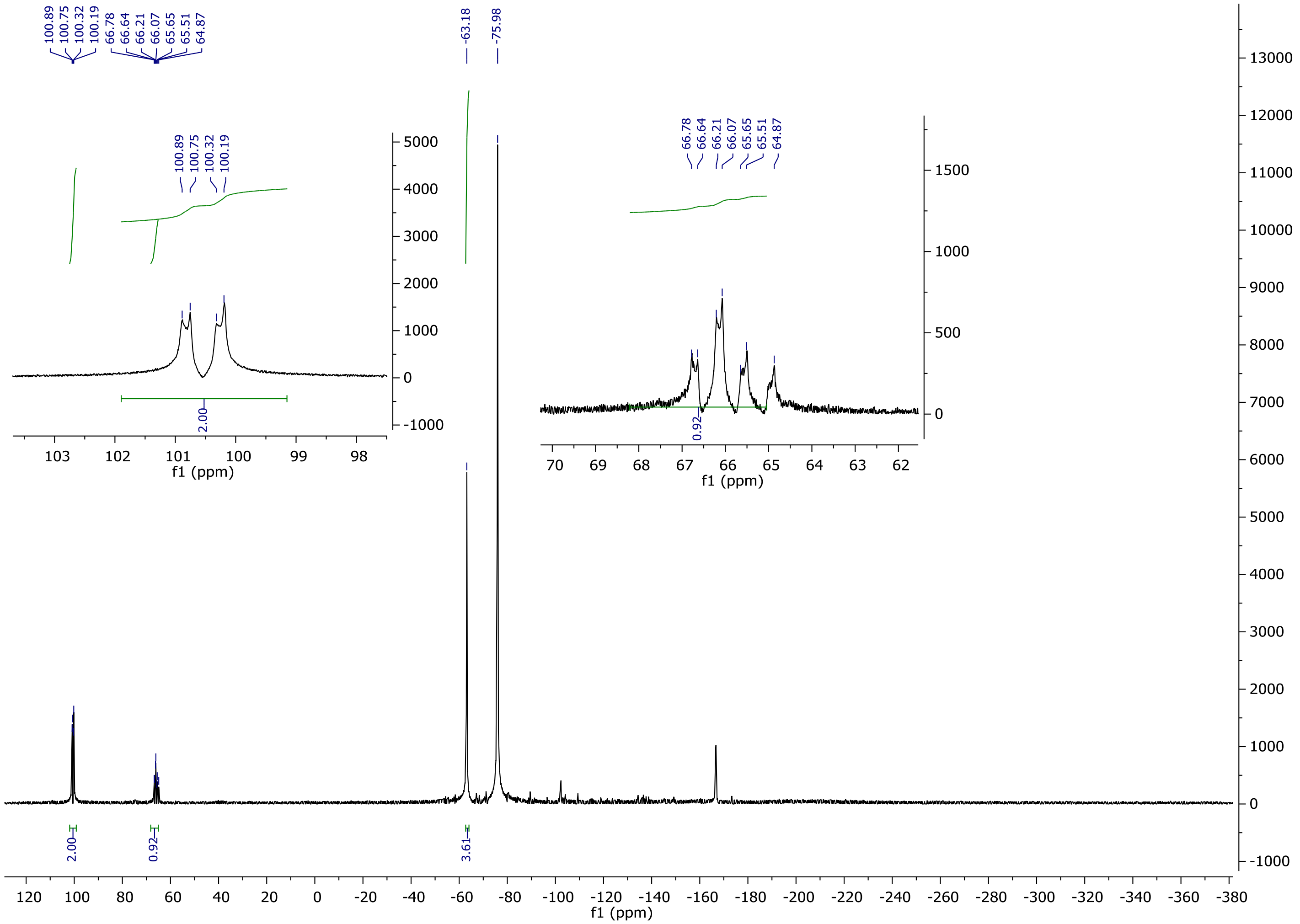

Note. Based on internal standard α,α,α-trifluorotoluene (at –63.18 ppm, 14.2 mg, 0.097 mmol), ^{19}F NMR yield: 81%. In combination with the yield for **3c**, the three-step total yield: 65%. Singlet at 64.87 ppm is the signal of 4-Cl-$PhSO_2F$ (**5c**); Singlet at –75.98 ppm is the signal of excess TFA.

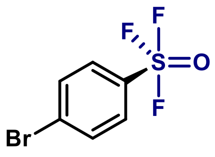

4d

4-bromophenylsulfur(VI) trifluoridoxide **4d** was prepared according to the general method in section 7.

^{19}F{^{1}H} NMR (282 MHz, CH_3CN/CD_3CN) δ 100.6 (d, *J* = 159.5 Hz, 2F), 65.9 (t, *J* = 159.5 Hz, 1F) (due to the (pseudo) trigonal-bipyramidal coordination, ^{19}F NMR spectrum shows AB_2 pattern, which will be discussed in detail in section 9).

^{19}F NMR spectra (282 MHz)

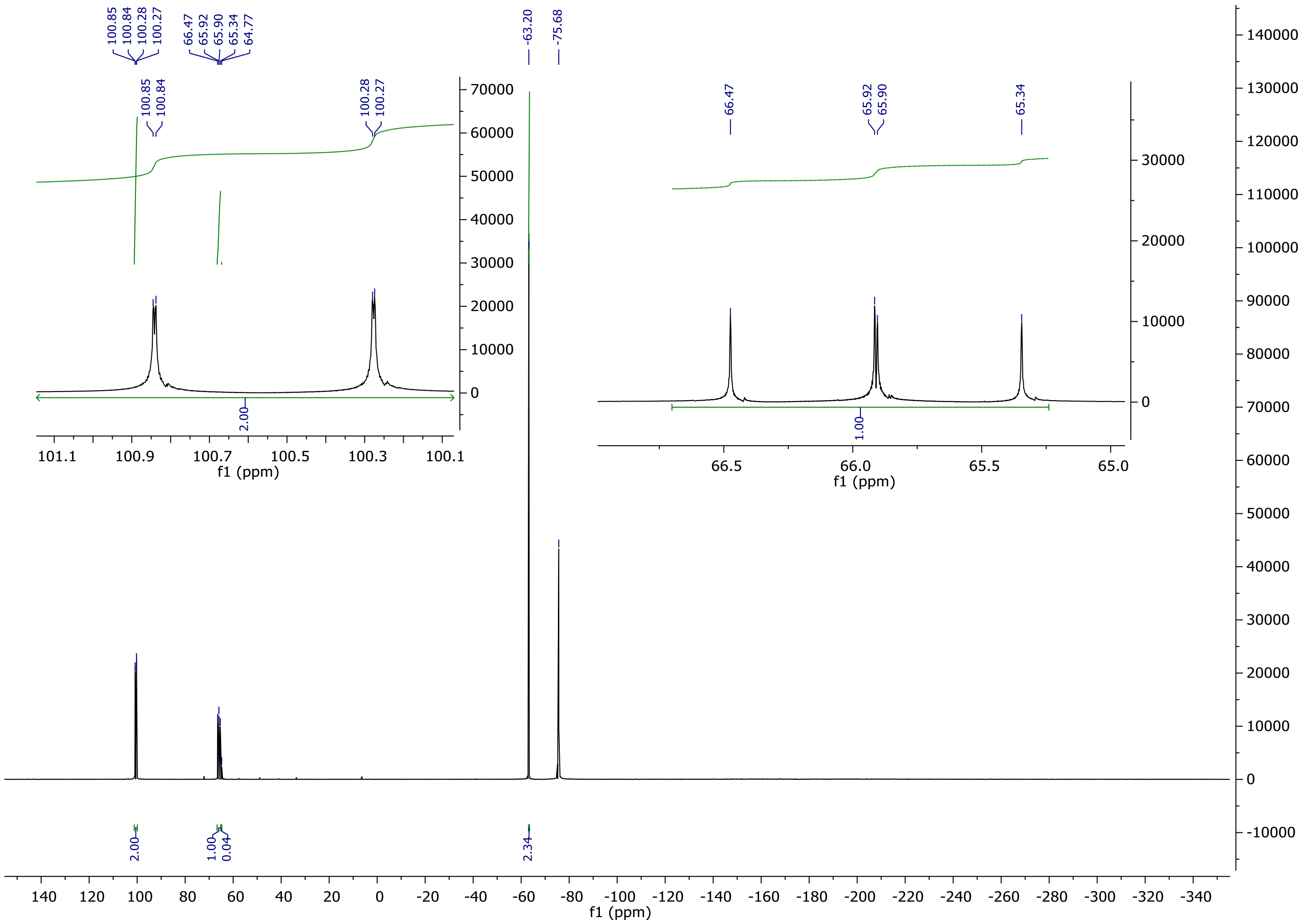

Note. Based on internal standard α,α,α-trifluorotoluene (at –63.20 ppm, 10.2 mg, 0.070 mmol), ^{19}F NMR yield: 90%. In combination with the yield for **3d**, the three-step total yield: 77%. Singlet at 64.77 ppm is the signal of 4-Br-$PhSO_2F$ (**5b**); Singlet at –75.68 ppm is the signal of excess TFA.

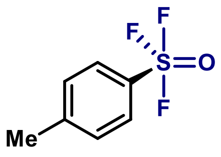

4e

4-methylphenylsulfur(VI) trifluoridoxide **4e** was prepared according to the general method in section 7.

^{19}F{^{1}H} NMR (282 MHz, CH_3CN/CD_3CN) δ 99.3 (d, *J* = 156.7 Hz, 2F), 65.6 (t, *J* = 156.7 Hz, 1F) (due to the (pseudo) trigonal-bipyramidal coordination, ^{19}F NMR spectrum shows AB_2 pattern, which will be discussed in detail in the section 9).

^{19}F NMR spectra (282 MHz)

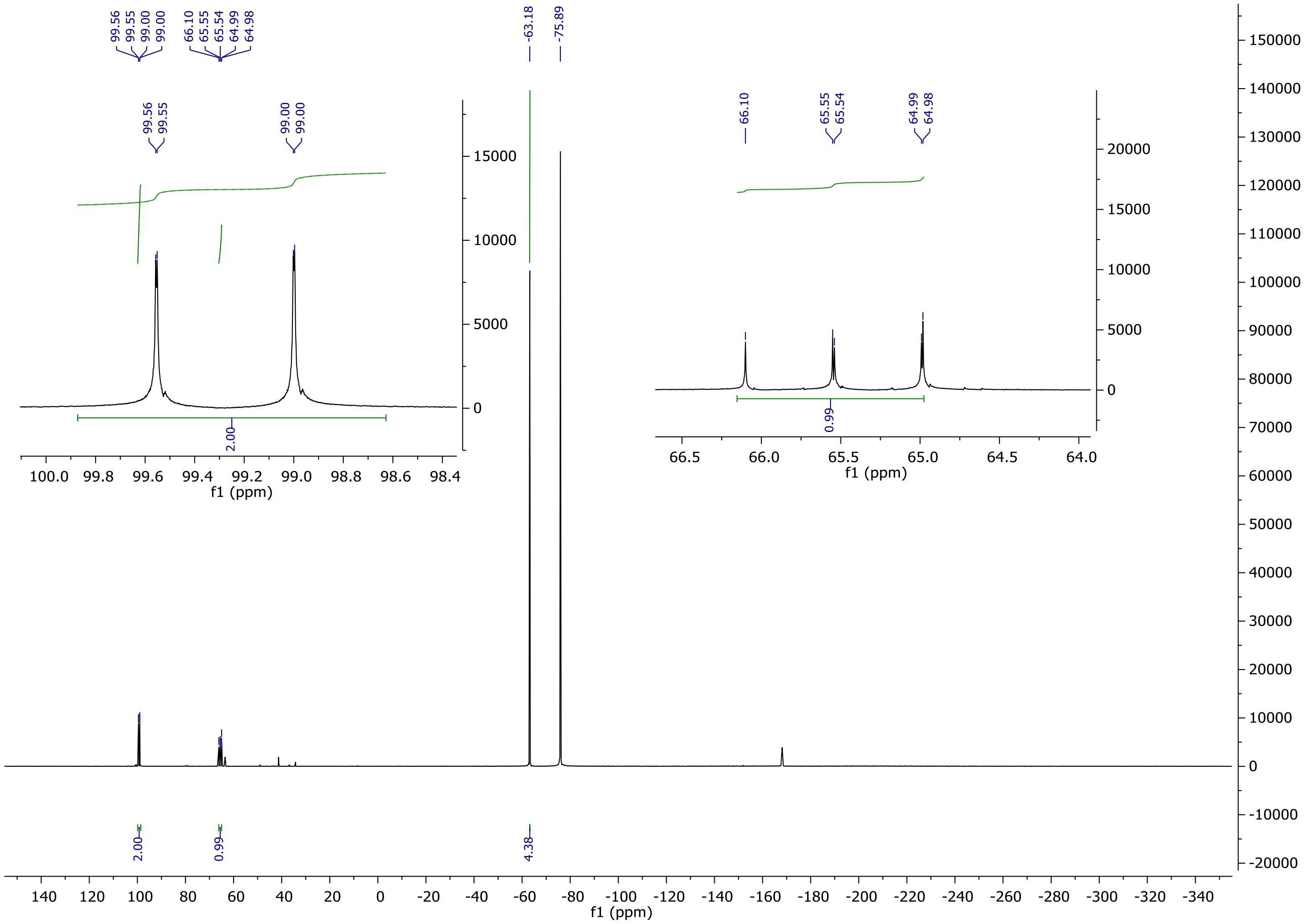

Note. Based on internal standard α,α,α-trifluorotoluene (at –63.18 ppm, 12.3 mg, 0.084 mmol), ^{19}F NMR yield: 58%. In combination with the yield for **3e**, the three-step total yield: 50%. Singlet at 64.98 ppm is the signal of 4-Me-$PhSO_2F$ (**5d**); Singlet at –75.89 ppm is the signal of excess TFA.

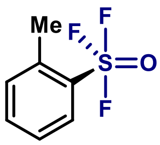

4f

2-methylphenylsulfur(VI) trifluoridoxide **4f** was prepared according to the general method in section 7.

^{19}F{^{1}H} NMR (282 MHz, CH_3CN/CD_3CN) δ 103.4 (d, *J* = 158.8 Hz, 2F), 69.0 (t, *J* = 156.7 Hz, 1F) (due to the (pseudo) trigonal-bipyramidal coordination, ^{19}F NMR spectrum should show AB_2 pattern. But because of the small amount of product, the peak became broad).

^{19}F NMR spectra (282 MHz)

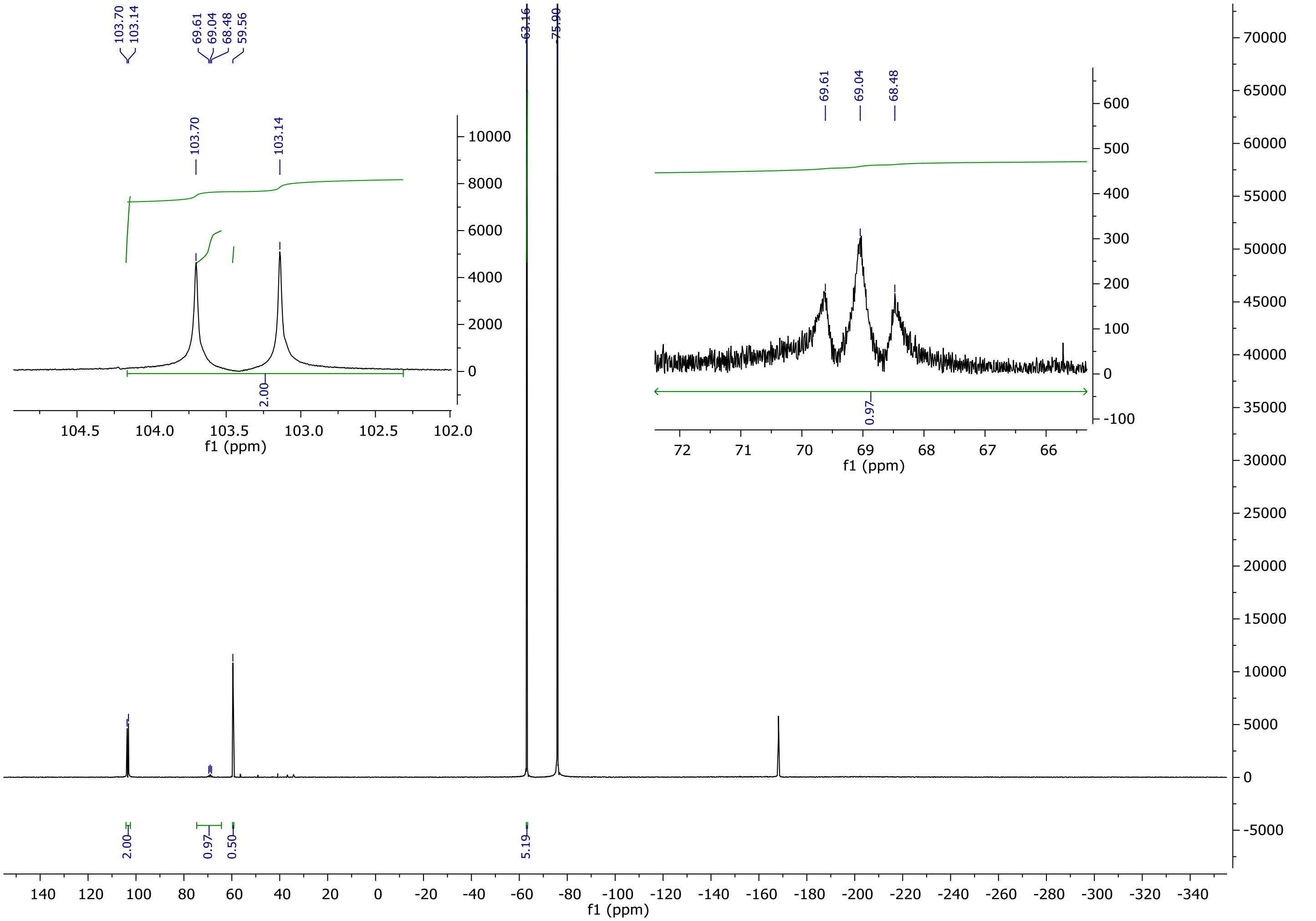

Note. Based on internal standard α,α,α-trifluorotoluene (at –63.16 ppm, 10.3 mg, 0.071 mmol), ^{19}F NMR yield: 41%. In combination with the yield for **3f**, the three-step total yield: 34%. Singlet at 59.6 ppm is the signal of 2-Me-$PhSO_2F$; Singlet at –75.90 ppm is the signal of excess TFA.

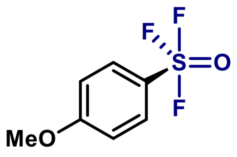

4g

4-methoxyphenylsulfur(VI) trifluoridoxide **4g** was prepared according to the general method in section 7.

^{19}F{^{1}H} NMR (282 MHz, CH_3CN/CD_3CN) δ 97.7 (d, J = 158.3 Hz, 2F), 68.2 (t, J = 158.3 Hz, 1F) (due to the (pseudo) trigonal-bipyramidal coordination, ^{19}F NMR spectrum should show AB_2 pattern. But because of the small amount of product, the peak became broad).

^{19}F NMR spectra (282 MHz)

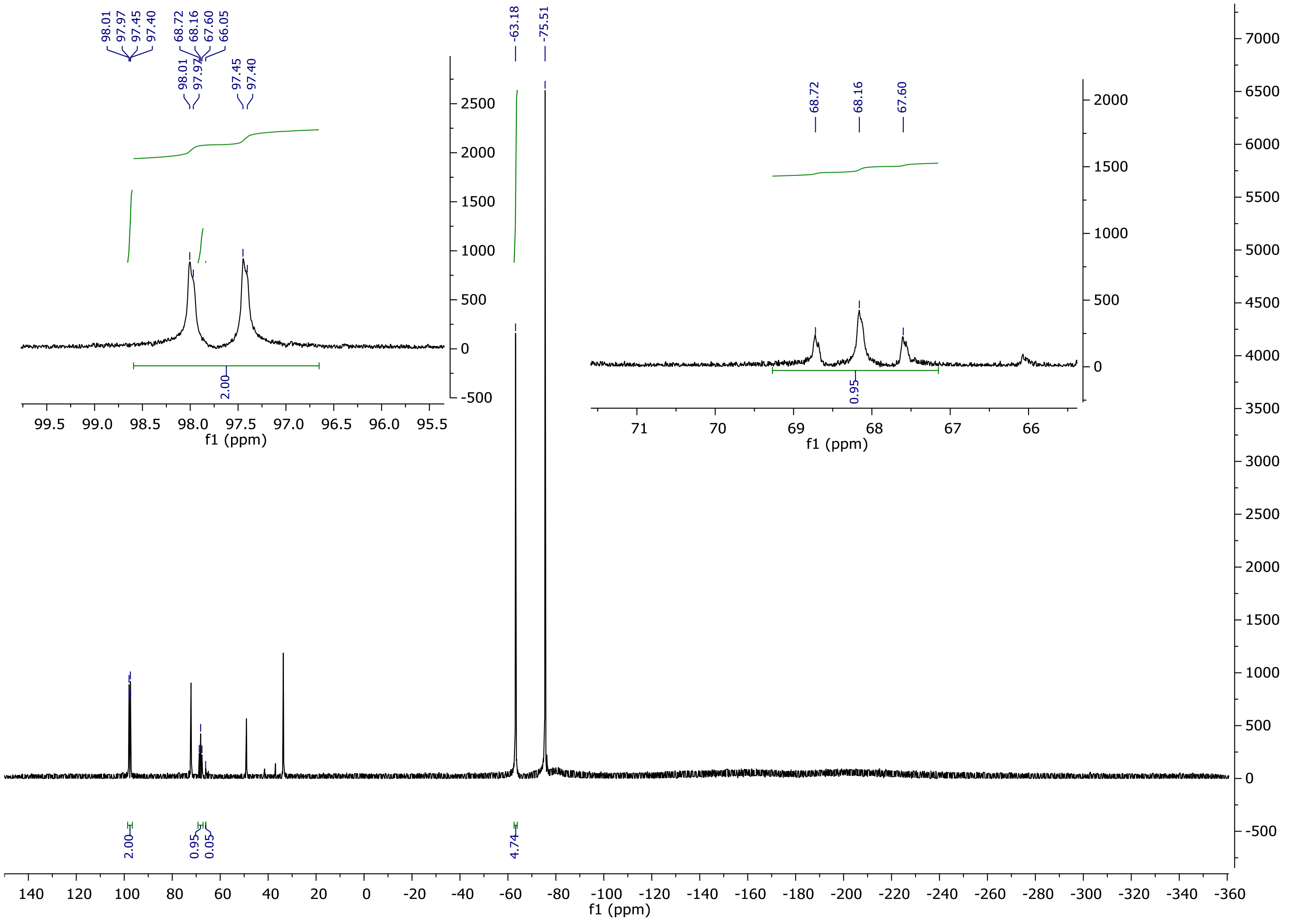

Note. Based on internal standard α,α,α-trifluorotoluene (at –63.18 ppm, 8.3 mg, 0.057 mmol), ^{19}F NMR yield: 36%. In combination with the yield for **3g**, the three-step total yield: 27%. Other signals are complicated; Singlet at –75.51 ppm is the signal of excess TFA.

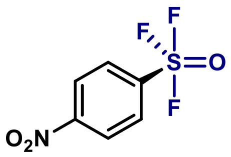

4h

4-nitrophenylsulfur(VI) trifluoridoxide **4h** was prepared according to the general method in section 7.

^{19}F{^{1}H} NMR (282 MHz, CH_3CN/CD_3CN) δ 102.8 (d, *J* = 162.3 Hz, 2F), 65.2 (t, *J* = 162.3 Hz, 1F) (due to the (pseudo) trigonal-bipyramidal coordination, ^{19}F NMR spectrum shows AB_2 pattern, which will be discussed in detail in the section 9).

^{19}F NMR spectra (282 MHz)

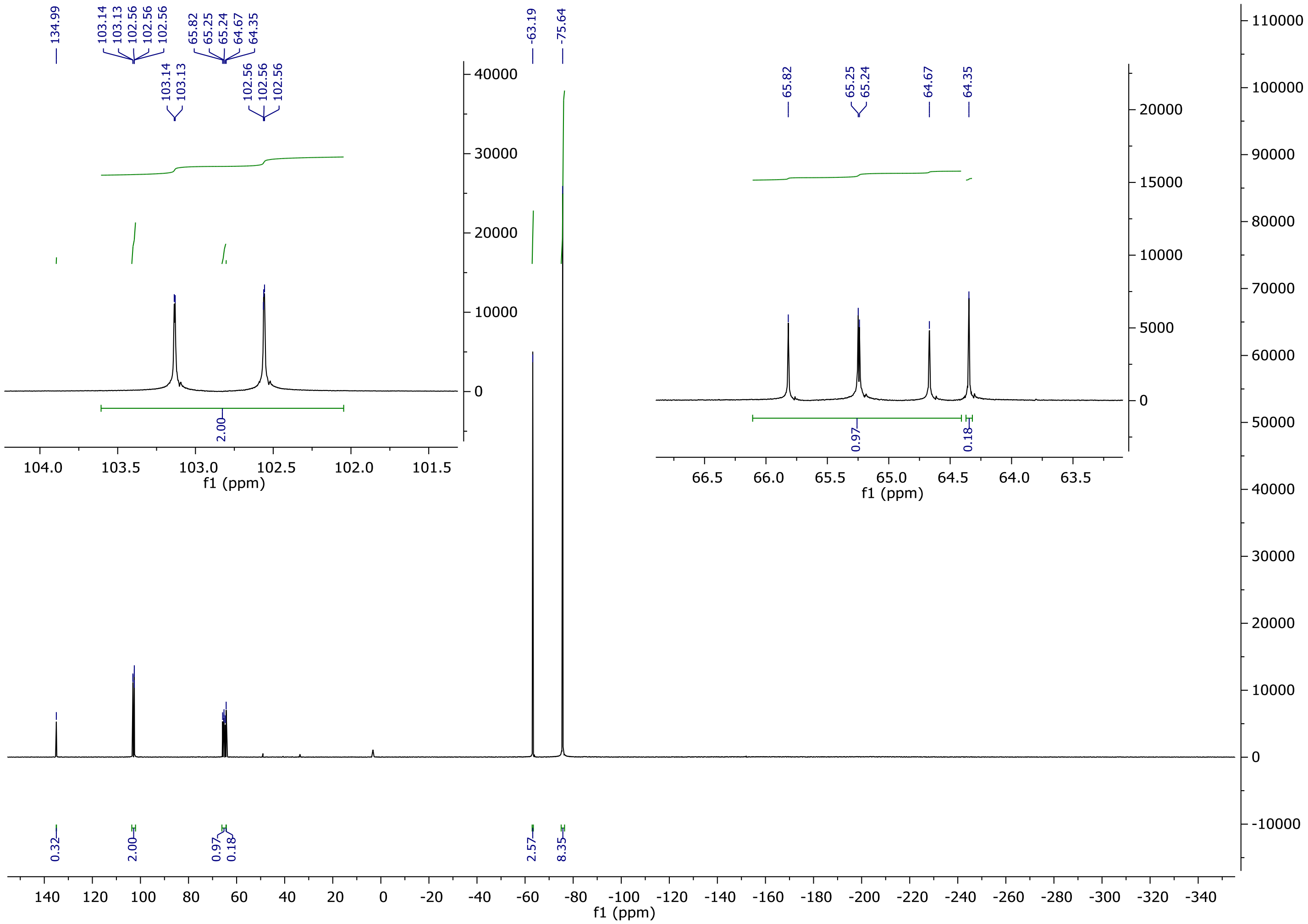

Note. Based on internal standard α,α,α-trifluorotoluene (at –63.19 ppm, 9.8 mg, 0.067 mmol), ^{19}F NMR yield: 78%. In combination with the yield for **3h**, the three-step total yield: 48%. Singlet at 135.0 ppm is the signal of 4-NO_2-$PhSF_4Cl$; Singlet at 64.4 ppm is the signal of NO_2PhSO_2F (**5f**); Singlet at –75.64 ppm is the signal of excess TFA.

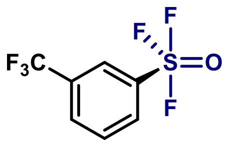

4i

3-(trifluoromethyl)phenylsulfur(VI) trifluoridoxide **4i** was prepared according to the general method in section 7.

^{19}F{^{1}H} NMR (282 MHz, CH_3CN/CD_3CN) δ 101.6 (d, *J* = 161.2 Hz, 2F), 65.8 (t, *J* = 161.2 Hz, 1F) (due to the (pseudo) trigonal-bipyramidal coordination, ^{19}F NMR spectrum shows AB_2 pattern, which will be discussed in detail in the section 9), –63.5 (s, 3F).

^{19}F NMR spectra (282 MHz)

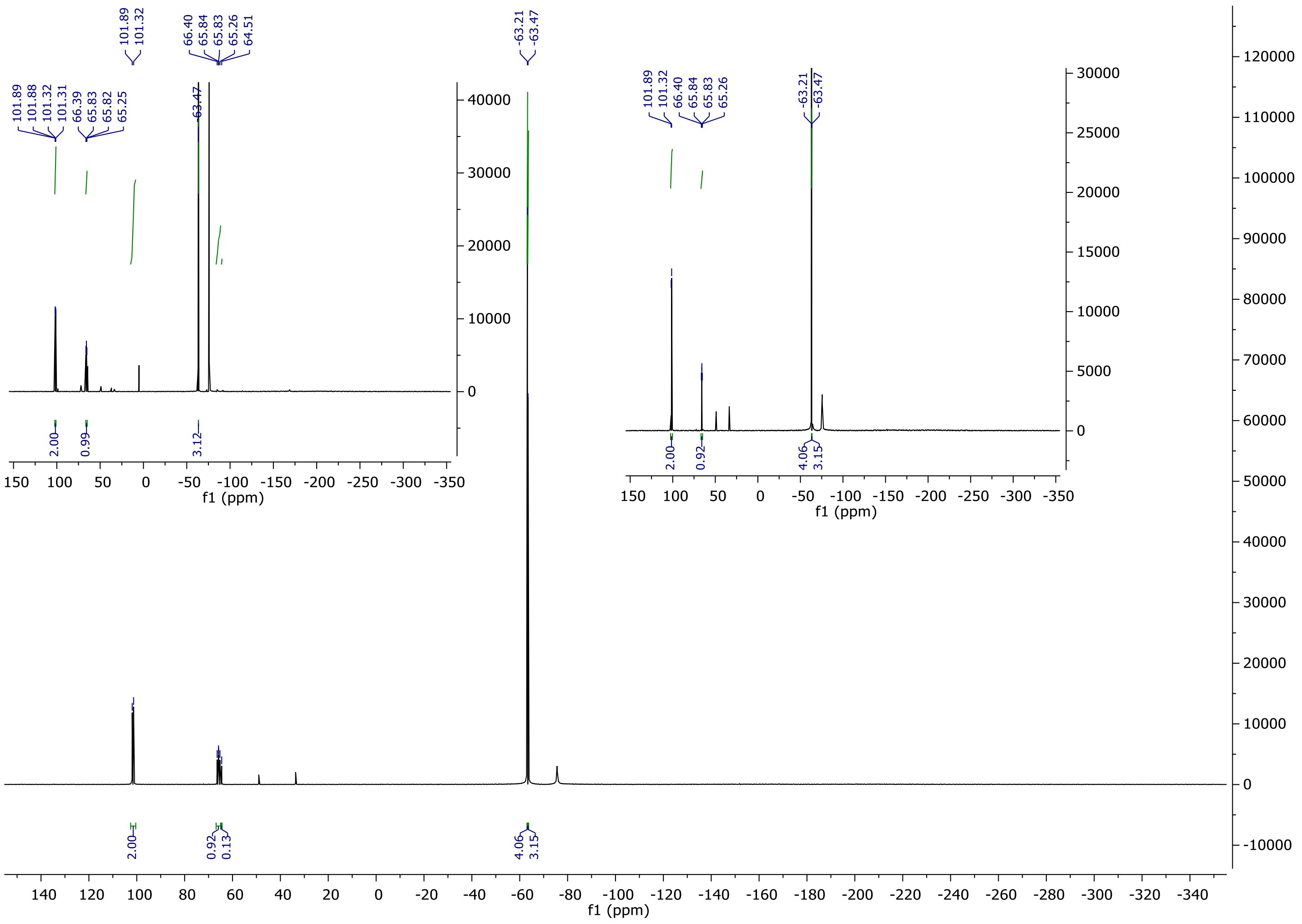

Note. Based on internal standard α,α,α-trifluorotoluene (at –63.21 ppm, 16.0 mg, 0.110 mmol), ^{19}F NMR yield: 81%. In combination with the yield for **3i**, the three-step total yield: 61%. Singlet at 64.5 ppm is the signal of 3-CF_3PhSO_2F (**5g**).

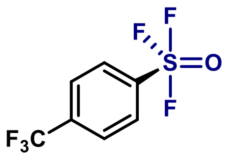

4j

4-(trifluoromethyl)phenylsulfur(VI) trifluoridoxide **4j** was prepared according to the general method in section 7.

^{19}F{^{1}H} NMR (282 MHz, CH_3CN/CD_3CN) δ 102.1 (d, *J* = 161.0 Hz, 2F), 64.9 (t, *J* = 161.2 Hz, 1F) (due to the (pseudo) trigonal-bipyramidal coordination, ^{19}F NMR spectrum shows AB_2 pattern, which will be discussed in detail in the section 9), –64.0 (s, 3F).

^{19}F NMR spectra (282 MHz)

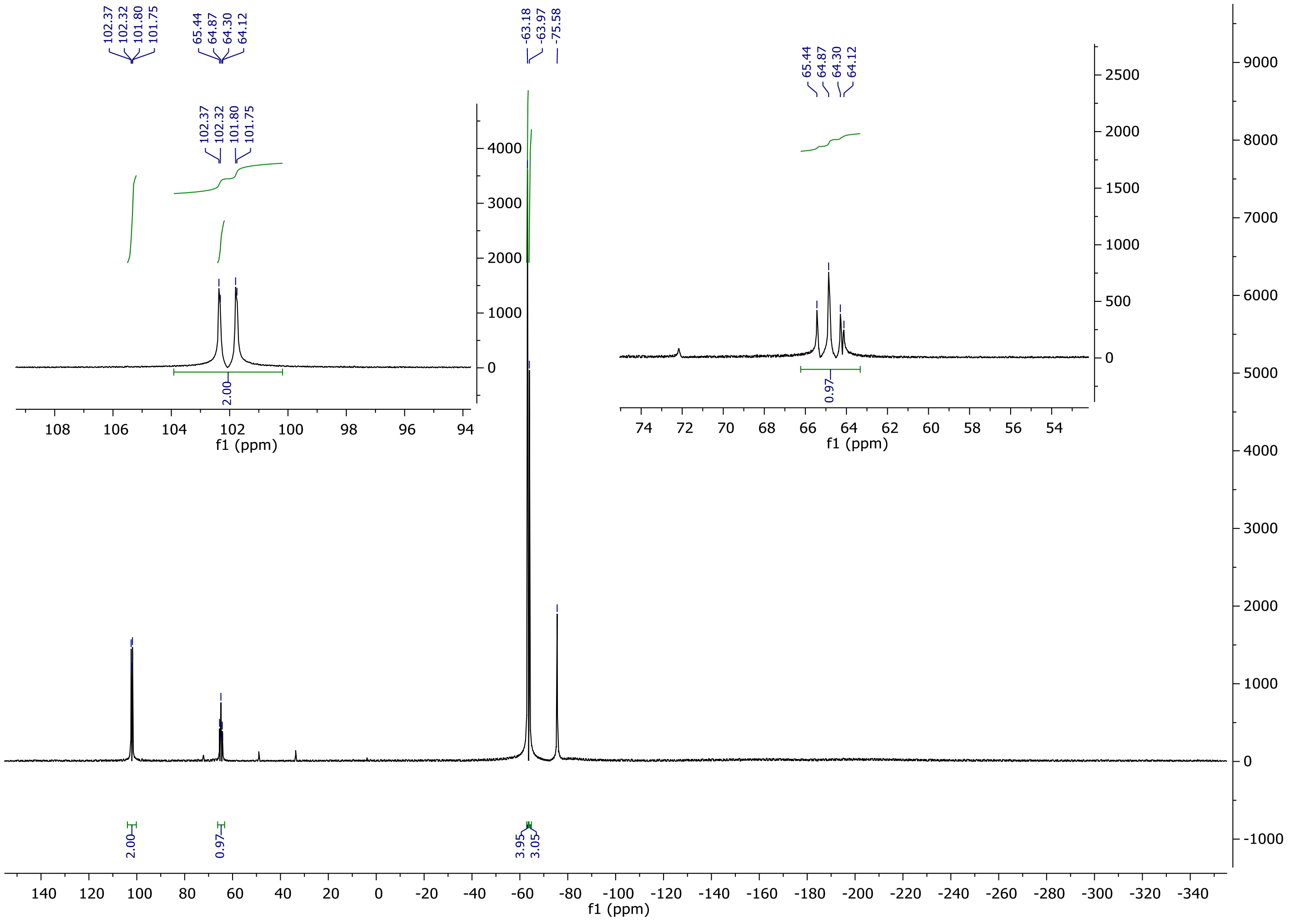

Note. Based on internal standard α,α,α-trifluorotoluene (at –63.18 ppm, 15.4 mg, 0.105 mmol), ^{19}F NMR yield: 80%. In combination with the yield for **3j**, the three-step total yield: 61%. Singlet at 64.1 ppm is the signal of 4-CF_3-$PhSO_2F$ (**5h**); Singlet at –75.58 ppm is the signal of excess TFA.

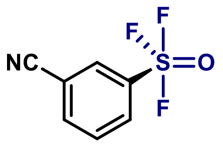

4k

3-nitrilphenylsulfur(VI) trifluoridoxide **4k** was prepared according to the general method in section 7.

^{19}F{^{1}H} NMR (282 MHz, CH_3CN/CD_3CN) δ 101.6 (d, *J* = 161.8 Hz, 2F), 66.0 (t, *J* = 161.8 Hz, 1F) (due to the (pseudo) trigonal-bipyramidal coordination, ^{19}F NMR spectrum shows AB_2 pattern, which will be discussed in detail in the section 9).

^{19}F NMR spectra (282 MHz)

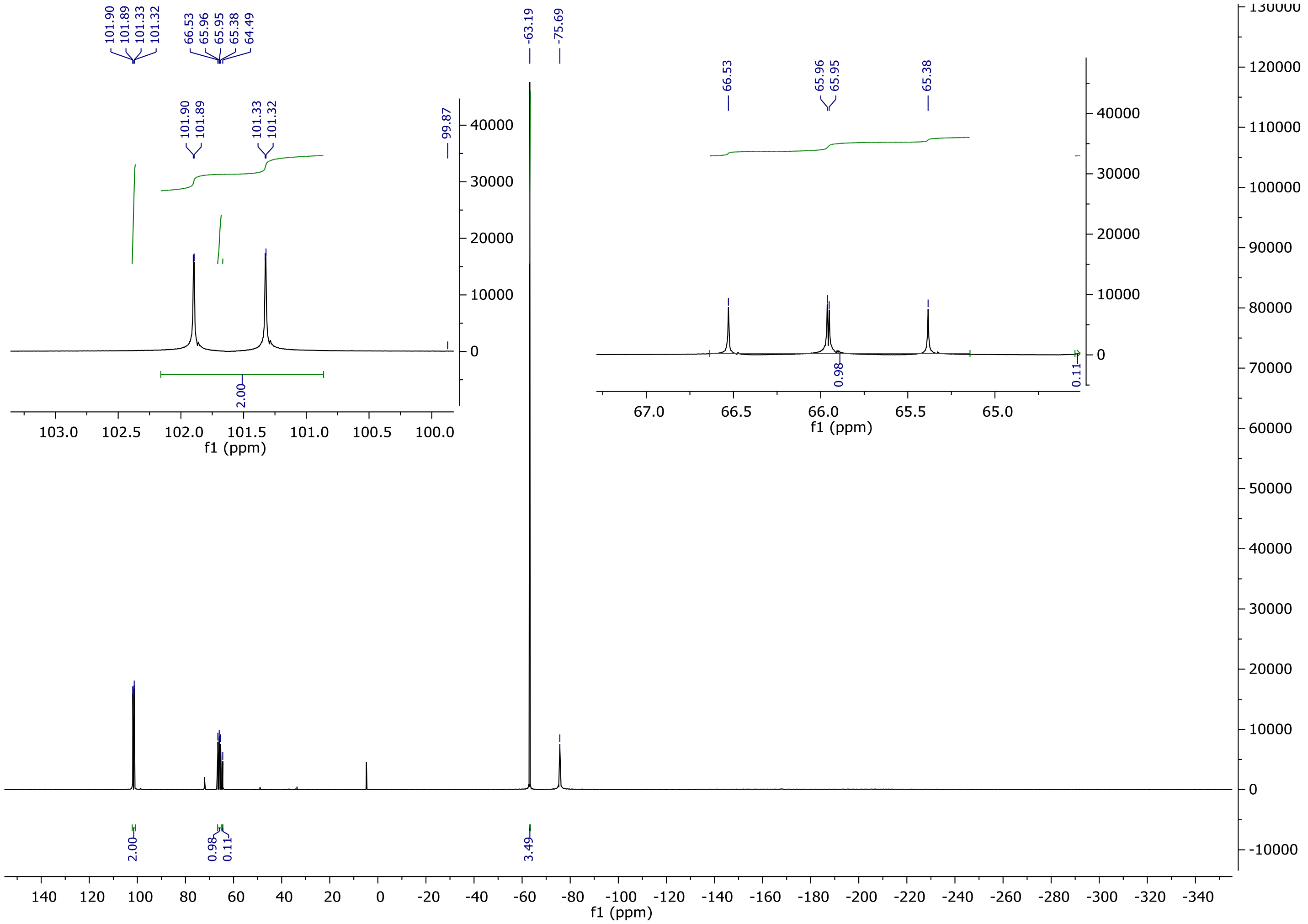

Note. Based on internal standard α,α,α-trifluorotoluene (at - 63.18 ppm, 13.6 mg, 0.093 mmol), ^{19}F NMR yield: 80%. In combination with the yield for **3k**, the three-step total yield: 67%. Singlet at 64.5 ppm is the signal of 3-CN-$PhSO_2F$; Singlet at –75.69 ppm is the signal of excess TFA.

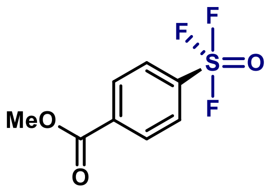

4l

Methyl 4-benzoatesulfur(VI) trifluoridoxide **4l** was prepared according to the general method in section 7.

^{19}F{^{1}H} NMR (282 MHz, CH_3CN/CD_3CN) δ 101.7 (d, *J* = 160.1 Hz, 2F), 64.5 (t, *J* = 160.1 Hz, 1F) (due to the (pseudo) trigonal-bipyramidal coordination, ^{19}F NMR spectrum shows AB_2 pattern, which will be discussed in detail in the section 9).

^{19}F NMR spectra (282 MHz)

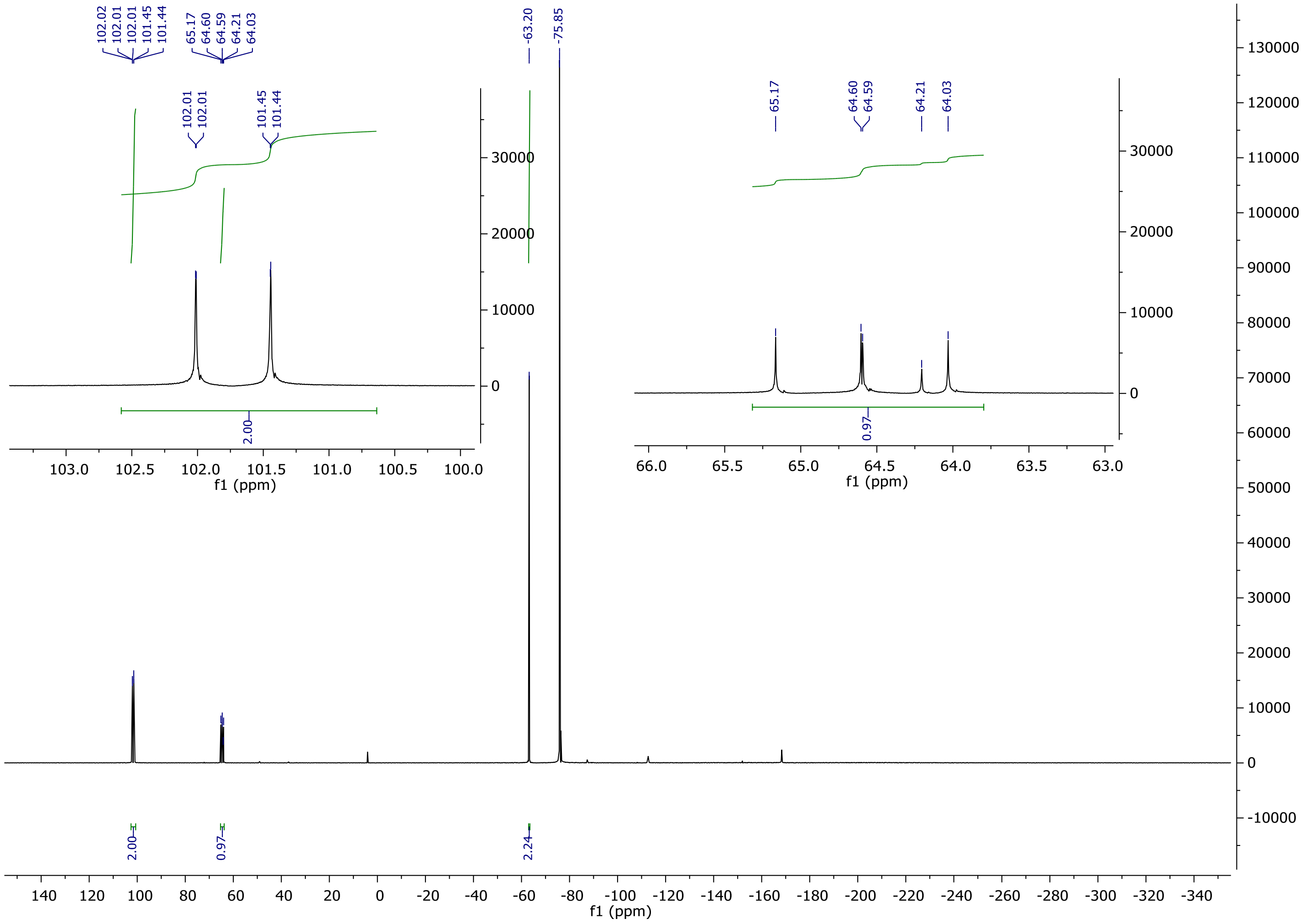

Note. Based on internal standard α,α,α-trifluorotoluene (at –63.20 ppm, 9.7 mg, 0.066 mmol), ^{19}F NMR yield: 89%. In combination with the yield for **3l**, the three-step total yield: 75%. Singlet at 64.2 ppm is the signal of 4-COOMe-$PhSO_2F$; Singlet at –75.85 ppm is the signal of excess TFA.

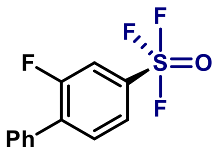

4m

(2-Fluoro[1,1'-biphenyl]-4-yl)sulfur(VI) trifluoridoxide **4m** was prepared according to the general method in section 7.

^{19}F{^{1}H} NMR (282 MHz, CH_3CN/CD_3CN) δ 100.7 (d, *J* = 160.2 Hz, 2F), 66.4 (t, *J* = 160.2 Hz, 1F) (due to the (pseudo) trigonal-bipyramidal coordination, ^{19}F NMR spectrum shows AB_2 pattern, which will be discussed in detail in the section 9), –114.0 (s, 1F).

^{19}F NMR spectra (282 MHz)

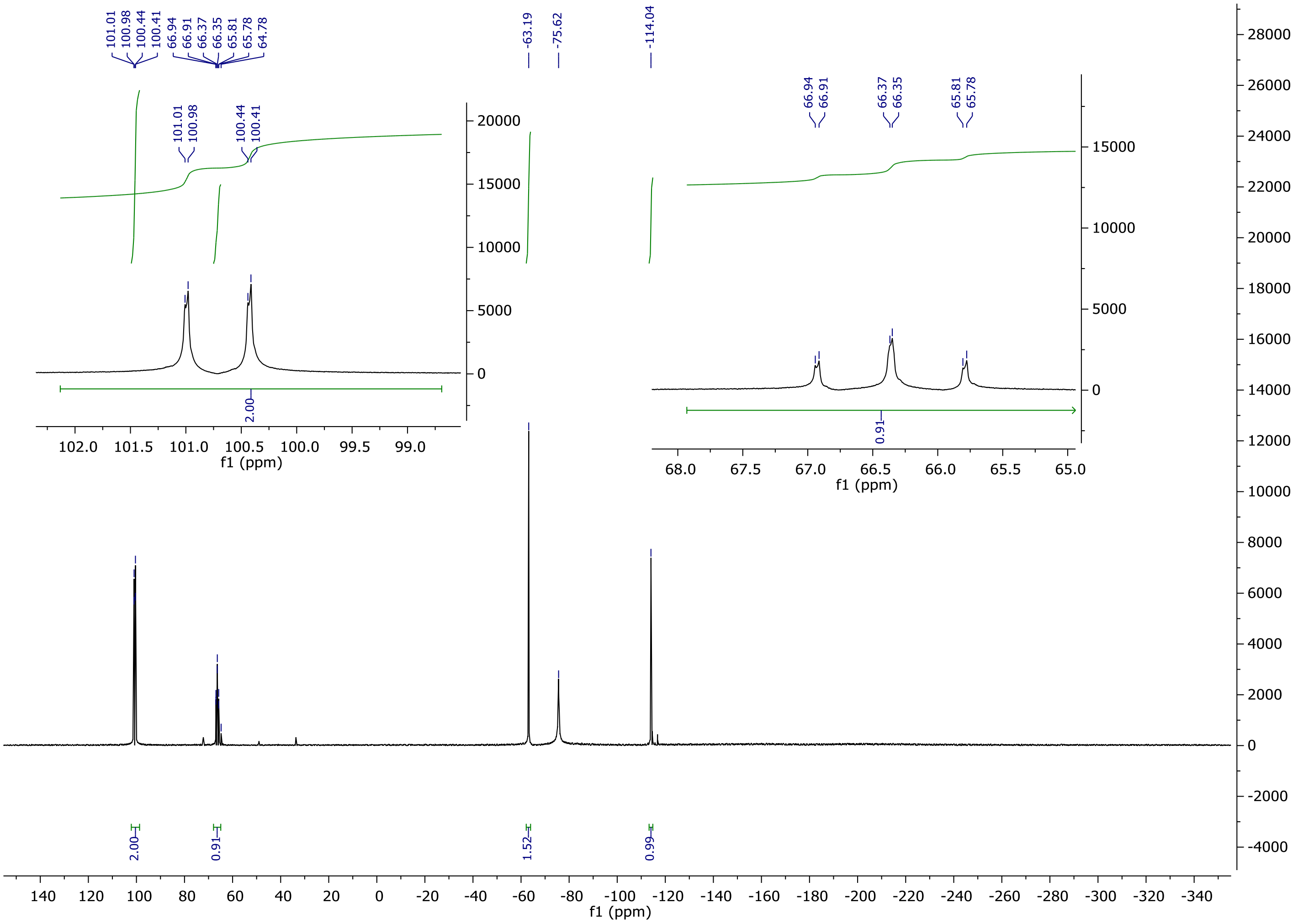

Note. Based on internal standard α,α,α-trifluorotoluene (at –63.20 ppm, 7.0 mg, 0.048 mmol), ^{19}F NMR yield: 94%. In combination with the yield for **3m**, the three-step total yield: 70%. Singlet at 64.78 ppm is the signal of (3-F)(4-Ph)-$PhSO_2F$ (**5j**); Singlet at –75.62 ppm is the signal of excess TFA.

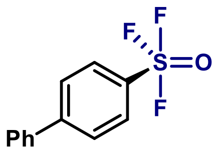

4n

([1,1'-biphenyl]-4-yl)sulfur(VI) trifluoridoxide **4n** was prepared according to the general method in section 7.

^{19}F{^{1}H} NMR (282 MHz, CH_3CN/CD_3CN) δ 99.8 (d, *J* = 157.8 Hz, 2F), 65.8 (t, *J* = 157.8 Hz, 1F) (due to the (pseudo) trigonal-bipyramidal coordination, ^{19}F NMR spectrum shows AB_2 pattern, which will be discussed in detail in the section 9).

^{19}F NMR spectra (282 MHz)

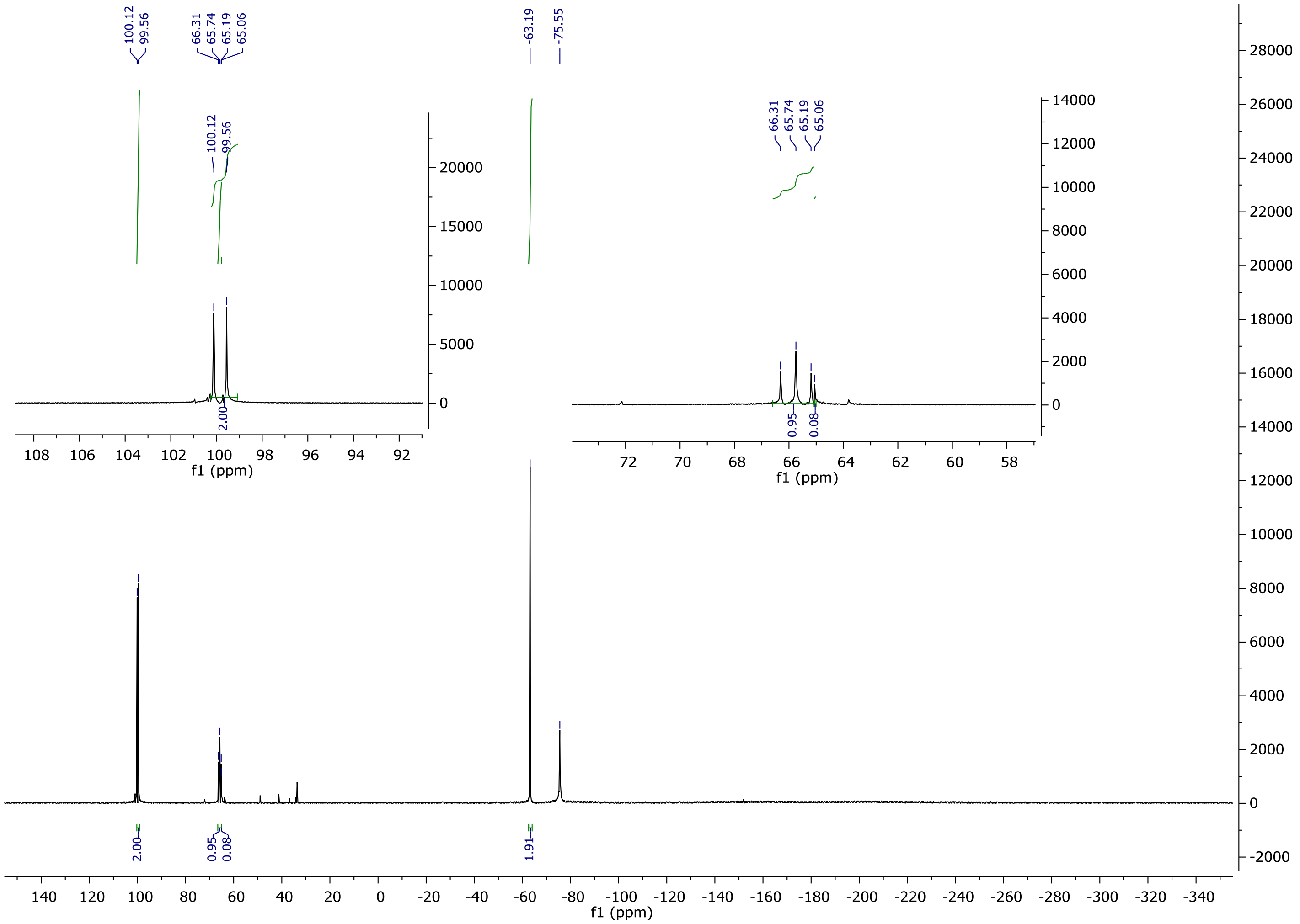

Note. Based on internal standard α,α,α-trifluorotoluene (at –63.19 ppm, 8.0 mg, 0.054 mmol), ^{19}F NMR yield: 85%. In combination with the yield for **3n**, the three-step total yield: 65%. Singlet at 65.06 ppm is the signal of (4-Ph)-$PhSO_2F$ (**5i**); Singlet at –75.55 ppm is the signal of excess TFA.

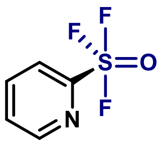

4o

2-Pyridinylsulfur(VI) trifluoridoxide **4o** was prepared according to the general method in section 7.

^{19}F{^{1}H} NMR (282 MHz, CH_3CN/CD_3CN) δ 92.8 (d, *J* = 161.4 Hz, 2F), 57.2 (t, *J* = 160.1 Hz, 1F) (due to the (pseudo) trigonal-bipyramidal coordination, ^{19}F NMR spectrum shows AB_2 pattern, which will be discussed in detail in the section 9).

^{19}F NMR spectra (282 MHz)

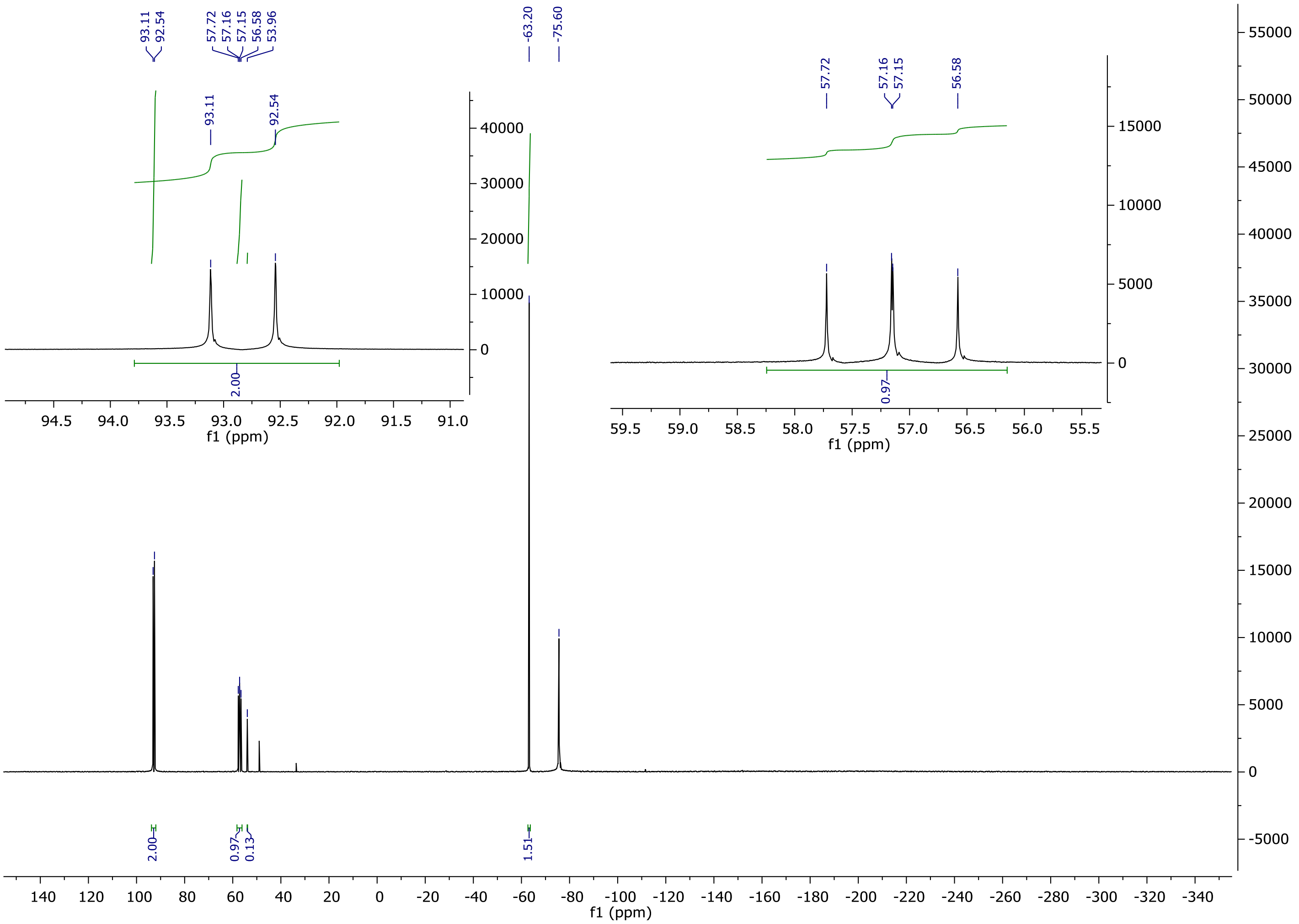

Note. Based on internal standard α,α,α-trifluorotoluene (at –63.20 ppm, 5.2 mg, 0.036 mmol), ^{19}F NMR yield: 71%. In combination with the yield for **3o**, the three-step total yield: 52%. Singlet at 54.0 ppm is the signal of 2-PySO$_2$F (**5k**); Singlet at –75.60 ppm is the signal of excess TFA.

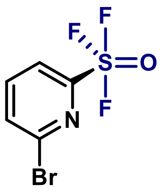

4p

6-Bromo-2-pyridinylsulfur(VI) trifluoridoxide **4p** was prepared according to the general method in section 7.

^{19}F{^{1}H} NMR (282 MHz, CH_3CN/CD_3CN) δ 93.8 (d, *J* = 164.9 Hz, 2F), 59.2 (t, *J* = 164.9 Hz, 1F) (due to the (pseudo) trigonal-bipyramidal coordination, ^{19}F NMR spectrum shows AB_2 pattern, which will be discussed in detail in the section 9).

^{19}F NMR spectra (282 MHz)

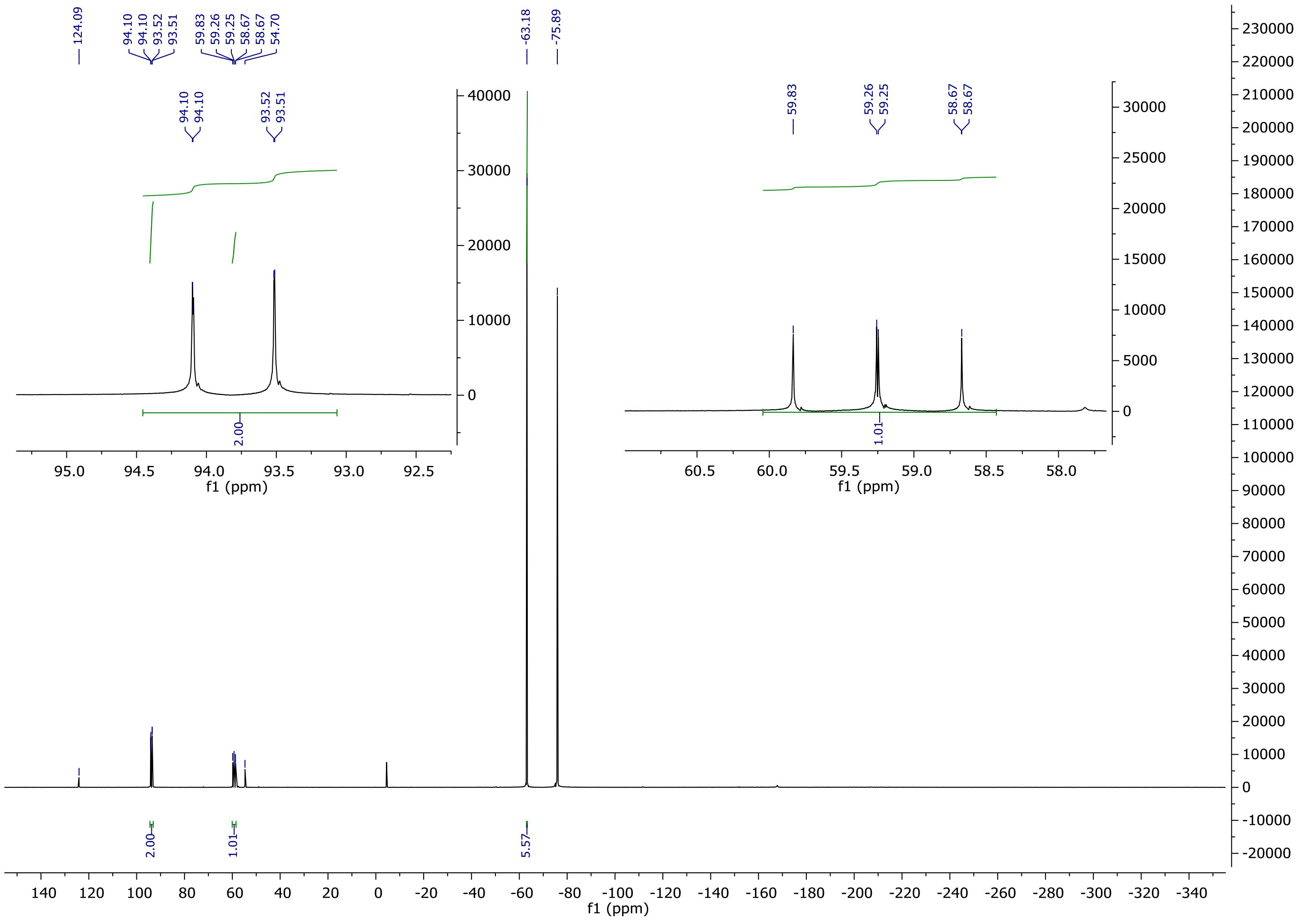

Note. Based on internal standard α,α,α-trifluorotoluene (at –63.18 ppm, 14.6 mg, 0.100 mmol), ^{19}F NMR yield: 54%. In combination with the yield for **3p**, the three-step total yield: 43%. Singlet at 124.1 ppm is the signal of 6-Br-2-$PySF_4Cl$ (**6i**); Singlet at 54.7 ppm is the signal of 6-Br-2-$PySO_2F$; Singlet at –75.89 ppm is the signal of excess TFA.

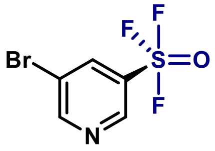

4q

5-Bromo-3-pyridinylsulfur(VI) trifluoridoxide **4q** was prepared according to the general method in section 7.

^{19}F{^{1}H} NMR (282 MHz, CH_3CN/CD_3CN) δ 102.3 (d, *J* = 164.8 Hz, 2F), 69.6 (t, *J* = 164.8 Hz, 1F) (due to the (pseudo) trigonal-bipyramidal coordination, ^{19}F NMR spectrum shows AB_2 pattern, which will be discussed in detail in the section 9).

^{19}F NMR spectra (282 MHz)

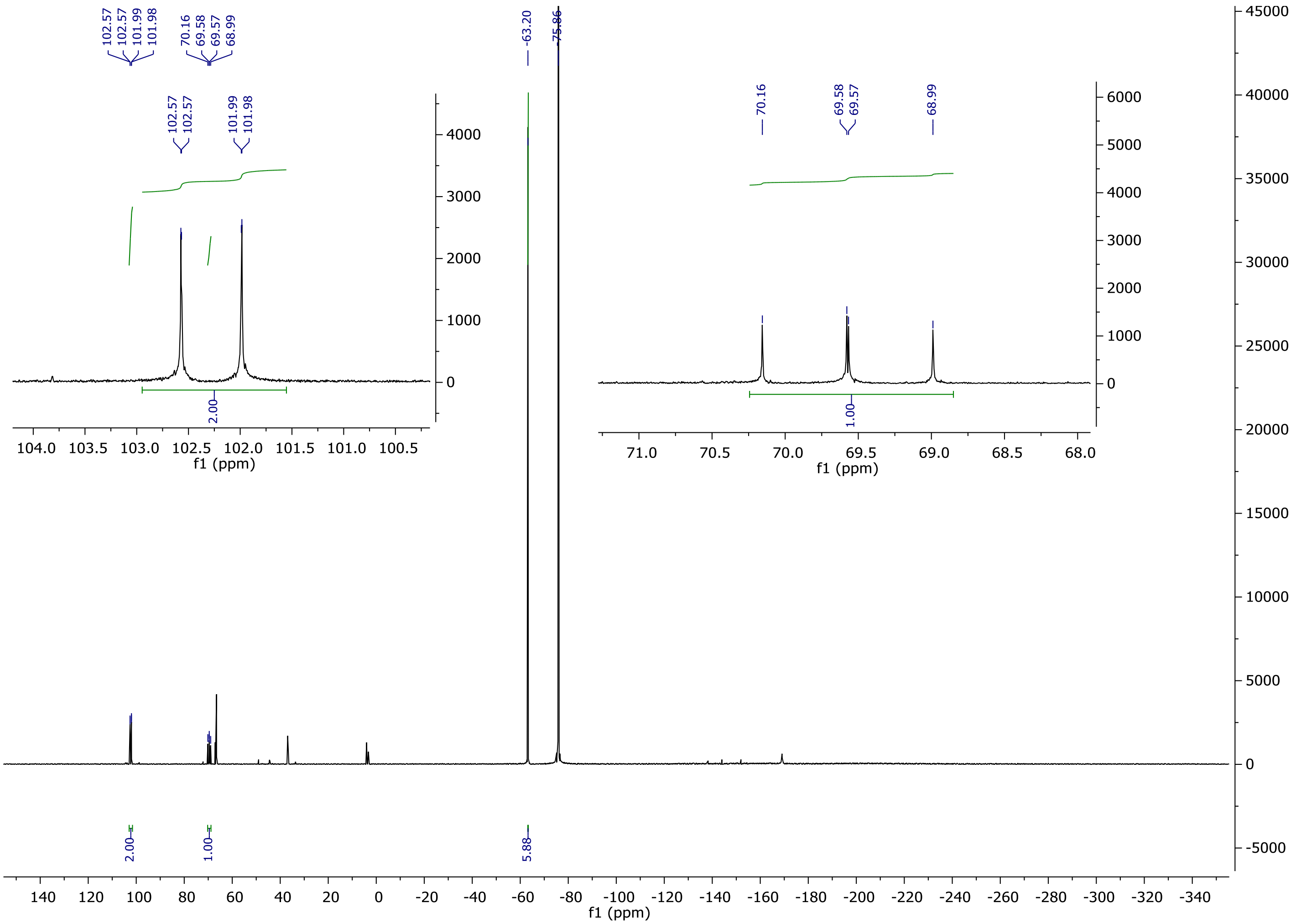

Note. Based on internal standard α,α,α-trifluorotoluene (at –63.20 ppm, 13.7 mg, 0.094 mmol), ^{19}F NMR yield: 48%. In combination with the yield for **3q**, the three-step total yield: 34%. Other signals are complicated; Singlet at –75.86 ppm is the signal of excess TFA.

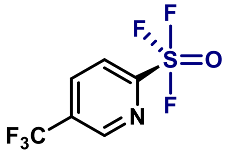

4r

4-(Trifluoromethyl)-2-pyridinylsulfur(VI) trifluoridoxide **4r** was prepared according to the general method in section 7.

^{19}F{^{1}H} NMR (282 MHz, CH_3CN/CD_3CN) δ 93.7 (d, *J* = 164.2 Hz, 2F), 58.1 (t, *J* = 164.2 Hz, 1F) (due to the (pseudo) trigonal-bipyramidal coordination, ^{19}F NMR spectrum shows AB_2 pattern, which will be discussed in detail in the section 9), –63.4 (s, 3F).

^{19}F NMR spectra (282 MHz)

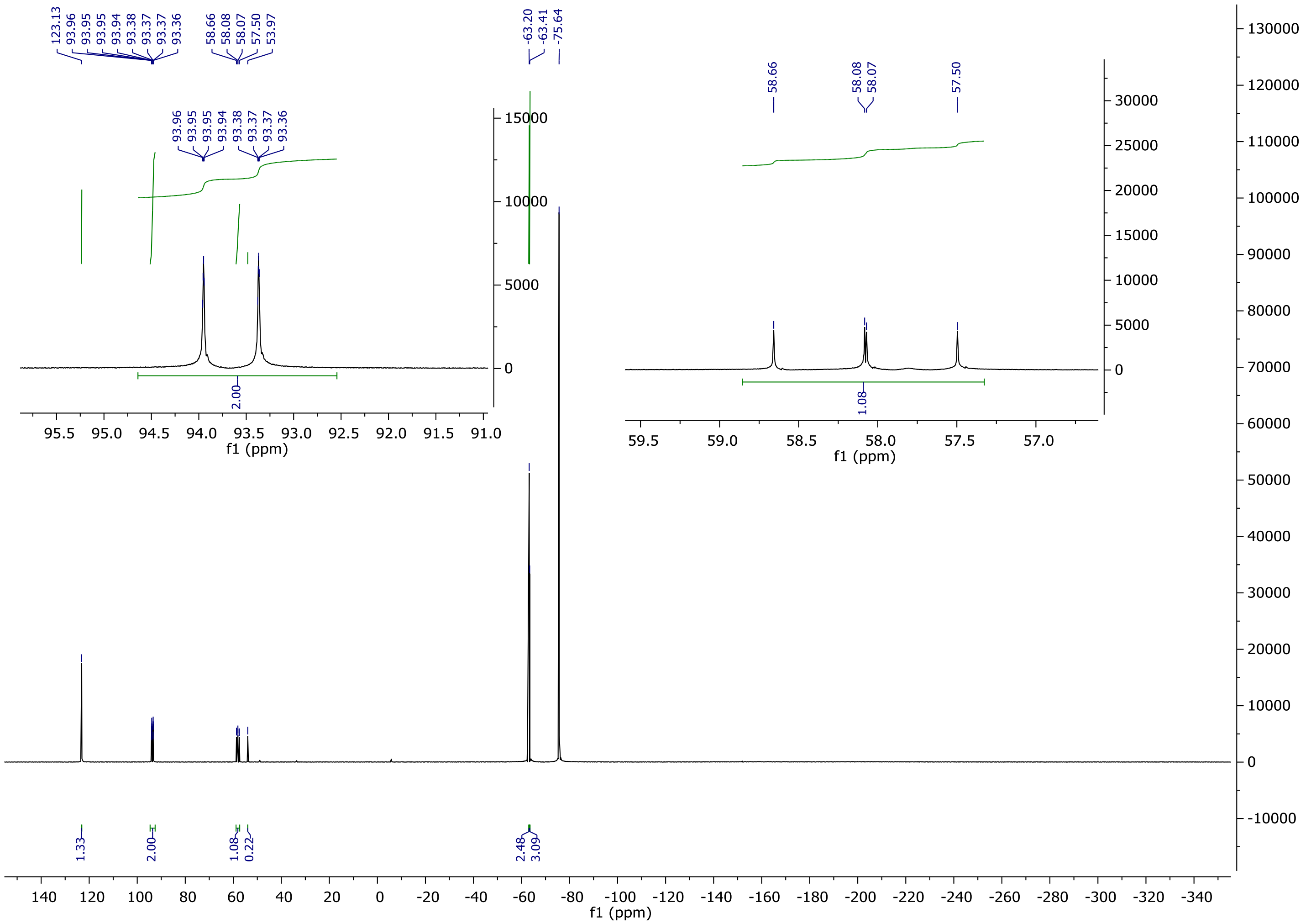

Note. Based on internal standard α,α,α-trifluorotoluene (at –63.20 ppm, 7.4 mg, 0.050 mmol), ^{19}F NMR yield: 61%. In combination with the yield for **3r**, the three-step total yield: 42%. Singlet at 123.1 ppm is the signal of 4-CF_3-2-$PySF_4Cl$; Singlet at 54.0 ppm is the signal of 4-CF_3-2-$PySO_2F$; Singlet at –75.64 ppm is the signal of excess TFA.

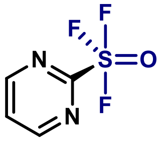

4s

Note. The sulfur(II) precursor was prepared from the reaction of N-chlorophthalimide with 2-pyrimidinethiol according to the literature [15] because the attempt to synthesize the 2-pyrimidinyl zinc halide is failed.

The preparation of 2-pyrimidinylsulfur(VI) trifluoridoxide **4s** is based on the procedure described below:

In a glovebox under Argon, the 2-pyrimidinyl-S-Phth precursor (**3s**, 0.1 mmol, 1 equiv.) and anhydrous $ZnCl_2$ (0.2 mmol, 2 equiv.) were added to an oven-dried 12 mL round-bottom reaction vial equipped with a stir bar. Subsequently, 0.5 mL dry and degassed MeCN was added to dissolve the mixture. The suspension was stirred for 10 min followed by addition of trichloroisocyanuric acid (TCICA, 210 mg, 0.9 mmol, 9 equiv.), rigorously dried KF (93 mg, 16 mmol, 16 equiv.), 0.5 mL MeCN and TFA solution (1M in MeCN, 0.2 mL). Then the vial was sealed with a septum-pad cap and the reaction was stirred at the room temperature in the glovebox for 24 h. Upon the completion of reaction, the atmosphere in the vial was vented carefully and the internal standard α, α, α-trifluorotoluene was added into the mixture. After 10 min stirring, an aliquot of the resulting solution was filtered under argon. The NMR sample was prepared with 0.4 mL of the filtered aliquot and 0.1 mL CD_3CN for ^{19}F NMR yield determination.

$^{19}F\{^1H\}$ NMR (282 MHz, CH_3CN/CD_3CN) δ 87.8 (d, *J* = 159.1 Hz, 2F), 51.5 (t, *J* = 159.1 Hz, 1F) (due to the (pseudo) trigonal-bipyramidal coordination, ^{19}F NMR spectrum shows AB_2 pattern, which will be discussed in detail in the section 9).

Note. With the general method, the desired product **4s** was obtained only in the yield of 3 – 6% ($ArSF_4Cl$ is the major product). With the above-mentioned procedure, based on internal standard α,α,α-trifluorotoluene (at –63.20 ppm, 6.9 mg, 0.047 mmol), ^{19}F NMR yield: 15%. In addition of **4s**, the signal of 2-pyrimidinyl-SF_3 [51.0 ppm (d, J = 90.2 Hz, 2F) and –51.9 ppm (t, J = 90.2 Hz, 1F)] was found in the ^{19}F NMR spectrum; Other signals are complicated; Singlet at –75.95 ppm is the signal of excess TFA.

^{19}F NMR spectra (282 MHz)

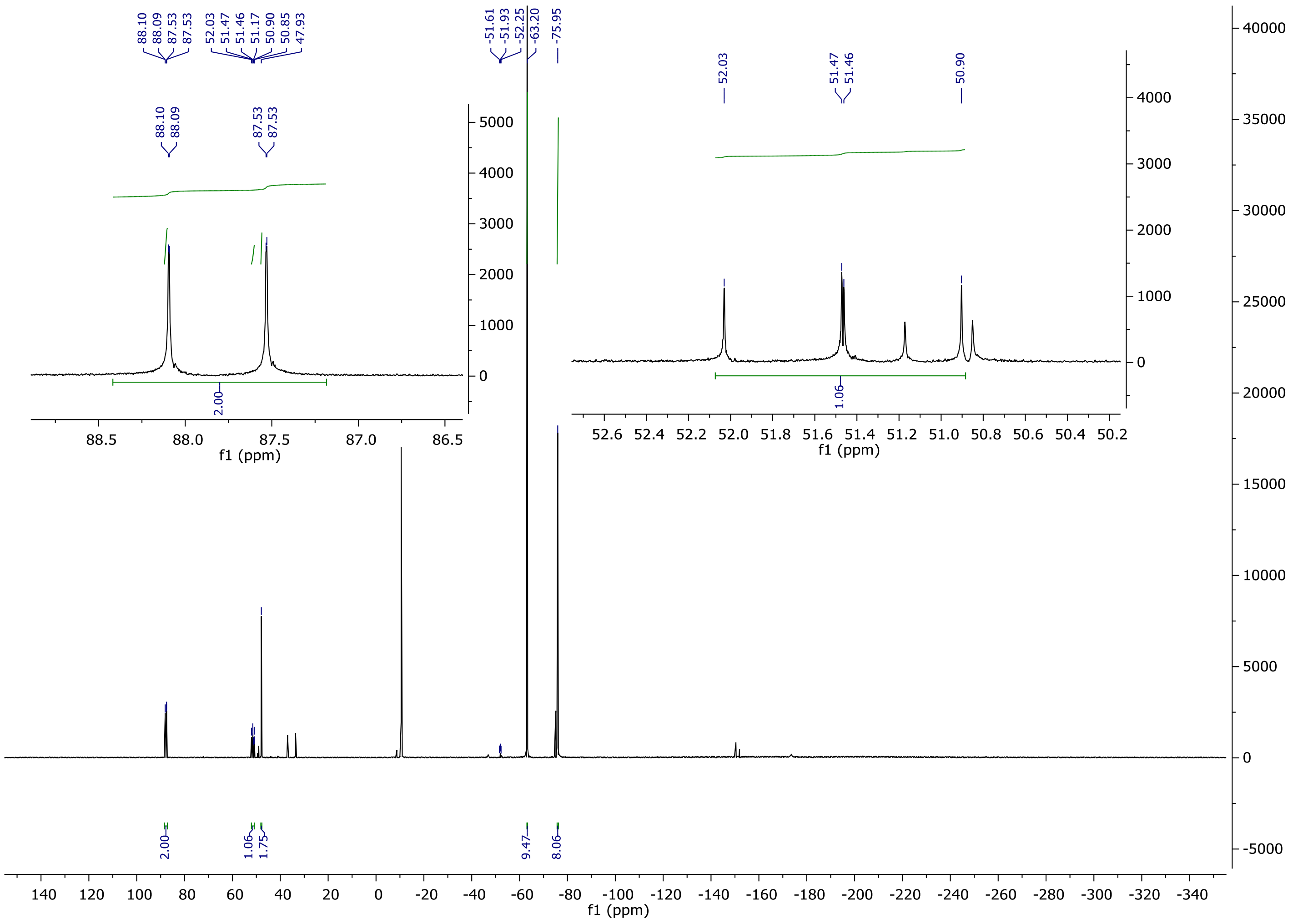

9. Analysis of the structure of $ArSOF_3$

Some molecules which contain three spin systems while two of the nuclei are magnetically equivalent could show AX_2 or AB_2 patterns in the NMR spectra. For AB_2 patterns, the spectrum depends only on the ratio of $\Delta\nu/J$. If the $\Delta\nu/J$ is large enough, the system is usually AX_2, which consists of an A triplet and a B doublet. However, as $\Delta\nu/J$ becomes smaller, the double-intensity middle line of A and both B lines split into two lines. [16] According to our results, all the ^{19}F NMR spectra of $ArSOF_3$ showed typical AB_2 patterns. [17] Furthermore, we measured one sample (**4d**) at different NMR machines. As a result, we found that at 43 MHz or 300 MHz NMR machines, the splitting of AB_2 patterns is pretty obvious (Figure S1 & S2). However, at 500 MHz or 600 MHz NMR machines, the splitting became inconspicuous (Figure S3 & S4). This may be due to the general fact that the linewidth on the 600 and 500 MHz NMR is not as good as on the 43 or 300 MHz. Another possible explanation is the $\Delta\nu$ is much larger at 600 and 500 MHz NMR than the one at 43 or 300 MHz machines.

The peaks of ~4% intensity next to the major peak is also not an impurity. It is the ^{19}F-^{34}S isotope [18], which is 4.2% natural abundance. The major peak arise from the ^{19}F-^{32}S-Species with a natural abundance of 95%.

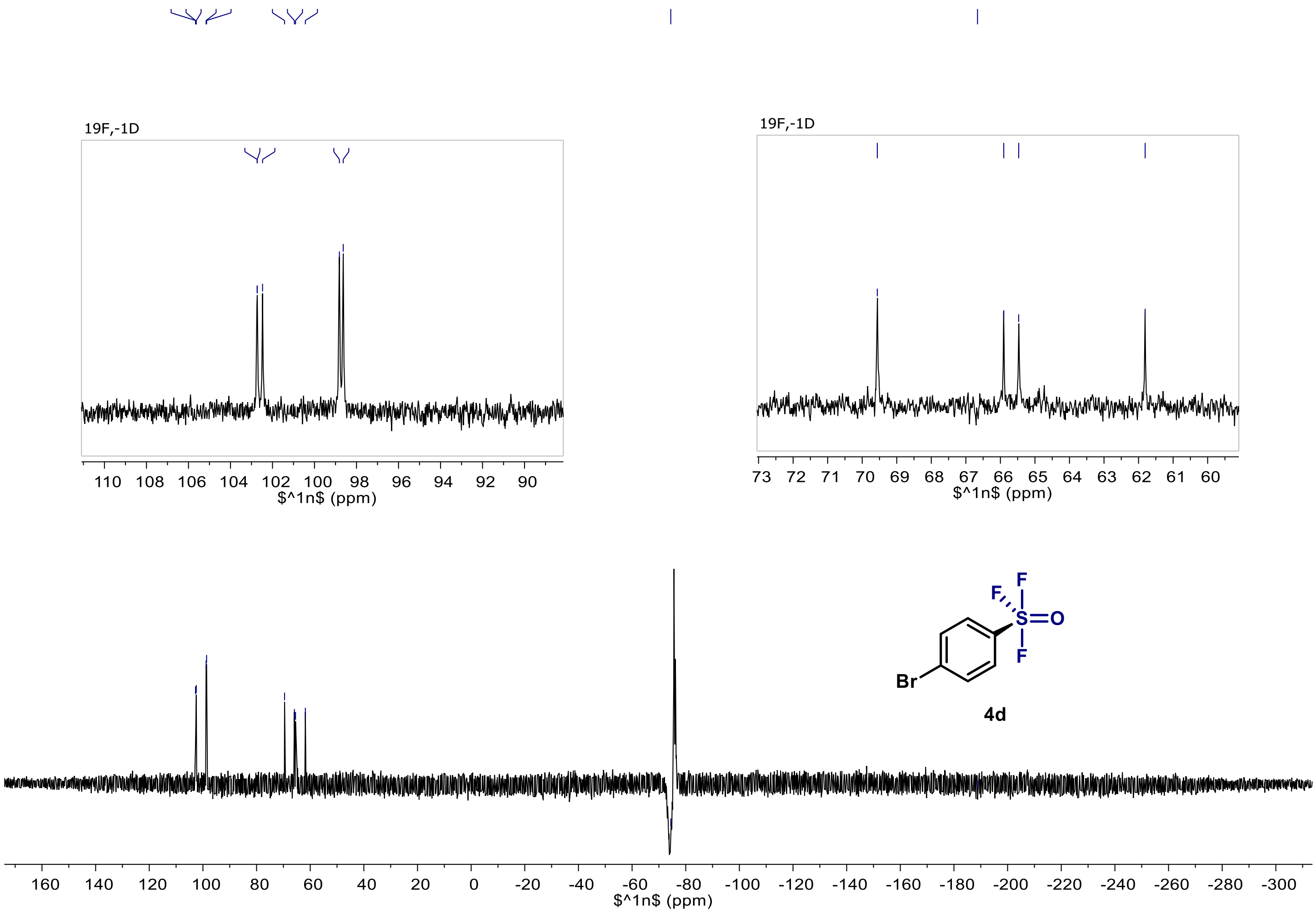

Figure S1. The ^{19}F NMR spectrum of **4d** at 43 MHz NMR machine.

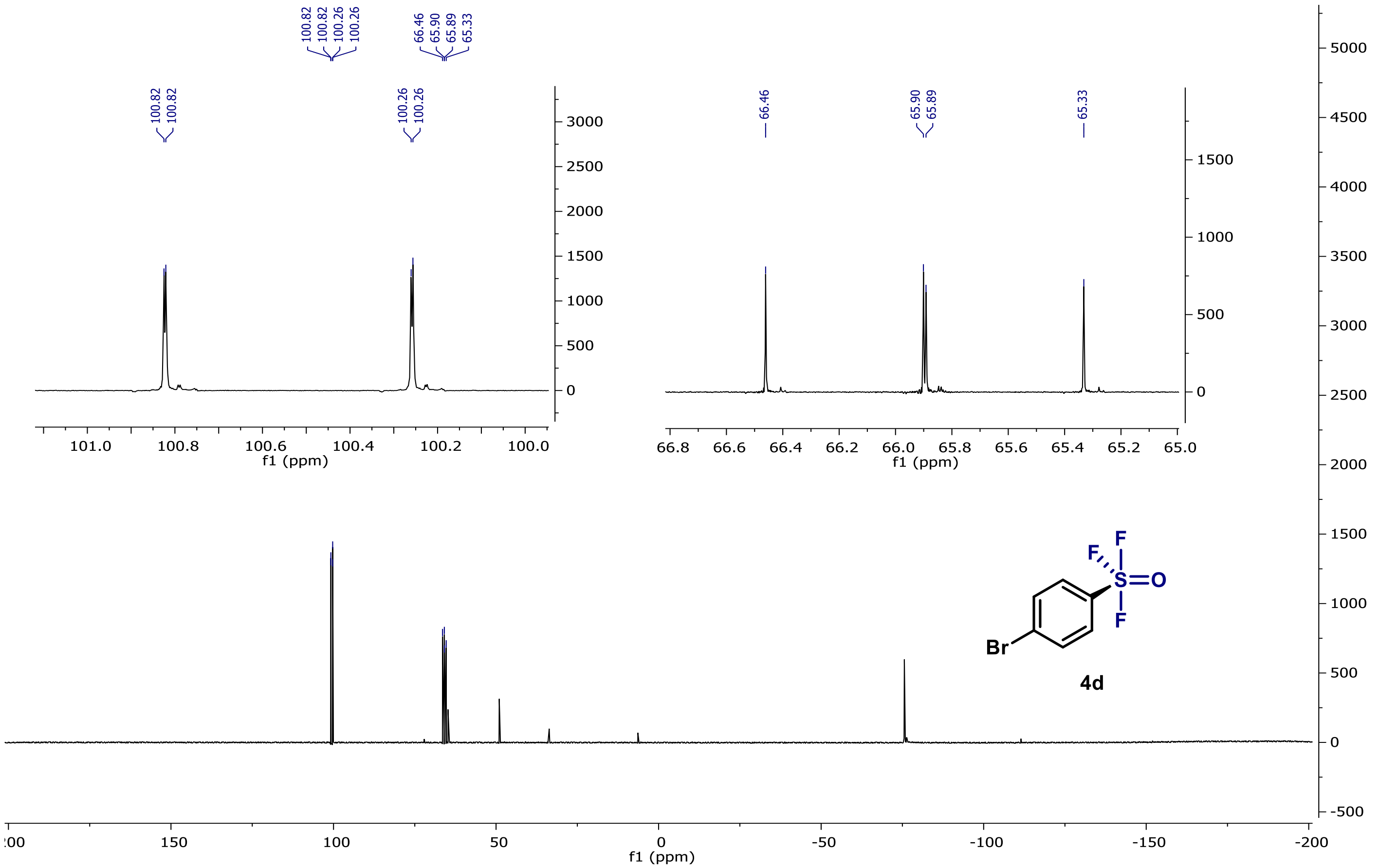

Figure S2. The ^{19}F NMR spectrum of **4d** at 300 MHz NMR machine.

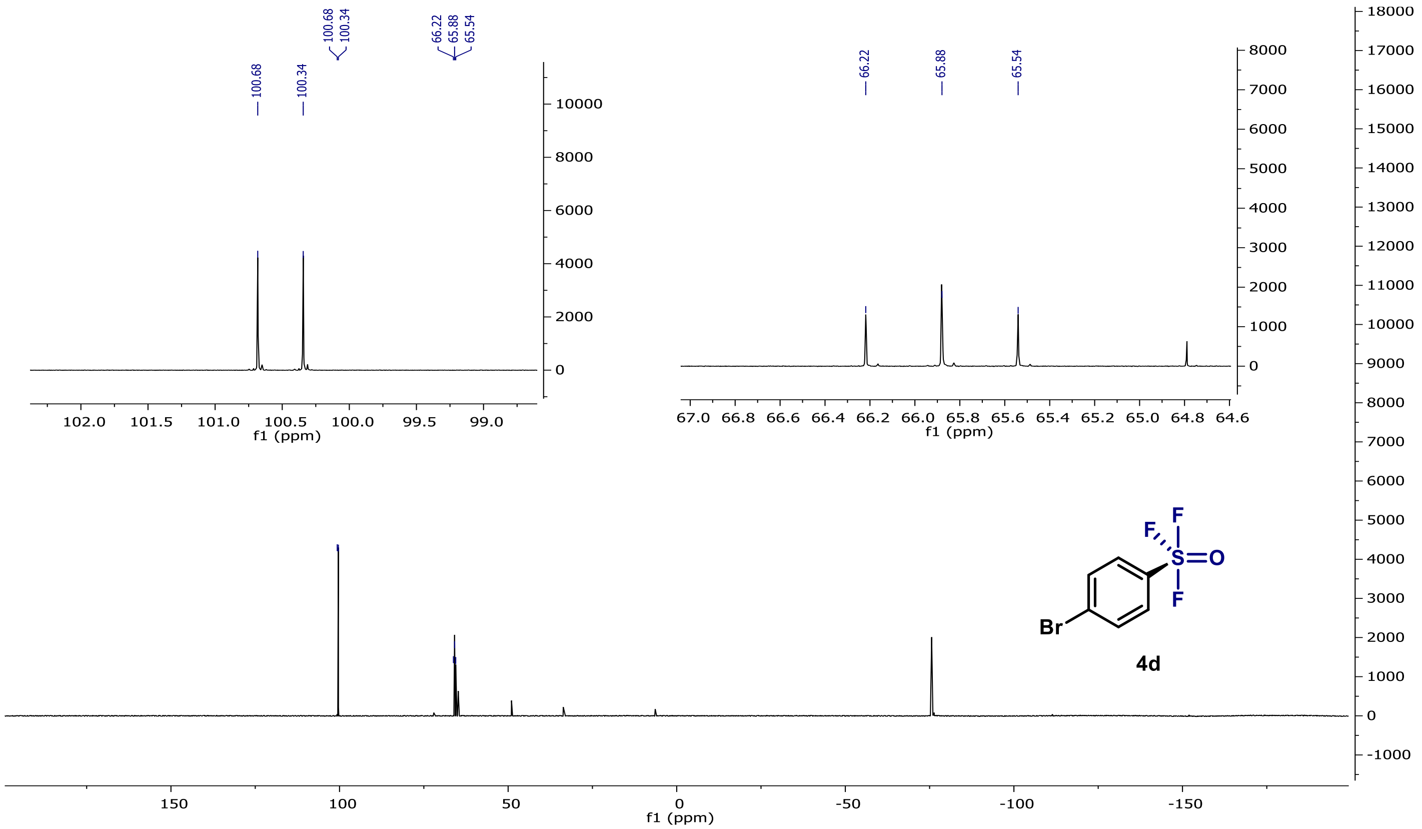

Figure S3. The ^{19}F NMR spectrum of **4d** at 500 MHz NMR machine.

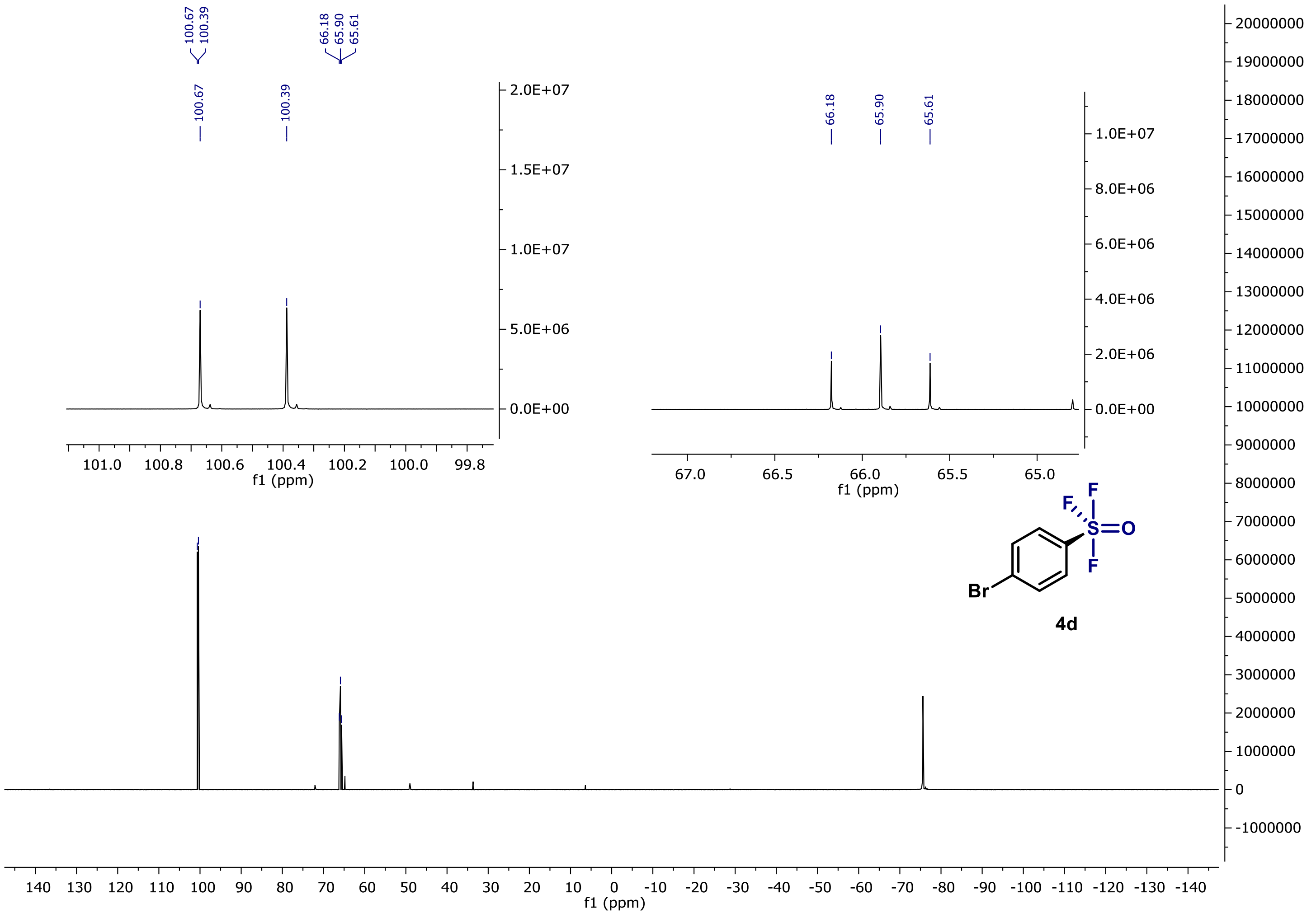

Figure S4. The ^{19}F NMR spectrum of **4d** at 600 MHz NMR machine.

Moreover, spin simulations have been made at 43, 300 and 600 MHz machines. The measured results were found to match the simulated results perfectly (Figure S5). And the VT ^{19}F NMR (-30 °C to 60 °C) showed the temperature did not influence the splitting or the stability of $ArSOF_3$ compound in solution (Figure S6).

Simulation parameters

#	Label	Value	N	Spin	LineWidth (Hz)
1	A	65.90 ppm	1	1/2	0.6
	JA-B	159.50 Hz			
2	B	100.53 ppm	2	1/2	0.6
	JB-A	159.50 Hz			

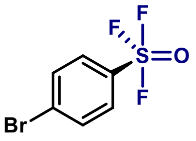

4d

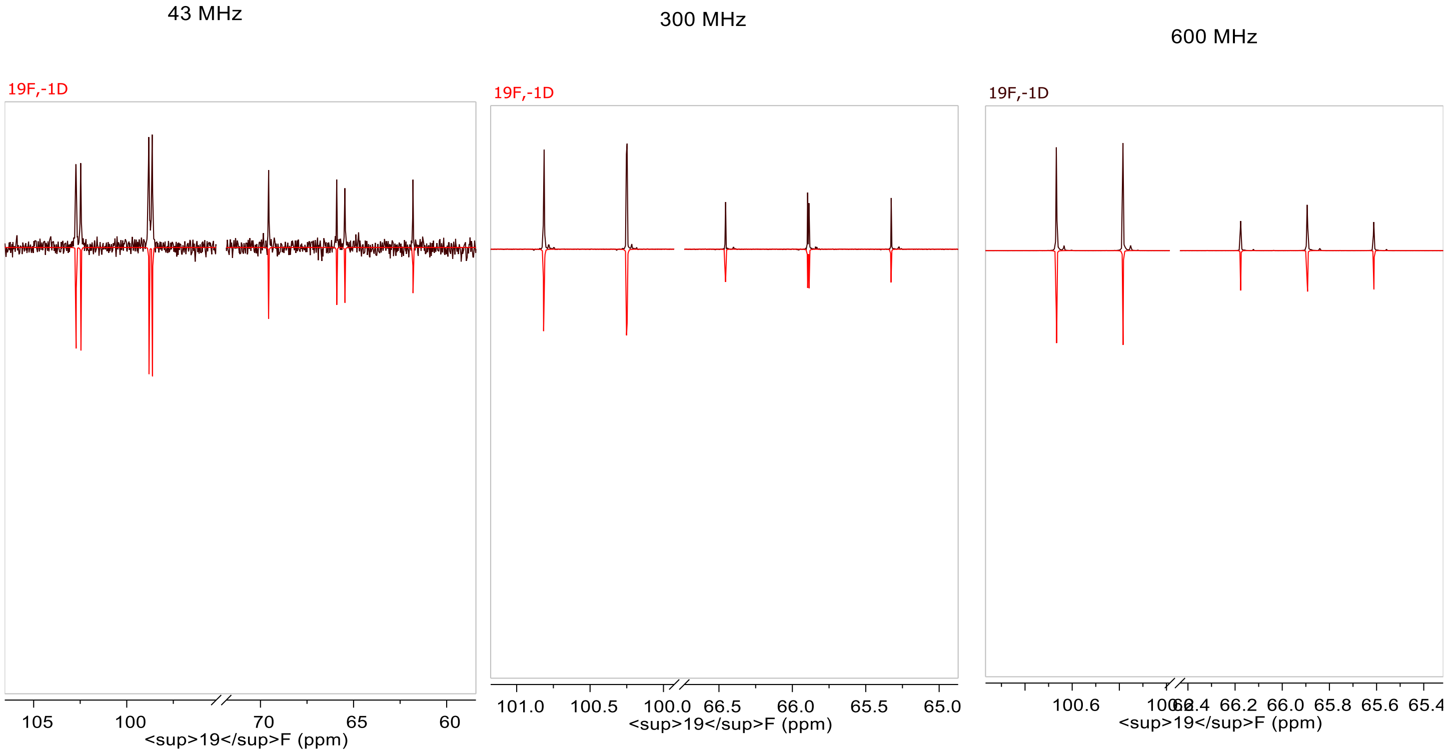

Figure S5. The spin simulations of **4d** at 43, 300 and 600 MHz NMR machines (Red line is the simulation result, black is the measured one).

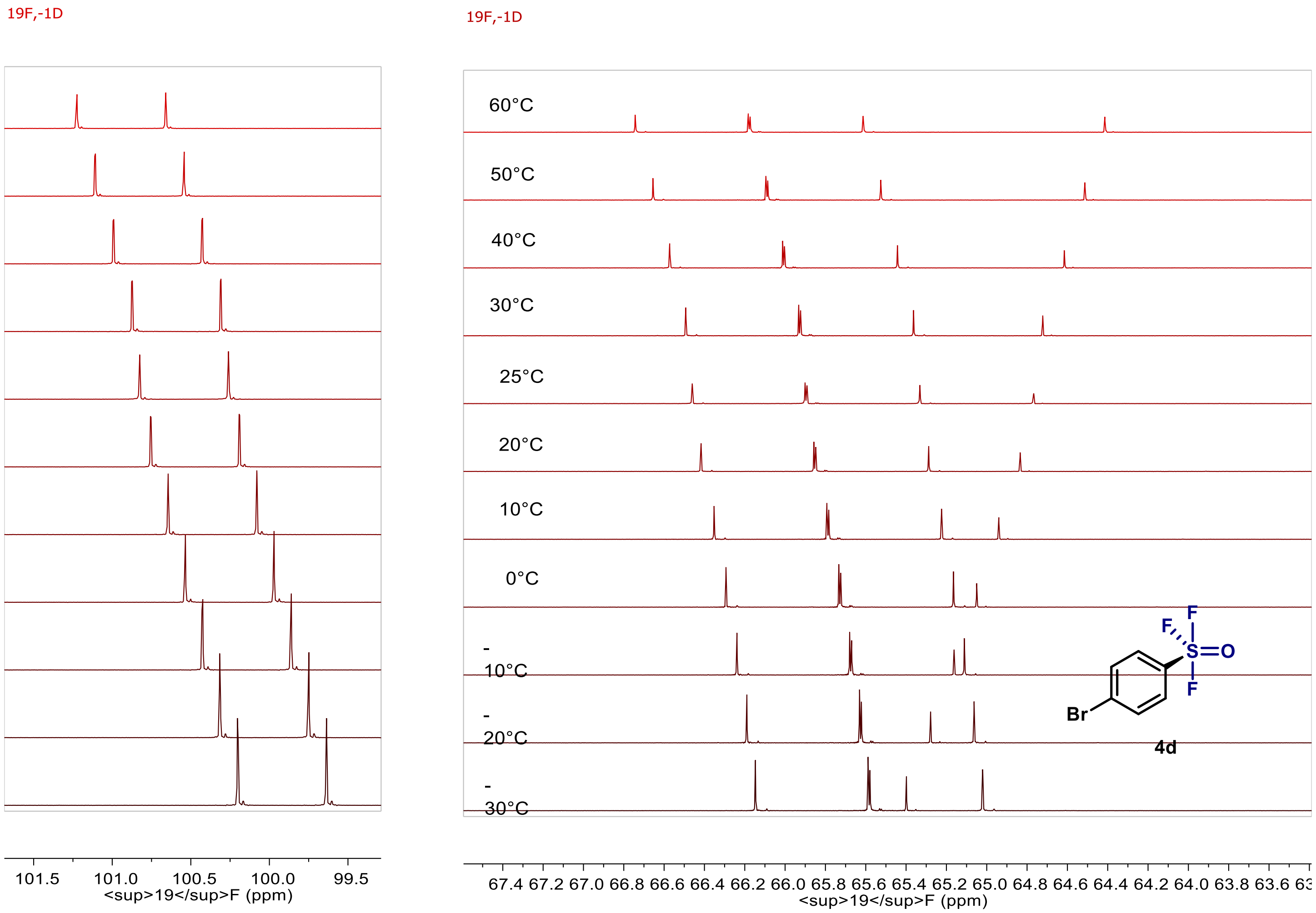

Figure S6. VT ^{19}F NMR of **4d** (from -30 ℃ to 60 ℃).

The AB_2 multiplicity pattern in the ^{19}F NMR spectrum of **4d** indicates the formation of rigid (pseudo)trigonal-bipyramidal coordination with one equatorial and two apical fluorine atoms. Based on the VSEPT theory and the previous literature [17], [19], a *trans*-structure with two F in the axial could be speculated.

Finally, an ESI-MS of **4d** was measured (Figure S7). Although the exact mass of **4d** was not found due to the sensitivity of **4d** towards moisture, a signal of key fragment ($4\text{-BrC}_6H_4SOF_2^+$) was detected.

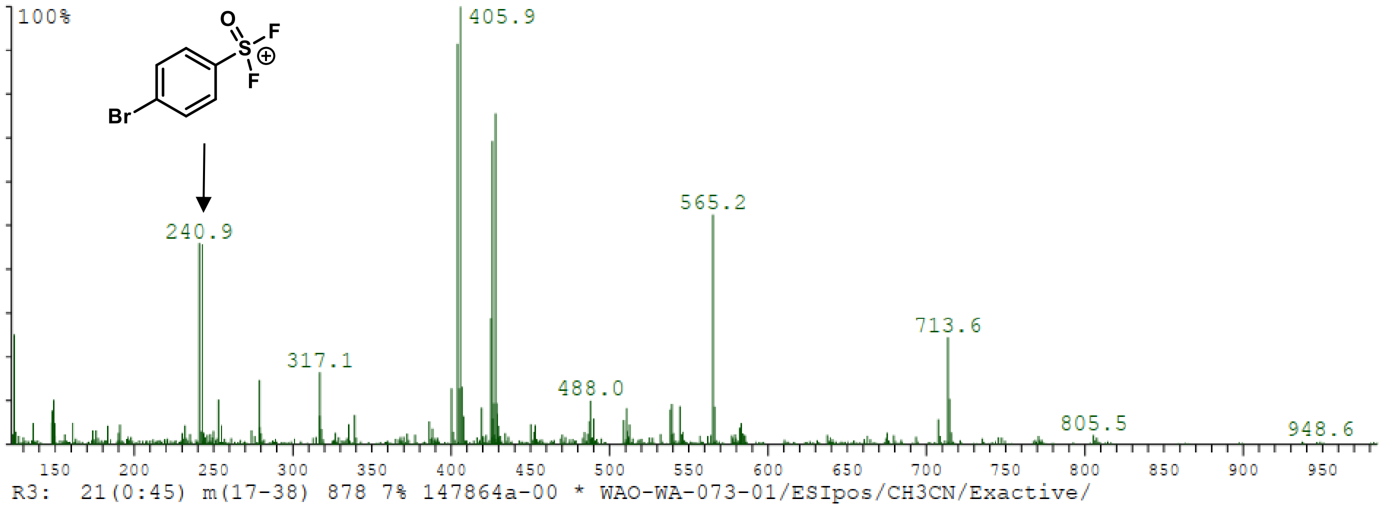

Figure S7. ESI-MS of **4d**.

10. General method for the synthesis of $ArSO_2F$

In a glovebox under Argon, the Ar-S-Phth precursor (**3**, 0.1 mmol, 1 equiv.), trichloroisocyanuric acid (TCICA, 210 mg, 0.9 mmol, 9 equiv.) and rigorously dried KF (93 mg, 16 mmol, 16 equiv.) was added to an oven-dried 12 mL round-bottom reaction vial equipped with a stir bar. Under vigorous stirring, 1 mL dry and degassed MeCN was added to the mixture followed by addition of MeOH solution (1M in MeCN, 0.2 mL). *Note: methanol should be dried over magnesium in advance.* Then the vial was sealed with a septum-pad cap and the reaction was stirred at the room temperature in the glovebox for 24 h. Upon the completion of reaction, the atmosphere in the vial was vented carefully. The resulting suspension was filtered while the precipitate was washed by MeCN (2 mL × 3). The crude product was then concentrated and further purified through a silica column chromatography (Pentane/EtOAc = 10:1).

11. Scope of $ArSO_2F$

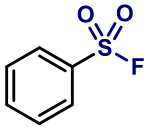

5a

Synthesis of benzenesulfonyl fluoride (5a). The title compound was prepared from Ph-S-Phth (**3a**, 0.1 mmol) according to the general method in section 10. After the reaction, the product was purified through a column chromatography (Pentane/EtOAc = 10:1). Colorless oil was obtained as the desired product. In combination with the yield for **3a**, the three-step total yield: 80%. NMR data are in accordance with the literature report. [20]

^{1}H NMR (300 MHz, $CDCl_3$) δ 8.03 (d, *J* = 7.4 Hz, 2H), 7.79 (t, *J* = 7.2 Hz, 1H), 7.64 (t, *J* = 8.1 Hz, 2H).
^{19}F{^{1}H} NMR (282 MHz, $CDCl_3$) δ 65.8 (s, 1F)
^{13}C NMR (75 MHz, $CDCl_3$) δ 135.5, 133.1 (d, *J* = 24.4 Hz), 129.6, 128.4.
GC-MS (EI) $[M]^+$ 160.0

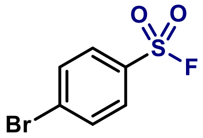

5b

Synthesis of 4-bromophenylsulfonyl fluoride (5b). The title compound was prepared from 4-Br-Ph-S-Phth (**3d**, 0.1 mmol) according to the general method in section 10. After the reaction, the product was purified through a column chromatography (Pentane/EtOAc = 10:1). White solid was obtained as the desired product. In combination with the yield for **3d**, the three-step total yield: 81%. NMR data are in accordance with the literature report. [21]

^{1}H NMR (300 MHz, $CDCl_3$) δ 7.86 – 7.90 (m, 2H), 7.76 – 7.81 (m, 2H).
^{19}F{^{1}H} NMR (282 MHz, $CDCl_3$) δ 66.4 (s, 1F)
^{13}C NMR (75 MHz, $CDCl_3$) δ 133.1, 132.0 (d, *J* = 25.8 Hz), 131.3, 129.8.
GC-MS (EI) $[M]^+$ 237.9

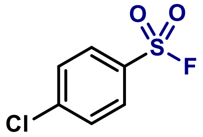

5c

Synthesis of 4-chlorophenylsulfonyl fluoride (5c). The title compound was prepared from 4-Cl-Ph-S-Phth (**3c**, 0.1 mmol) according to the general method in section 10. After the reaction, the product was purified through a

column chromatography. White solid was obtained as the desired product. In combination with the yield for **3c**, the three-step total yield: 72%. NMR data are in accordance with the literature report. [21]

^{1}H NMR (300 MHz, $CDCl_3$) δ 7.94 – 7.98 (m, 2H), 7.59 – 7.64 (m, 2H).
^{19}F{^{1}H} NMR (282 MHz, $CDCl_3$) δ 66.4 (s, 1F)
^{13}C NMR (75 MHz, $CDCl_3$) δ 142.6, 131.6 (d, J = 26.0 Hz), 130.1, 129.8.
GC-MS (EI) $[M]^+$ 194.0

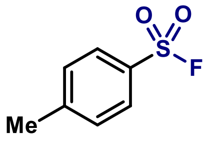

5d

Synthesis of 4-methylphenylsulfonyl fluoride (5d). The title compound was prepared from 4-Me-Ph-S-Phth (**3e**, 0.1 mmol) according to the general method in section 10. After the reaction, the product was purified through a column chromatography (Pentane/EtOAc = 10:1). White solid was obtained as the desired product. In combination with the yield for **3e**, the three-step total yield: 75%. NMR data are in accordance with the literature report. [21]

^{1}H NMR (300 MHz, $CDCl_3$) δ 7.92 – 7.88 (m, 2H), 7.40 – 7.44 (m, 2H), 2.49 (s, 3H).
^{19}F{^{1}H} NMR (282 MHz, $CDCl_3$) δ 66.2 (s, 1F)
^{13}C NMR (75 MHz, $CDCl_3$) δ 147.0, 130.2, 131.1 (d, J = 24.0 Hz), 129.8, 128.4.
GC-MS (EI) $[M]^+$ 174.0

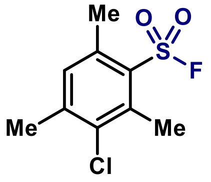

5e

Synthesis of 3-chloro-2,4,6-trimethylphenylsulfonyl fluoride (5e). The title compound was prepared from 2,4,6-triMe-Ph-S-Phth (**3v**, 0.1 mmol) according to the general method in section 10. After the reaction, the product was purified through a column chromatography (Pentane/EtOAc = 10:1). Colorless oil was obtained as the desired product. In combination with the yield for **3v**, the three-step total yield: 58%.

Rf (Pentane/EtOAc = 10:1): 0.54
^{1}H NMR (300 MHz, $CDCl_3$) δ 7.12 – 7.14 (m, 1H), 2.75 (d, J = 2.2 Hz, 3H), 2.63 (dd, J = 1.9, 0.6 Hz, 3H), 2.44 (s, 3H).
^{19}F{^{1}H} NMR (282 MHz, $CDCl_3$) δ 68.7 (s, 1F)

^{13}**C NMR** (75 MHz, $CDCl_3$) δ 143.5, 141.4 (d, *J* = 22.7 Hz) 137.8, 137.7 (d, *J* = 1.4 Hz), 132.9 (d, *J* = 2.0 Hz), 131.8 (d, *J* = 1.4 Hz), 22.6 (d, *J* = 1.4 Hz), 21.6, 19.1 (d, *J* = 3.1 Hz).

HRMS (ESI): Calc'd for $C_9H_{10}ClFO_2S^+$ $[M]^+$ 236.00686; found 236.00688

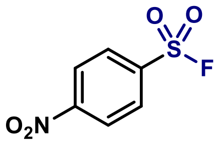

5f

Synthesis of 4-nitrophenylsulfonyl fluoride (5f). The title compound was prepared from 4-NO_2-Ph-S-Phth (**3h**, 0.1 mmol) according to the general method in section 10. After the reaction, the product was purified through a column chromatography (Pentane/EtOAc = 10:1). White solid was obtained as the desired product. In combination with the yield for **3h**, the three-step total yield: 63%. NMR data are in accordance with the literature report. [22]

^{1}**H NMR** (300 MHz, $CDCl_3$) δ 8.47 – 8.52 (m, 2H), 8.22 – 8.27 (m, 2H).

^{19}**F{**^{1}**H} NMR** (282 MHz, $CDCl_3$) δ 66.2 (s, 1F)

^{13}**C NMR** (75 MHz, $CDCl_3$) δ 151.8, 138.4 (d, *J* = 27.2 Hz), 130.0, 124.8.

GC-MS (EI) $[M]^+$ 174.0

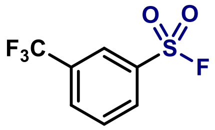

5g

Synthesis of 3-(trifluoromethyl)phenylsulfonyl fluoride (5g). The title compound was prepared from 3-CF_3-Ph-S-Phth (**3i**, 0.1 mmol) according to the general method in section 10. After the reaction, the product was purified through a column chromatography (Pentane/EtOAc = 10:1). White solid was obtained as the desired product. In combination with the yield for **3i**, the three-step total yield: 71%. NMR data are in accordance with the literature report. [23]

^{1}**H NMR** (300 MHz, $CDCl_3$) δ 8.29 (s, 1H), 8.23 (d, *J* = 8.0 Hz, 1H), 8.05 (d, *J* = 7.4 Hz, 1H), 7.82 (d, *J* = 8.0 Hz, 1H).

^{19}**F{**^{1}**H} NMR** (282 MHz, $CDCl_3$) δ 66.2 (s, 1F), -63.1 (s, 3F).

^{13}**C NMR** (75 MHz, $CDCl_3$) δ 134.3 (d, *J* = 26.7 Hz), 132.2 (q, *J* = 3.6 Hz), 131.6, 130.6, 125.6 (q, *J* = 3.6 Hz), 124.5, 119.0 (d, *J* = 272.9 Hz).

GC-MS (EI) $[M]^+$ 228.0

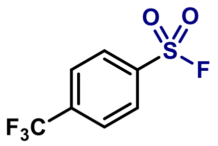

5h, 68%

Synthesis of 4-(trifluoromethyl)phenylsulfonyl fluoride (5h). The title compound was prepared from 4-CF_3-Ph-S-Phth (**3j**, 0.1 mmol) according to the general method in section 10. After the reaction, the product was purified through a column chromatography (Pentane/EtOAc = 10:1). White solid was obtained as the desired product. In combination with the yield for **3j**, the three-step total yield: 68%. NMR data are in accordance with the literature report. [21]

^{1}H NMR (300 MHz, $CDCl_3$) δ 8.10 (d, *J* = 8.3 Hz, 2H), 7.85 (d, *J* = 8.3 Hz, 2H).
^{19}F{^{1}H} NMR (282 MHz, $CDCl_3$) δ 65.8 (s, 1F), -63.6 (s, 3F).
^{13}C NMR (75 MHz, $CDCl_3$) δ 137.2 (d, *J* = 33.7 Hz), 136.6 (d, *J* = 27.5 Hz), 129.1, 126.9 (q, *J* = 3.7 Hz), 122.7 (d, *J* = 273.4 Hz).
GC-MS (EI) $[M]^+$ 228.0

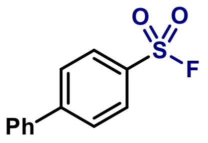

5i

Synthesis of 4-Biphenylsulfonyl fluoride 5i. The title compound was prepared from 4-Ph-Ph-S-Phth (**3n**, 0.1 mmol) according to the general method in section 10. After the reaction, the product was purified through a column chromatography (Pentane/EtOAc = 10:1). White solid was obtained as the desired product. In combination with the yield for **3n**, the three-step total yield: 53%. NMR data are in accordance with the literature report. [22]

^{1}H NMR (300 MHz, $CDCl_3$) δ 8.05 – 8.10 (m, 2H), 7.80 – 7.84 (m, 2H), 7.60 – 7.64 (m, 2H), 7.43 – 7.54 (m, 3H).
^{19}F{^{1}H} NMR (282 MHz, $CDCl_3$) δ 66.4 (s, 1F).
^{13}C NMR (75 MHz, $CDCl_3$) δ 148.6, 138.5, 131.4 (d, *J* = 24.6 Hz), 129.2, 129.1, 129.0, 128.2, 127.4.

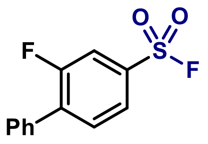

5j

Synthesis of 2-fluoro-[1,1'-biphenyl]-4-sulfonyl fluoride (5j). The title compound was prepared from 2-fluoro-[1,1'-biphenyl]-4-S-Phth (**3m**, 0.1 mmol) according to the general method in section 10. After the reaction, the

product was purified through a column chromatography (Pentane/EtOAc = 10:1). White solid was obtained as the desired product. In combination with the yield for **3m**, the three-step total yield: 60%.

Rf (Pentane/EtOAc = 10:1): 0.55

^{1}H NMR (300 MHz, $CDCl_3$) δ 7.88 (dd, *J* = 8.2, 1.8 Hz, 1H), 7.82 (dd, *J* = 8.2, 1.8 Hz, 1H), 7.71 (t, *J* = 7.7 Hz, 1H), 7.45 – 7.59 (m, 5H).

^{19}F{^{1}H} NMR (282 MHz, $CDCl_3$) δ 66.3 (s, 1F), -112.6 (s, 1F).

^{13}C NMR (75 MHz, $CDCl_3$) δ 160.5, 158.3 (d, *J* = 73.8 Hz), 137.0 (d, *J* = 13.5 Hz), 133.2, 132.1 (d, *J* = 3.7 Hz), 129.4, 129.0 (d, *J* = 3.1 Hz), 128.9, 124.5 (d, *J* = 4.0 Hz), 116.8 (d, *J* = 27.6 Hz).

HRMS (GC-EI): Calc'd for $C_{12}H_8F_2O_2S^+$ $[M]^+$ 254.02076; found 254.02101.

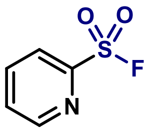

5k

Synthesis of 2-pyridinylsulfonyl fluoride (5k). The title compound was prepared from 2-Py-S-Phth (**3o**, 0.1 mmol) according to the general method in section 10. After the reaction, the product was purified through a column chromatography (Pentane/EtOAc = 10:1). Colorless oil was obtained as the desired product. In combination with the yield for **3o**, the three-step total yield: 63%. NMR data are in accordance with the literature report. [24]

^{1}H NMR (300 MHz, $CDCl_3$) δ 8.96 (d, *J* = 4.6 Hz, 1H), 8.15 (d, *J* = 7.7 Hz, 1H), 8.07 (d, *J* = 7.7 Hz, 1H), 7.73 (dd, *J* = 7.7, 4.6 Hz, 1H).

^{19}F{^{1}H} NMR (282 MHz, $CDCl_3$) δ 55.8 (s, 1F).

^{13}C NMR (75 MHz, $CDCl_3$) δ 151.5 (d, *J* = 30.4 Hz), 151.0 (d, *J* = 1.1 Hz), 138.6, 129.1, 124.1 (d, *J* = 2.1 Hz).

GC-MS (EI) $[M]^+$ 161.0

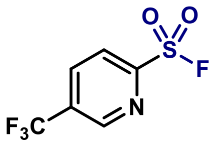

5l

Synthesis of 5-(trifluoromethyl)-2-pyridinesulfonyl fluoride (5l). The title compound was prepared from 5-CF_3-2-Py-S-Phth (**3r**, 0.1 mmol) according to the general method in section 10. After the reaction, the product was purified through a column chromatography (Pentane/EtOAc = 10:1). White solid was obtained as the desired product. In combination with the yield for **3r**, the three-step total yield: 61%.

Rf (Pentane/EtOAc = 10:1): 0.55

^{1}H NMR (300 MHz, $CDCl_3$) δ 9.11 (s, 1H), 8.27 – 8.35 (m, 2H).

^{19}F{^{1}H} NMR (282 MHz, $CDCl_3$) δ 55.7 (s, 1F), 62.9 (s, 3F).

^{13}C NMR (75 MHz, $CDCl_3$) δ 154.2 (d, *J* = 32.4 Hz), 148.0 (qd, *J* = 3.8, 1.1 Hz), 136.3 (q, *J* = 3.5 Hz), 131.7 (q, *J* = 34.4 Hz), 124.0 (d, *J* = 2.2 Hz), 122.1 (d, *J* = 273.7 Hz).

HRMS (ESI): Calc'd for $C_6H_4F_4NO_2S^+$ $[M+H]^+$ 229.98934; found 229.98655

12. General method for the synthesis of $ArSF_4Cl$

In a glovebox under Argon, the Ar-S-Phth precursor (**3**, 0.2 mmol, 1 equiv.), trichloroisocyanuric acid (TCICA, 840 mg, 3.6 mmol, 18 equiv.) and rigorously dried KF (372 mg, 64 mmol, 32 equiv.) was added to an oven-dried 12 mL round-bottom reaction vial equipped with a stir bar. Under vigorous stirring, 2 mL dry and degassed MeCN was added to the mixture followed by addition of TFA solution (0.1 M in MeCN, 0.2 mL). Then the vial was sealed with a septum-pad cap and the reaction was stirred at the room temperature in the glovebox for 24 h. Upon the completion of reaction, the atmosphere in the vial was vented carefully and the internal standard α, α, α-trifluorotoluene was added into the mixture. After 10 min stirring, an aliquot of the resulting solution was filtered under argon. The NMR sample was prepared with 0.4 mL of the filtered aliquot and 0.1 mL CD_3CN for ^{19}F NMR yield determination.

Please note, although the $ArSF_4Cl$ is not so sensitive to the moisture (for example, **6i** remained untouched even in ‘wet MeCN’ with 10% water), dry reaction vials, dry solvent, experiment and work-up process under the argon benefited the reaction. Unfortunately, these compounds are still not stable enough for column chromatography isolation.

13. Scope of $ArSF_4Cl$

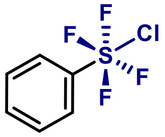

6a

Phenyltetrafluoro-λ^6-sulfanyl chloride **6a** was prepared according to the general method in section 12. The product is consistent with previously reported characterization data. [6]

^{19}F{^{1}H} NMR (282 MHz, CH_3CN/CD_3CN) δ 136.7 (s, 4F).

^{19}F NMR spectra (282 MHz)

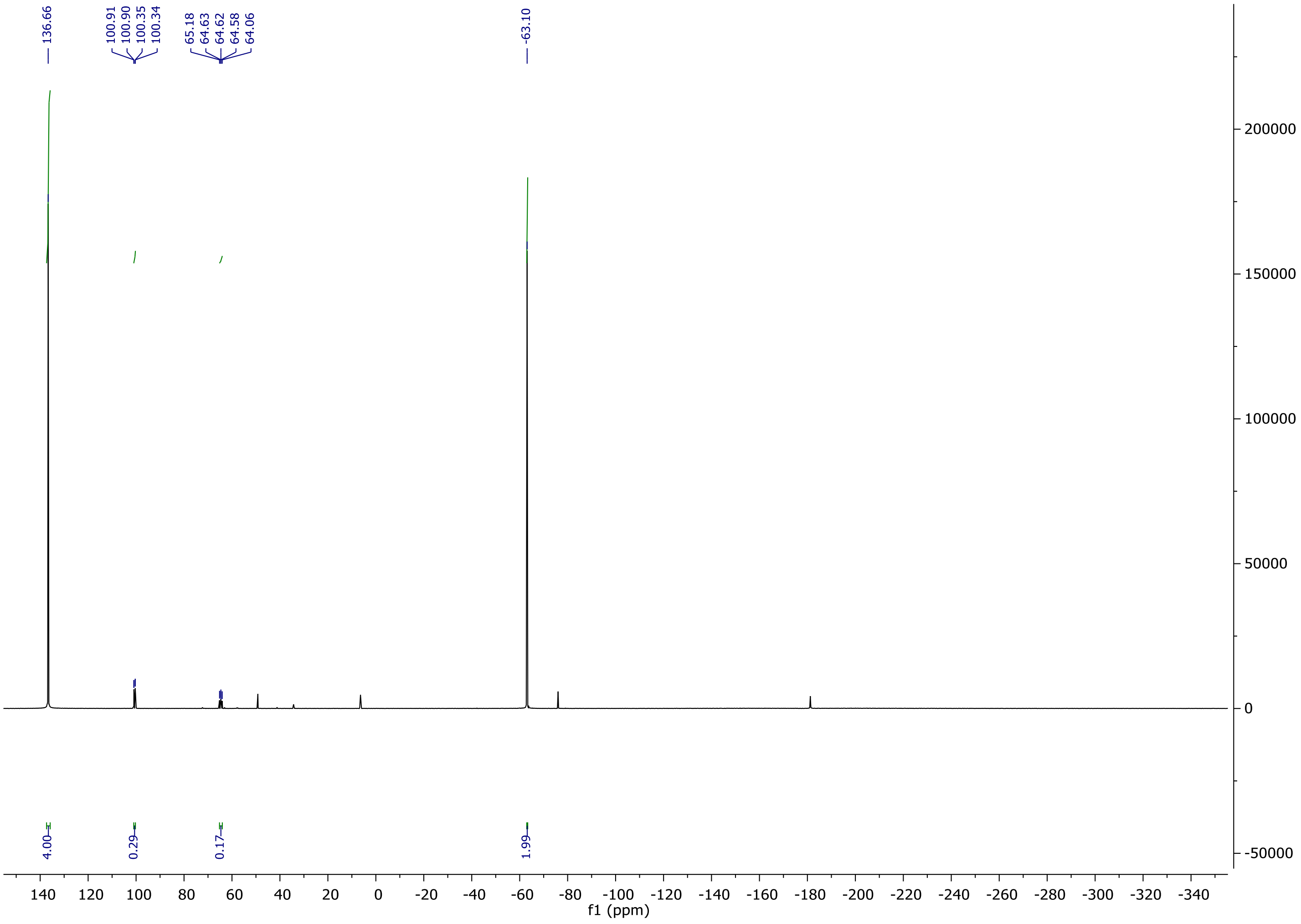

Note. Based on internal standard α,α,α-trifluorotoluene (at –63.10 ppm, 16.9 mg, 0.116 mmol), ^{19}F NMR yield: 85%. In combination with the yield for **3a**, the three-step total yield: 69%. Doublet at 100.6 ppm and triplet at 64.6 ppm belong to the signal of $PhSOF_3$ (**4a**, yield from **3a**, 12%); Singlet at 64.6 ppm is the signal of $PhSO_2F$ (**5a,** yield from **3a**, yield 2%).

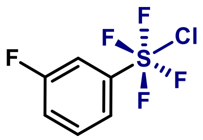

6b

3-Fluorophenyltetrafluoro-λ[6]-sulfanyl chloride **6b** was prepared according to the general method in section 12. The product is consistent with previously reported characterization data. [6]

^{19}F{^{1}H} NMR (282 MHz, CH_3CN/CD_3CN) δ 136.0 (s, 4F), –111.4 (s, 1F).

^{19}F NMR spectra (282 MHz)

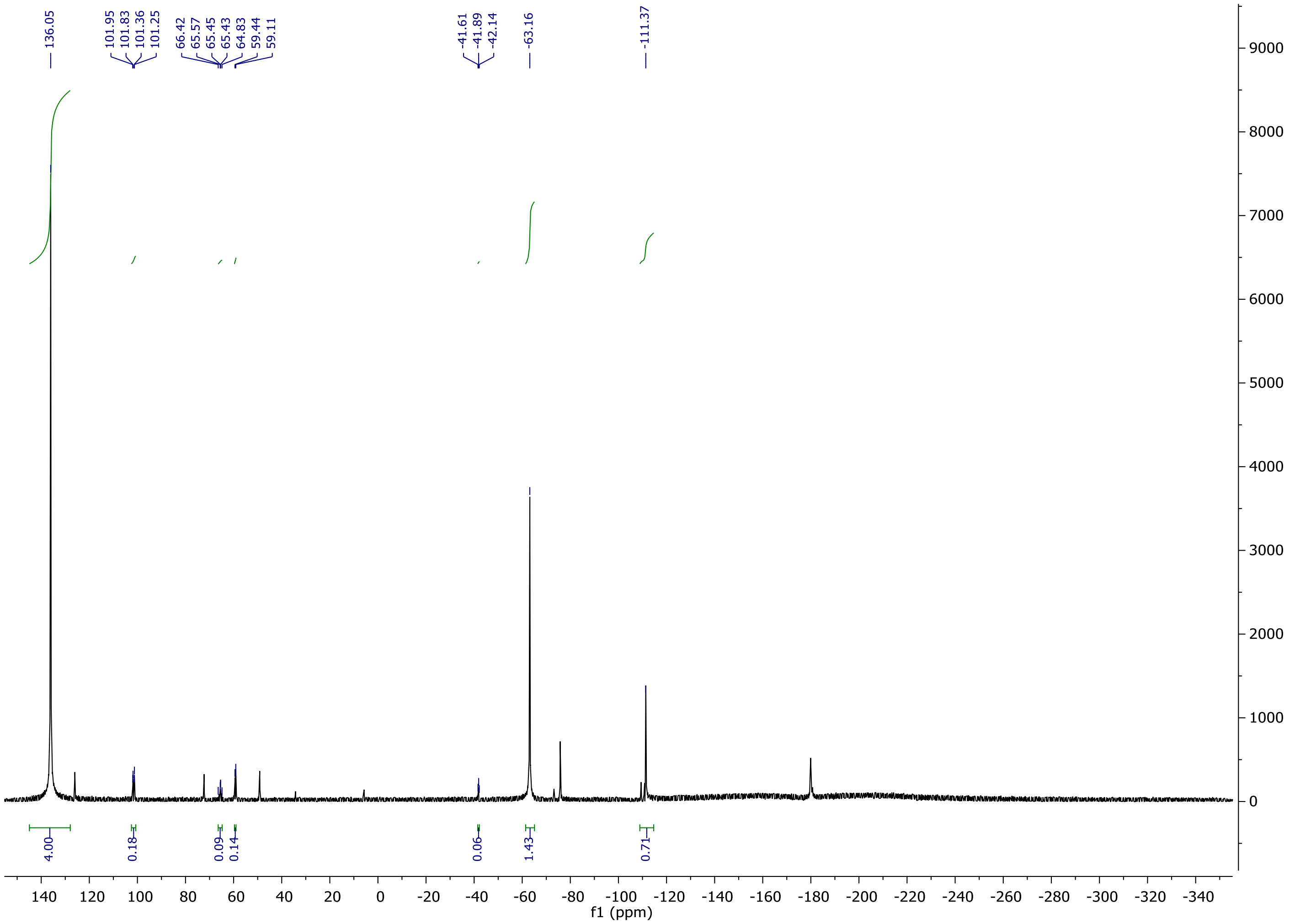

Note. Based on internal standard α,α,α-trifluorotoluene (at –63.16 ppm, 12.0 mg, 0.082 mmol), ^{19}F NMR yield: 87%. In combination with the yield for **3b**, the three-step total yield: 71%. Doublet at 101.5 ppm and triplet at 65.5 ppm belong to the signal of 3-F-$PhSOF_3$ (**4b**, yield from **3b**, 7%); Doublet at 59.3 ppm and triplet at -41.9 ppm belong to the signal of 3-F-$PhSF_3$ (yield from **3b**, 5%).

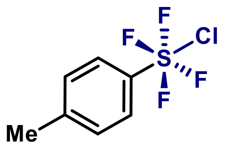

6c

4-Methylphenyltetrafluoro-λ[6]-sulfanyl chloride **6c** was prepared according to the general method in section 12. The product is consistent with previously reported characterization data. [25]
^{19}F{^{1}H} NMR (282 MHz, CH_3CN/CD_3CN) δ 137.7 (s, 4F).

^{19}F NMR spectra (282 MHz)

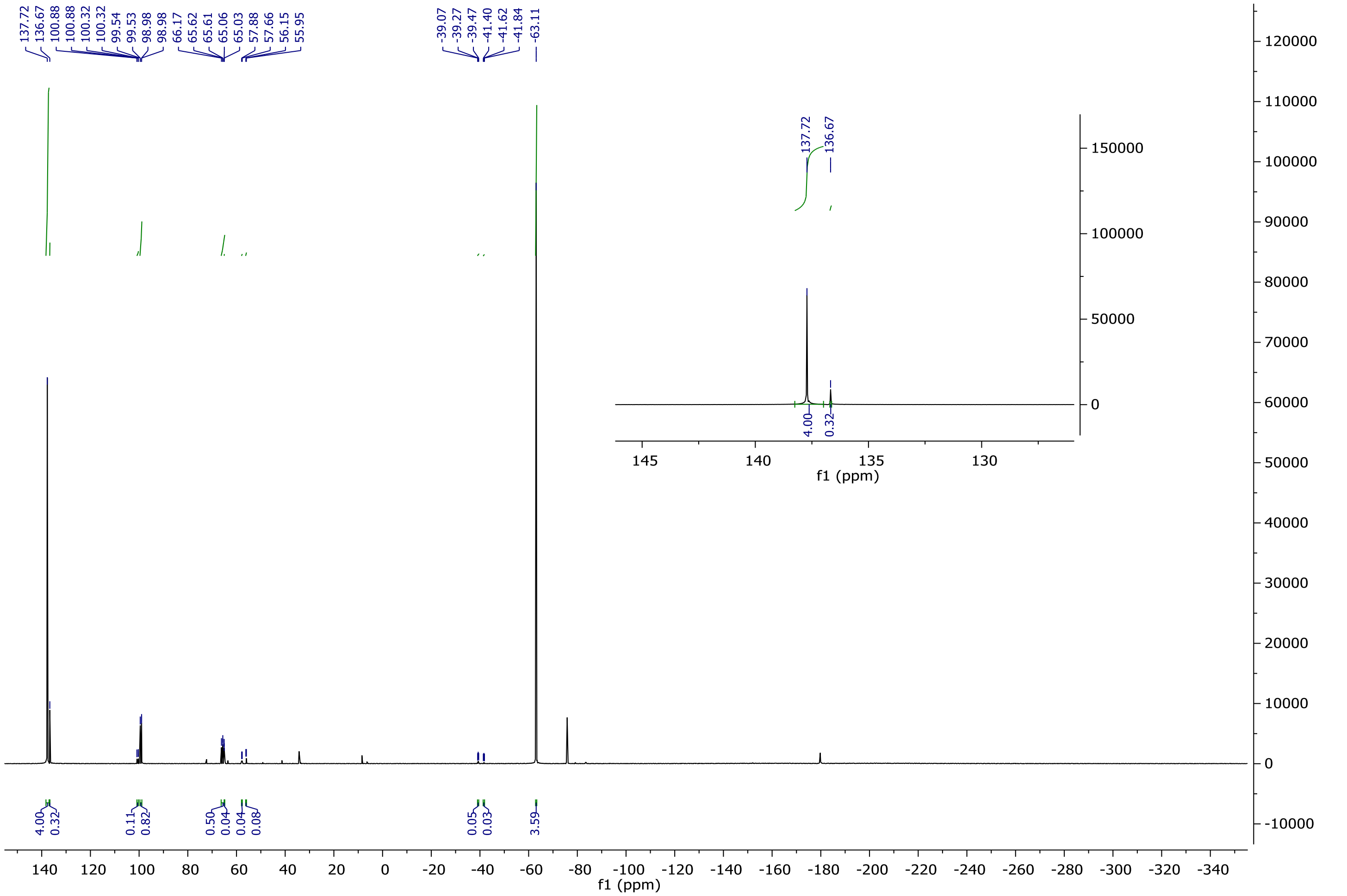

Note. For the substrates with electron-donating substituent groups (–Me as well as –OMe) at para-position, an interesting phenomenon was observed. For both of the desired product (Ar-SF_4Cl) and byproducts ($ArSOF_3$ and $ArSF_3$), the signals of an isomer were found very close to the signals of major product. The ratios of major product to isomer are **6c** (at 137.7 ppm) : **6c’** (at 136.7 ppm) = 12.5:1. However, the structure of isomer and the reason leading to this phenomenon is still unclear. Based on internal standard α,α,α-trifluorotoluene (at –63.11 ppm, 18.1 mg, 0.124 mmol), ^{19}F NMR yield of **6c**: 52%. In combination with the yield for **3e**, the three-step total yield: 44%. The yield of 4-Me-$PhSOF_3$ (99.3 ppm, d, 2F; 65.6 ppm, t, 1F) and its isomer (101.6 ppm, d, 2F; 65.2 ppm, t, 1F) from **3e** is 24% (7:1). The signals at 65.0 (s), 56.0 (d) & –39.3 (t) and 57.8 (d) & –41.6 (t) belong to 4-Me-SO_2F, 4-Me-$PhSF_3$ and its isomer, respectively.

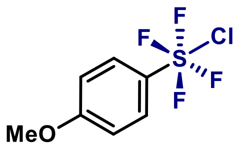

6d

4-Methoxyphenyltetrafluoro-λ[6]-sulfanyl chloride **6d** was prepared according to the general method in section 12.

^{19}F{^{1}H} NMR (282 MHz, CH_3CN/CD_3CN) δ 137.7 (s, 4F).

^{19}F NMR spectra (282 MHz)

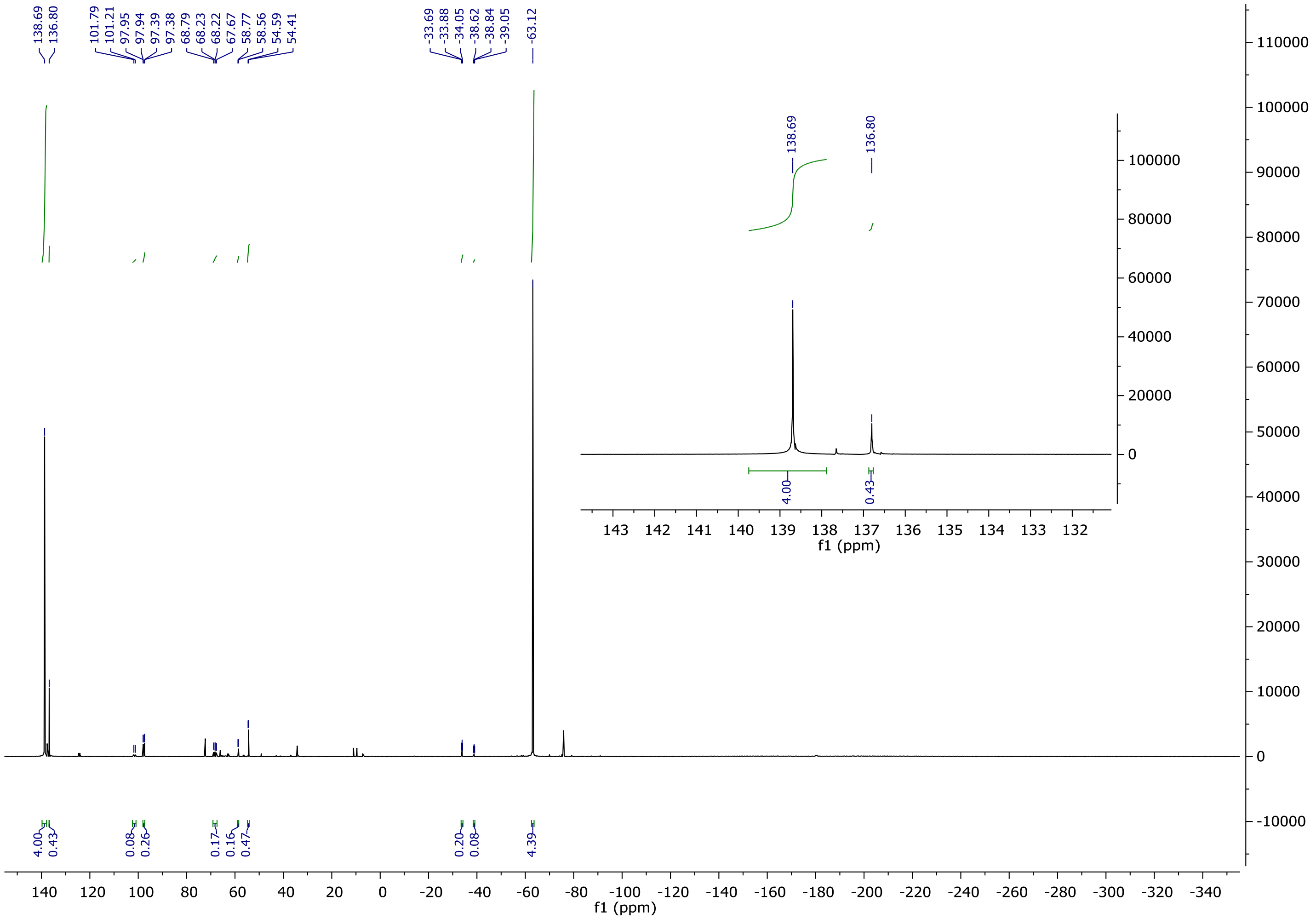

Note. For the substrates with electron-donating substituent groups (–OMe as well as –Me) at para-position, an interesting phenomenon was observed. For both of the desired product (Ar-SF_4Cl) and byproducts ($ArSOF_3$ and $ArSF_3$), the signals of an isomer were found very close to the signals of major product. The ratios of major product to isomer are **6d** (at 138.7 ppm) : **6d'** (at 136.8 ppm) = 9.3:1. However, the structure of isomer and the reason leading to this phenomenon is still unclear. Based on internal standard α,α,α-trifluorotoluene (at –63.12 ppm, 20.5 mg, 0.140 mmol), ^{19}F NMR yield of **6d**: 48%. In combination with the yield for **3g**, the three-step total yield: 37%. The yield of 4-MeO-PhSOF_3 (97.7 ppm, d, 2F; 68.2 ppm, t, 1F) and its isomer (101.6 ppm, d, 2F; 67.8 ppm, t, 1F) from **3g** is 8% (3.25:1). The singlet at 66.1 is the signal of 4-MeOPhSO_2F; The signals of 56.0 (d) & –39.3 (t) and 57.8 (d) & –41.6 (t) belong to 4-Me-PhSF_3 and its isomer with the yield of 15%.

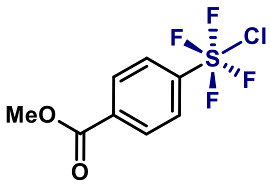

6e

Methyl 4-benzoatetetrafluoro-λ[6]-sulfanyl chloride **6e** was prepared according to the general method in section 12.

^{19}F{^{1}H} NMR (282 MHz, CH_3CN/CD_3CN) δ 135.5 (s, 4F).

^{19}F NMR spectra (282 MHz)

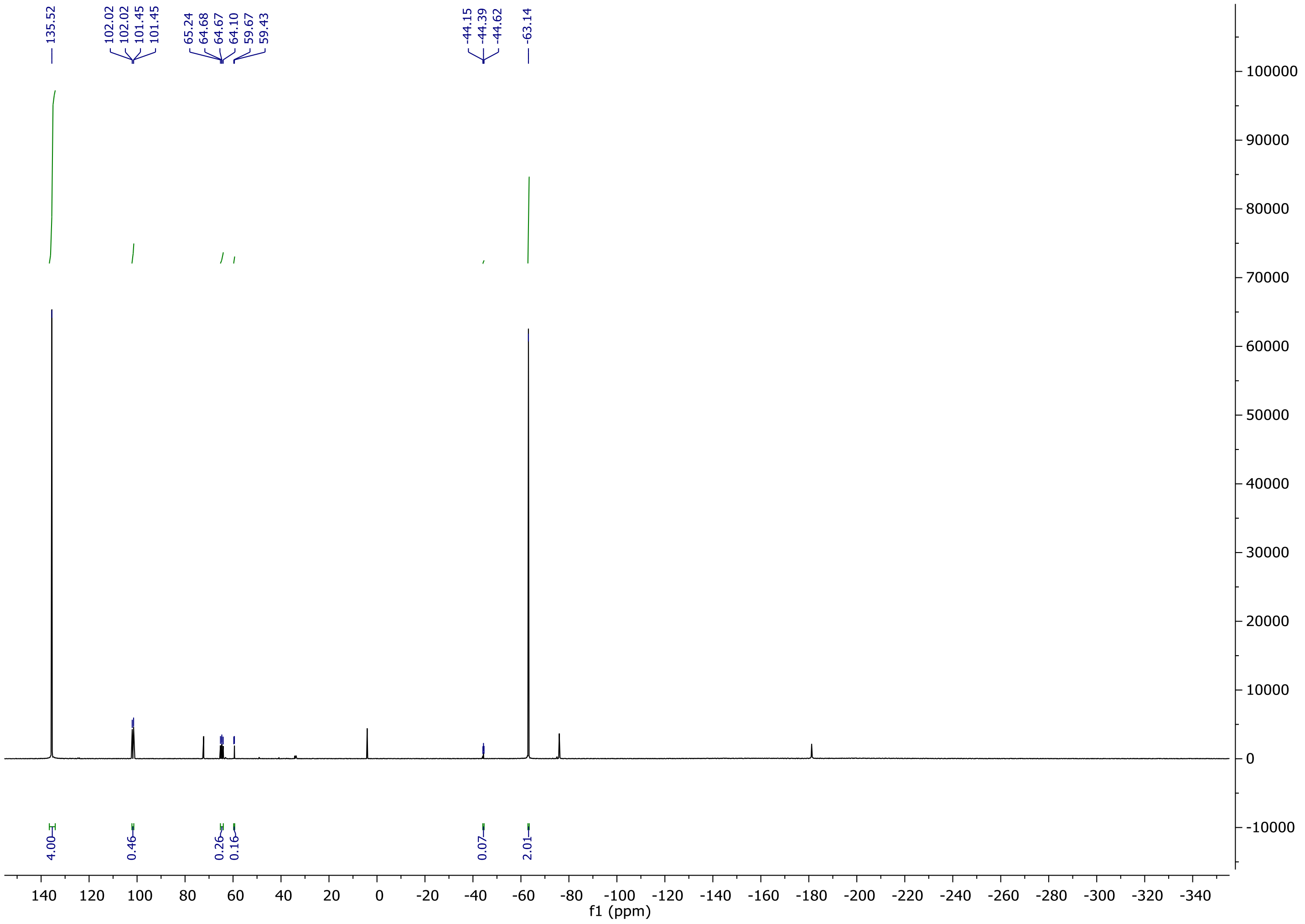

Note. Based on internal standard α, α, α-trifluorotoluene (at –63.14 ppm, 14.9 mg, 0.102 mmol), ^{19}F NMR yield: 77%. In combination with the yield for **3l**, the three-step total yield: 65%. Doublet at 101.7 ppm and triplet at 64.7 ppm belong to the signal of 4-MeOOC-$PhSOF_3$ (**4l**, yield from **3l**, 15%); Doublet at 59.5 ppm and triplet at –44.4 ppm belong to the signal of 4-MeOOC-$PhSF_3$ (yield from **3l**, 5%).

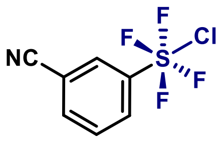

6f

3-Nitrilphenyltetrafluoro-λ^6-sulfanyl chloride **6f** was prepared according to the general method in section 12.

^{19}F{^{1}H} NMR (282 MHz, CH_3CN/CD_3CN) δ 135.3 (s, 4F).

^{19}F NMR spectra (282 MHz)

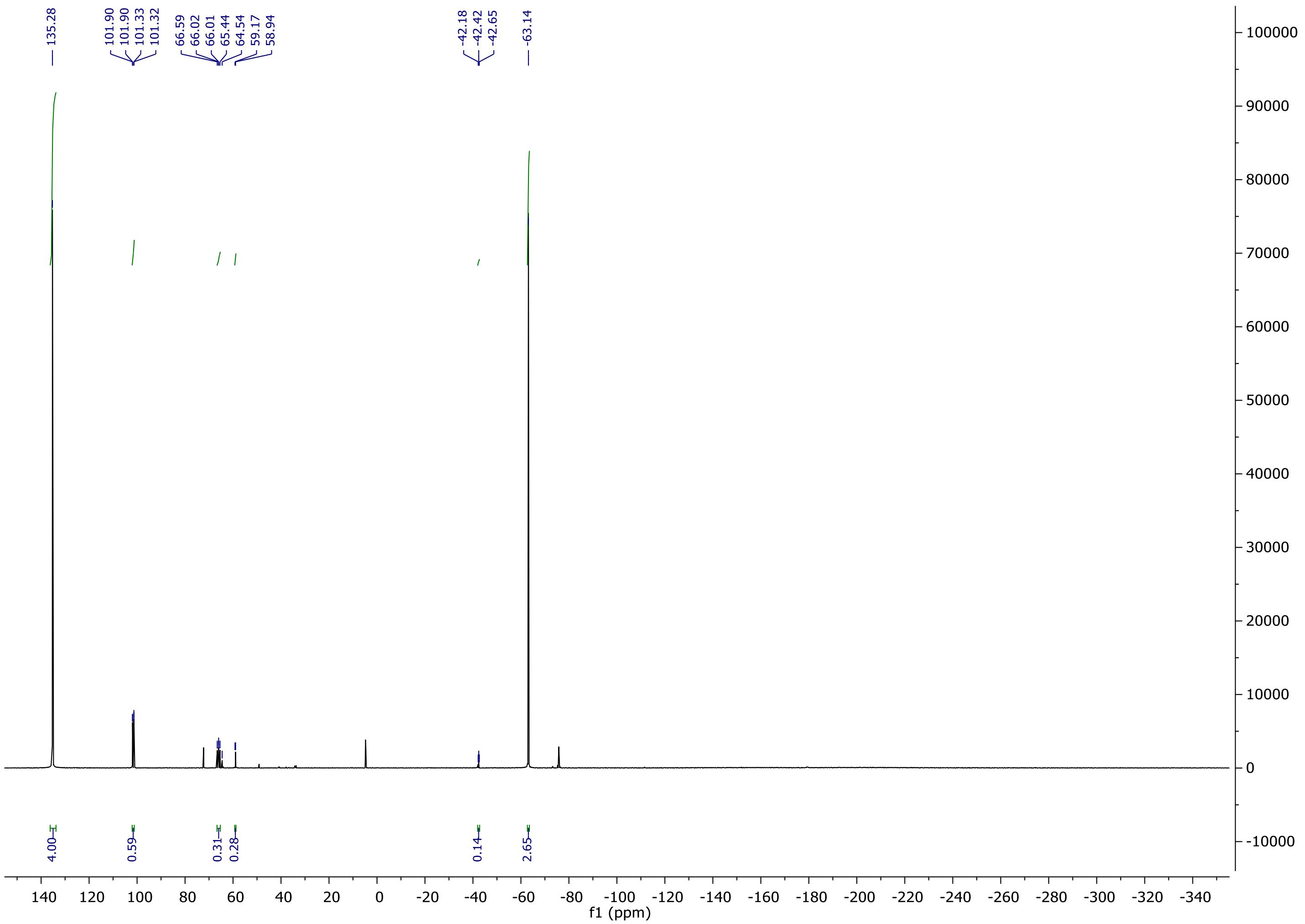

Note. Based on internal standard α,α,α-trifluorotoluene (at –63.14 ppm, 18.0 mg, 0.124 mmol), ^{19}F NMR yield: 70%. In combination with the yield for **3k**, the three-step total yield: 55%. Doublet at 101.6 ppm and triplet at 66.0 ppm belong to the signal of 3-CN-$PhSOF_3$ (**4k**, yield from **3k**, 18%); Doublet at 59.0 ppm and triplet at -42.4 ppm belong to the signal of 3-CN-$PhSF_3$ (yield from **3k**, 8%).

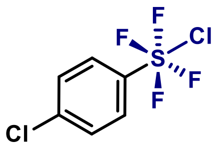

6g

4-Chlorophenyltetrafluoro-λ[6]-sulfanyl chloride **6g** was prepared according to the general method in section 12. The product is consistent with previously reported characterization data. [6]

$^{19}F\{^{1}H\}$ NMR (282 MHz, CH_3CN/CD_3CN) δ 136.8 (s, 4F).

^{19}F NMR spectra (282 MHz)

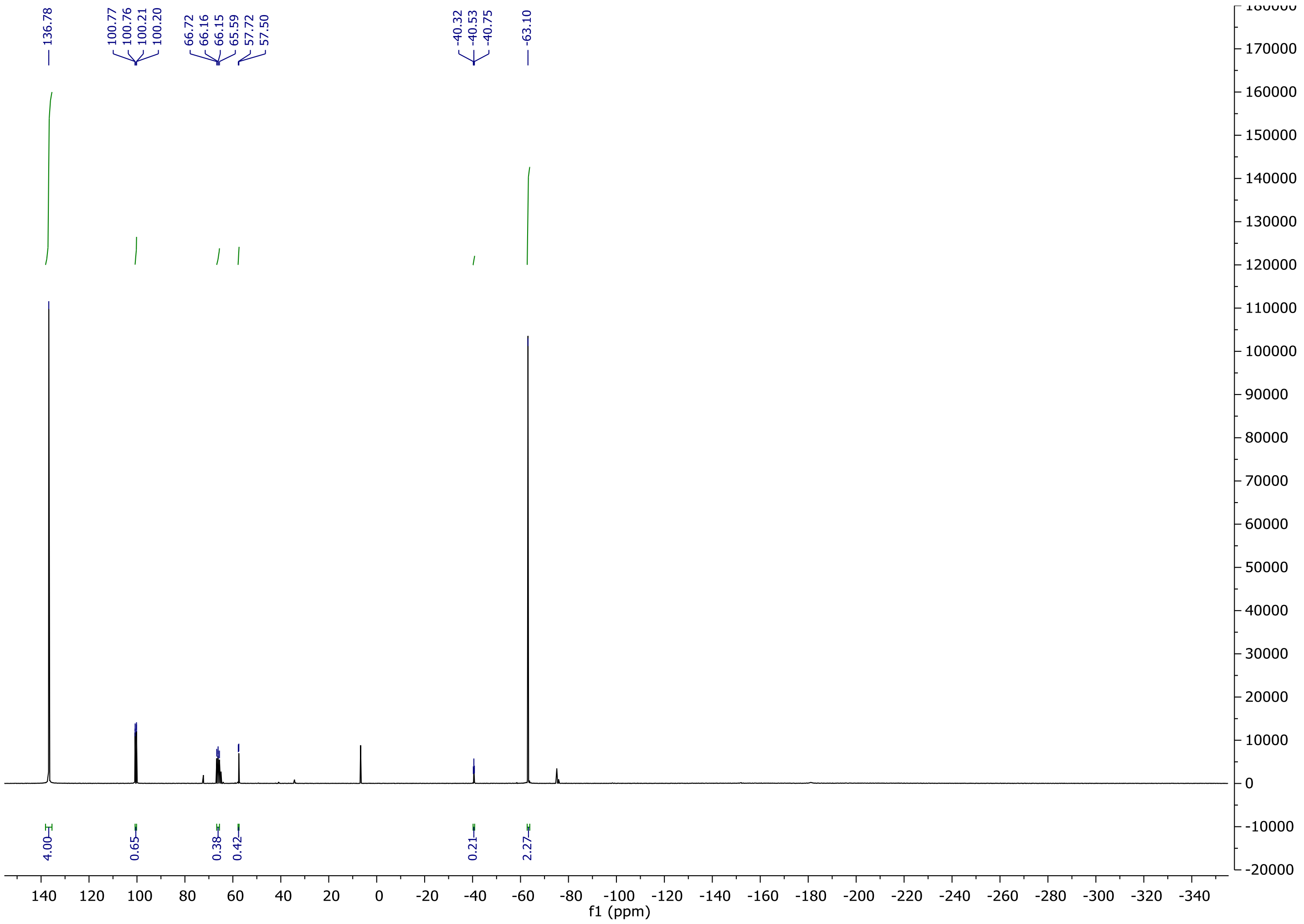

Note. Based on internal standard α,α,α-trifluorotoluene (at –63.10 ppm, 14.9 mg, 0.102 mmol), ^{19}F NMR yield: 67%. In combination with the yield for **3c**, the three-step total yield: 55%. Doublet at 100.5 ppm and triplet at 66.1 ppm belong to the signal of 4-Cl-$PhSOF_3$ (**4c**, yield from **3c**, 20%); Doublet at 57.6 ppm and triplet at –40.5 ppm belong to the signal of 4-Cl-$PhSF_3$ (yield from **3c**, 12%).

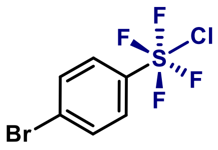

6h

4-Bromophenyltetrafluoro-λ^6-sulfanyl chloride **6h** was prepared according to the general method in section 12. The product is consistent with previously reported characterization data. [6]

^{19}F{^{1}H} NMR (282 MHz, CH_3CN/CD_3CN) δ 136.6 (s, 4F).

^{19}F NMR spectra (282 MHz)

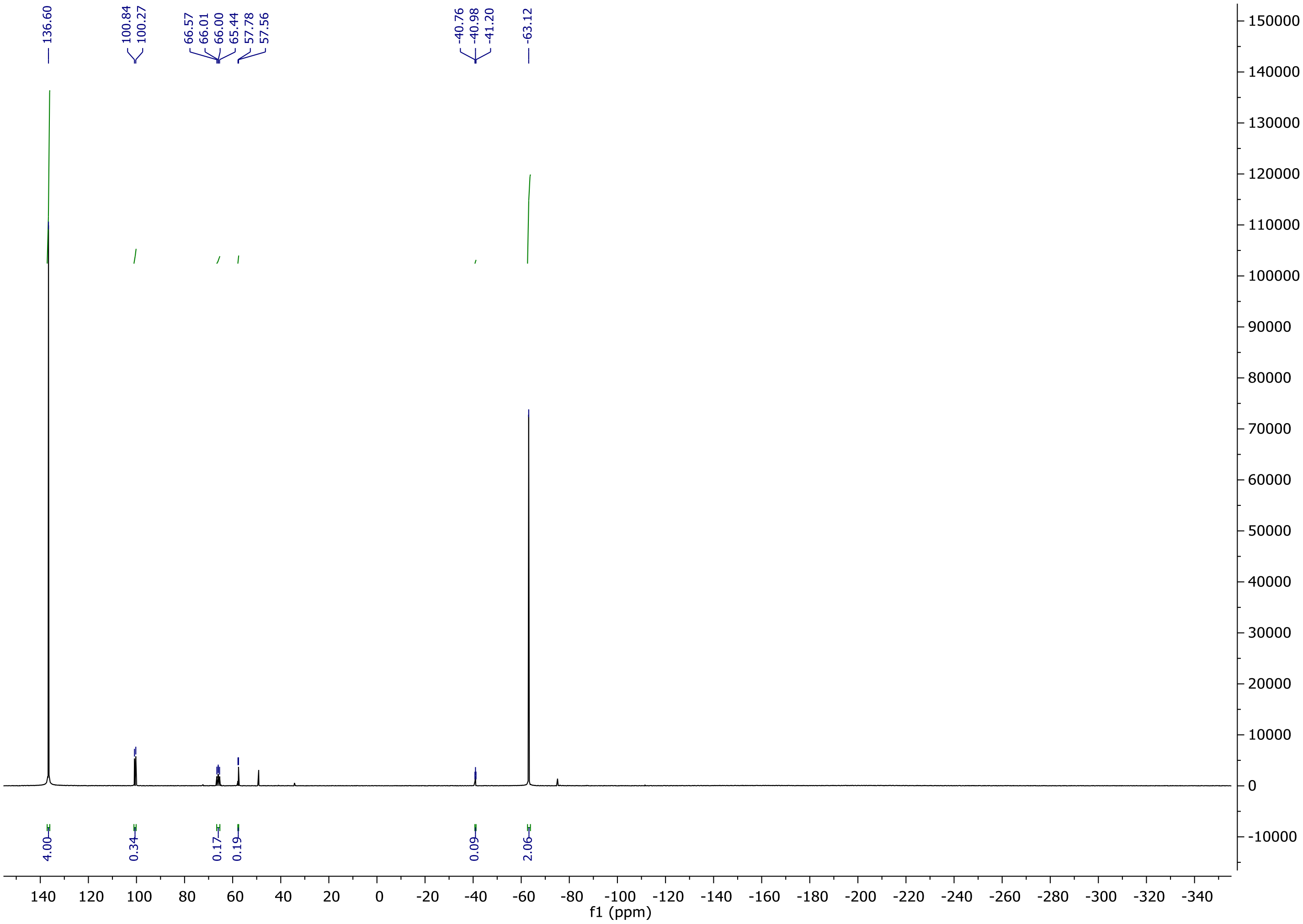

Note. Based on internal standard α,α,α-trifluorotoluene (at –63.12 ppm, 15.2 mg, 0.104 mmol), ^{19}F NMR yield: 76%. In combination with the yield for **3d**, the three-step total yield: 65%. Doublet at 100.6 ppm and triplet at 66.0 ppm belong to the signal of 4-Br-$PhSOF_3$ (**4d**, yield from **3d**, 13%); Doublet at 57.7 ppm and triplet at –41.0 ppm belong to the signal of 4-Br-$PhSF_3$ (yield from **3d**, 7%).

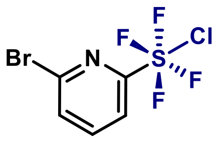

6i

6-Bromo-2-pyridinyltetrafluoro-λ[6]-sulfanyl chloride **6i** was prepared according to the general method in section 12. The product is consistent with previously reported characterization data. [26]

^{19}F{^{1}H} NMR (282 MHz, CH_3CN/CD_3CN) δ 124.2 (s, 4F).

^{19}F NMR spectra (282 MHz)

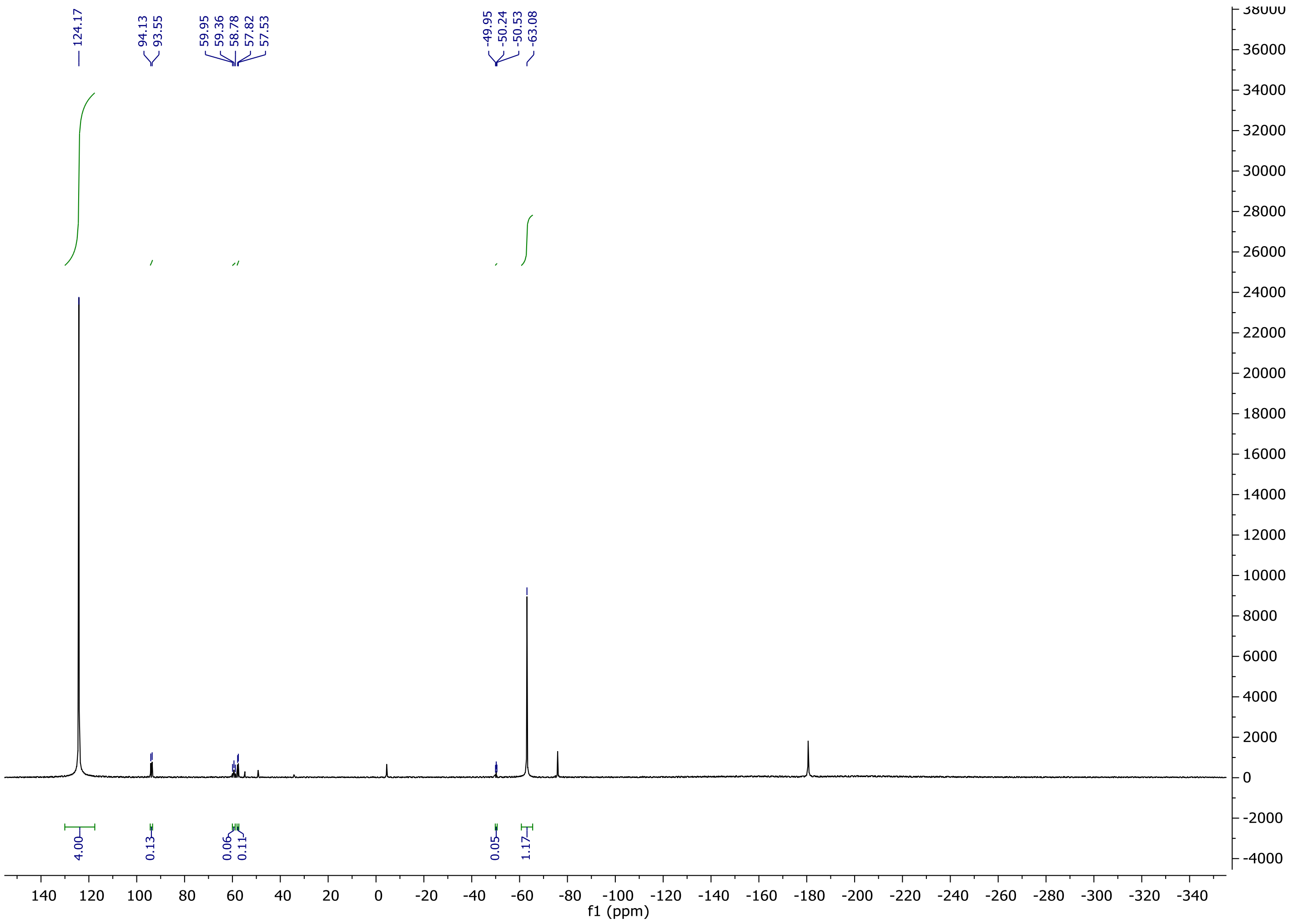

Note. Based on internal standard α,α,α-trifluorotoluene (at –63.08 ppm, 10.2 mg, 0.070 mmol), ^{19}F NMR yield: 90%. In combination with the yield for **3p**, the three-step total yield: 72%. Doublet at 93.8 ppm and triplet at 59.7 ppm belong to the signal of 6-Br-2-$PySOF_3$ (**4p**, yield from **3p**, 5%); Doublet at 57.7 ppm and triplet at –50.2 ppm belong to the signal of 6-Br-2-$PySF_3$ (yield from **3p**, 4%).

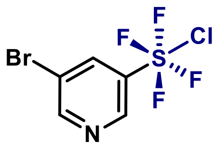

6j

5-Bromo-3-pyridinyltetrafluoro-λ[6]-sulfanyl chloride **6j** was prepared according to the general method in section 12.

^{19}F{^{1}H} NMR (282 MHz, CH_3CN/CD_3CN) δ 137.1 (s, 4F).

^{19}F NMR spectra (282 MHz)

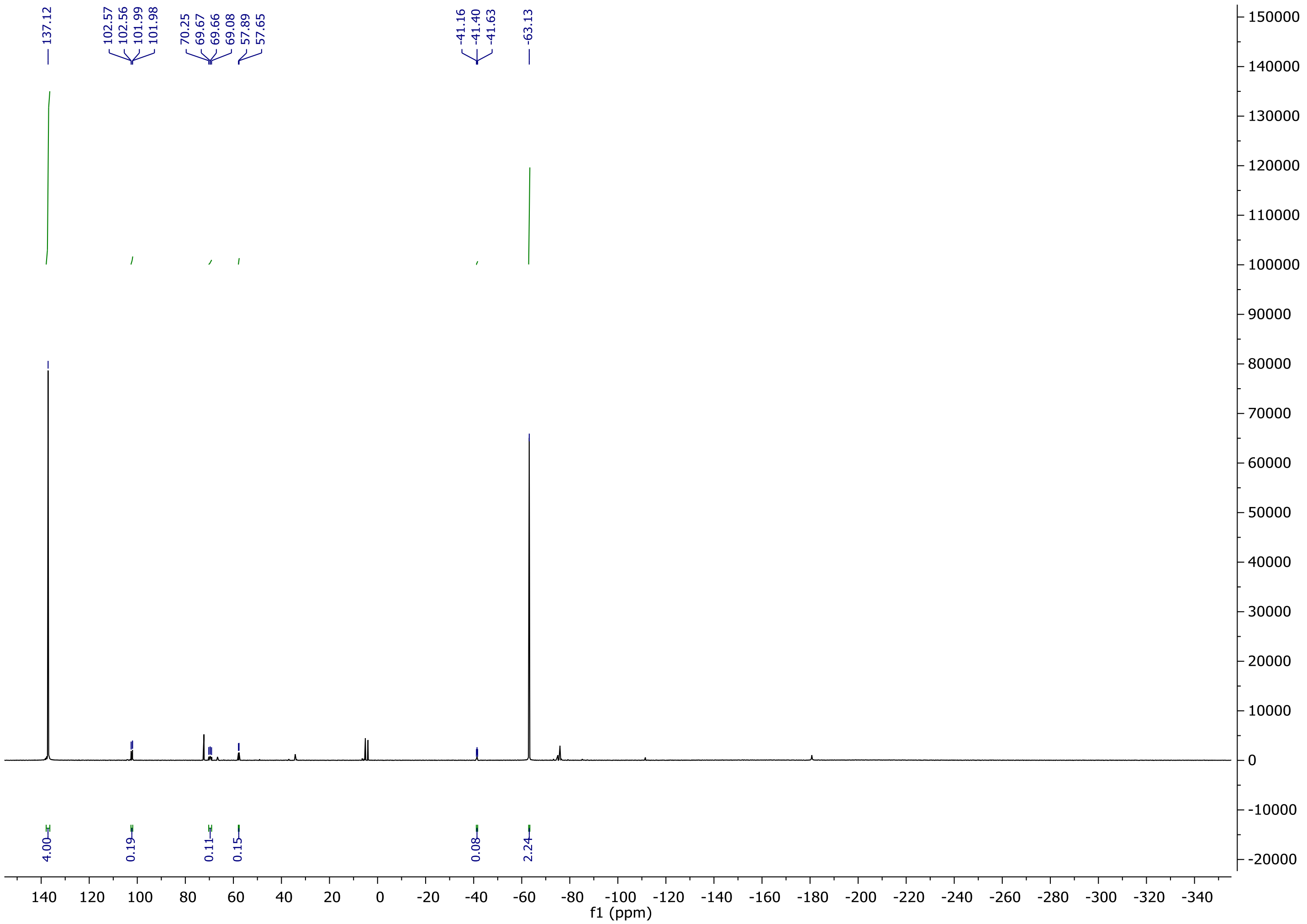

Note. Based on internal standard α,α,α-trifluorotoluene (at –63.13 ppm, 17.8 mg, 0.122 mmol), ^{19}F NMR yield: 82%. In combination with the yield for **3q**, the three-step total yield: 58%. Doublet at 102.3 ppm and triplet at 69.7 ppm belong to the signal of 5-Br-3-$PySOF_3$ (**4q**, yield from **3q**, 7%); Doublet at 57.8 ppm and triplet at -41.4 ppm belong to the signal of 6-Br-3-$PySF_3$ (yield from **3q**, 6%).

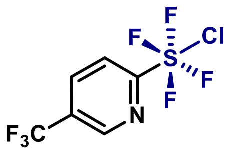

6k

5-(trifluoromethyl)-2-pyridinyltetrafluoro-λ^6-sulfanyl chloride **6k** was prepared according to the general method in section 12.

^{19}F{^{1}H} NMR (282 MHz, CH_3CN/CD_3CN) δ 123.2 (s, 4F), –63.22 (s, 3F).

^{19}F NMR spectra (282 MHz)

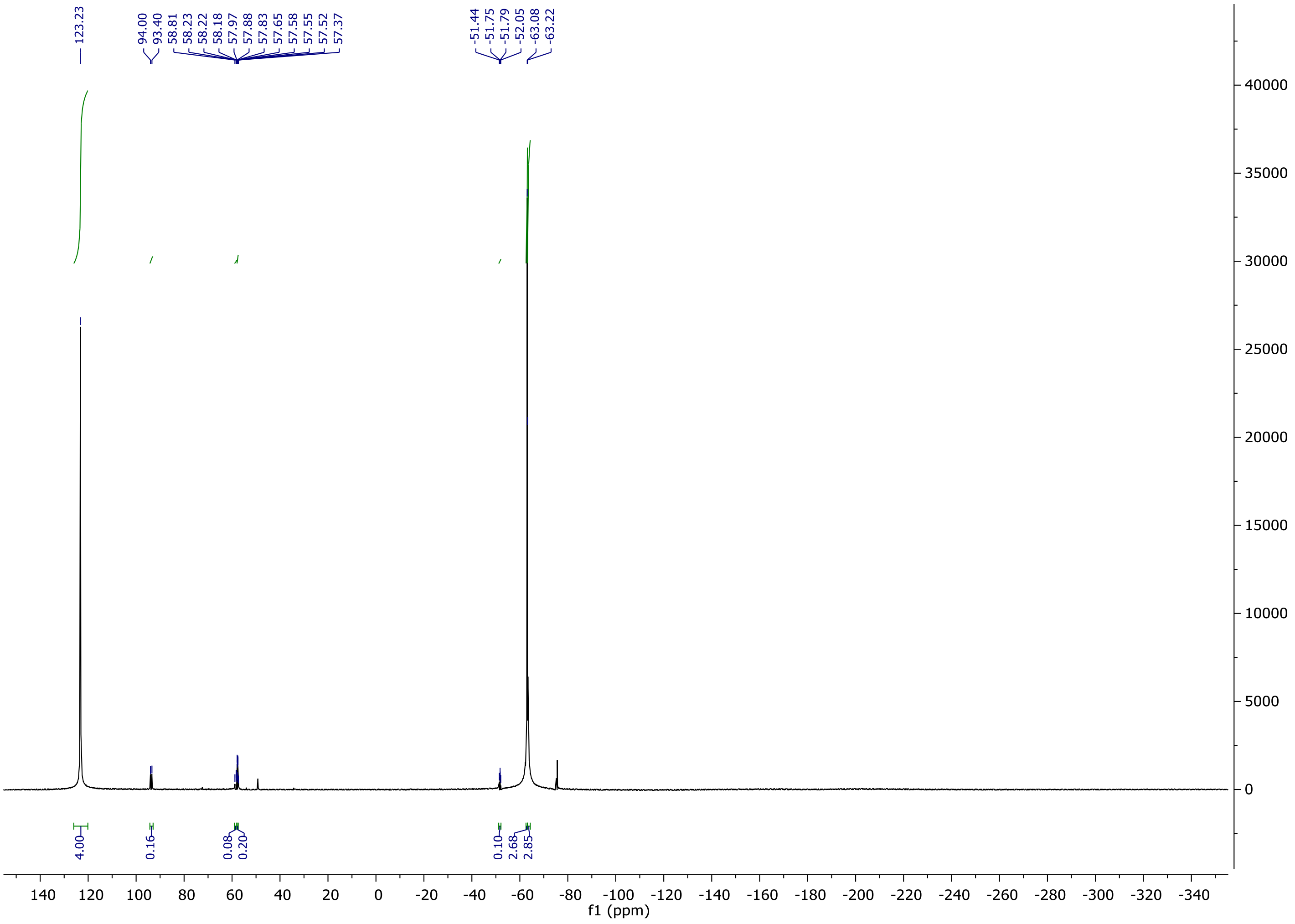

Note. Based on internal standard α,α,α-trifluorotoluene (at –63.08 ppm, 22.2 mg, 0.152 mmol), ^{19}F NMR yield: 85%. In combination with the yield for **3r**, the three-step total yield: 59%. Doublet at 93.7 ppm and triplet at 58.2 ppm belong to the signal of 5-CF_3-2-PySOF_3 (**4r**, yield from **3r**, 7%); Doublet at 57.7 ppm and triplet at –51.8 ppm belong to the signal of 5- CF_3-2-PySF_3 (yield from **3r**, 8%).

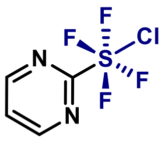

6l

Note. The sulfur(II) precursor was prepared from the reaction of N-chlorophthalimid with 2-Pyrimidinethiol according to the literature [10] because the attempt to synthesize the 2-pyrimidinyl zinc halide is failed.

In addition to the general method, the preparation of 2-pyrimidinyltetrafluoro-λ^6-sulfanyl chloride **6l** was also based on the procedure described below:

Method B: In a glovebox under Argon, the 2-pyrimidinyl-S-Phth precursor (**3s**, 0.1 mmol, 1 equiv.), trichloroisocyanuric acid (TCICA, 210 mg, 0.9 mmol, 9 equiv.) and rigorously dried KF (93 mg, 16 mmol, 16 equiv.) was added to an oven-dried 12 mL round-bottom reaction vial equipped with a stir bar. Under vigorous stirring, 1 mL dry and degassed MeCN was added to the mixture followed by addition of TFA solution (1 M in MeCN, 0.2 mL). Then the vial was sealed with a septum-pad cap and the reaction was stirred at the room temperature in the glovebox for 24 h. Upon the completion of reaction, the atmosphere in the vial was vented carefully and the internal standard α, α, α-trifluorotoluene was added into the mixture. After 10 min stirring, an aliquot of the resulting solution was filtered under argon. The NMR sample was prepared with 0.4 mL of the filtered aliquot and 0.1 mL CD_3CN for ^{19}F NMR yield determination.

Method B gave the comparable yield of desired product (61%) to the general method (65%), even with more TFA (3 eq. TFA led to the yield of 58%). Unlike other substrates which were transformed to $ArSOF_3$ in the presence of 2 eq. TFA, the loading of acid did not influence the yield of **6l** so significantly. In contrast, in order to obtain 2-pyrimidinylsulfur(VI) trifluoridoxide **4s**, a special method should be used with the assistance of $ZnCl_2$. The detailed method have been described in section 8.

$^{19}F\{^1H\}$ NMR (282 MHz, CH_3CN/CD_3CN) δ 117.8 (s, 4F).

^{19}F NMR spectra (via **Method B**)

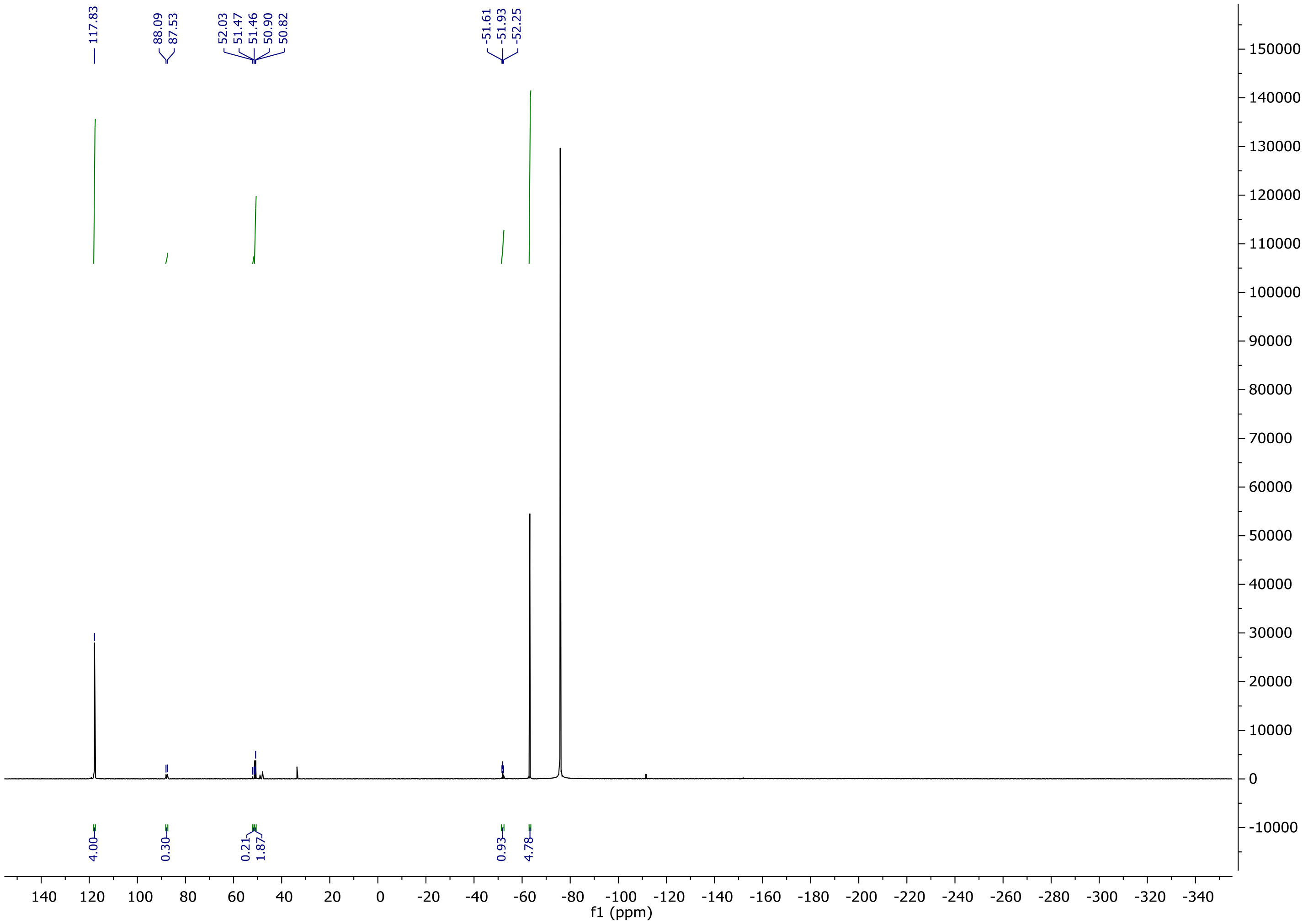

14. Study on the mechanism for the oxidation step toward $ArSOF_3$, $ArSO_2F$ and $ArSF_4Cl$

Based on our experimental results, when 2 equiv. of TFA are used as additive, Ar–SOF_3 is obtained in excellent yields; however, when 2 equiv. of AcOH, H_2O or alcohol are used instead, Ar–SO_2F becomes the main product (MeOH or ethanol lead to the best yield). On the other hand, the amount of TFA added has a profound effect on the outcome of the reaction: When 2 equiv are used, Ar–SOF_3 is obtained in very high yields; by contrast, when only 10 mol% TFA is used instead, the major product of the reaction becomes the Ar–SF_4Cl. In order to understand the mechanism of the reaction for selective formation of different arylsulfur(VI) fluorides, several supporting experiments were explored.

14.1 ^{19}F NMR monitor of the reaction for $ArSOF_3$

Firstly, the reaction for generation of 4-Br–C_6H_4-SOF_3 (**4d**) was monitored by ^{19}F NMR to search the key intermediates and to follow the changing trend of each components. As shown in Figure S8, the starting material **3d** was found to be almost completely converted to 4-Br–C_6H_4–SOF (intermediate **E**, at 6.36 ppm[19] in ^{19}F NMR spectrum) in 1 h. Afterwards, the amount of intermediate **E** decreased with the increasing yield of **4d**. When the reaction was completed, nearly all intermediate **E** was transformed to the desired product.

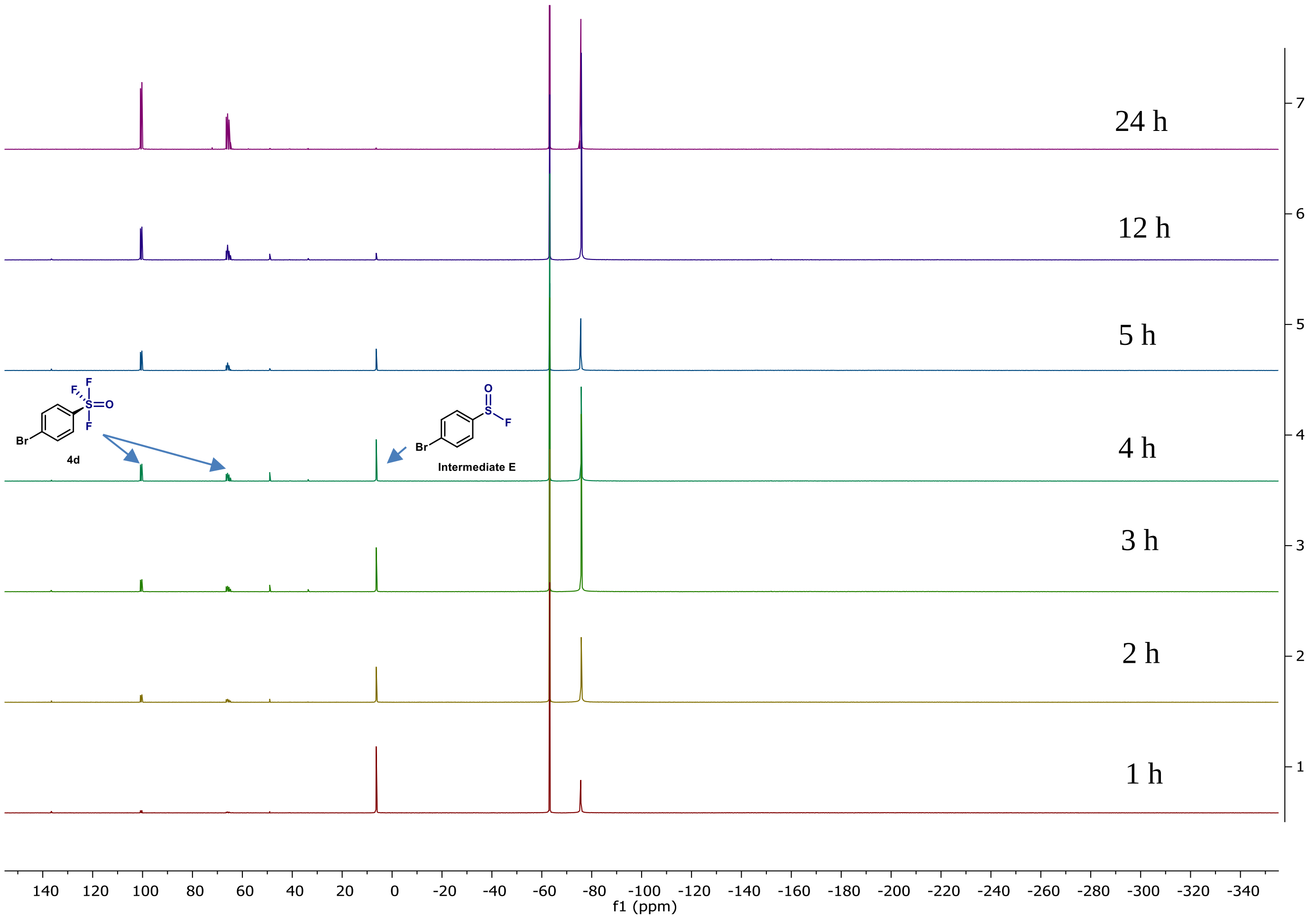

(A)

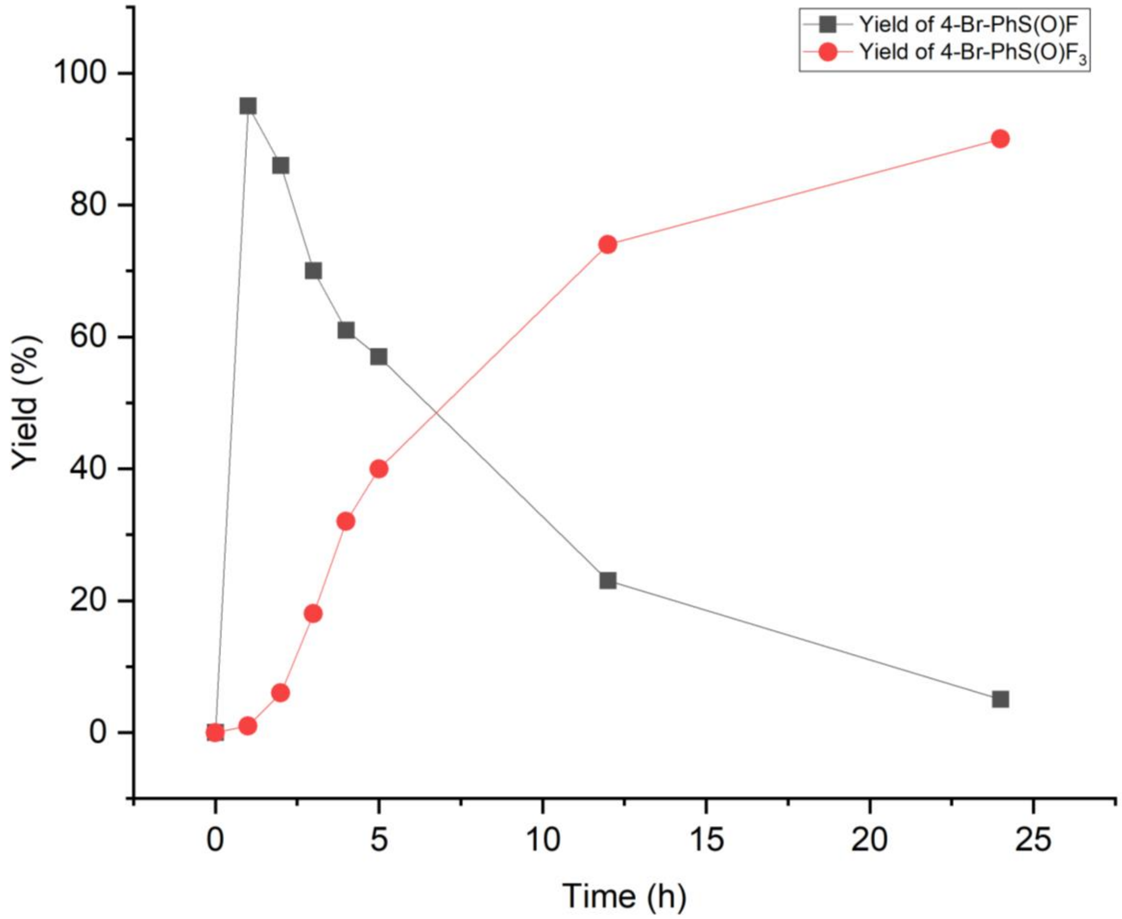

(B)

Figure S8. Monitor of the reaction for generation of **4d** by *in-situ* ^{19}F NMR, (A) ^{19}F NMR spectra; (B) changing trend of the yields of intermediate **E** and **4d**.

14.2 The reaction for the formation of $ArSO_2F$ in the presence of $H_2{}^{18}O$

Comparing the results with $MeOH/H_2O$ and TFA (2 equiv), it is reasonable to propose that the formation of Ar–SO_2F is the result of hydrolysis of the parent Ar–SOF_3. In order to understand where the O comes, labelling experiment with $H_2{}^{18}O$ have been performed. As a result, we observed 85% 4-Br–C_6H_4–$S^{18}O_2F$ and 13.7 % 4-Br–C_6H_4–$S^{18}O^{16}OF$ (Figure S9). This result implies that the most O of parent Ar–SOF_3 comes from the additive (in the presence of TFA, the best part of O comes from TFA) while a small amount of O of Ar–SOF_3 comes from other sources, such as glass.

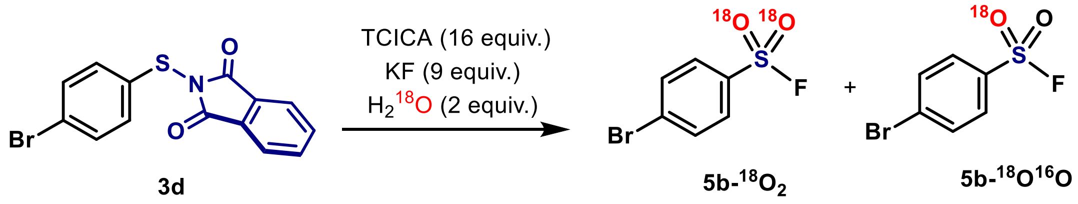

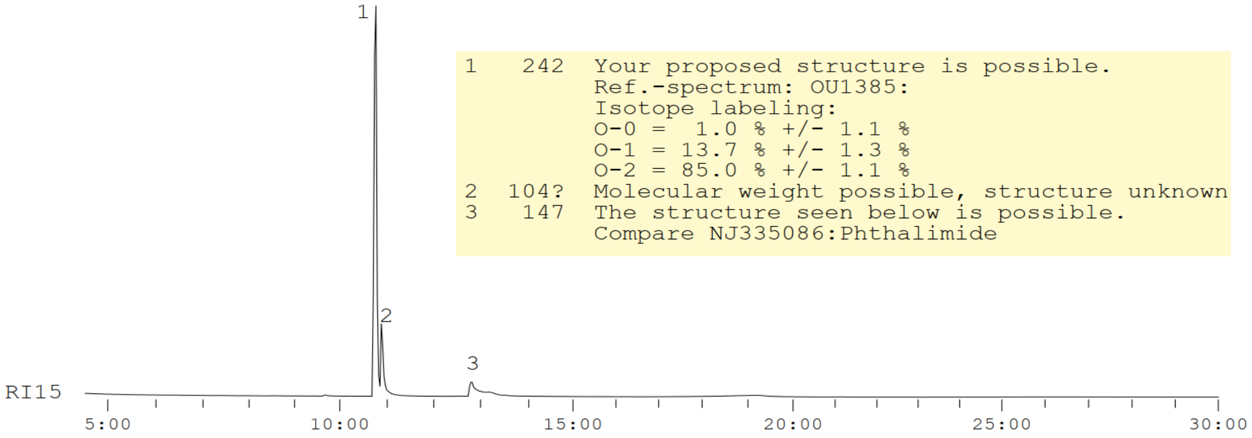

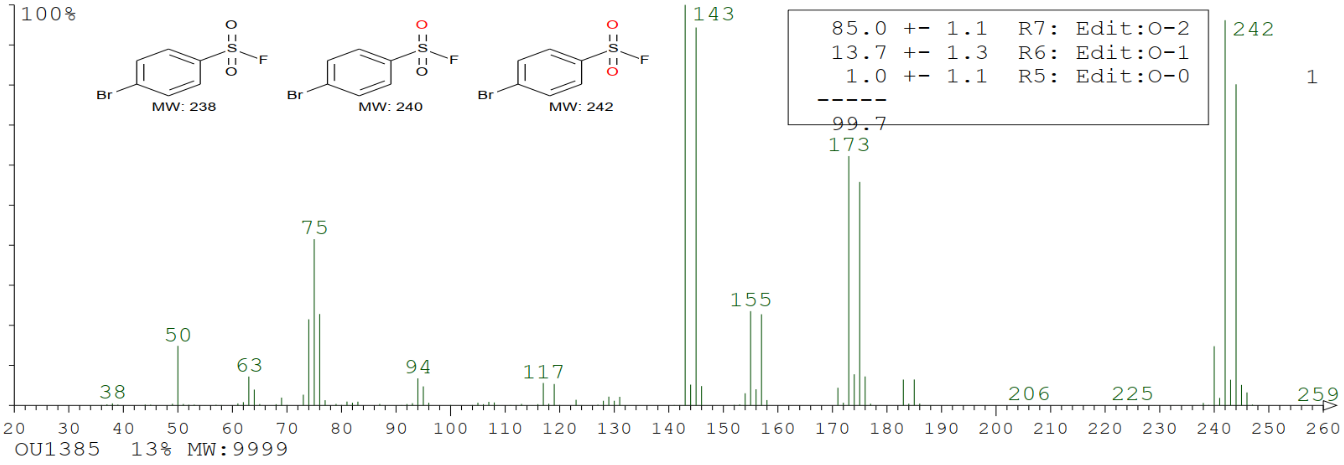

Figure S9. The reaction for 4-Br–C_6H_4–SO_2F in the presence of $H_2{}^{18}O$ and the HRMS of product.

14.3 ^{19}F NMR monitoring of the reaction for $ArSF_4Cl$

At last, the reaction for generation of 4-Br–C_6H_4–SF_4Cl (**6h**) was monitored by ^{19}F NMR (Figure S10). Unlike the result in section 14.1, the desired product **6h** was generated as the main product from the beginning. According to the literature from Togni[6], Ar–SF_3 was identified as the intermediate to Ar–SF_4Cl formation. Indeed, we also found the signal of 4-Br–C_6H_4–SF_3 (doublet at 57.7 ppm and triplet at -41.0 ppm, intermediate **G**) during the reaction. Similar to the result using diphenyl sulfide[6] as the starting material, 4-Br–C_6H_4-SOF_3 (**4d**) and 4-Br–C_6H_4–SF_3 (intermediate **G**) were found as the by-products after the reaction completing. Notably, in this case, the main effect of TFA is to activate the TCICA. However, it plays the role on formation of **4d** as well.

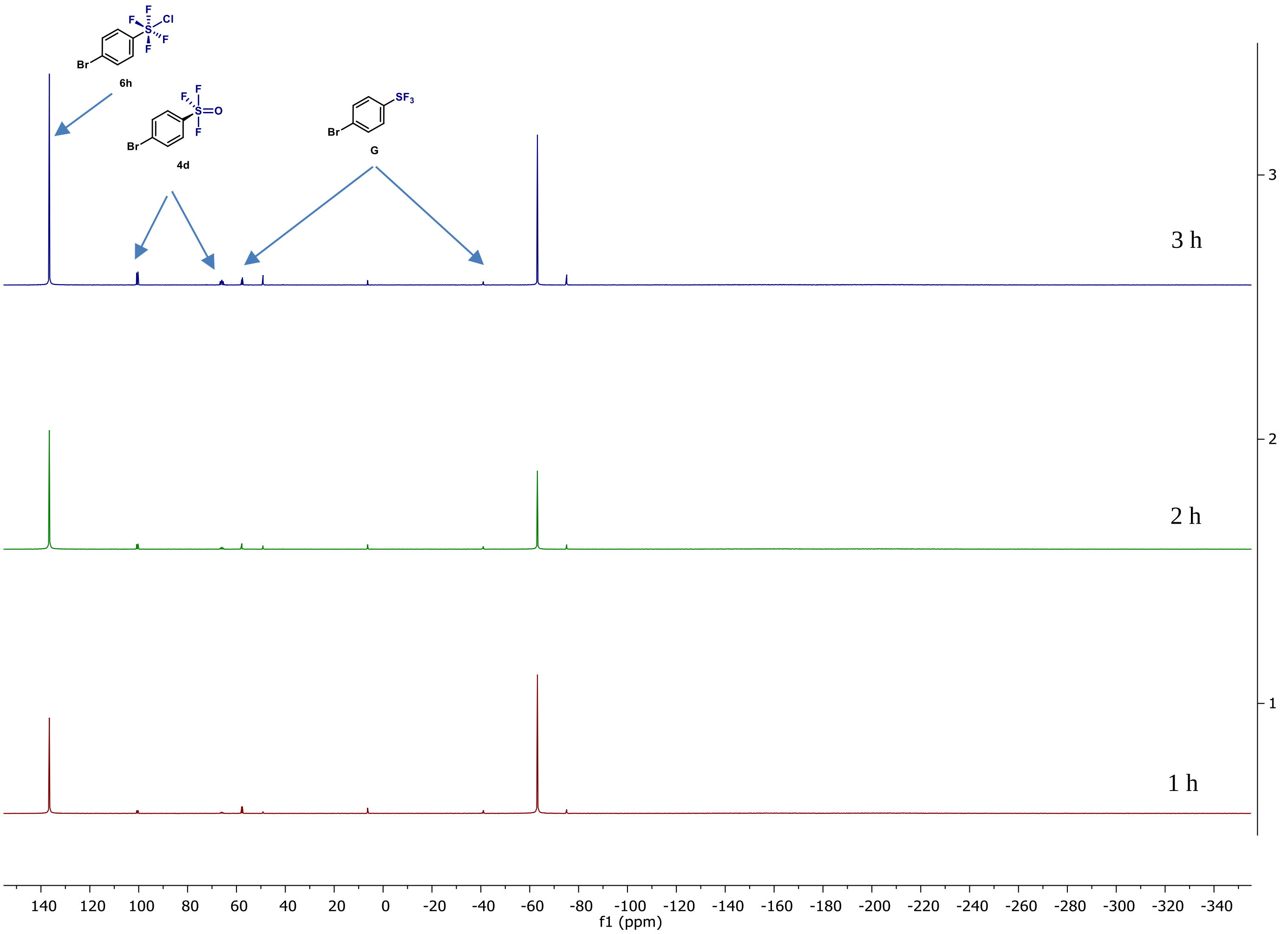

(A)

Time	Yield of **6h**	Yield of **4d**	Yield of **G**
1 h	32%	3%	7%
2 h	74%	9%	8%
3 h	80%	10%	9%

(B)

Figure S10. Monitoring of the reaction for the generation of **6h** by *in-situ* ^{19}F NMR, (A) ^{19}F NMR spectra; (B) changing trend of the yields of **6h**, **4d** and intermediate **G**.

14.4 Possible mechanism for the divergent formation of $ArSOF_3$, $ArSO_2F$ and $ArSF_4Cl$

Based on the above observations and literature, a possible mechanism for selective formation of different arylsulfur(VI) fluorides is proposed (Scheme S1).

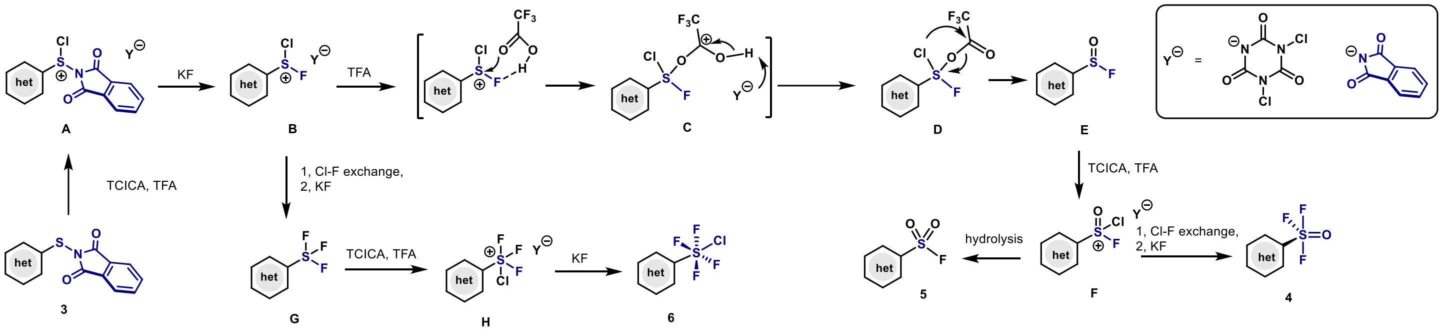

Scheme S1. A possible mechanism for selective formation of different arylsulfur(VI) fluorides.

The starting material **3** is firstly oxidized by activated TCICA to afford intermediate **A**, which undergoes F exchange with either Phth or Cl (Phth depicted in the Scheme), leading to intermediate **B**. In the presence of TFA, the sulfur center interacts with the O of additive. After electron transfer, the key intermediate **E** is formed. Afterwards, this intermediate is further oxidized to intermediate **F**. On one hand, with the Cl-F exchange and nucleophilic attack of KF, the Ar–SOF_3 (**4**) is obtained; On the other hand, after hydrolysis the reaction finally gives Ar–SO_2F (**5**) as the major product. However, if the loading of TFA is just 10 mol%, the intermediate B undergoes the Cl-F exchange step to generate intermediate **G** followed by second-step oxidation and nucleophilic attack of KF. As a result, the Ar–SF_4Cl (**6**) becomes the main product. It is important to mention that in the case of electron-donating groups in the Ar, intermediate F might not undergo Cl/F exchange leading to **4**, and formation of **5** is preferred.

15. General method on synthesis of ArS(O)(NR)F

The $ArSOF_3$ was synthesized according to the general method described in section 7. Upon the completion of reaction, the atmosphere in the vial was vented carefully and the suspension was transfored to a flame-dried Schlenk tube under argon. Then the solvent and other volatile constituent was evaporated under the vacuum. To the residue, dry and degassed Hexane/DCM (3:1) mixed solvent was added to extract the $ArSOF_3$ compound (4 mL × 3). The resulting solution was filtered under argon followed by the concentration of the filtrate under the vacuum. The crude product of $ArSOF_3$ was used immediately for the next step.

(Please note, the $ArSOF_3$ is extremely sensitive to the moisture and even glass. All reaction vials, flasks and NMR tubes used for the reaction and work-up process should be dried rigorously. Solvent should be ultra-dry (water content should be at least below 10 ppm). The reaction and work-up process should be handled always under argon and the work-up process should be handled in the above described order. For example, if the suspension upon the completion of reaction was filtered firstly and then the filtrate was concentrated under the vaccum, 37% to 70% $ArSOF_3$ will be destroyed and transformed to $ArSO_2F$.)

The crude product of $ArSOF_3$ (ca. 0.1 mmol, 1 equiv.) was dissolved in 1 mL dry and degassed MeCN followed by addition of Et_3N (0.3 mmol, 3 equiv., dry over 4Å molecular sieves) and amines (0.25 mmol, 2.5 equiv.) under argon. The mixture was stirred at room temperature for 18 h. Upon the completion of reaction, the resulting solution was then concentrated and further purified through a silica column chromatography (Pentane/EtOAc = 10:1).

Please note, although the desired product could be purified through silica column, ArS(O)(NR)F is still sensitive to the moisture. In the air, the product will be transformed slowly to corresponding sulfonamide [$ArS(O)_2$(NR)]. So the product should be restored under argon.

16. Scope for synthesis of ArS(O)(NR)F

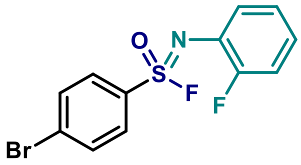

8a

Synthesis of 4-bromo-N-(2-fluorophenyl)benzenesulfonimidoyl fluoride (8a). The title compound was prepared from reaction of **4d** (0.1 mmol) and 2-fluorobenzeneamine **7a** according to the general method. After the reaction, the product was purified through a column chromatography (Pentane/EtOAc = 10:1). Pale yellow oil was obtained as the desired product (20 mg, 63% yield)

Rf (Pentane/EtOAc = 10:1): 0.56
^{1}H NMR (300 MHz, $CDCl_3$) δ 7.98 – 8.02 (m, 2H), 7.68 – 7.73 (m, 2H), 7.22 – 7.28 (m, 1H), 7.00 - 7.07 (m, 3H).
^{19}F{^{1}H} NMR (282 MHz, $CDCl_3$) δ 84.5 (d, *J* = 7.8 Hz, 1F), -123.1 (d, *J* = 7.8 Hz, 1F).
^{13}C NMR (75 MHz, $CDCl_3$) δ 161.38 (d, *J* = 265.5 Hz), 137.3, 132.9 (d, *J* = 27.0 Hz), 132.7, 131.2 (d, *J* = 3.7 Hz), 130.0 (d, *J* = 22.5 Hz), 129.4, 125.8 (d, *J* = 4.8 Hz), 125.6 (d, *J* = 7.2 Hz), 124.4 (d, *J* = 4.1 Hz).
HRMS (GC-EI): Calc'd for $C_{12}H_8BrF_2NOS^+$ $[M]^+$ 330.94727; found 330.94781

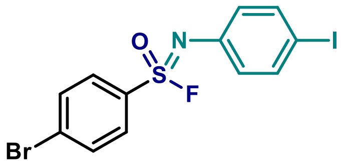

8b

Synthesis of 4-bromo-N-(4-iodophenyl)benzenesulfonimidoyl fluoride (8b). The title compound was prepared from reaction of **4d** (0.1 mmol) and 4-iodobenzeneamine **7b** according to the general method. After the reaction, the product was purified through a column chromatography (Pentane/EtOAc = 10:1). Pale yellow oil was obtained as the desired product (30 mg, 70% yield)

Rf (Pentane/EtOAc = 10:1): 0.56
^{1}H NMR (300 MHz, $CDCl_3$) δ 8.02 (dt, *J* = 8.9, 2.8 Hz, 2H), 7.74 – 7.79 (m, 2H), 7.60 – 7.65 (m, 2H), 6.98 - 7.03 (m, 2H).
^{19}F{^{1}H} NMR (282 MHz, $CDCl_3$) δ 82.9 (s, 1F).
^{13}C NMR (75 MHz, $CDCl_3$) δ 139.0 (d, *J* = 6.0 Hz), 138.3, 134.1 (d, *J* = 26.6 Hz), 133.1, 132.7, 130.2, 129.3, 125.9 (d, *J* = 4.4 Hz).
HRMS (GC-EI): Calc'd for $C_{12}H_8BrFINOS^+$ $[M]^+$ 438.85334; found 438.85402.

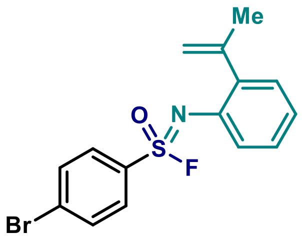

8c

Synthesis of 4-bromo-N-(2-(prop-1-en-2-yl)phenyl)benzenesulfonimidoyl fluoride (8c). The title compound was prepared from reaction of **4d** (0.1 mmol) and 2-(1-methylethenyl)-benzeneamine **7c** according to the general method. After the reaction, the product was purified through a column chromatography (Pentane/EtOAc = 10:1). Colorless oil was obtained as the desired product (24 mg, 68% yield).

Rf (Pentane/EtOAc = 10:1): 0.54

^{1}H NMR (300 MHz, $CDCl_3$) δ 8.01 (dt, *J* = 8.9, 2.8 Hz, 2H), 7.73 – 7.78 (m, 2H), 7.36 (dd, *J* = 8.3, 1.3 Hz, 1H), 7.21 - 7.26 (m, 2H), 7.11 (tdd, *J* = 8.9, 8.3, 1.3 Hz, 1H), 5.16 – 5.19 (m, 1H), 5.00 – 5.01 (m, 1H), 2.10 (dd, *J* = 1.5, 0.9 Hz, 3H).

^{19}F{^{1}H} NMR (282 MHz, $CDCl_3$) δ 84.8 (s, 1F).

^{13}C NMR (75 MHz, $CDCl_3$) δ 144.5, 139.0 (t, *J* = 5.0 Hz), 135.8 (d, *J* = 6.0 Hz), 134.8 (d, *J* = 27.8 Hz), 132.6, 129.8, 129.4, 129.3, 127.9, 124.6 (d, *J* = 1.1 Hz), 123.8 (d, *J* = 4.0 Hz), 115.3, 23.6.

HRMS (ESI): Calc'd for $C_{15}H_{13}BrFNOSNa^+$ $[M+Na]^+$ 375.97776; found 375.97771.

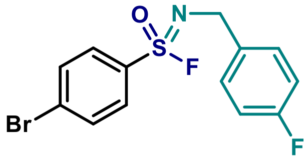

8d

Synthesis of 4-bromo-N-(4-fluorobenzyl)benzenesulfonimidoyl fluoride (8d). The title compound was prepared from reaction of **4d** (0.1 mmol) and 4-fluorobenzylamine **7d** according to the general method. After the reaction, the product was purified through a column chromatography (Pentane/EtOAc = 10:1). Colorless oil was obtained as the desired product (27 mg, 80% yield).

Rf (Pentane/EtOAc = 10:1): 0.56

^{1}H NMR (300 MHz, $CDCl_3$) δ 7.87 (dt, *J* = 8.9, 2.8 Hz, 2H), 7.61 – 7.65 (m, 2H), 7.26 – 7.31 (m, 2H), 6.96 (tt, *J* = 8.9, 2.8 Hz, 2H), 4.46 – 4.60 (m, 2H).

^{19}F{^{1}H} NMR (282 MHz, $CDCl_3$) δ 86.8 (s, 1F), -115.5 (s, 1F).

^{13}C NMR (75 MHz, $CDCl_3$) δ 162.0 (d, *J* = 245.1 Hz), 134.9 (d, *J* = 28.6 Hz), 132.5, 129.5, 129.1, 129.1, 129.0, 115.3 (d, *J* = 21.5 Hz), 45.8 (d, *J* = 3.6 Hz).

HRMS (GC-EI): Calc'd for $C_{13}H_{10}BrF_2NOS^+$ $[M]^+$ 344.96292; found 344.96295.

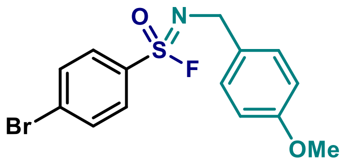

8e

Synthesis of 4-bromo-N-(4-methoxybenzyl)benzenesulfonimidoyl fluoride (8e). The title compound was prepared from reaction of **4d** (0.1 mmol) and 4-methoxybenzylamine **7e** according to the general method. After the reaction, the product was purified through a column chromatography (Pentane/EtOAc = 10:1). Colorless oil was obtained as the desired product (28 mg, 78% yield). NMR data are in accordance with the literature report. [27]

^{1}H NMR (300 MHz, $CDCl_3$) δ 7.86 (dt, *J* = 8.9, 2.8 Hz, 2H), 7.59 – 7.64 (m, 2H), 7.22 – 7.27 (m, 2H), 6.81 (dt, *J* = 8.9, 2.8 Hz, 2H), 4.44 – 4.57 (m, 2H), 3.73 (s, 3H).

^{19}F{^{1}H} NMR (282 MHz, $CDCl_3$) δ 87.0 (s, 1F).

^{13}C NMR (75 MHz, $CDCl_3$) δ 158.8, 135.0 (d, *J* = 28.9 Hz), 132.4, 131.2 (d, *J* = 5.0 Hz), 129.3, 129.1, 128.7, 113.9, 55.3, 46.0 (d, *J* = 3.5 Hz).

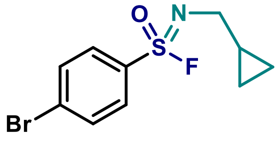

8f

Synthesis of 4-bromo-N-(cyclopropylmethy)benzenesulfonimidoyl fluoride (8f). The title compound was prepared from reaction of **4d** (0.1 mmol) and (cyclopropylmethyl)amine **7f** according to the general method. After the reaction, the product was purified through a column chromatography (Pentane/EtOAc = 10:1). Colorless oil was obtained as the desired product (23 mg, 81% yield).

Rf (Pentane/EtOAc = 10:1): 0.66

^{1}H NMR (300 MHz, $CDCl_3$) δ 7.92 (dt, *J* = 8.9, 2.8 Hz, 2H), 7.67 – 7.71 (m, 2H), 3.25 – 3.43 (m, 2H), 1.10 – 1.20 (m, 1H), 0.53 – 0.59 (m, 2H), 0.28 – 0.33 (m, 2H).

^{19}F{^{1}H} NMR (282 MHz, $CDCl_3$) δ 88.0 (s, 1F).

^{13}C NMR (75 MHz, $CDCl_3$) δ 135.4 (d, *J* = 29.5 Hz), 132.3, 129.1, 129.0, 48.4 (d, *J* = 3.8 Hz), 12.8 (d, *J* = 5.3 Hz), 3.84 (d, *J* = 15.1 Hz).

HRMS (ESI): Calc'd for $C_{10}H_{12}BrFNOS^+$ $[M+H]^+$ 291.98016; found 291.97998.

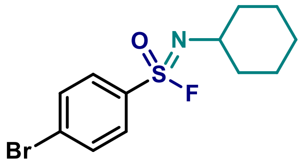

8g

Synthesis of 4-bromo-N-(cyclohexyl)benzenesulfonimidoyl fluoride (8g). The title compound was prepared from reaction of **4d** (0.1 mmol) and cyclohexanamine **7g** according to the general method. After the reaction, the product was purified through a column chromatography (Pentane/EtOAc = 10:1). Colorless oil was obtained as the desired product (24 mg, 76% yield).

Rf (Pentane/EtOAc = 10:1): 0.60

^{1}H NMR (300 MHz, $CDCl_3$) δ 7.90 (dt, J = 8.9, 2.8 Hz, 2H), 7.65 – 7.70 (m, 2H), 3.71 – 3.82 (m, 1H), 1.91 – 2.00 (m, 2H), 1.74 – 1.81 (m, 2H), 1.46 – 1.60 (m, 2H), 1.22 – 1.44 (m, 4H).

^{19}F{^{1}H} NMR (282 MHz, $CDCl_3$) δ 91.0 (s, 1F).

^{13}C NMR (75 MHz, $CDCl_3$) δ 135.8 (d, J = 29.7 Hz), 132.3, 129.1, 128.9, 54.1 (d, J = 4.0 Hz), 35.6 (d, J = 2.9 Hz), 35.4 (d, J = 5.9 Hz), 25.5, 24.6 (2C).

HRMS (ESI): Calc'd for $C_{12}H_{16}BrFNOS^+$ $[M+H]^+$ 320.01146; found 320.01113.

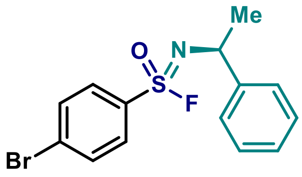

8h

Synthesis of 4-bromo-N-((R)-1-phenylethyl)benzenesulfonimidoyl fluoride (8h). The title compound was prepared from reaction of **4d** (0.1 mmol) and (R)-1-phenylethylamine **7h** according to the general method. After the reaction, the product was purified through a column chromatography (Pentane/EtOAc = 10:1). Colorless oil was obtained as the desired product (22 mg, 64% yield, d.r. = 1.0:1.1).

Rf (Pentane/EtOAc = 10:1): 0.54

^{1}H NMR (300 MHz, $CDCl_3$) δ 7.97 (dt, J = 8.9, 2.8 Hz, 2H), 7.72 – 7.75 (m, 0.53 × 2H, diastereomer A), 7.68 – 7.72 (m, 0.47 × 2H, diastereomer B), 7.25 – 7.48 (m, 5H), 5.06 – 5.18 (m, 1H), 1.68 (d, J = 6.7 Hz, 0.53 × 3H, diastereomer A), 1.63 (d, J = 6.7 Hz, 0.47 × 3H, diastereomer B).

^{19}F{^{1}H} NMR (282 MHz, $CDCl_3$) δ 90.6 (s, 0.47 × 1F, diastereomer B), 86.6 (s, 0.53 × 1F, diastereomer A).

^{13}C NMR (75 MHz, $CDCl_3$) δ 145.1 (d, J = 3.9 Hz, diastereomer A), 145.0 (d, J = 3.9 Hz, diastereomer B), 135.7 (d, J = 29.8 Hz), 132.7, 129.43 (diastereomer A), 129.38 (diastereomer B), 128.78 (diastereomer A), 128.74 (diastereomer B), 127.49 (diastereomer A), 127.41 (diastereomer B), 126.29 (diastereomer A), 126.24

(diastereomer B), 54.4 (d, J = 3.3 Hz, diastereomer A), 54.2 (d, J = 4.2 Hz, diastereomer B), 26.7 (d, J = 3.8 Hz, diastereomer B), 26.6 (d, J = 7.5 Hz, diastereomer A).

HRMS (ESI): Calc'd for $C_{14}H_{13}BrFNOSNa^+$ $[M+Na]^+$ 363.97776; found 363.97751.

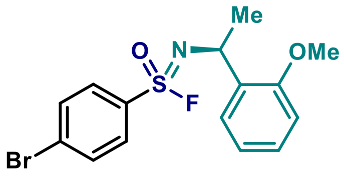

8i

Synthesis of 4-bromo-N-((R)-1-(2-methoxyphenyl)ethyl)benzenesulfonimidoyl fluoride (8i). The title compound was prepared from reaction of **4d** (0.1 mmol) and 1-(2-methoxyphenyl)ethylamine **7i** according to the general method. After the reaction, the product was purified through a column chromatography (Pentane/EtOAc = 10:1). Colorless oil was obtained as the desired product (22 mg, 64% yield, d.r. = 1.0:1.07).

Rf (Pentane/EtOAc = 10:1): 0.52

^{1}H NMR (300 MHz, $CDCl_3$) δ 7.92 – 7.97 (m, 2H), 7.67 – 7.71 (m, 2H), 7.53 – 7.57 (m, 1H), 7.20 – 7.27 (m, 1H), 6.93 – 7.00 (m, 1H), 6.88 (dd, J = 4.5, 1.1 Hz, 0.52 × 2H, diastereomer A), 6.85 (dd, J = 4.5, 1.1 Hz, 0.48 × 2H, diastereomer B), 5.45 – 5.54 (m, 1H), 3.87 (s, 0.52 × 3H, diastereomer A), 3.87 (s, 0.48 × 3H, diastereomer B), 1.58 (d, J = 6.7 Hz, 0.52 × 3H, diastereomer A), 1.54 (d, J = 6.7 Hz, 0.48 × 3H, diastereomer B).

^{19}F{^{1}H} NMR (282 MHz, $CDCl_3$) δ 90.9 (s, 0.48 × 1F, diastereomer B), 86.3 (s, 0.52 × 1F, diastereomer A).

^{13}C NMR (75 MHz, $CDCl_3$) δ 155.7, 139.2 (d, J = 25.1 Hz), 132.3, 129.11 (diastereomer A), 129.05 (diastereomer B), 128.4, 127.99 (diastereomer A), 127.89 (diastereomer B), 126.39 (diastereomer A), 126.29 (diastereomer B), 120.6, 110.33 (diastereomer A), 110.31 (diastereomer B), 55.32 (diastereomer A), 55.31 (diastereomer B), 48.26 (d, J = 4.3 Hz, diastereomer B), 47.93 (d, J = 3.3 Hz, diastereomer A), 25.17 (d, J = 2.9 Hz, diastereomer A), 24.84 (d, J = 6.8 Hz, diastereomer B).

HRMS (ESI): Calc'd for $C_{15}H_{15}BrFNO_2SNa^+$ $[M+Na]^+$ 393.98832; found 393.98816.

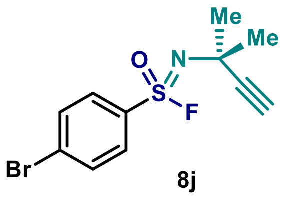

8j

Synthesis of 4-bromo-N-(1,1-dimethyl-2-propynyl)benzenesulfonimidoyl fluoride (8j). The title compound was prepared from reaction of **4d** (0.1 mmol) and 1,1-dimethyl-2-propynylamine **7j** according to the general

method. After the reaction, the product was purified through a column chromatography (Pentane/EtOAc = 10:1). Colorless oil was obtained as the desired product (20 mg, 68% yield).

Rf (Pentane/EtOAc = 10:1): 0.58

^{1}H NMR (300 MHz, $CDCl_3$) δ 7.86 – 7.92 (m, 2H), 7.65 – 7.70 (m, 2H), 2.46 (s, 1H), 1.73 (s, 3H), 1.72 (s, 3H).

^{19}F{^{1}H} NMR (282 MHz, $CDCl_3$) δ 88.0 (s, 1F).

^{13}C NMR (75 MHz, $CDCl_3$) δ 134.9 (d, *J* = 27.8 Hz), 131.3, 128.1, 128.0, 86.8 (d, *J* = 5.4 Hz), 68.6, 50.7 (d, *J* = 3.2 Hz), 31.7 (d, *J* = 4.2 Hz), 31.8 (d, *J* = 2.7 Hz).

HRMS (ESI): Calc'd for $C_{11}H_{11}BrFNOSNa+$ $[M+Na]^+$ 325.96211; found 325.96249.

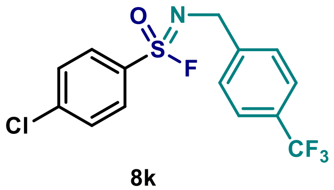

8k

Synthesis of 4-chloro-N-(4-(trifluoromethyl)benzyl)benzenesulfonimidoyl fluoride (8k). The title compound was prepared from reaction of **4c** (0.1 mmol) and 4-(trifluoromethyl)benzylamine **7k** according to the general method. After the reaction, the product was purified through a column chromatography (Pentane/EtOAc = 10:1). Colorless oil was obtained as the desired product (26 mg, 73% yield).

Rf (Pentane/EtOAc = 10:1): 0.56

^{1}H NMR (300 MHz, $CDCl_3$) δ 8.03 (dt, *J* = 8.9, 2.8 Hz, 2H), 7.59 – 7.63 (m, 2H), 7.56 – 7.58 (m, 1H), 7.49 – 7.55 (m, 3H), 4.62 – 4.77 (m, 2H).

^{19}F{^{1}H} NMR (282 MHz, $CDCl_3$) δ 86.6 (s, 1F), -62.5 (s, 3F).

^{13}C NMR (75 MHz, $CDCl_3$) δ 142.9, 141.1, 134.1 (d, *J* = 28.4 Hz), 130.0 (d, *J* = 18.6 Hz), 129.6, 129.0, 127.6, 125.4 (q, *J* = 3.8 Hz), 124.1 (d, *J* = 270.8 Hz), 46.0 (d, *J* = 3.6 Hz).

HRMS (GC-EI): Calc'd for $C_{14}H_{11}ClF_4NOS^+$ $[M+H]^+$ 352.01805; found 352.01818.

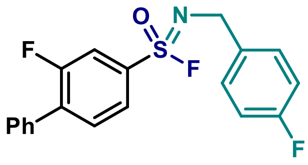

8l

Synthesis of N-(4-fluorobenzyl)(2-fluoro[1,1'-biphenyl]-4-yl)sulfonimidoyl fluoride (8l). The title compound was prepared from reaction of **4m** (0.1 mmol) and 4-fluorobenzylamine **7d** according to the general method. After

the reaction, the product was purified through a column chromatography (Pentane/EtOAc = 10:1). Colorless oil was obtained as the desired product (28 mg, 77% yield).

Rf (Pentane/EtOAc = 10:1): 0.55

^{1}H NMR (300 MHz, $CDCl_3$) δ 7.86 – 7.95 (m, 2H), 7.63 (t, *J* = 7.8 Hz, 1H), 7.44 – 7.60 (m, 5H), 7.36 – 7.41 (m, 2H), 7.01 – 7.09 (m, 2H), 4.56 – 4.70 (m, 2H).

^{19}F{^{1}H} NMR (282 MHz, $CDCl_3$) δ 87.0 (s, 1F), -113.7 (s, 1F), -115.5 (s, 1F).

^{13}C NMR (75 MHz, $CDCl_3$) δ 162.2 (d, *J* = 225.0 Hz), 158.9 (d, *J* = 232.6 Hz), 135.8 (d, *J* = 29.1 Hz), 135.4 (d, *J* = 13.6 Hz), 134.8 (d, *J* = 3.1 Hz), 134.7 (d, *J* = 2.9 Hz), 133.6 (d, *J* = 1.2 Hz), 131.5 (d, *J* = 3.7 Hz), 129.1 (d, *J* = 3.9 Hz), 129.0 (d, *J* = 3.0 Hz), 128.8, 123.5 (d, *J* = 4.0 Hz), 116.0 (d, *J* = 27.8 Hz), 115.3 (d, *J* = 21.5 Hz), 45.9 (d, *J* = 3.5 Hz).

HRMS (EI): Calc'd for $C_{19}H_{14}F_3NOS^+$ $[M]^+$ 361.07482; found 361.07543.

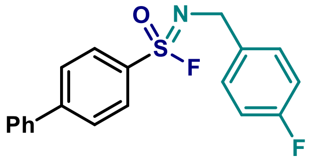

8m

Synthesis of N-(4-fluorobenzyl)([1,1'-biphenyl]-4-yl)sulfonimidoyl fluoride (8m). The title compound was prepared from reaction of **4n** (0.1 mmol) and 4-fluorobenzylamine **7d** according to the general method. After the reaction, the product was purified through a column chromatography (Pentane/EtOAc = 10:1). Colorless oil was obtained as the desired product (24 mg, 70% yield).

Rf (Pentane/EtOAc = 10:1): 0.53

^{1}H NMR (300 MHz, $CDCl_3$) δ 8.14 (d, *J* = 8.5 Hz, 2H), 7.75 (d, *J* = 8.5 Hz, 2H), 7.57 – 7.65 (m, 2H), 7.35 – 7.53 (m, 5H), 7.04 (t, *J* = 8.5 Hz, 2H), 4.57 – 4.71 (m, 2H).

^{19}F{^{1}H} NMR (282 MHz, $CDCl_3$) δ 85.8 (s, 1F), -115.8 (s, 1F).

^{13}C NMR (75 MHz, $CDCl_3$) δ 162.0 (d, *J* = 245.0 Hz), 147.2, 138.9, 134.7 (d, *J* = 34.7 Hz), 129.1, 129.0, 128.8, 128.1, 127.7, 127.4, 123.0 (d, *J* = 12.0 Hz), 115.3 (d, *J* = 21.4 Hz), 45.8 (d, *J* = 3.3 Hz).

HRMS (GC-EI): Calc'd for $C_{19}H_{15}F_2NOS^+$ $[M]^+$ 343.08369; found 343.08397.

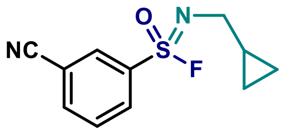

8n

Synthesis of N-(cyclopropylmethy)-3-nitrilebenzenesulfonimidoyl fluoride (8n). The title compound was prepared from reaction of **4k** (0.1 mmol) and (cyclopropylmethyl)amine **7f** according to the general method. After the reaction, the product was purified through a column chromatography (Pentane/EtOAc = 10:1). White solide was obtained as the desired product (16 mg, 67% yield).

Rf (Pentane/EtOAc = 5:1): 0.49

^{1}H NMR (300 MHz, $CDCl_3$) δ 8.37 (s, 1H), 8.29 (d, *J* = 8.8 Hz, 1H), 7.94 (d, *J* = 7.8 Hz, 1H), 7.71 (t, *J* = 7.8 Hz, 1H), 3.27 – 3.45 (m, 2H), 1.17 (hept, *J* = 8.1 Hz, 1H), 0.59 (d, *J* = 8.1 Hz, 2H), 0.32 (d, *J* = 4.7 Hz, 2H).

^{19}F{^{1}H} NMR (282 MHz, $CDCl_3$) δ 89.6 (s, 1F).

^{13}C NMR (75 MHz, $CDCl_3$) δ 138.0 (d, *J* = 31.6 Hz), 136.8, 133.8, 131.4, 130.1, 123.2, 116.7, 113.8, 48.6 (d, *J* = 3.9 Hz), 12.8 (d, *J* = 5.3 Hz), 4.0 (d, *J* = 14.5 Hz).

HRMS (GC-EI): Calc'd for $C_{11}H_{12}FN_2OS^+$ $[M+H]^+$ 239.06489; found 23.906476.

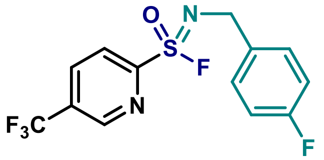

8o

Synthesis of N-(4-fluorobenzyl)-3-(trifluoromethyl)pyridinylsulfonimidoyl fluoride (8o). The title compound was prepared from reaction of **4r** (0.1 mmol) and 4-fluorobenzylamine **7d** according to the general method. After the reaction, the product was purified through a column chromatography (Pentane/EtOAc = 10:1). Colorless oil was obtained as the desired product (16 mg, 47% yield).

Rf (Pentane/EtOAc = 10:1): 0.56

^{1}H NMR (300 MHz, $CDCl_3$) δ 8.96 (s, 1H), 8.25 (d, *J* = 8.3 Hz, 1H), 8.15 (dd, *J* = 8.3, 1.7 Hz, 1H), 7.28 – 7.32 (m, 2H), 6.94 – 7.00 (m, 2H), 4.57 – 4.67 (m, 2H).

^{19}F{^{1}H} NMR (282 MHz, $CDCl_3$) δ 79.2 (s, 1F), -62.7 (s, 3F), -115.2 (s, 1F).

^{13}C NMR (75 MHz, $CDCl_3$) δ 161.1 (d, *J* = 245.7 Hz), 147.4 (d, *J* = 214.1 Hz), 138.2 (d, *J* = 29.2 Hz), 134.9 (d, *J* = 7.0 Hz), 134.1, 133.7, 133.2 (d, *J* = 7.4 Hz), 128.1 (d, *J* = 8.1 Hz), 122.3, 114.4 (d, *J* = 21.6 Hz), 45.3 (d, *J* = 3.8 Hz).

HRMS (ESI): Calc'd for $C_{13}H_9F_5N_2OSNa^+$ $[M+Na]^+$ 359.02480; found 359.02504.

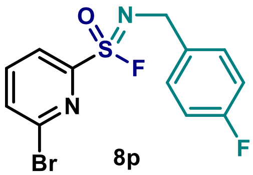

8p

Synthesis of 6-bromo-N-(4-fluorobenzyl)-pyridinylsulfonimidoyl fluoride (8o). The title compound was prepared from reaction of **4p** (0.1 mmol) and 4-fluorobenzylamine **7d** according to the general method. After the reaction, the product was purified through a column chromatography (Pentane/EtOAc = 10:1). Pale yellow oil was obtained as the desired product (14 mg, 41% yield).

Rf (Pentane/EtOAc = 10:1): 0.56

^{1}H NMR (300 MHz, $CDCl_3$) δ 8.04 (dd, *J* = 6.2, 2.3 Hz, 1H), 7.67 – 7.76 (m, 2H), 7.30 (dd, *J* = 8.4, 5.4 Hz, 2H), 8.15 (t, *J* = 8.7 Hz, 2H), 4.53 – 4.66 (m, 2H).

^{19}F{^{1}H} NMR (282 MHz, $CDCl_3$) δ 77.4 (s, 1F), -115.4 (s, 1F).

^{13}C NMR (75 MHz, $CDCl_3$) δ 163.7 (d, *J* = 277.9 Hz), 152.5 (d, *J* = 52.5 Hz), 141.4, 139.0, 137.8 (d, *J* = 24.0 Hz), 132.0, 128.1 (d, *J* = 8.2 Hz), 121.2, 114.3 (d, *J* = 21.4 Hz), 45.3 (d, *J* = 3.8 Hz).

HRMS (ESI): Calc'd for $C_{12}H_9BrF_2N_2OSNa^+$ $[M+Na]^+$ 368.94792; found 368.94781.

17. References

[1] E. Carbonnel, T. Besset, T. Poisson, D. Labar, X. Pannecoucke, P. Jubault, *Chem. Commun.* **2017**, *53*, 5706-5709.

[2] J. Li, W. Rao, S. Y. Wang, S. J. Ji, *J. Org. Chem.* **2019**, *84*, 11542-11552.

[3] Y. Zheng, F. L. Qing, Y. Huang, X. H. Xu, *Adv. Synth. Catal.* **2016**, *358*, 3477-3481.

[4] Y. Huang, X. Li, X. Wang, Y. Yu, J. Zheng, W. Wu, H. Jiang, *Chem. Sci.* **2017**, *8*, 7047-7051.

[5] J. T. Reeves, K. Camara, Z. S. Han, Y. Xu, H. Lee, C. A. Busacca, C. H. Senanayake, *Org. Lett.* **2014**, *16*, 1196-1199.

[6] C. R. Pitts, D. Bornemann, P. Liebing, N. Santschi, A. Togni, *Angew. Chem. Int. Ed.* **2019**, *58*, 1950-1954.

[7] S.-J. Zhu, H.-Z. Ying, Y. Wu, N. Qiu, T. Liu, B. Yang, X.-W. Dong, Y.-Z. Hu, *RSC Adv.* **2015**, *5*, 103172-103183.

[8] W. C. Gao, Y. Z. Shang, H. H. Chang, X. Li, W. L. Wei, X. Z. Yu, R. Zhou, *Org. Lett.* **2019**, *21*, 6021-6024.

[9] J. X. Zou, J. H. Chen, T. Shi, Y. S. Hou, F. Cao, Y. Q. Wang, X. D. Wang, Z. Jia, Q. Y. Zhao, Z. Wang, *ACS Catal.* **2019**, *9*, 11426-11430.

[10] S. Graßl, C. Hamze, T. J. Koller, P. Knochel, *Chem. Eur. J.,* **2019**, *25*, 3752-3755.

[11] Y. Chen, M. Tang, Y. Wu, X. Su, X. Li, S. Xu, S. Zhuo, J. Ma, D. Yuan, C. Wang, *Angew. Chem. Int. Ed.* **2019**, *58*, 14731-14739.

[12] H. M. Gillis, L. Greene, A. J. S. Thompson, *Synlett.* **2009**, *2009*, 112-116.

[13] Z. Song, Y. Wu, T. Xin, C. Jin, X. Wen, H. Sun, Q.-L. Xu, *Chem. Commun.* **2016**, *52*, 6079-6082.

[14] S. E. Denmark, E. Hartmann, D. J. Kornfilt, H. Wang, *Nat. Chem.* **2014**, *6*, 1056 - 1064.

[15] M. Tudge, M. Tamiya, C. Savarin, G. R. Humphrey, *Org. Lett.* **2006**, *8*, 565-568.

[16] E. W. Garbisch Jr, *J. Chem. Ed.* **1968**, *45*, 402 - 416.

[17] R. Braun, A. Cowley, M. Cushner, R. Lagow, *Inorg. Chem.* **1978**, *17*, 1679-1681.

[18] R. A. Stewart, S. Fujiwara, F. Aubke, *J. Chem. Phys.* **1968**, *49*, 965-966.

[19] I. Ruppert, *Chem. Ber.* **1980**, *113*, 1047-1052.

[20] Y. Liu, D. Yu, Y. Guo, J. C. Xiao, Q. Y. Chen, C. Liu, *Org. Lett.* **2020**, *22*, 2281-2286.

[21] M. Perez-Palau, J. Cornella, *Eur. J. Org. Chem.* **2020**, *2020*, 2497-2500.

[22] T. Zhong, M. K. Pang, Z. D. Chen, B. Zhang, J. Weng, G. Lu, *Org Lett* **2020**, *22*, 3072-3078.

[23] A. Haas, M. Spitzer, M. Lieb, *Chem. Ber.* **1988**, *121*, 1329-1340.

[24] G. Laudadio, A. d. A. Bartolomeu, L. M. Verwijlen, Y. Cao, K. T. de Oliveira, T. Noël, *J. Am. Chem. Soc.* **2019**, *141*, 11832-11836.

[25] B. Cui, S. Jia, E. Tokunaga, N. Saito, N. Shibata, *Chem. Commun.* **2017**, *53*, 12738-12741.

[26] P. Das, M. Takada, K. Matsuzaki, N. Saito, N. Shibata, *Chem. Commun.* **2017**, *53*, 3850-3853.

[27] B. Gao, S. Li, P. Wu, J. E. Moses, K. B. Sharpless, *Angew. Chem. Int. Ed.* **2018**, *57*, 1939-1943.

18. NMR Spectra

¹H Spectra (300 MHz, $CDCl_3$)

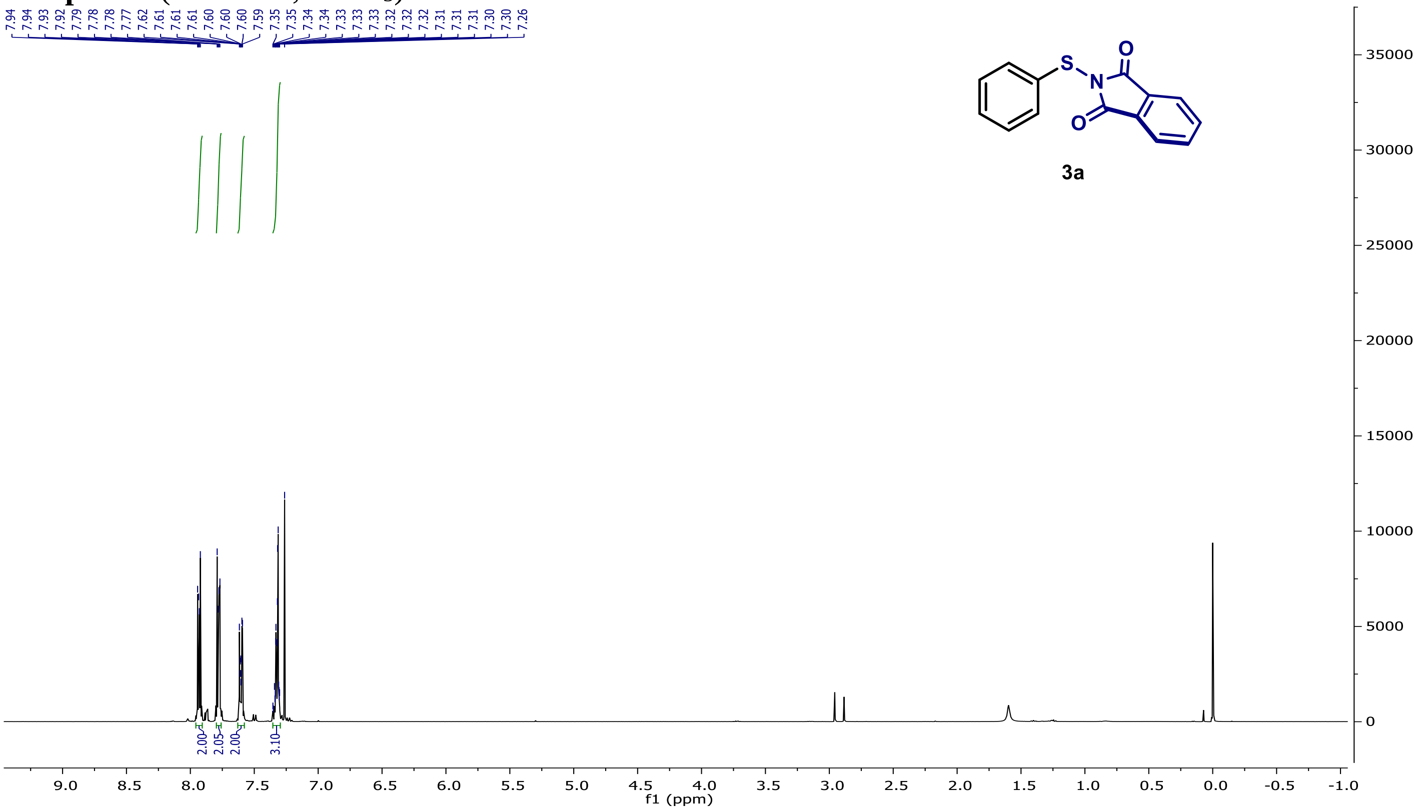

¹³C Spectra (75 MHz, $CDCl_3$)

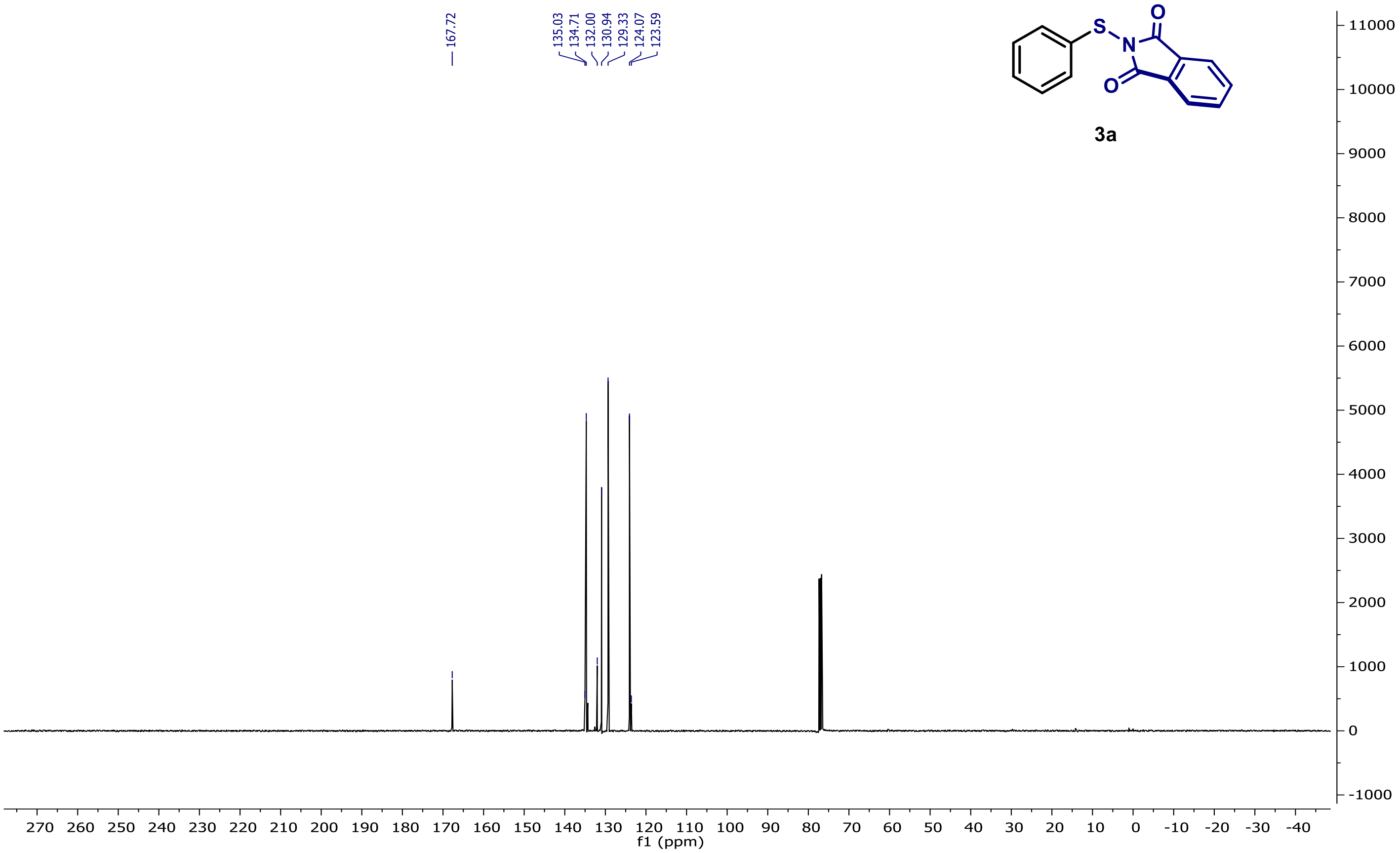

^{1}H Spectra (300 MHz, $CDCl_3$)

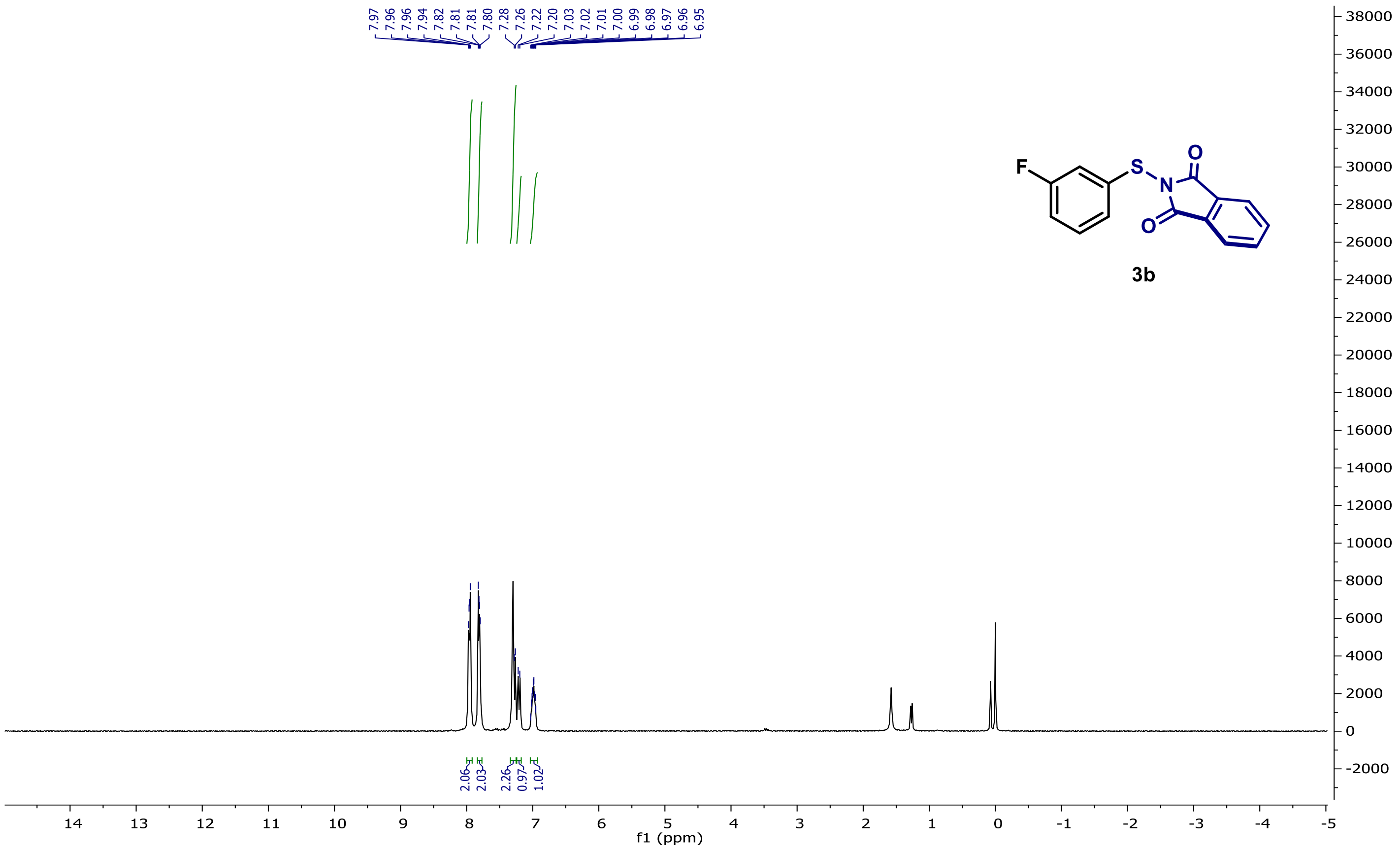

^{19}F{^{1}H} NMR (282 MHz, $CDCl_3$)

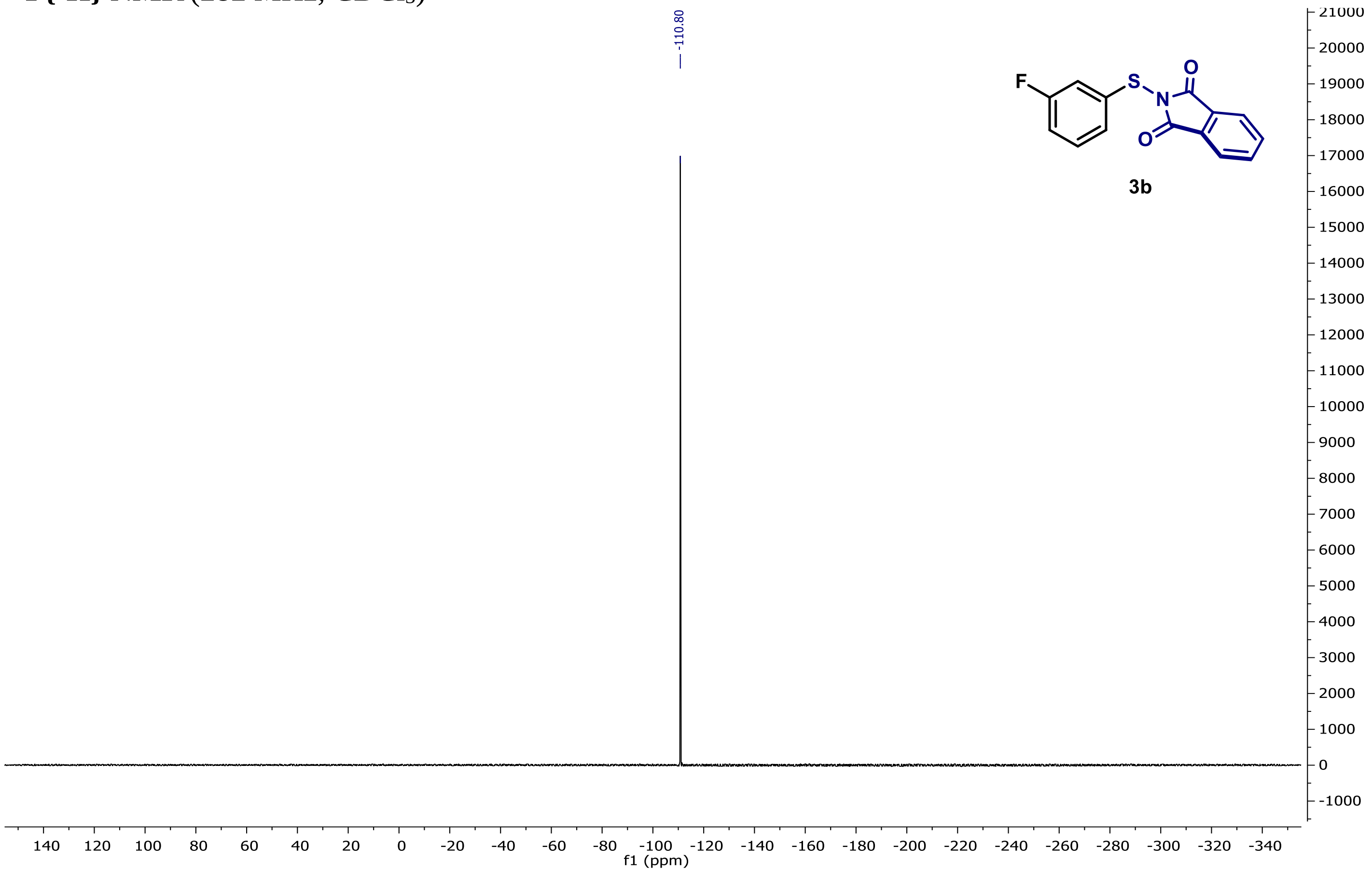

^{13}C Spectra (75 MHz, $CDCl_3$)

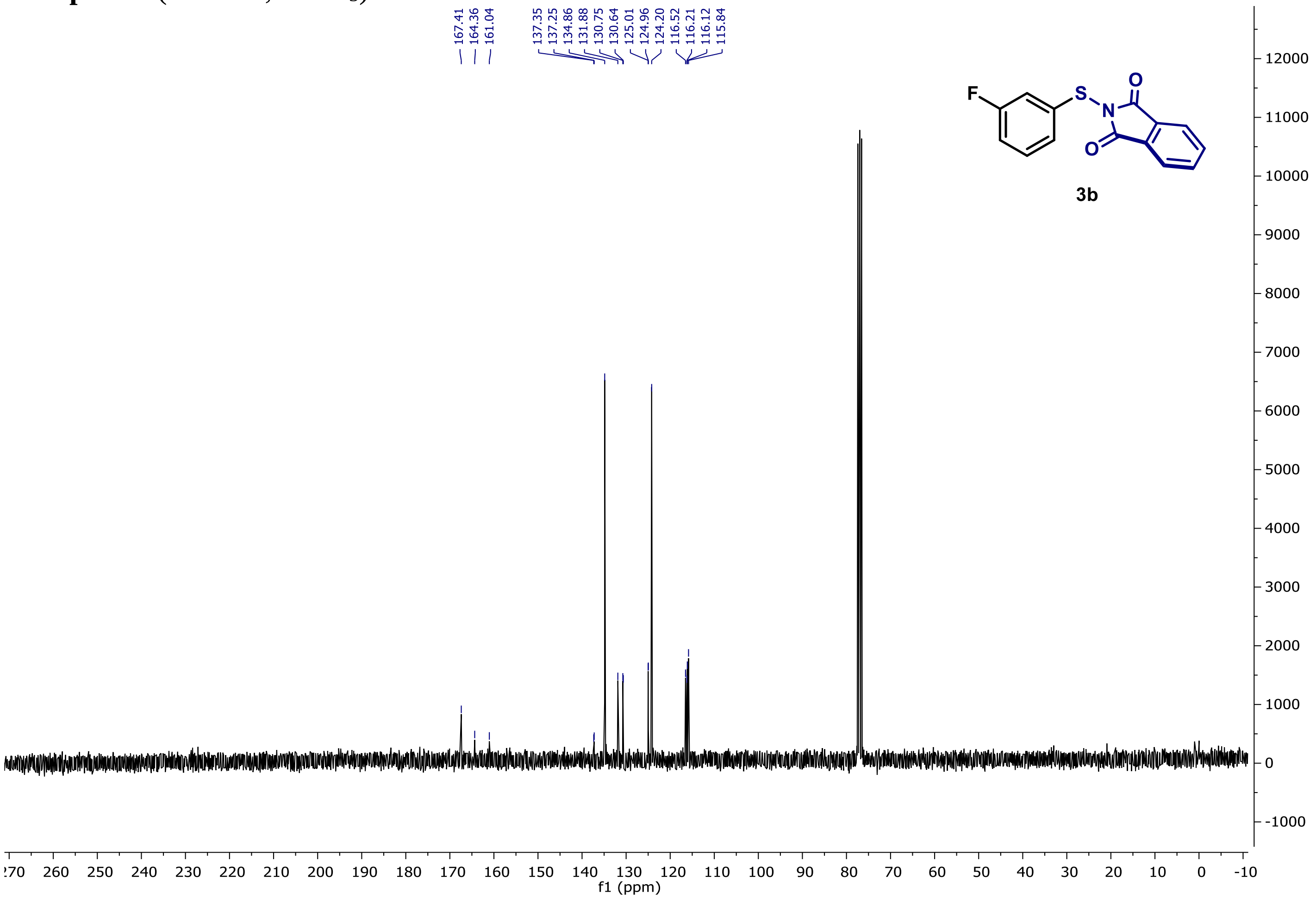

^{1}H Spectra (300 MHz, $CDCl_3$)

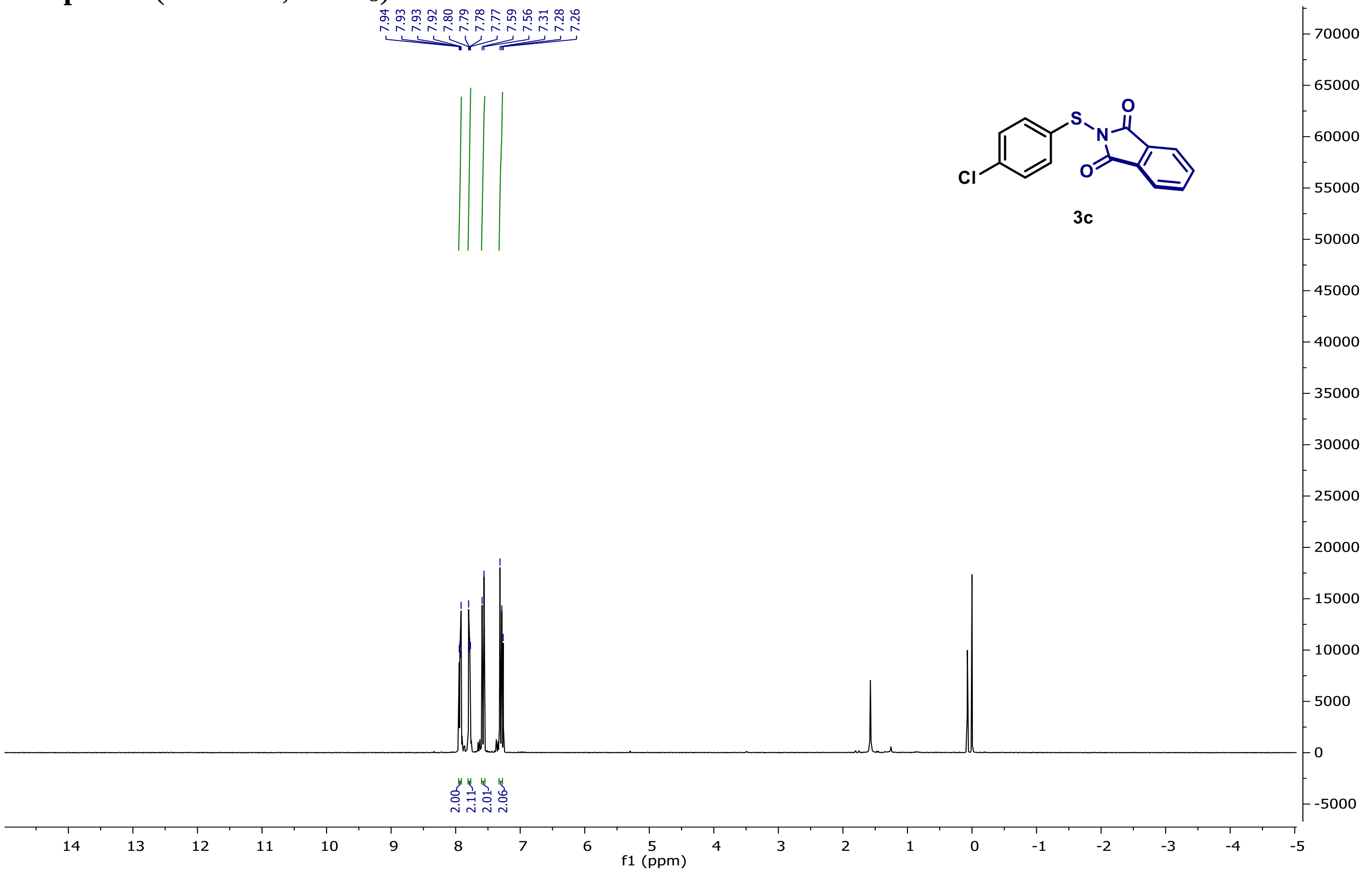

^{13}C Spectra (75 MHz, $CDCl_3$)

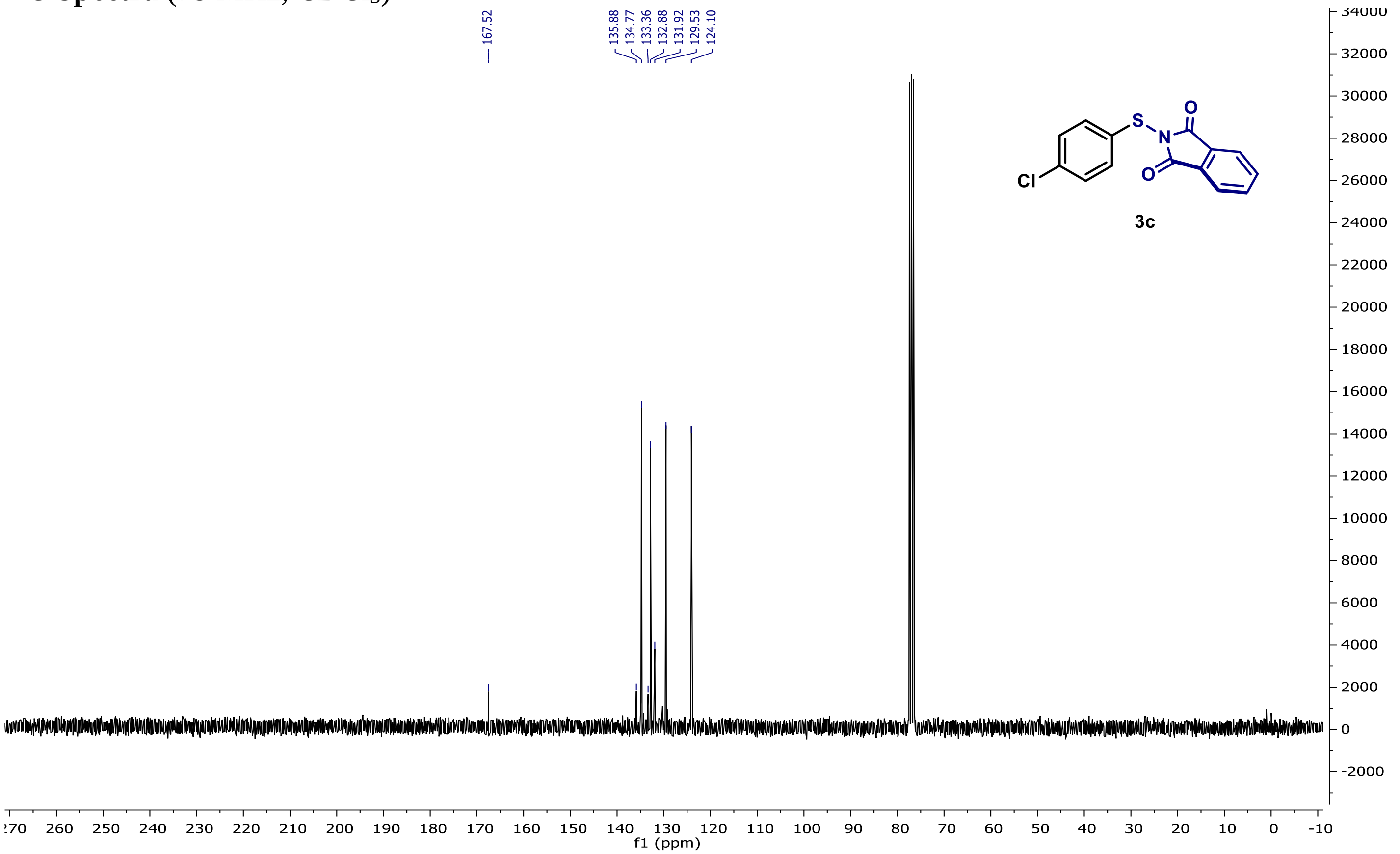

[1]H Spectra (300 MHz, $CDCl_3$)

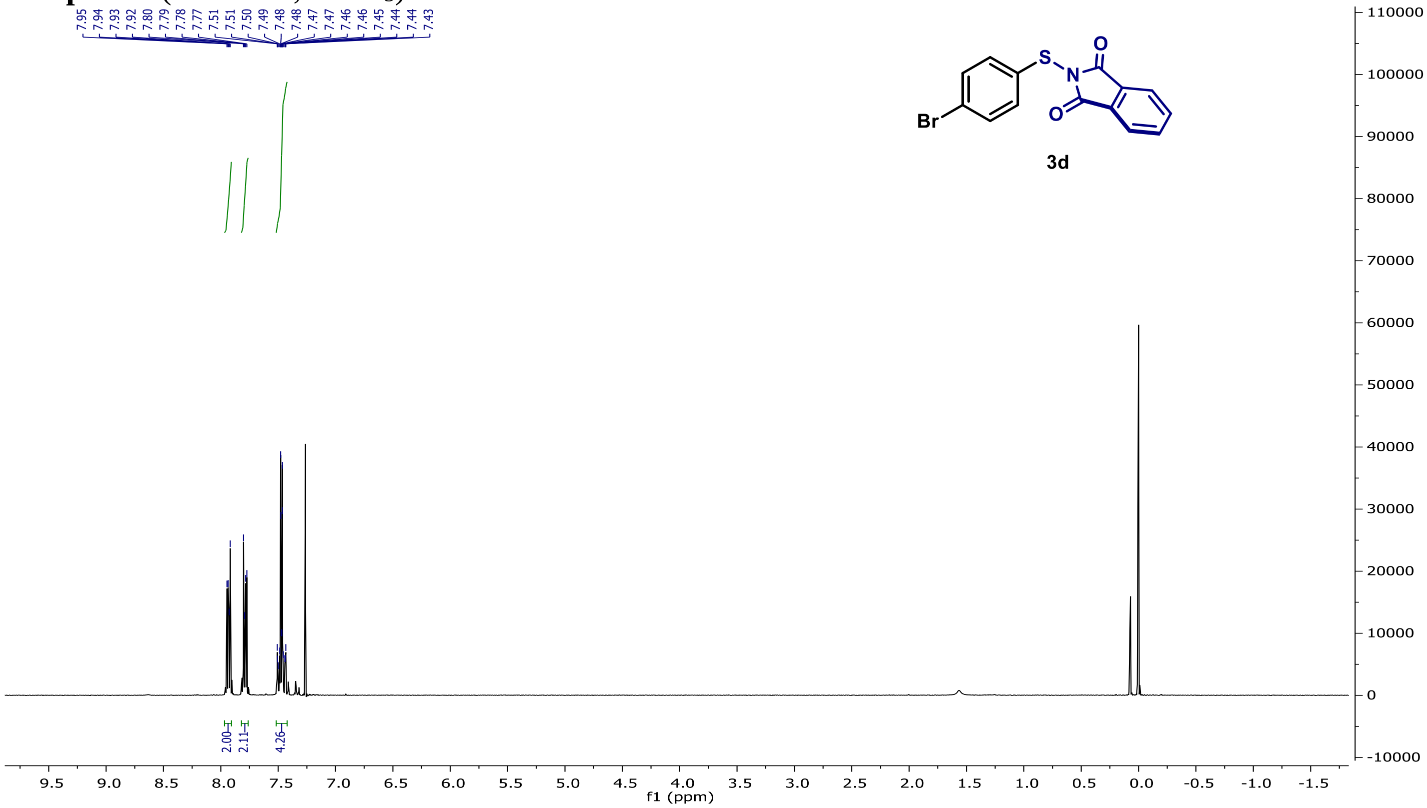

[13]C Spectra (75 MHz, $CDCl_3$)

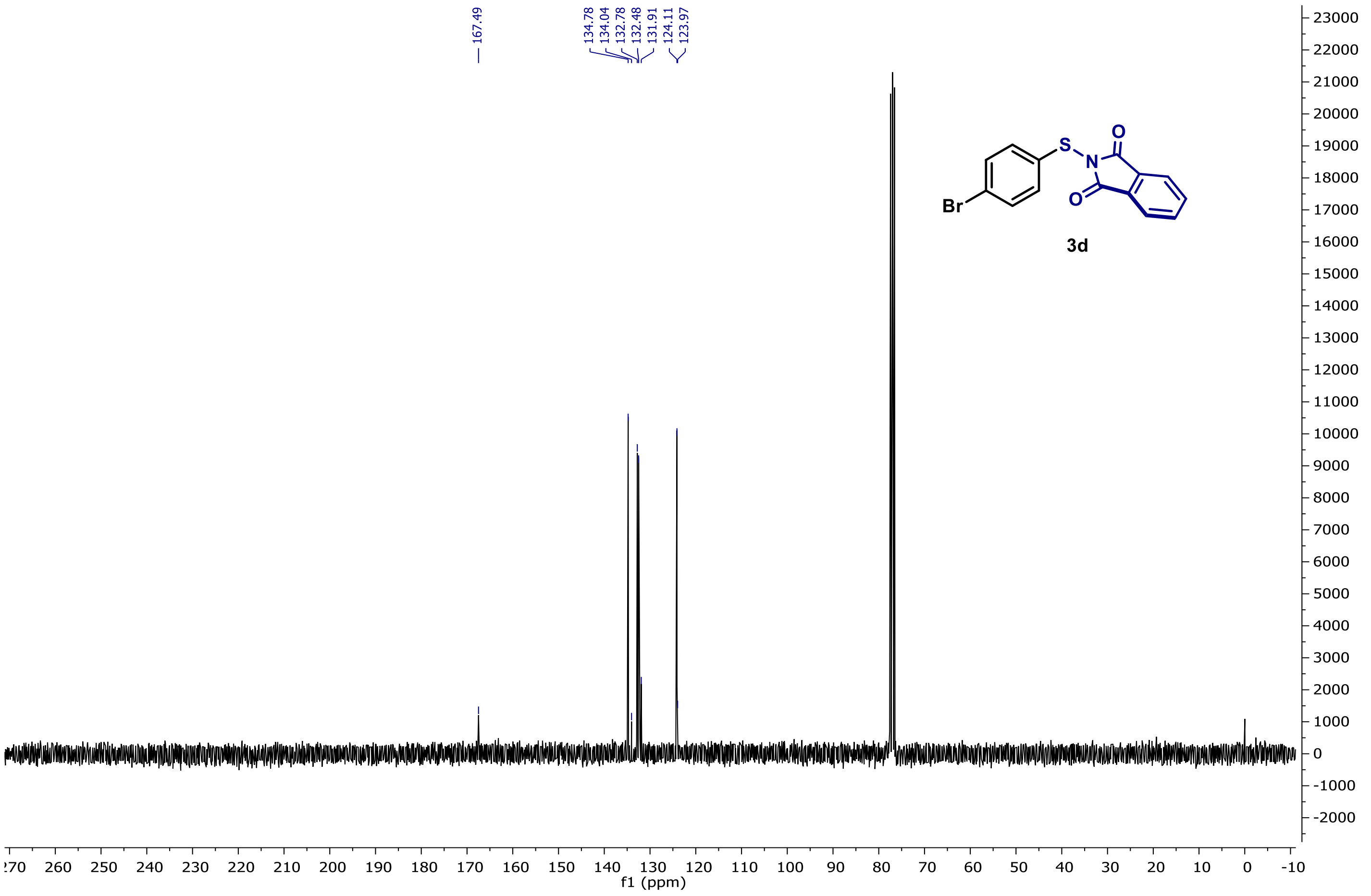

^{1}H Spectra (300 MHz, $CDCl_3$)

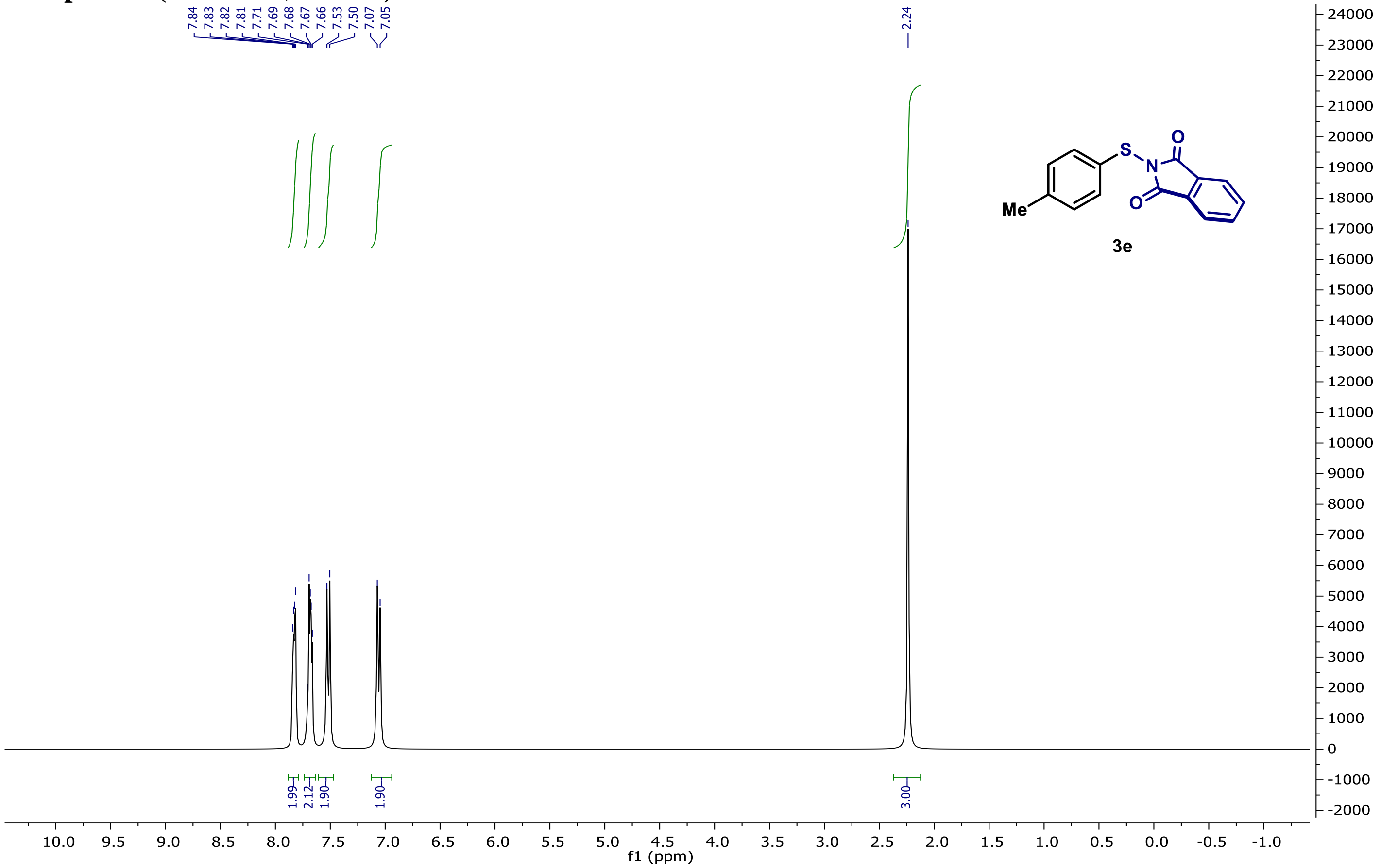

^{13}C Spectra (75 MHz, $CDCl_3$)

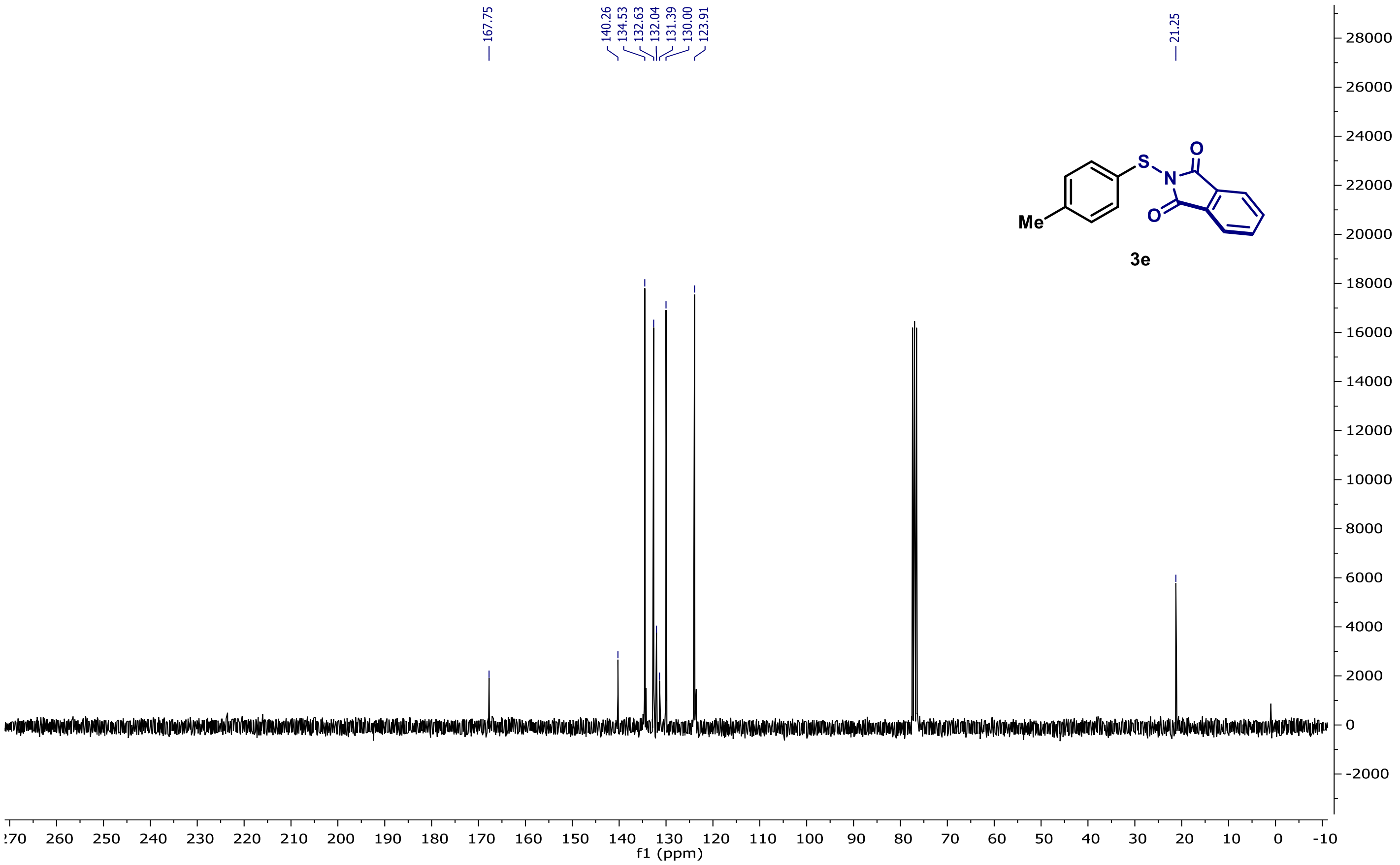

[1]H Spectra (300 MHz, $CDCl_3$)

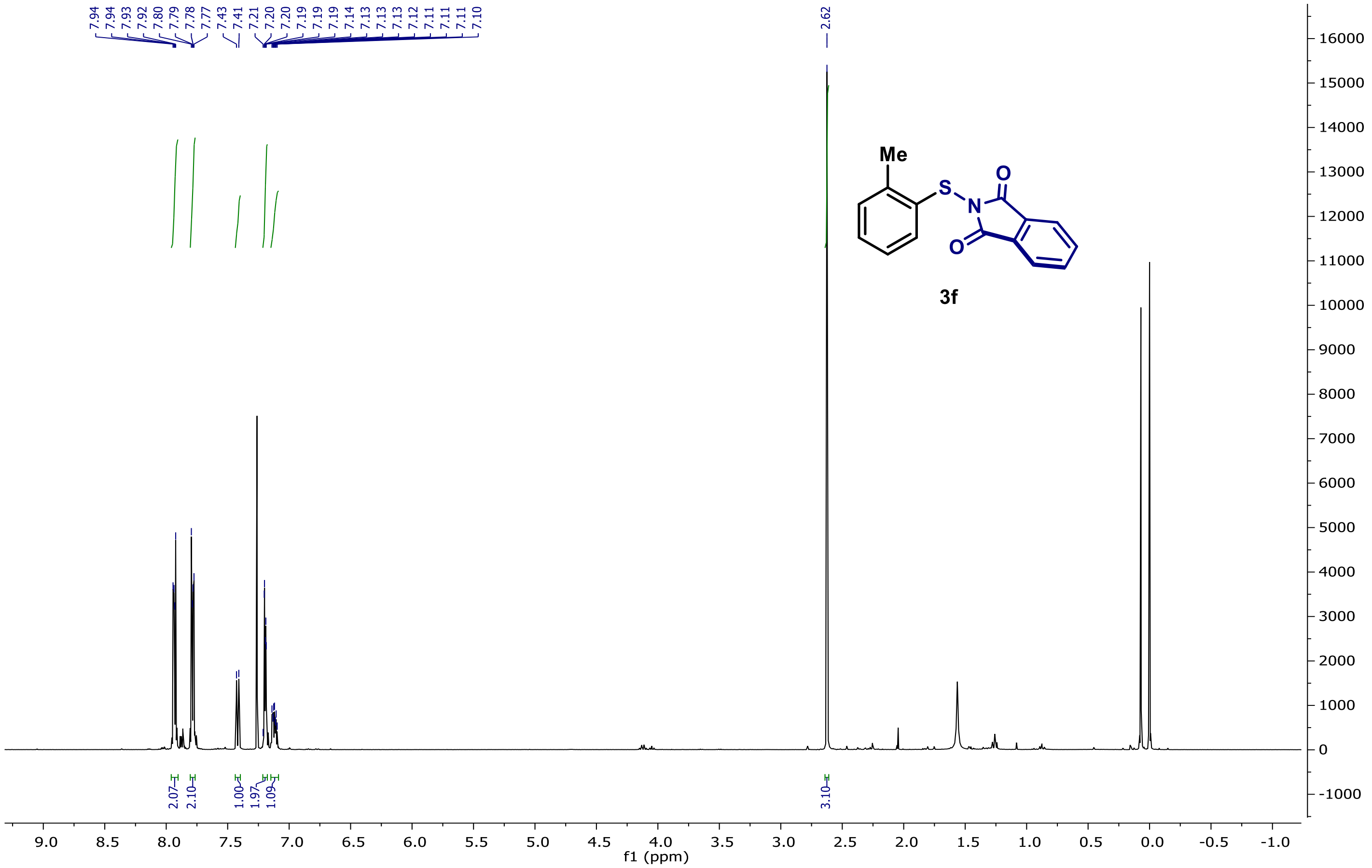

[13]C Spectra (75 MHz, $CDCl_3$)

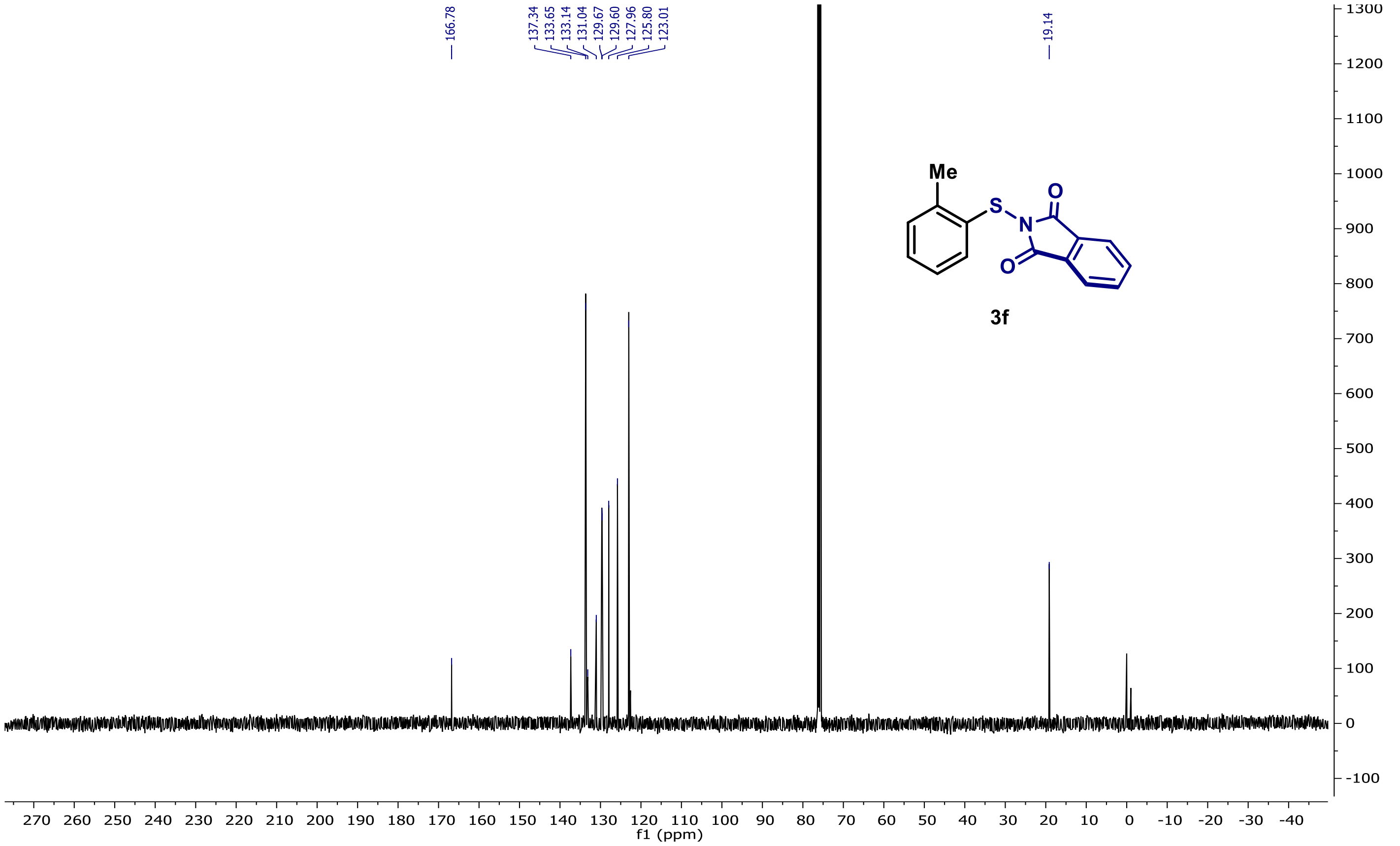

[1]H Spectra (300 MHz, $CDCl_3$)

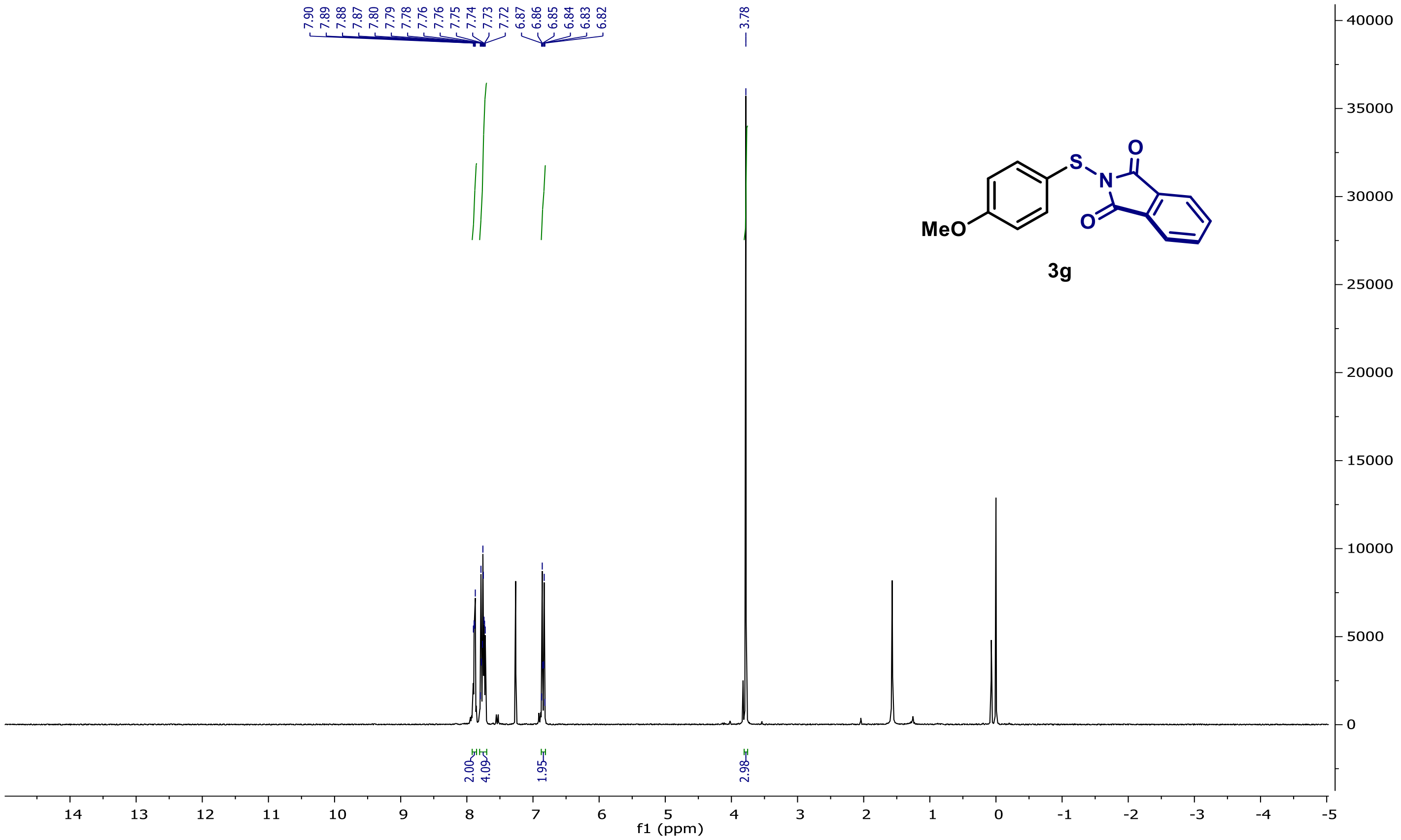

[13]C Spectra (75 MHz, $CDCl_3$)

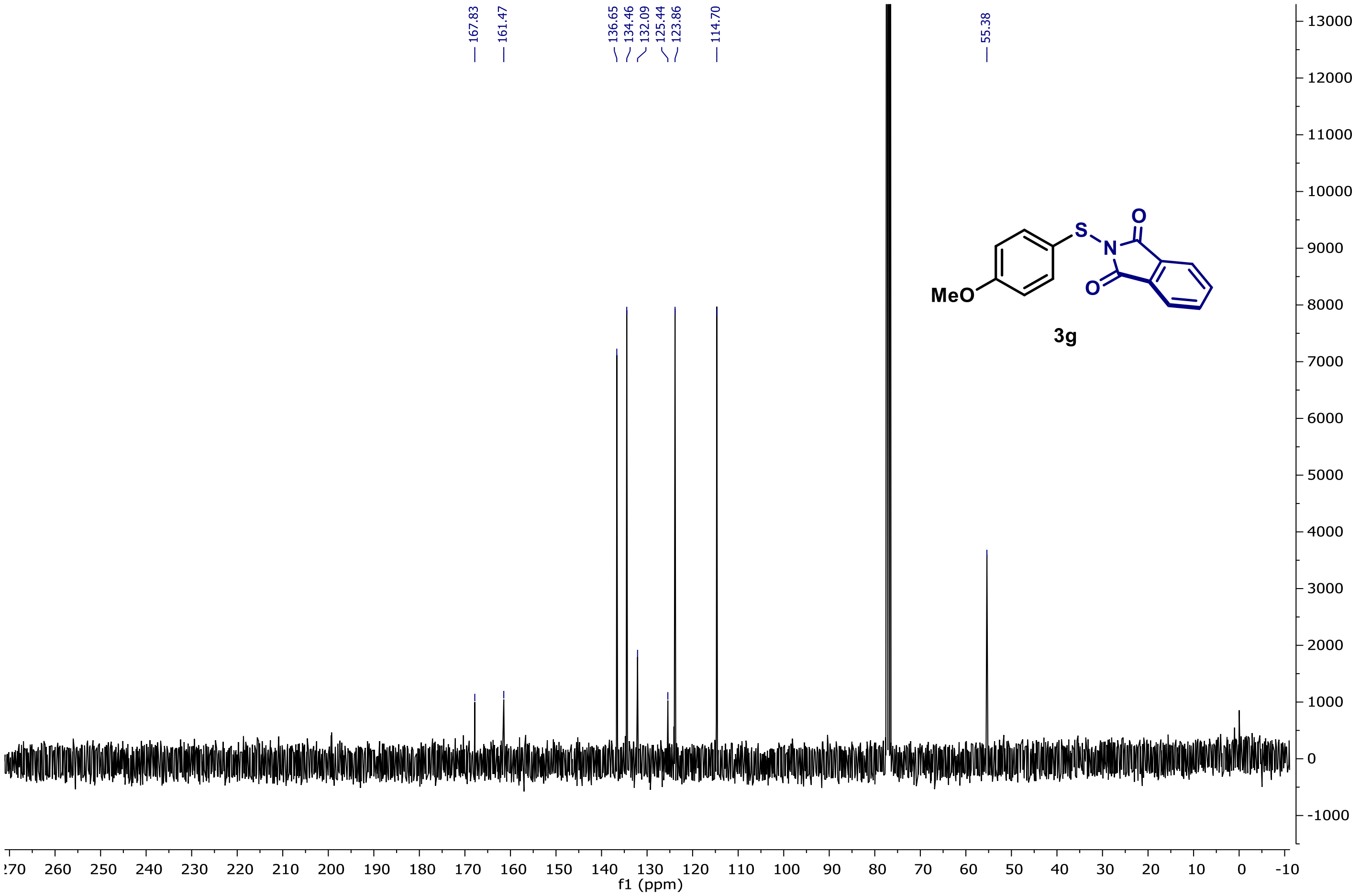

^{1}H Spectra (300 MHz, $CDCl_3$)

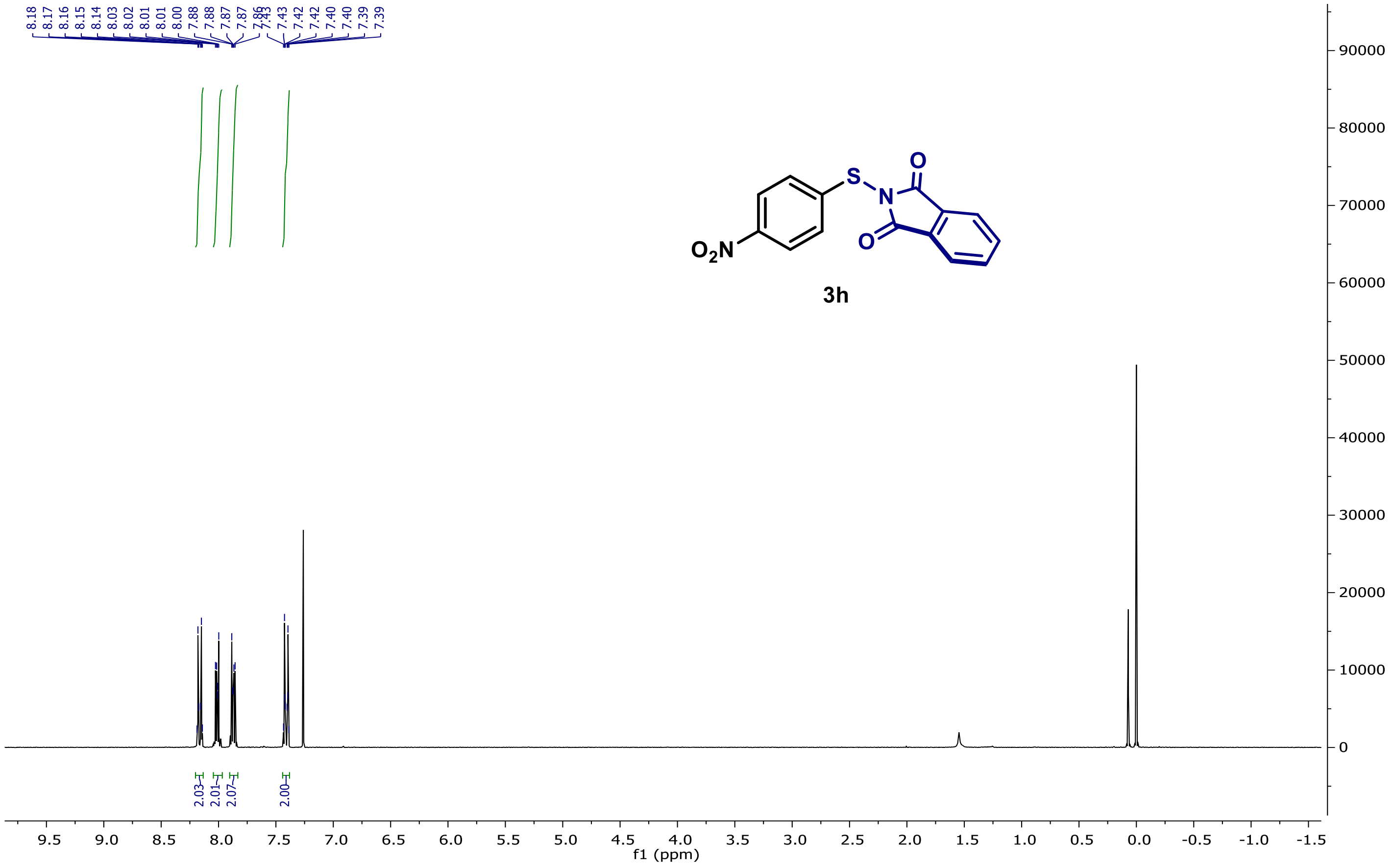

^{13}C Spectra (75 MHz, $CDCl_3$)

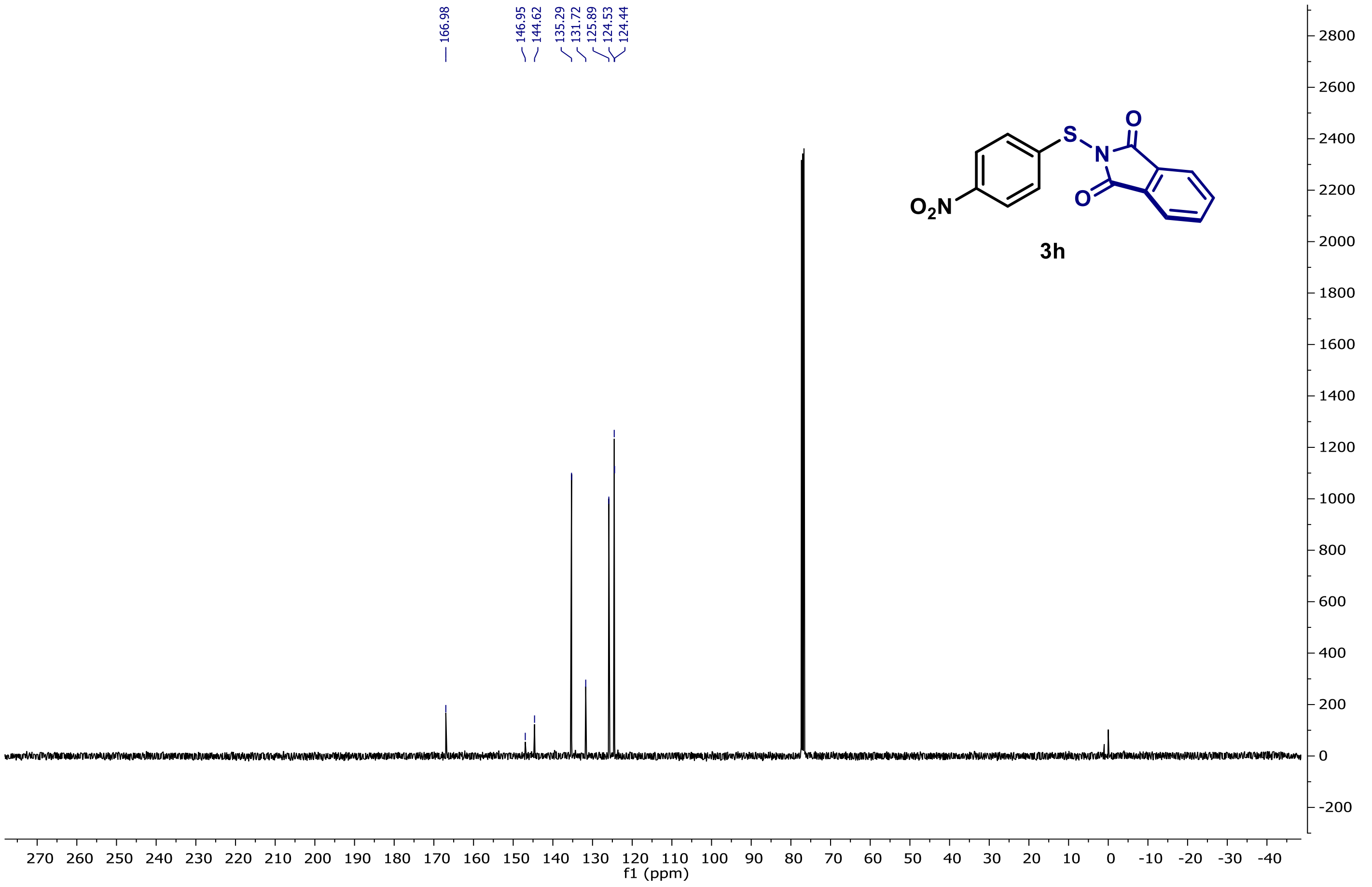

^{1}H Spectra (300 MHz, $CDCl_3$)

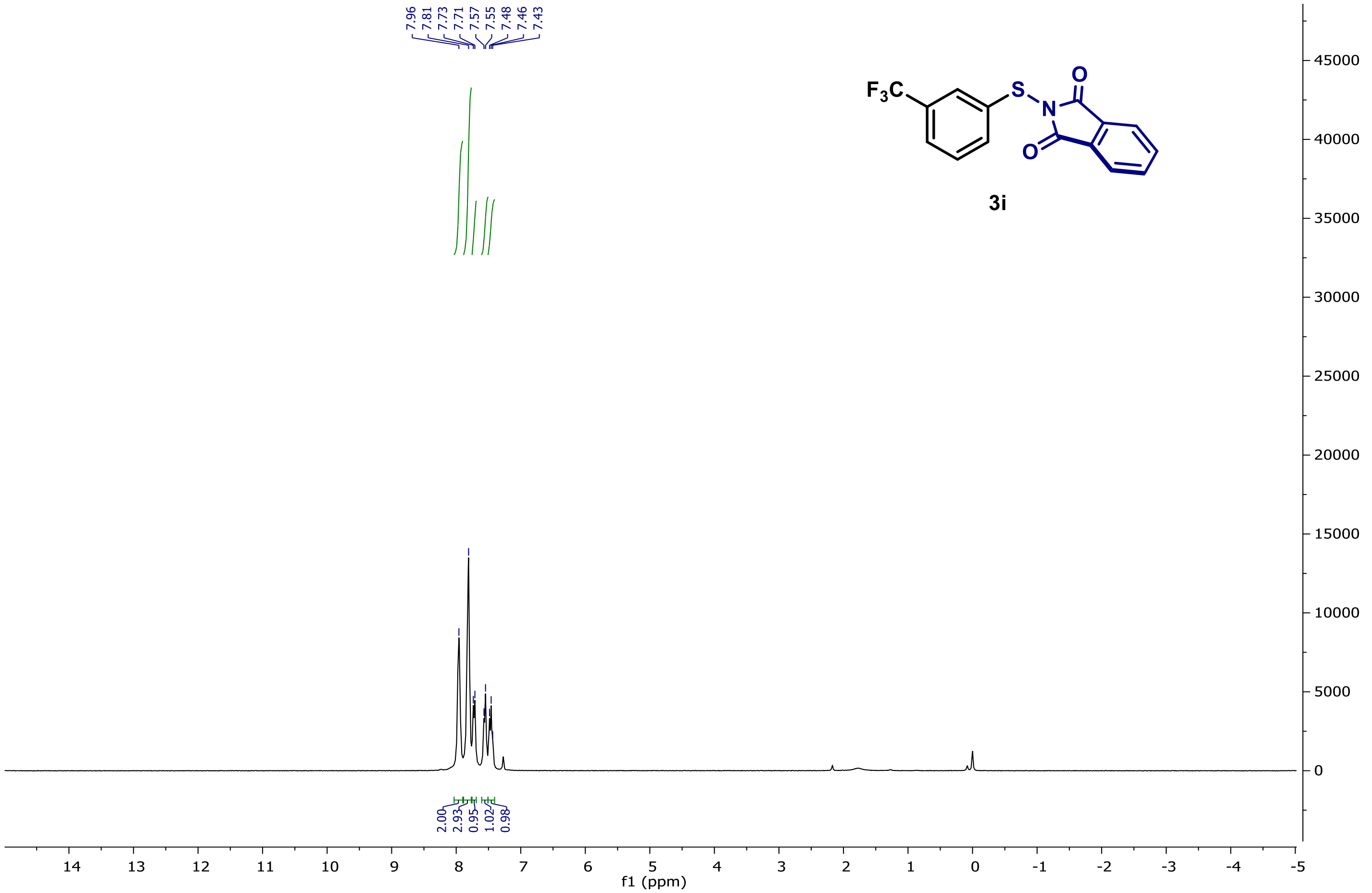

^{19}F{^{1}H} NMR (282 MHz, $CDCl_3$)

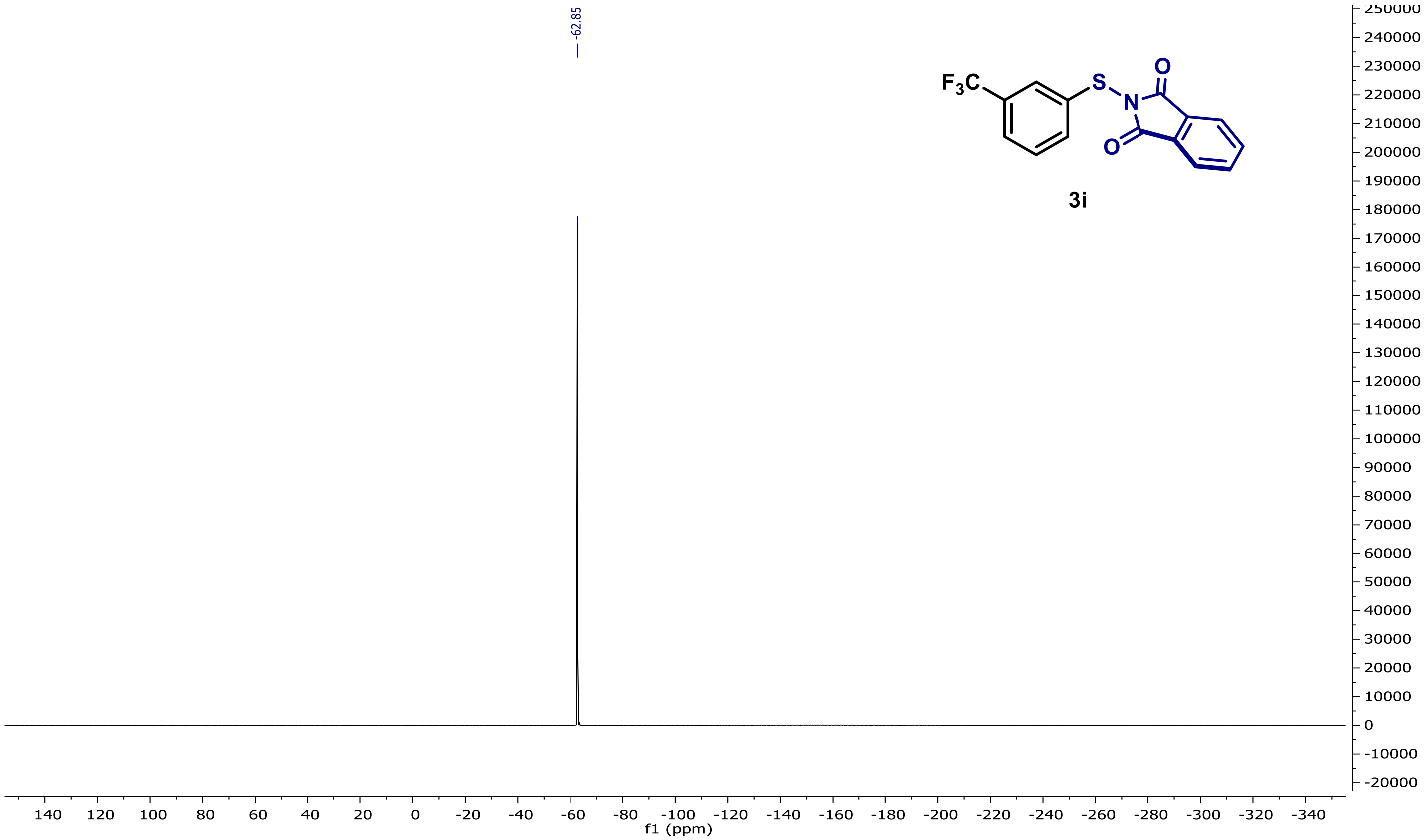

^{13}C Spectra (75 MHz, $CDCl_3$)

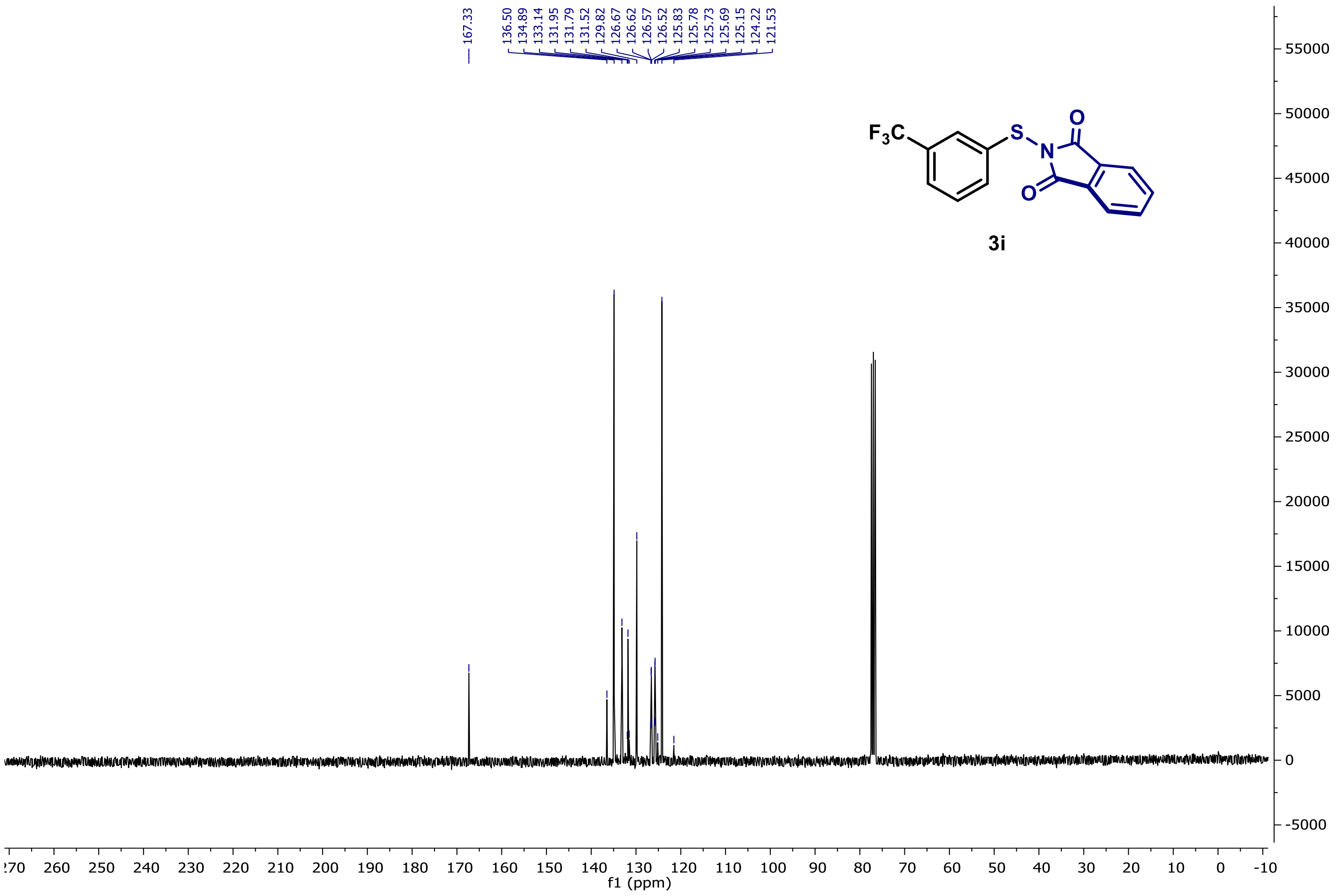

^{1}**H Spectra** (300 MHz, $CDCl_3$)

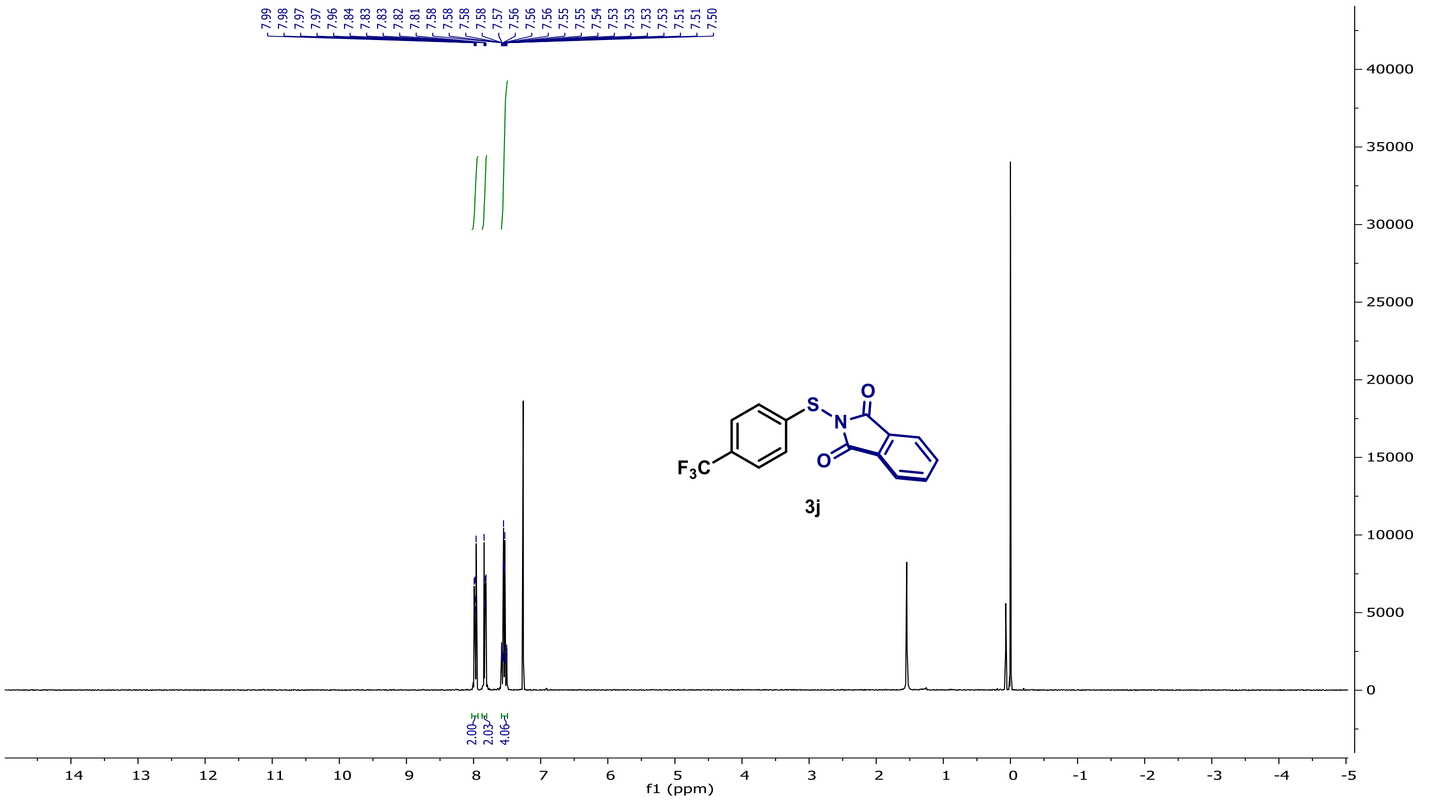

^{19}**F{^{1}H} NMR** (282 MHz, $CDCl_3$)

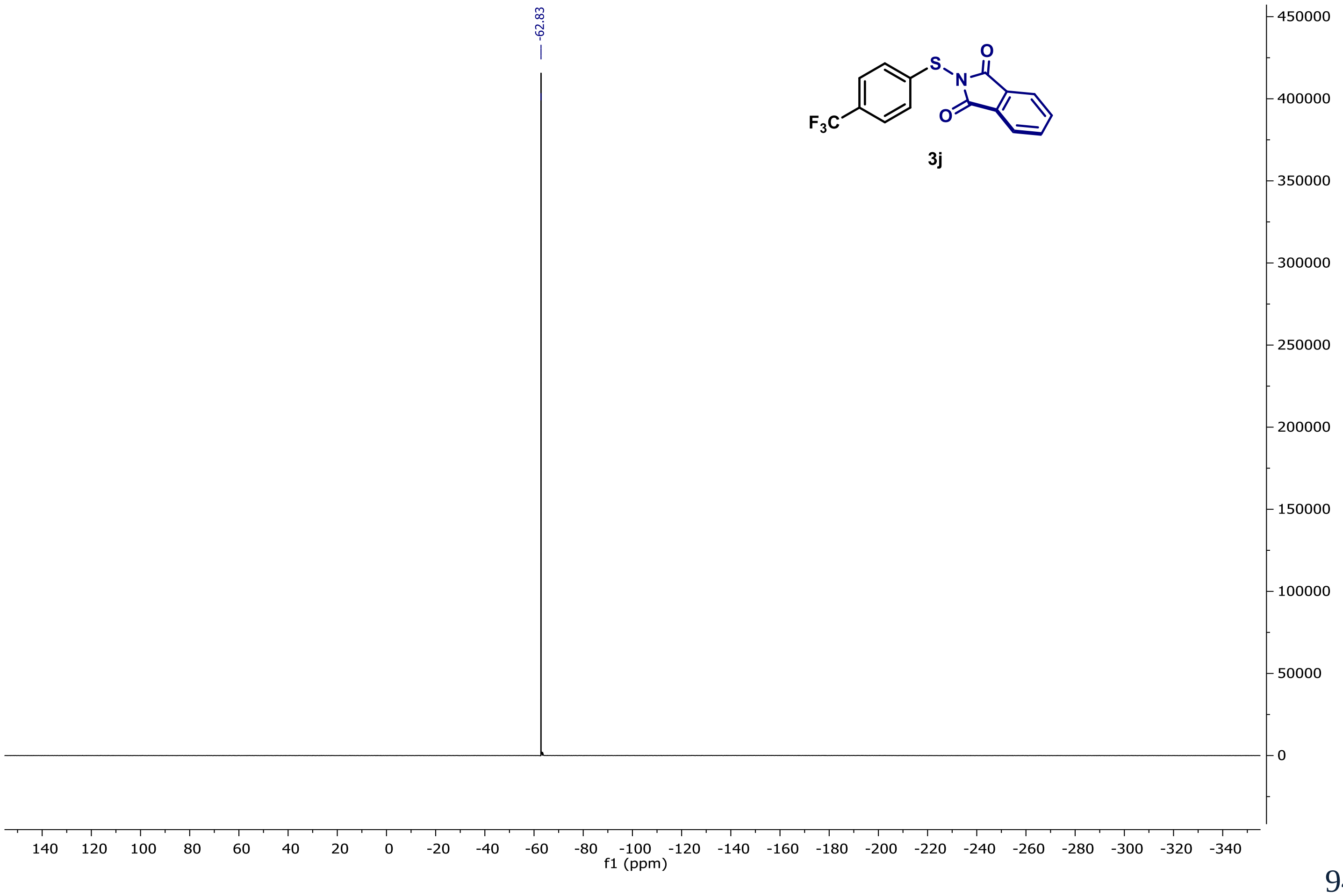

^{13}C Spectra (75 MHz, $CDCl_3$)

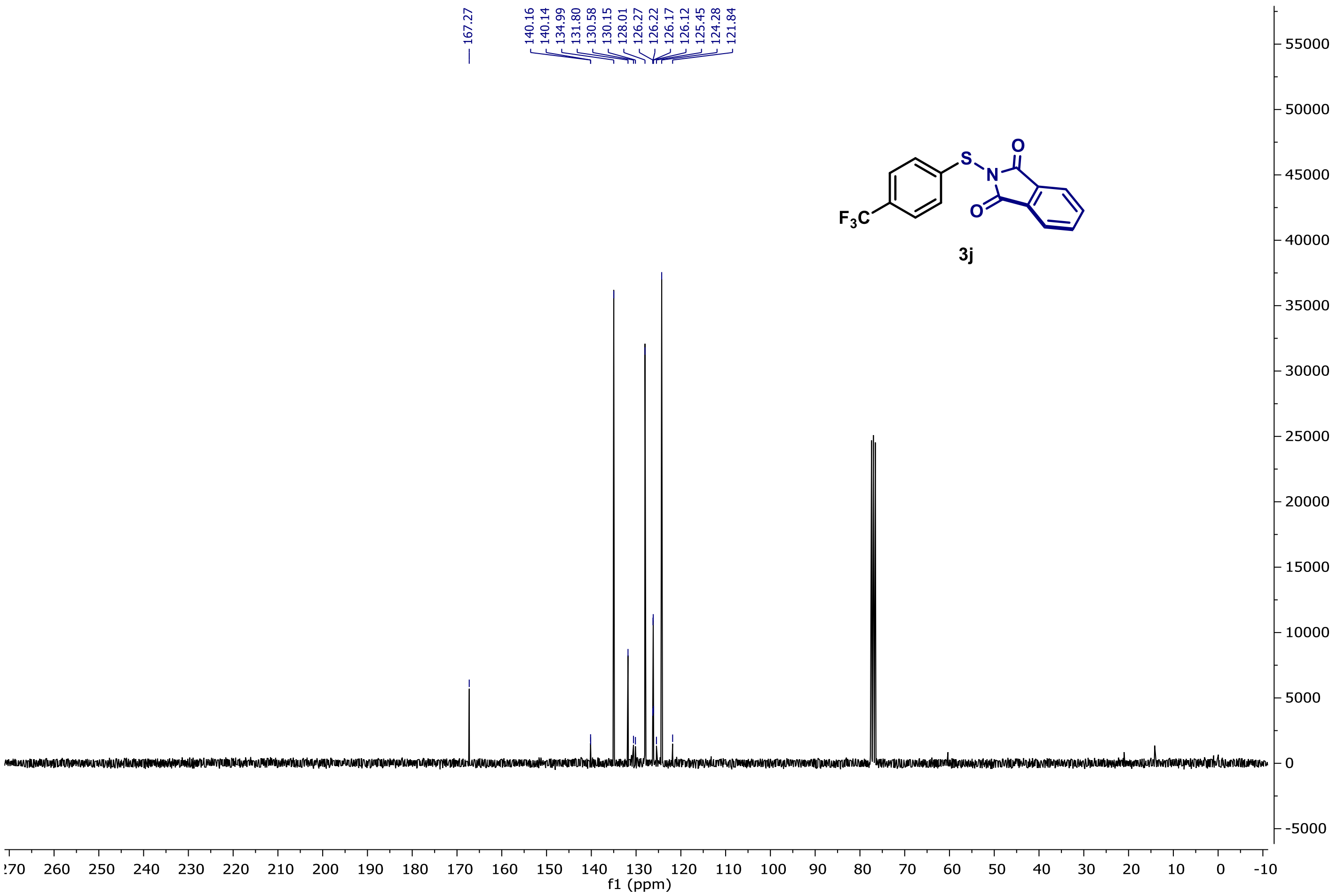

^{1}**H Spectra** (300 MHz, $CDCl_3$)

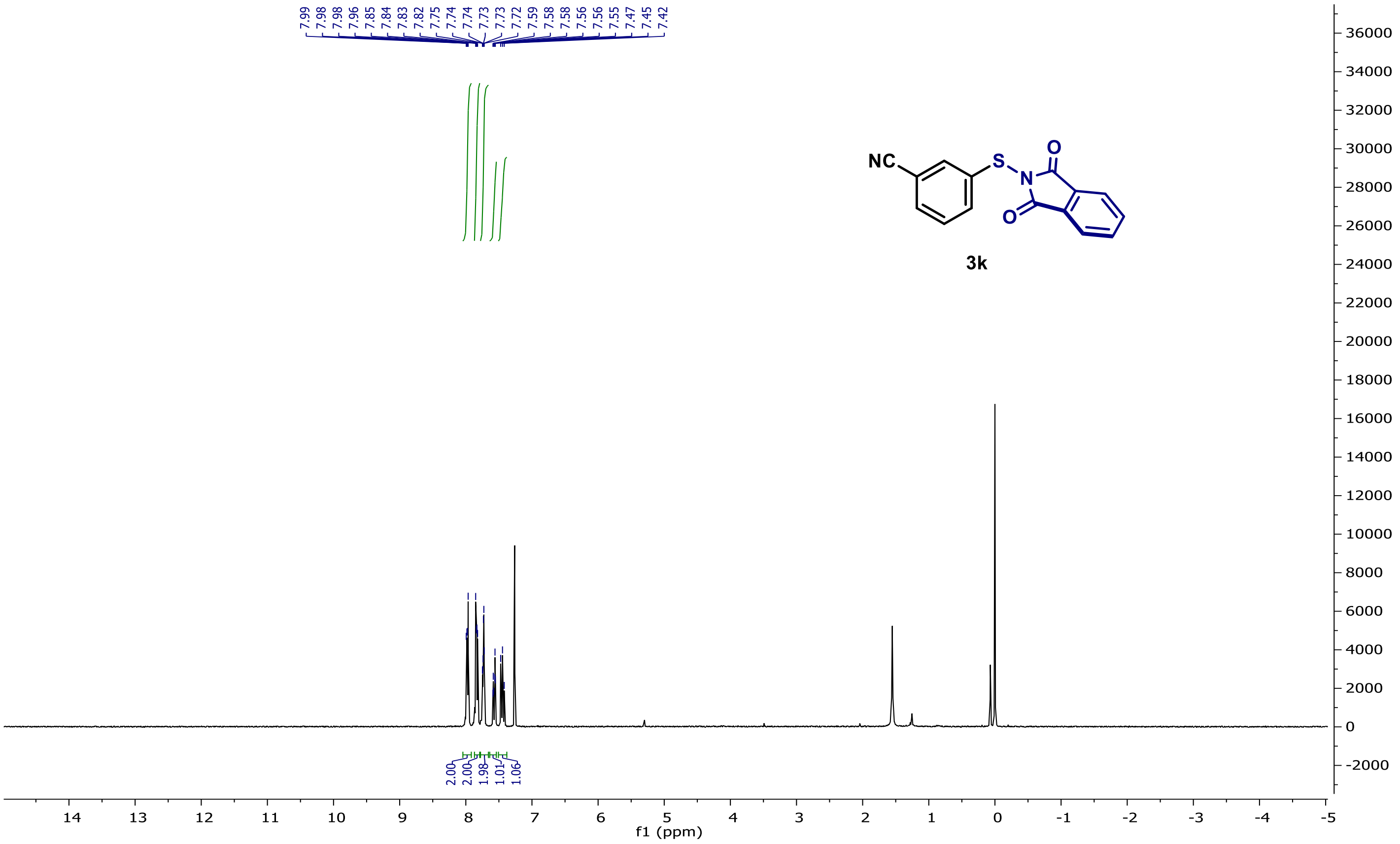

^{13}**C Spectra** (75 MHz, $CDCl_3$)

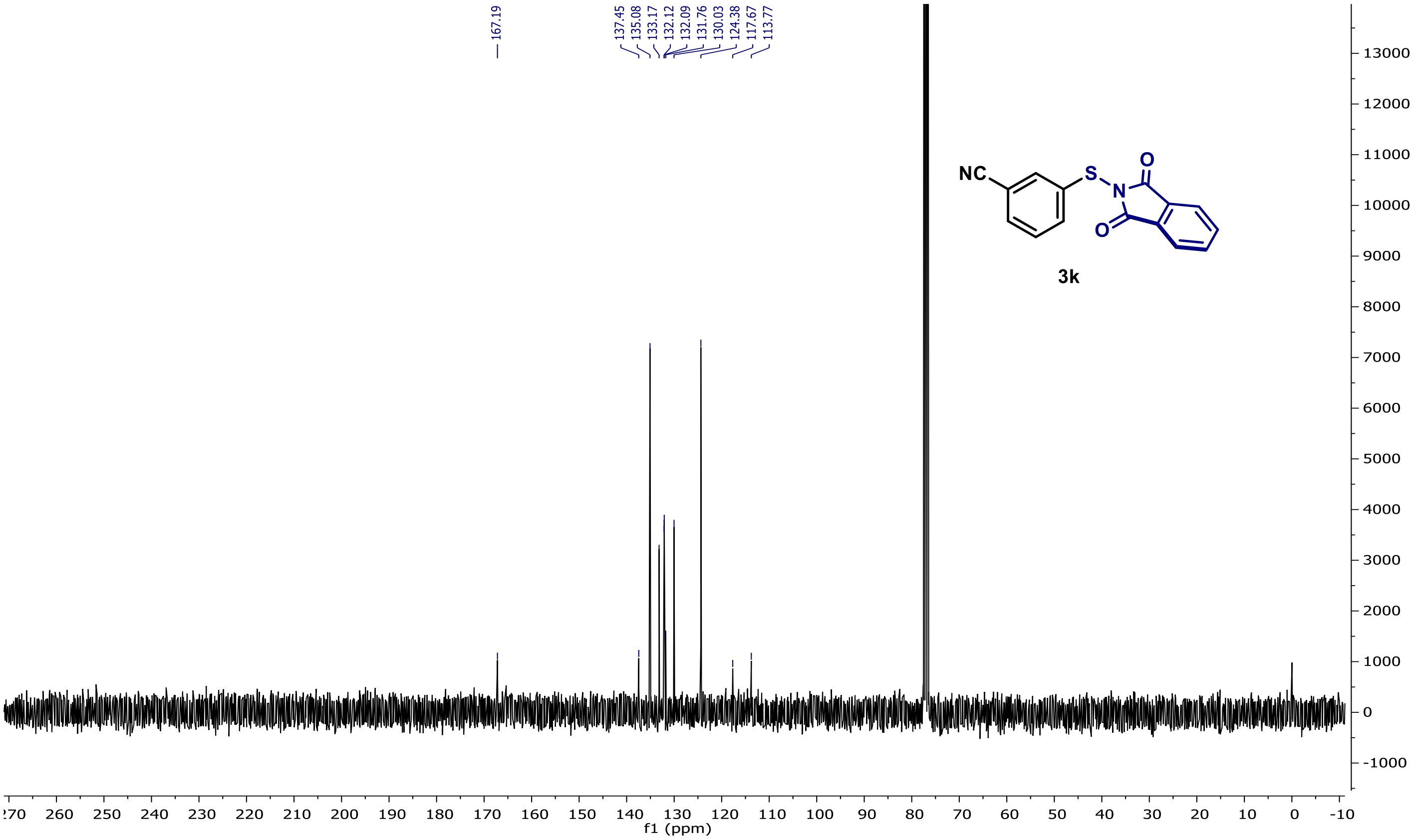

^{1}H Spectra (300 MHz, $CDCl_3$)

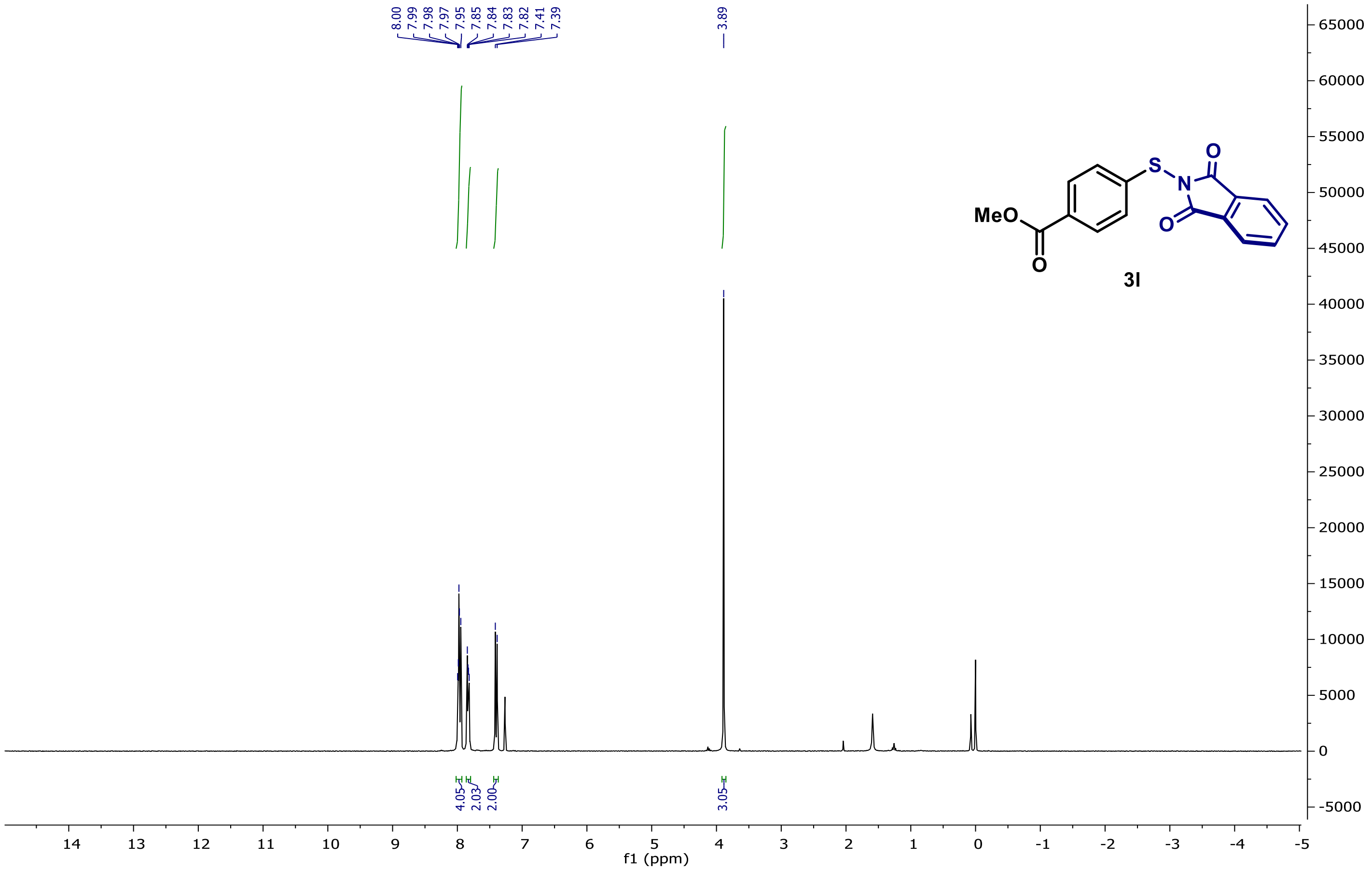

^{13}C Spectra (75 MHz, $CDCl_3$)

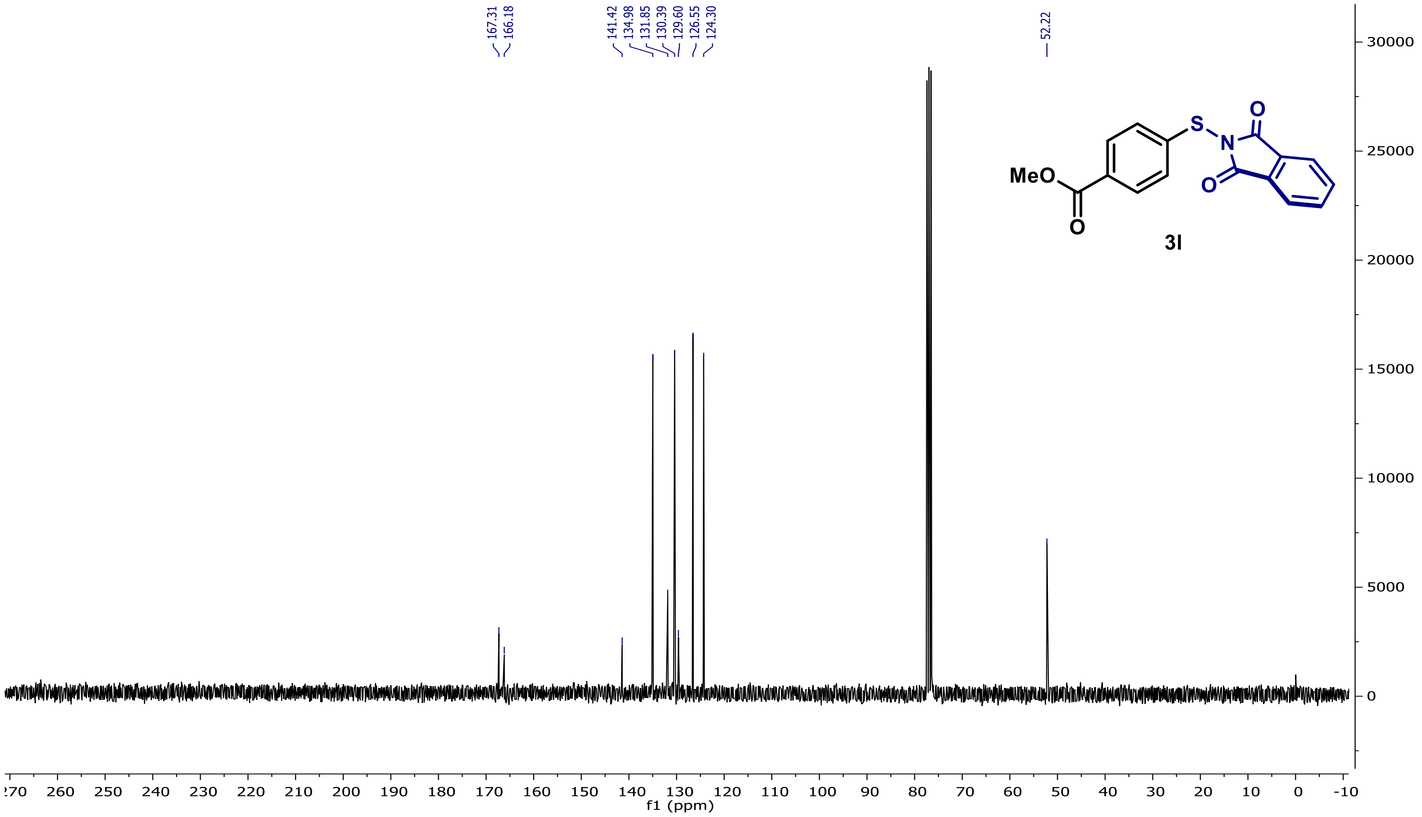

^{1}H Spectra (300 MHz, $CDCl_3$)

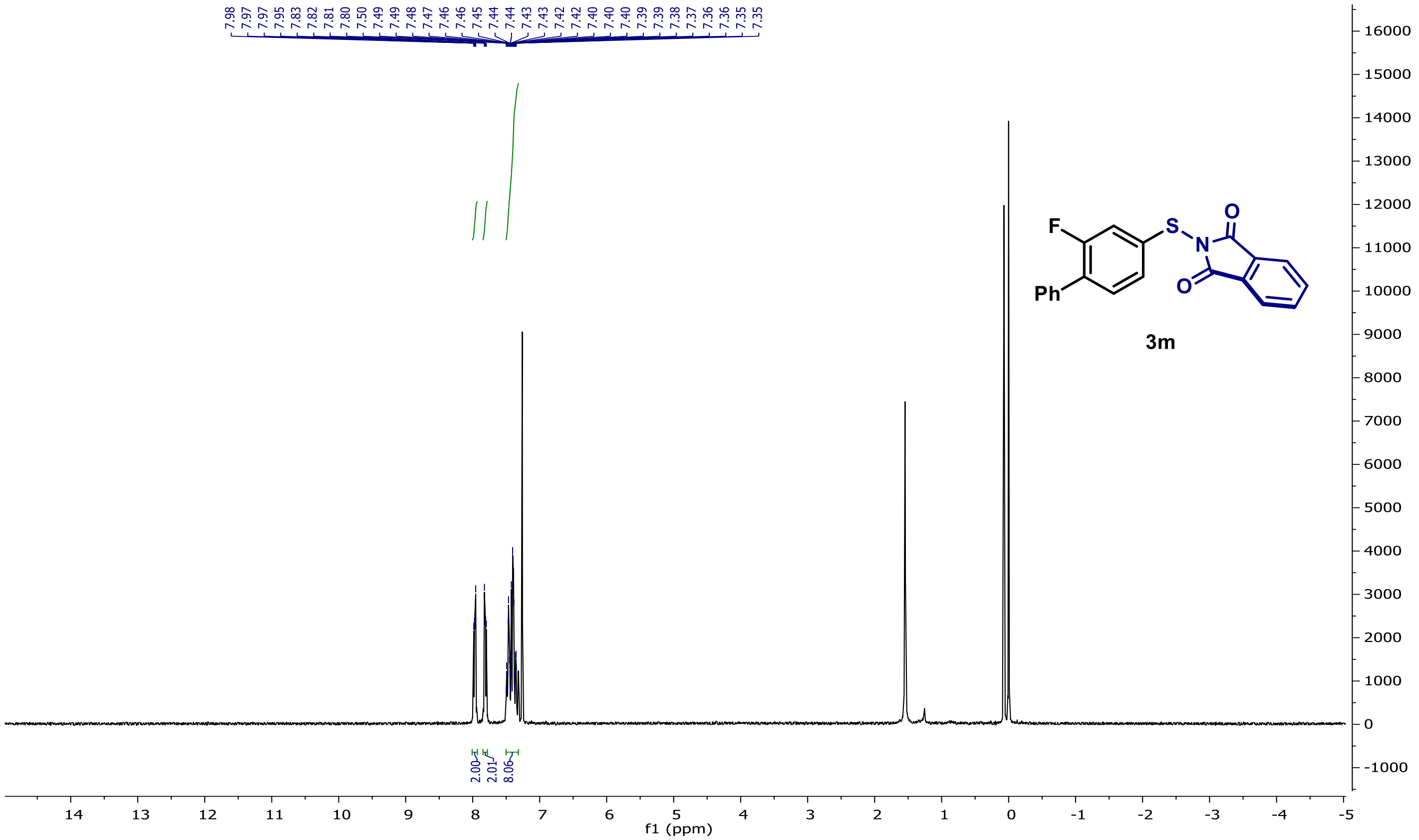

^{19}F{^{1}H} NMR (282 MHz, $CDCl_3$)

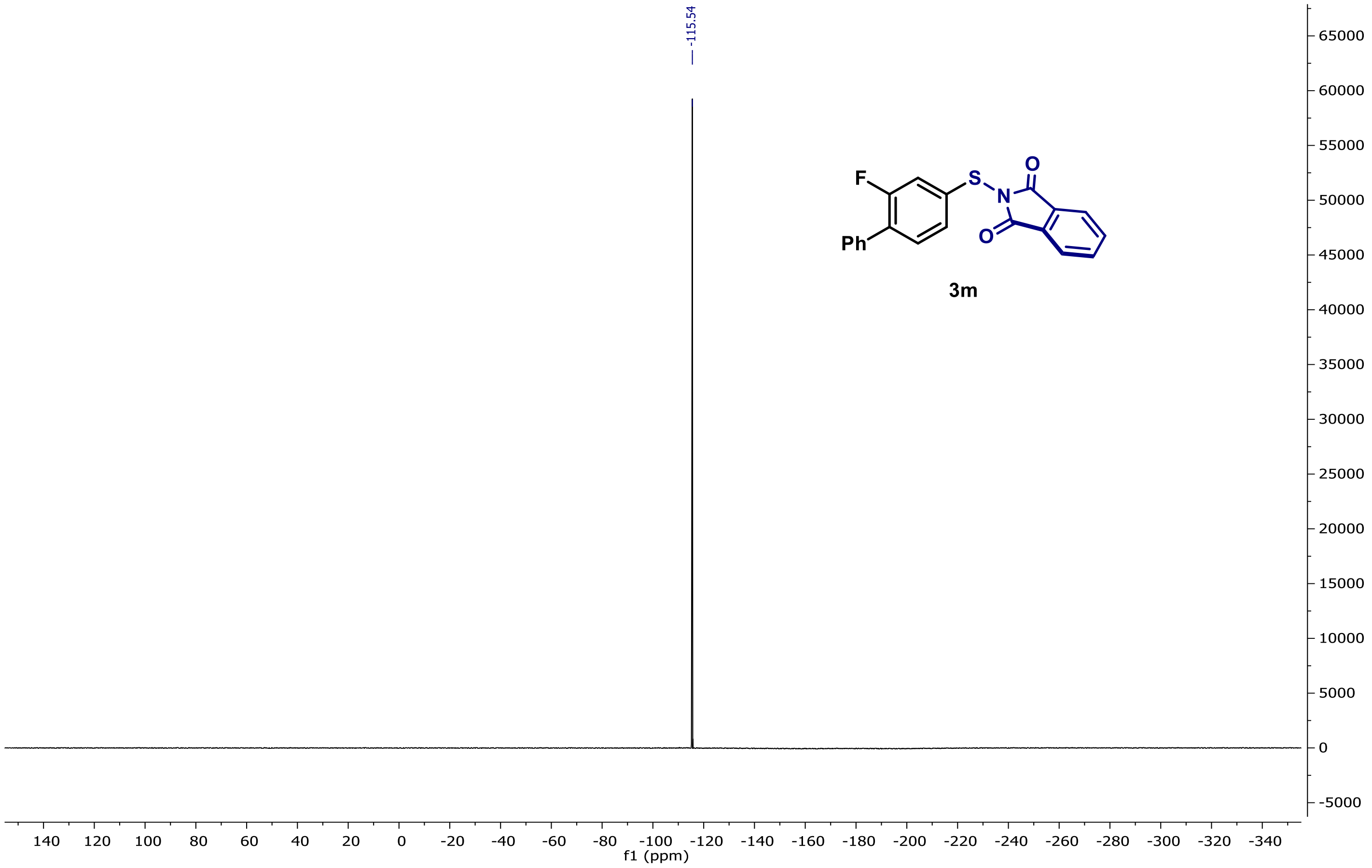

[13]C Spectra (75 MHz, $CDCl_3$)

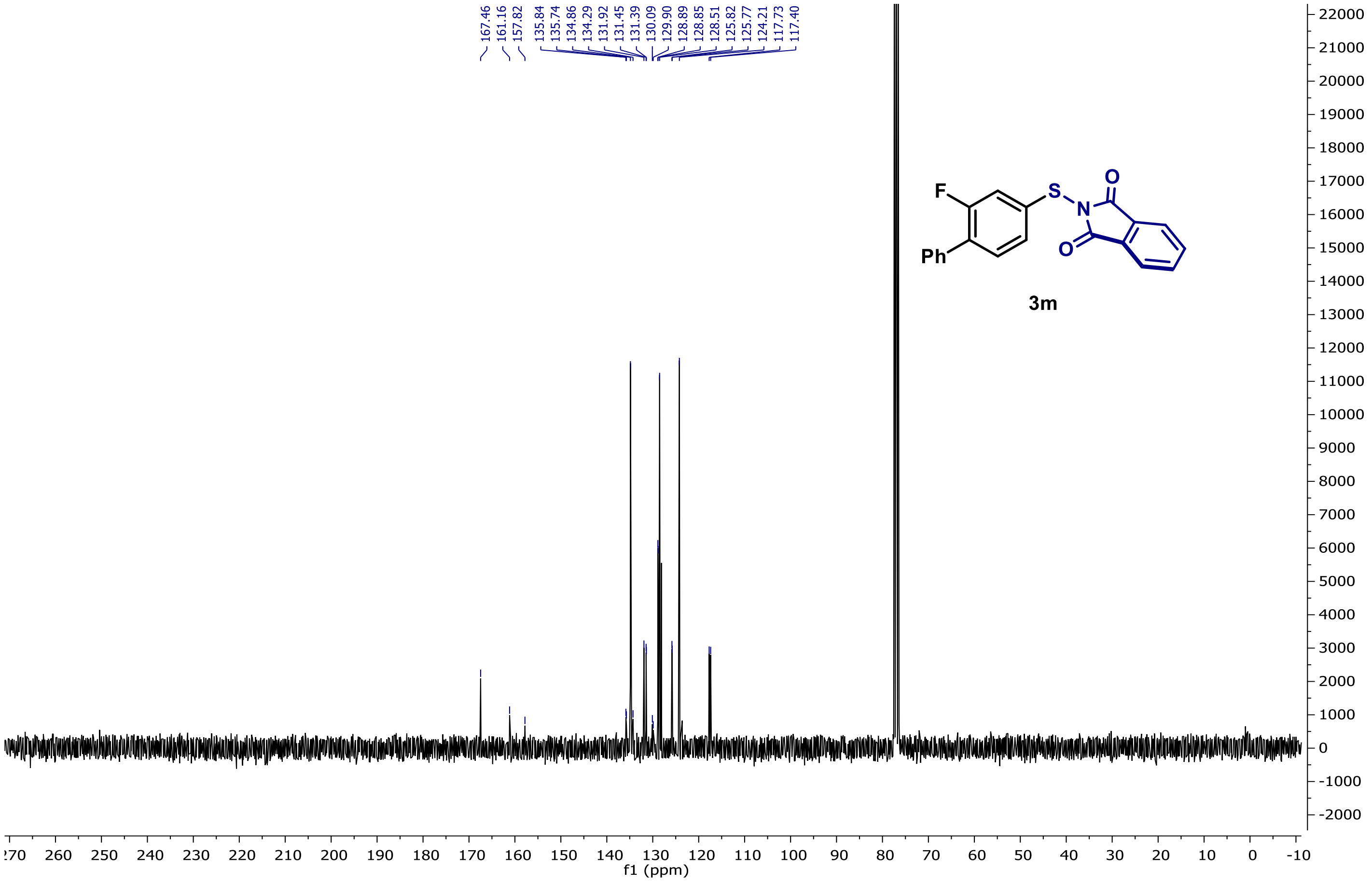

^{1}H Spectra (300 MHz, $CDCl_3$)

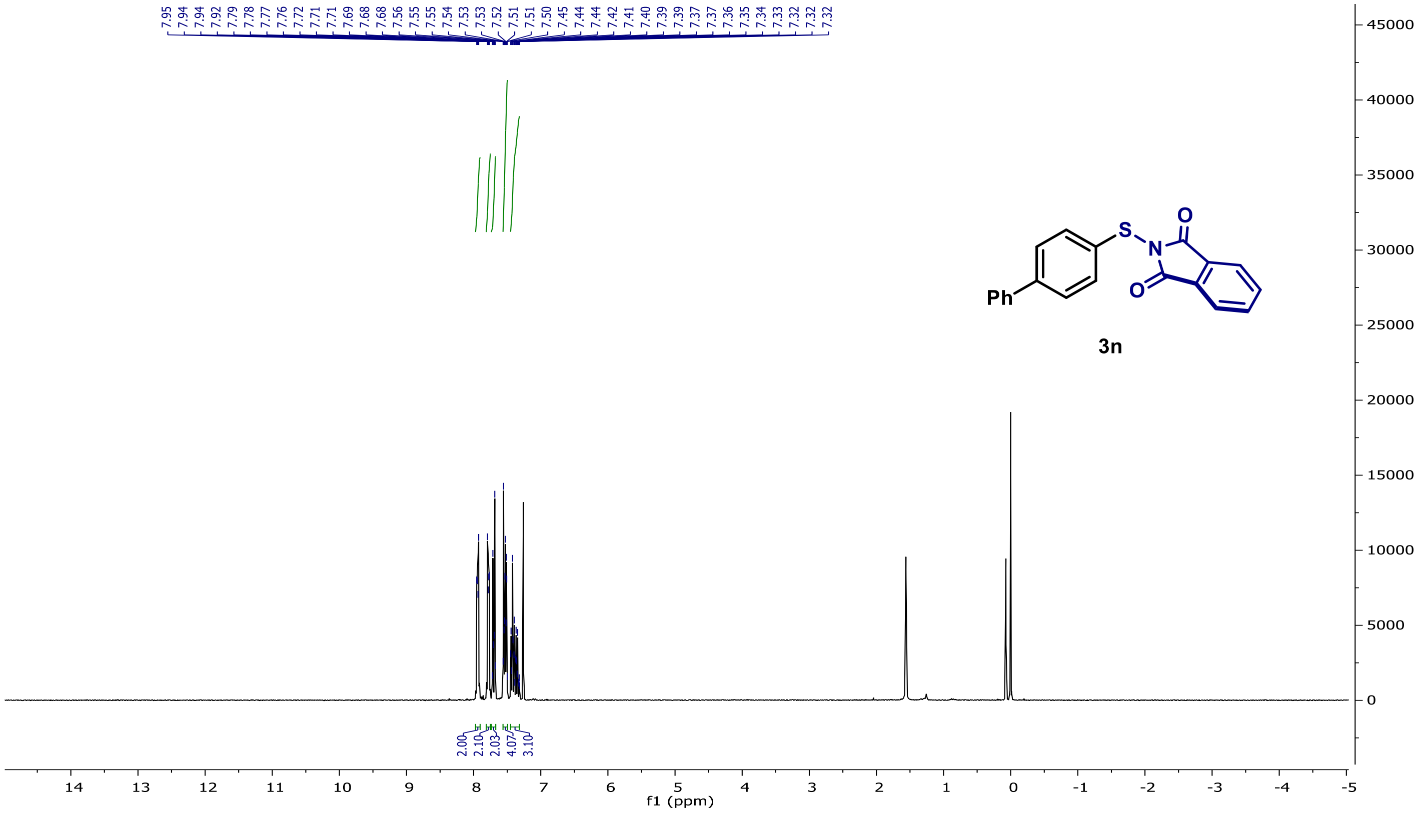

^{31}C Spectra (75 MHz, $CDCl_3$)

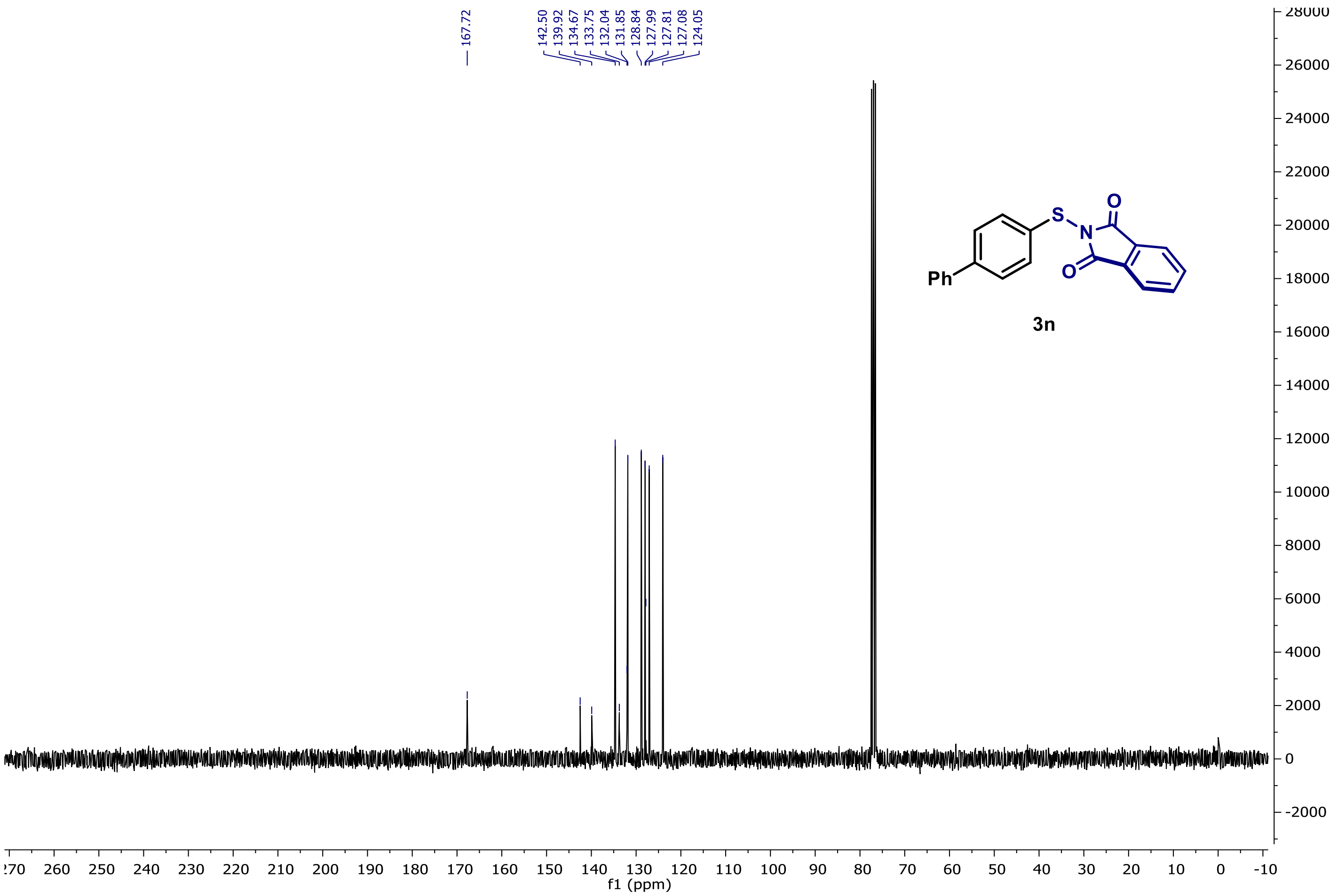

^{1}H Spectra (300 MHz, $CDCl_3$)

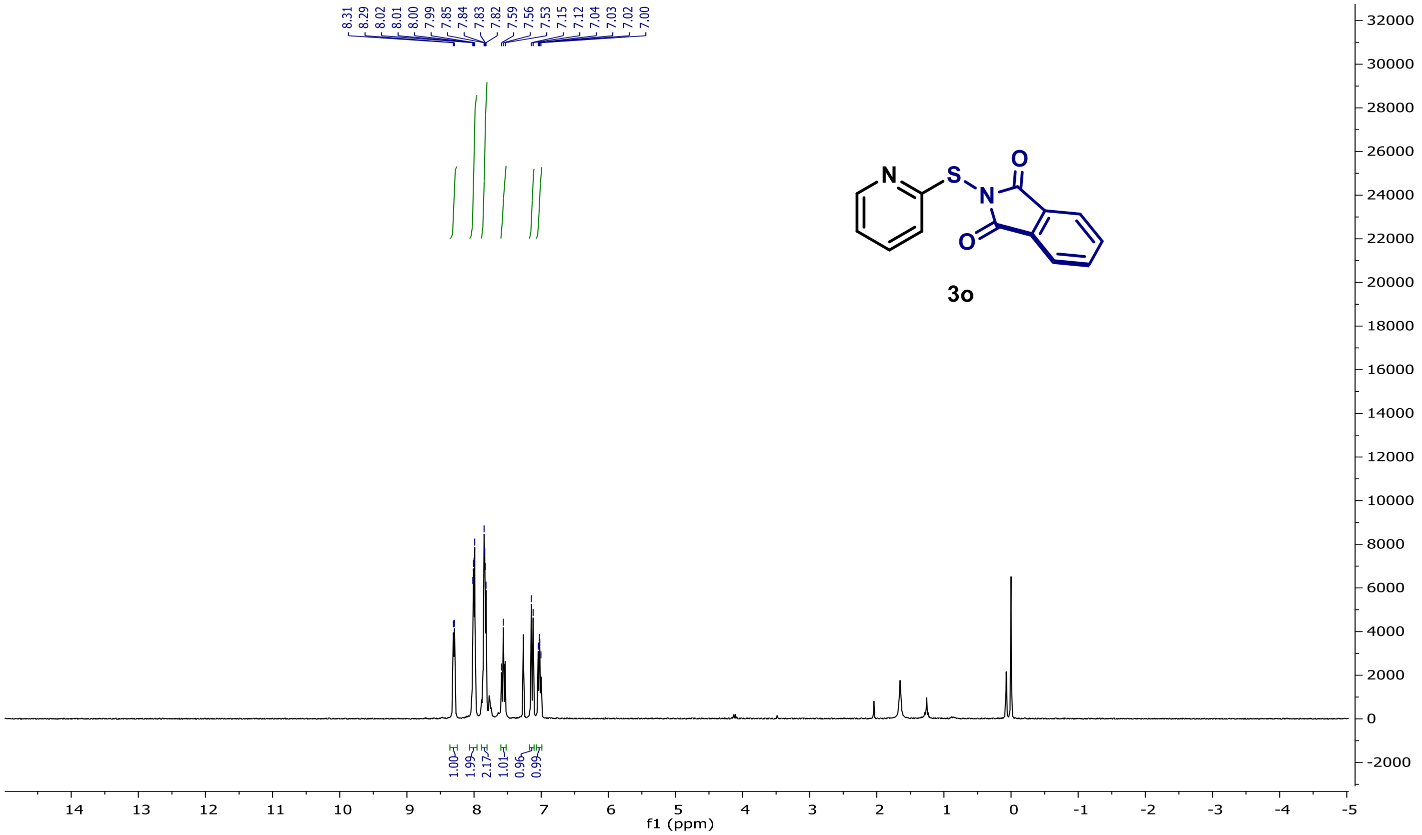

^{13}C Spectra (75 MHz, $CDCl_3$)

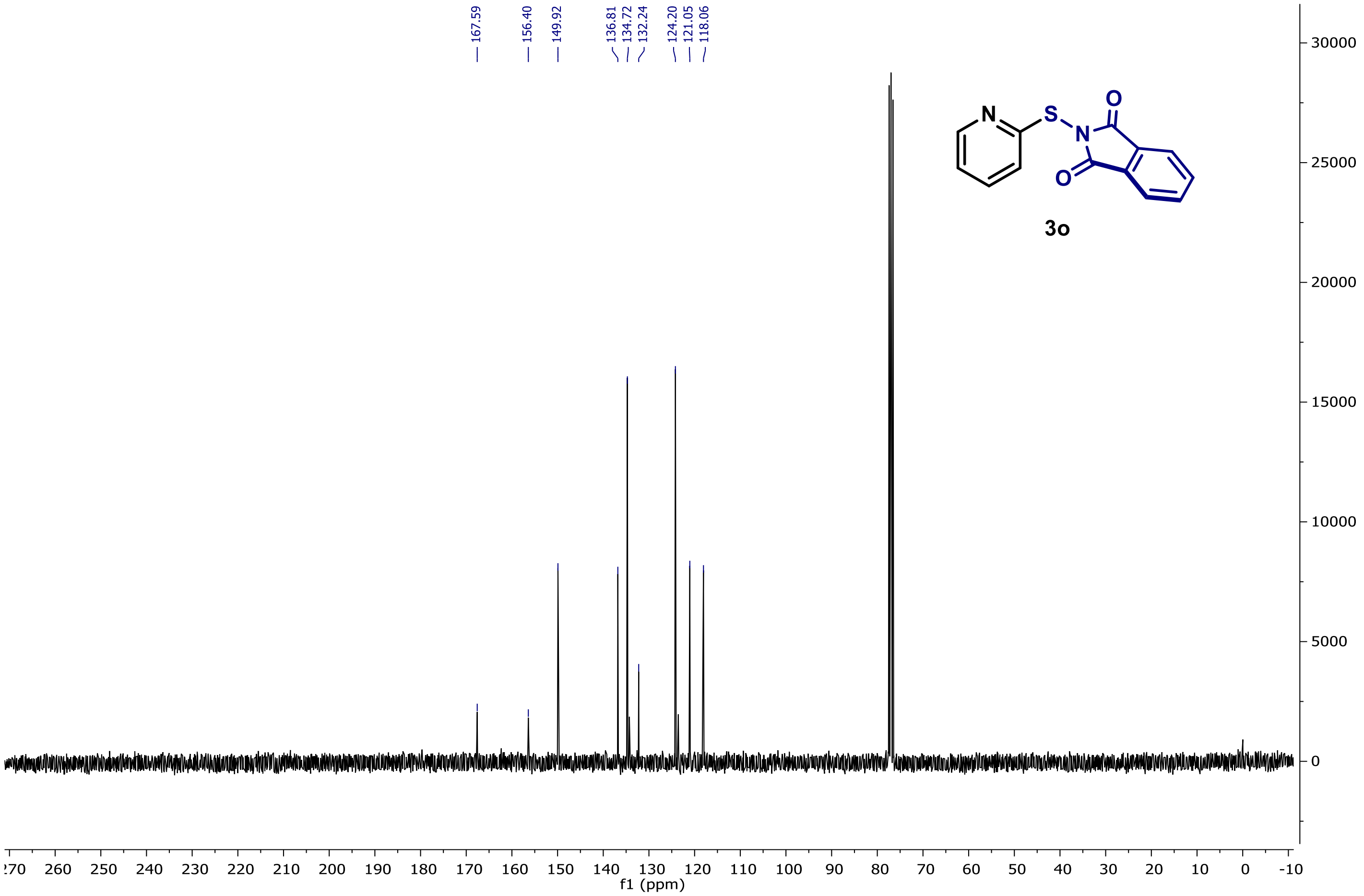

^{1}H Spectra (300 MHz, CDCl$_3$)

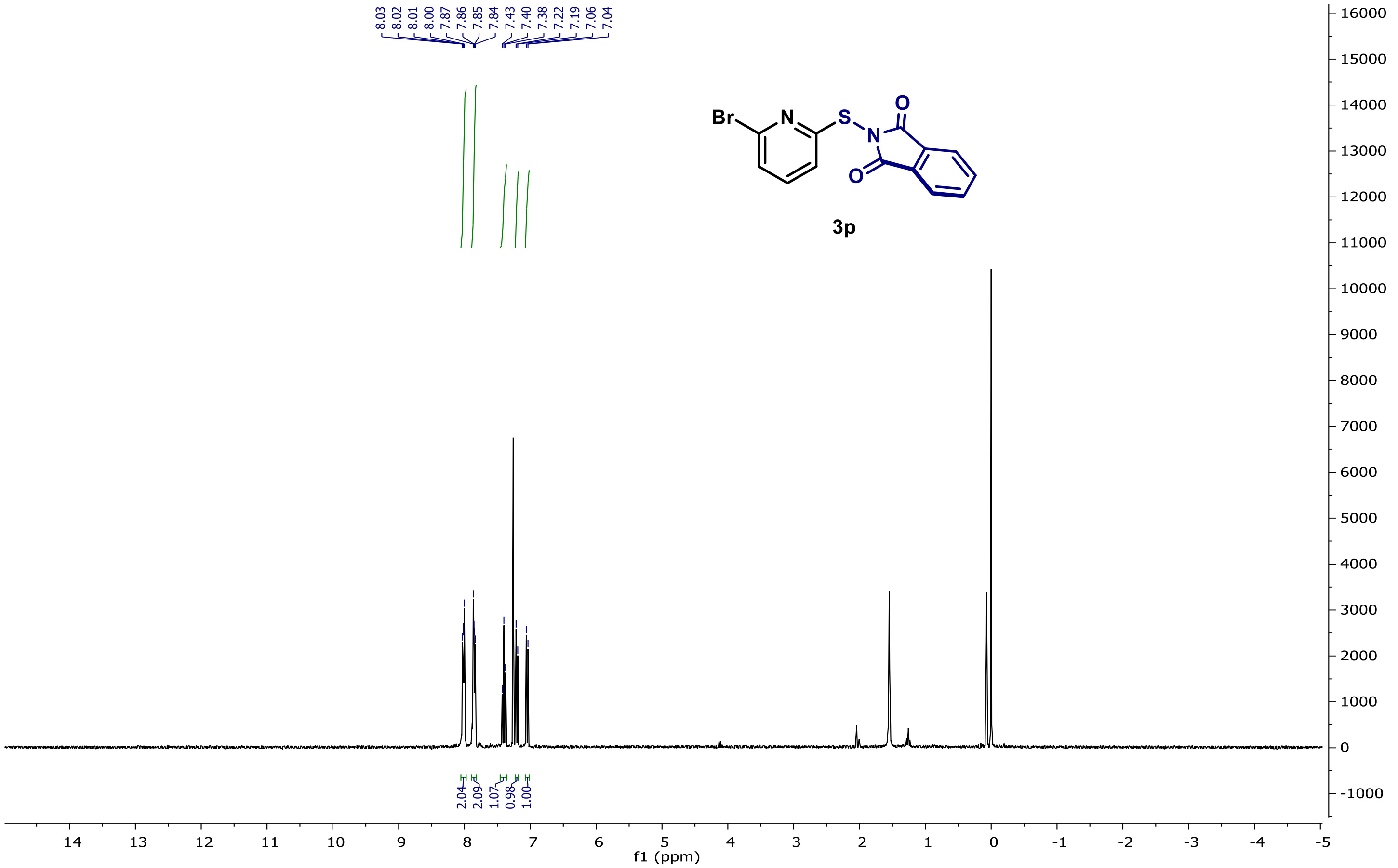

^{31}C Spectra (75 MHz, CDCl$_3$)

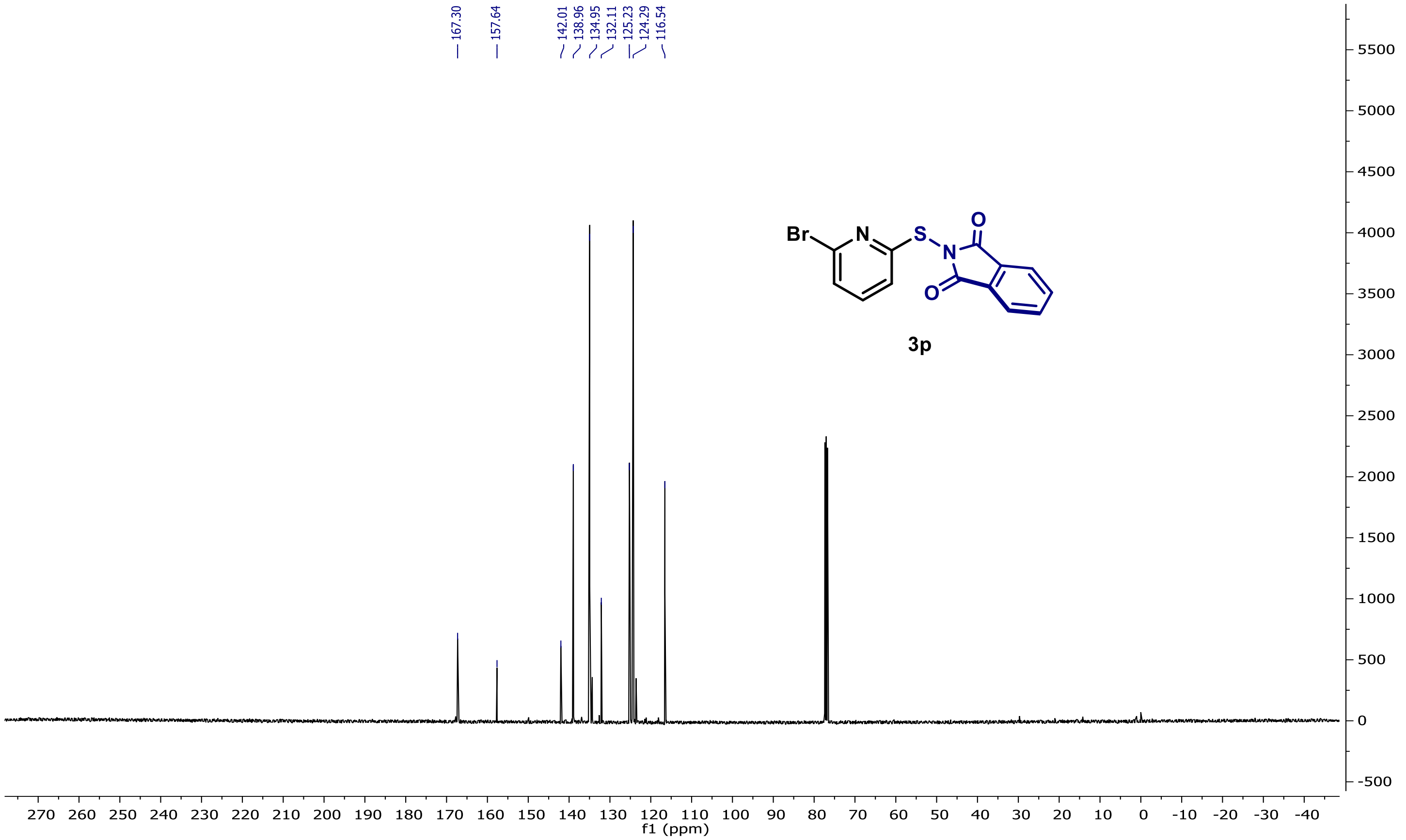

^{1}H Spectra (300 MHz, $CDCl_3$)

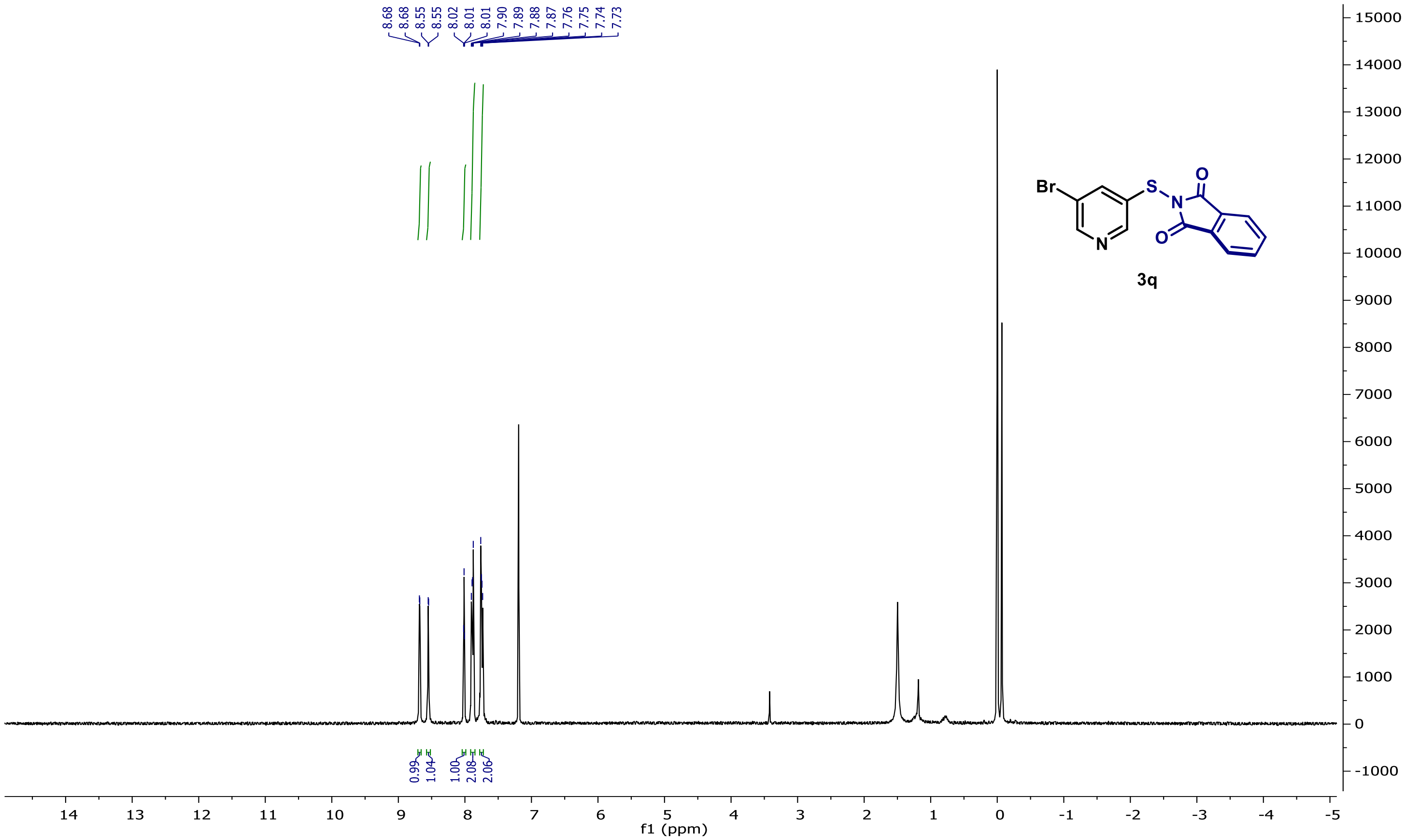

^{13}C Spectra (75 MHz, $CDCl_3$)

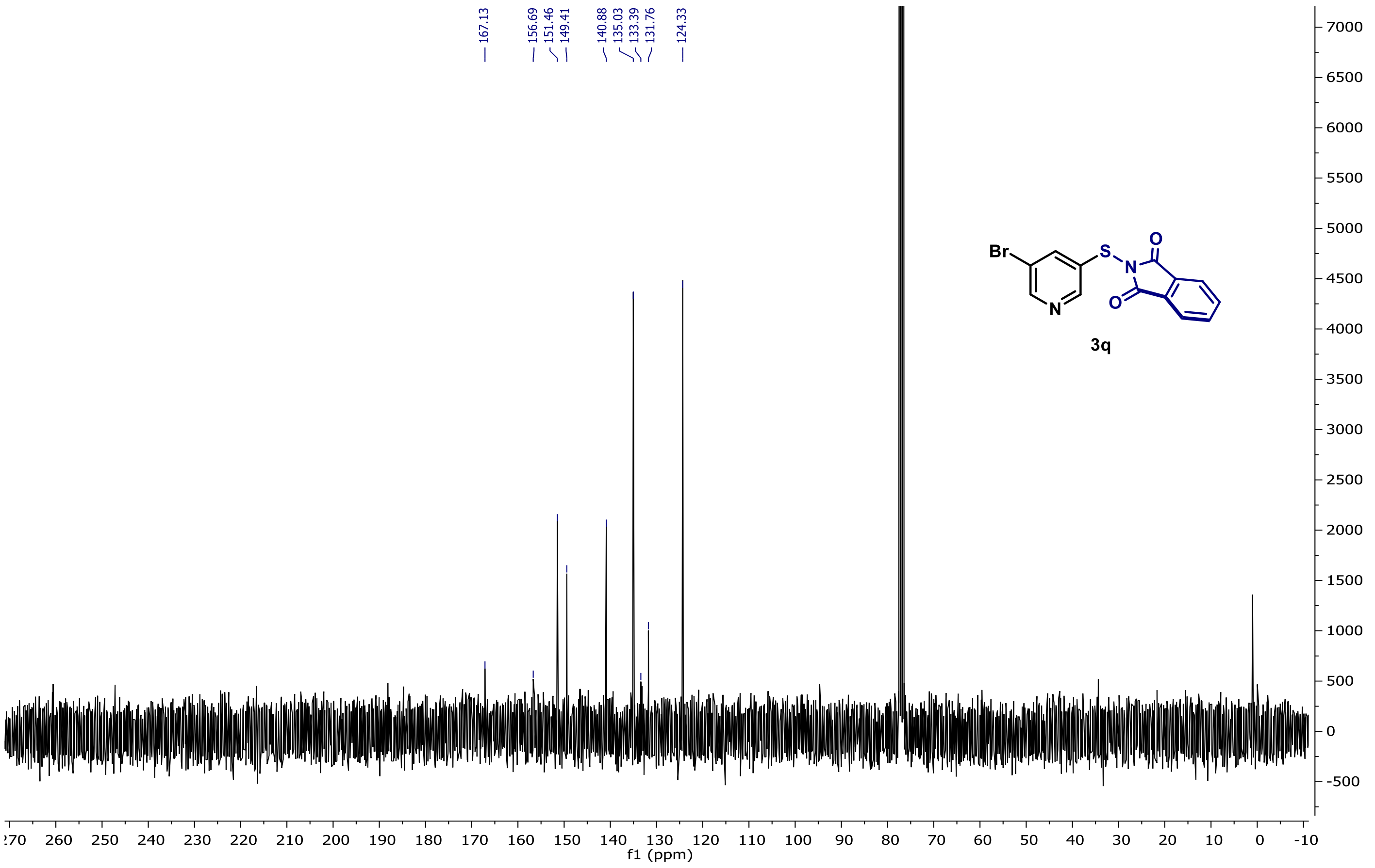

[1]H Spectra (300 MHz, $CDCl_3$)

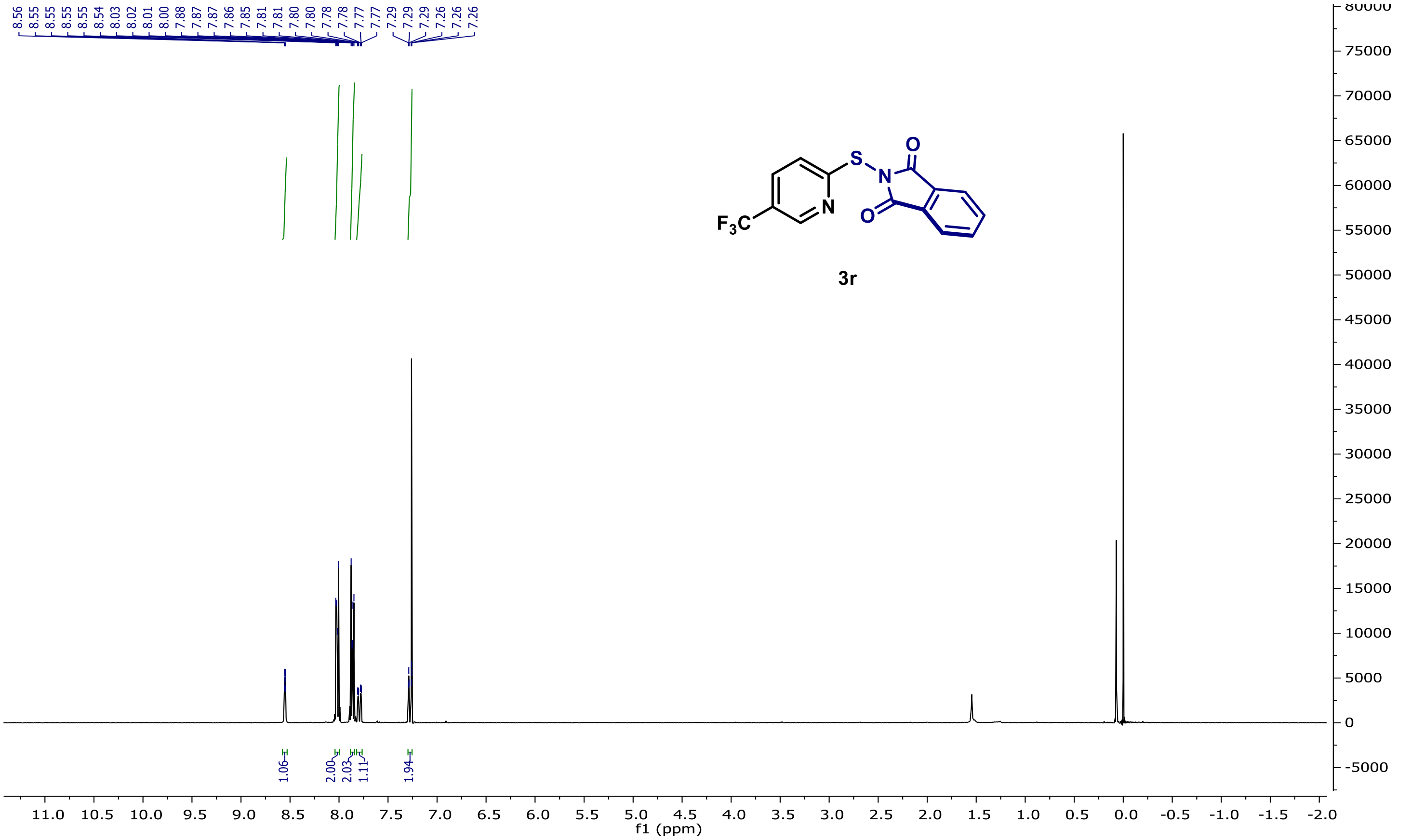

^{19}F{^{1}H} NMR (282 MHz, $CDCl_3$)

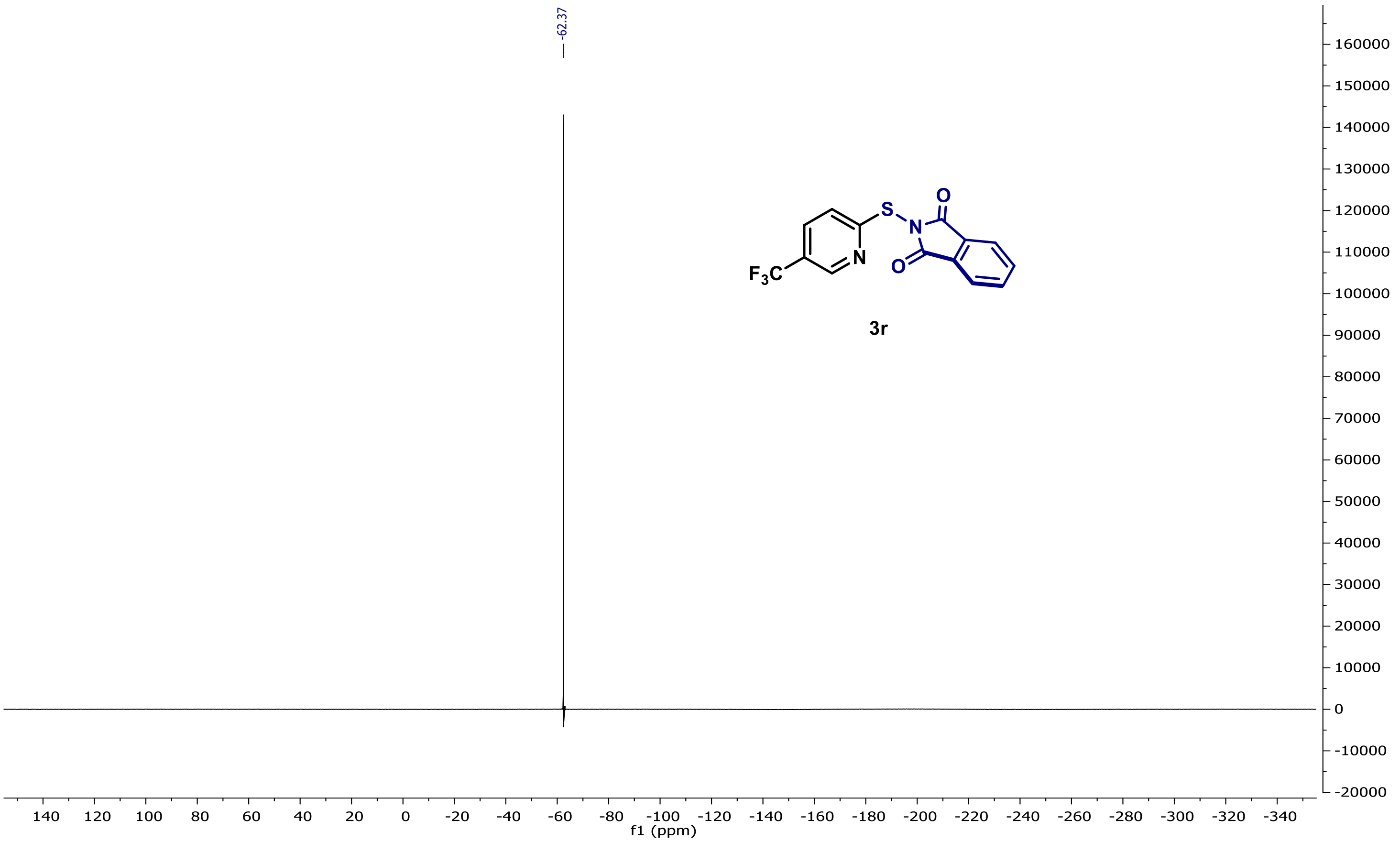

[31]C Spectra (75 MHz, $CDCl_3$)

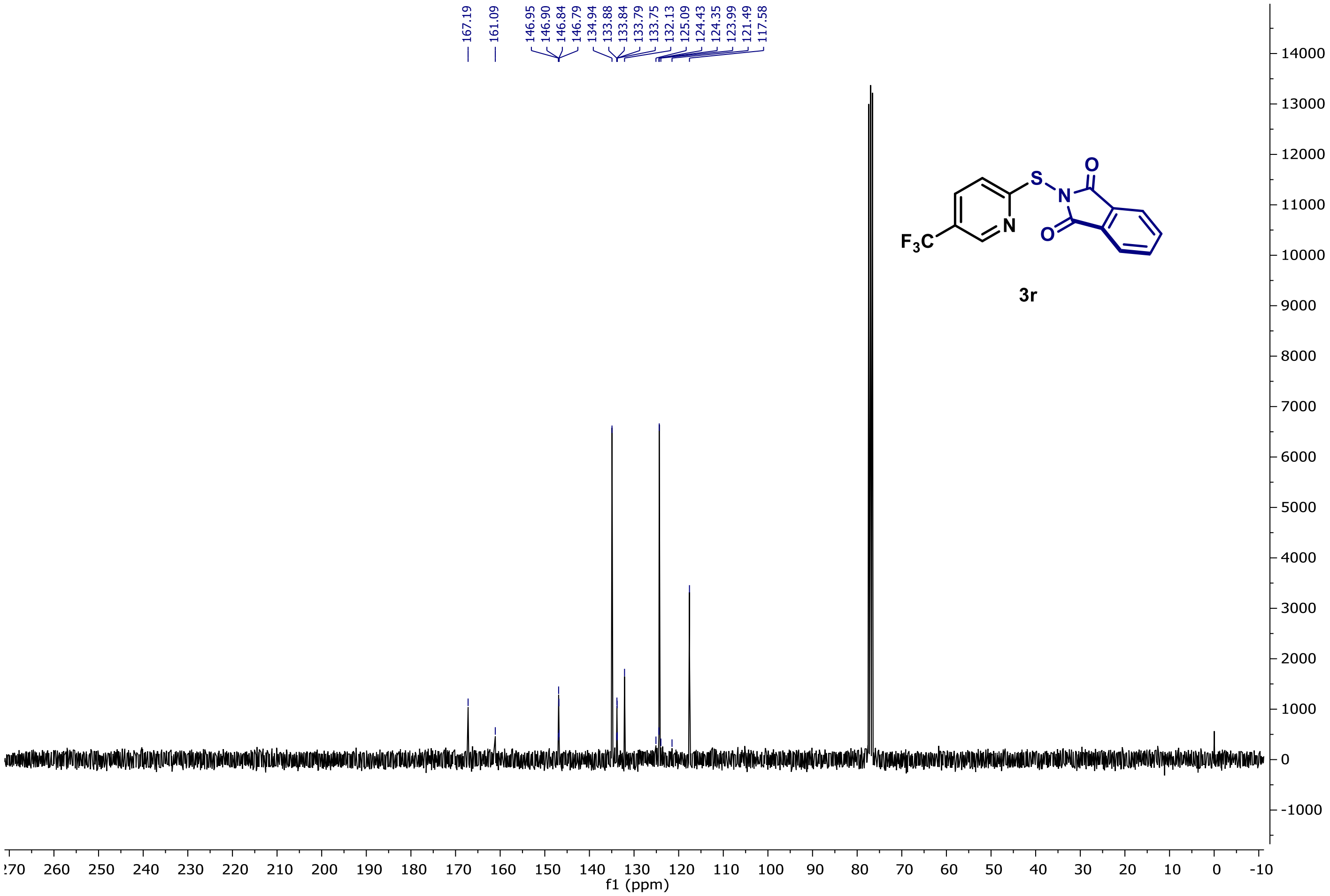

¹H Spectra (300 MHz, $CDCl_3$)

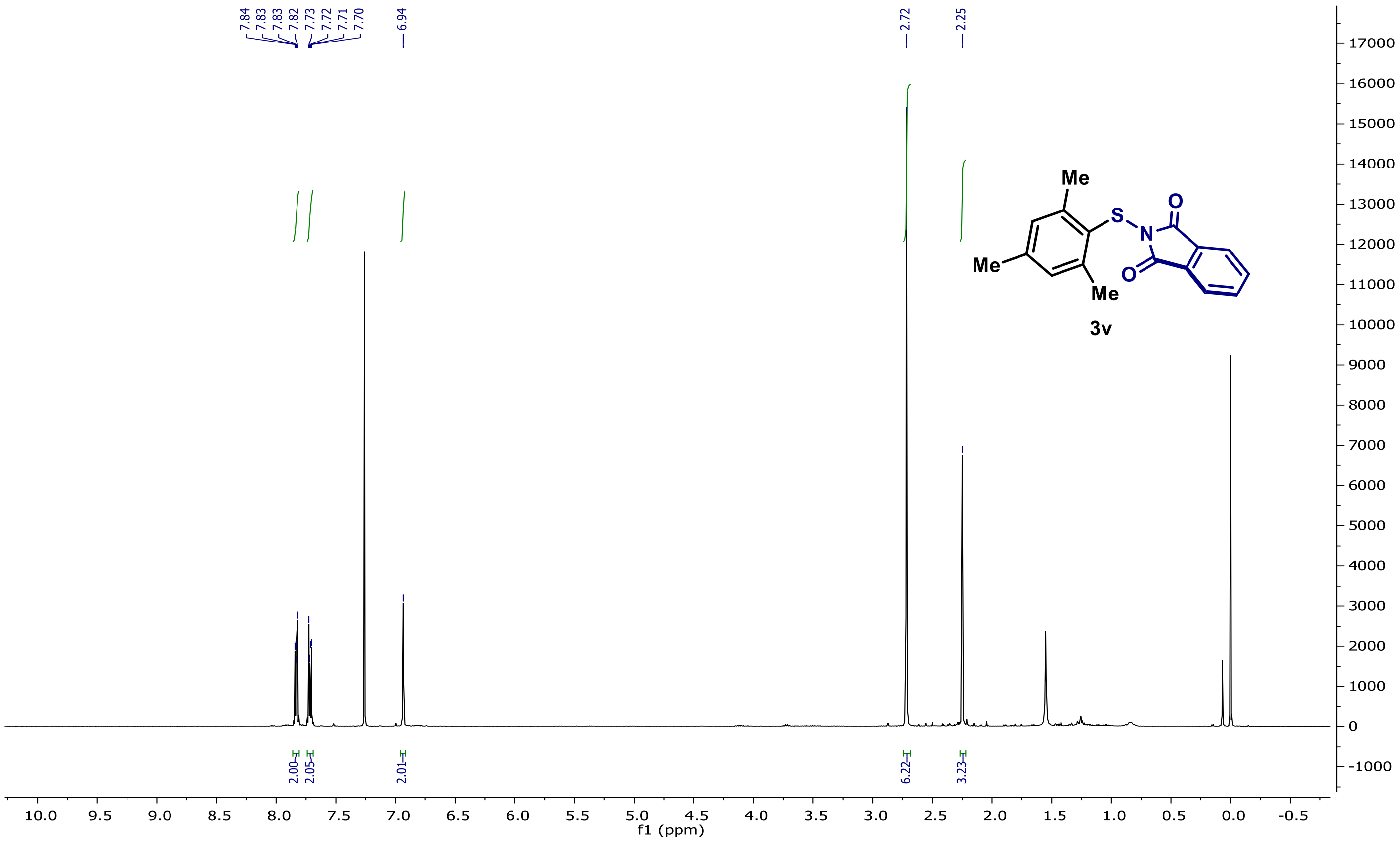

^{31}C Spectra (75 MHz, $CDCl_3$)

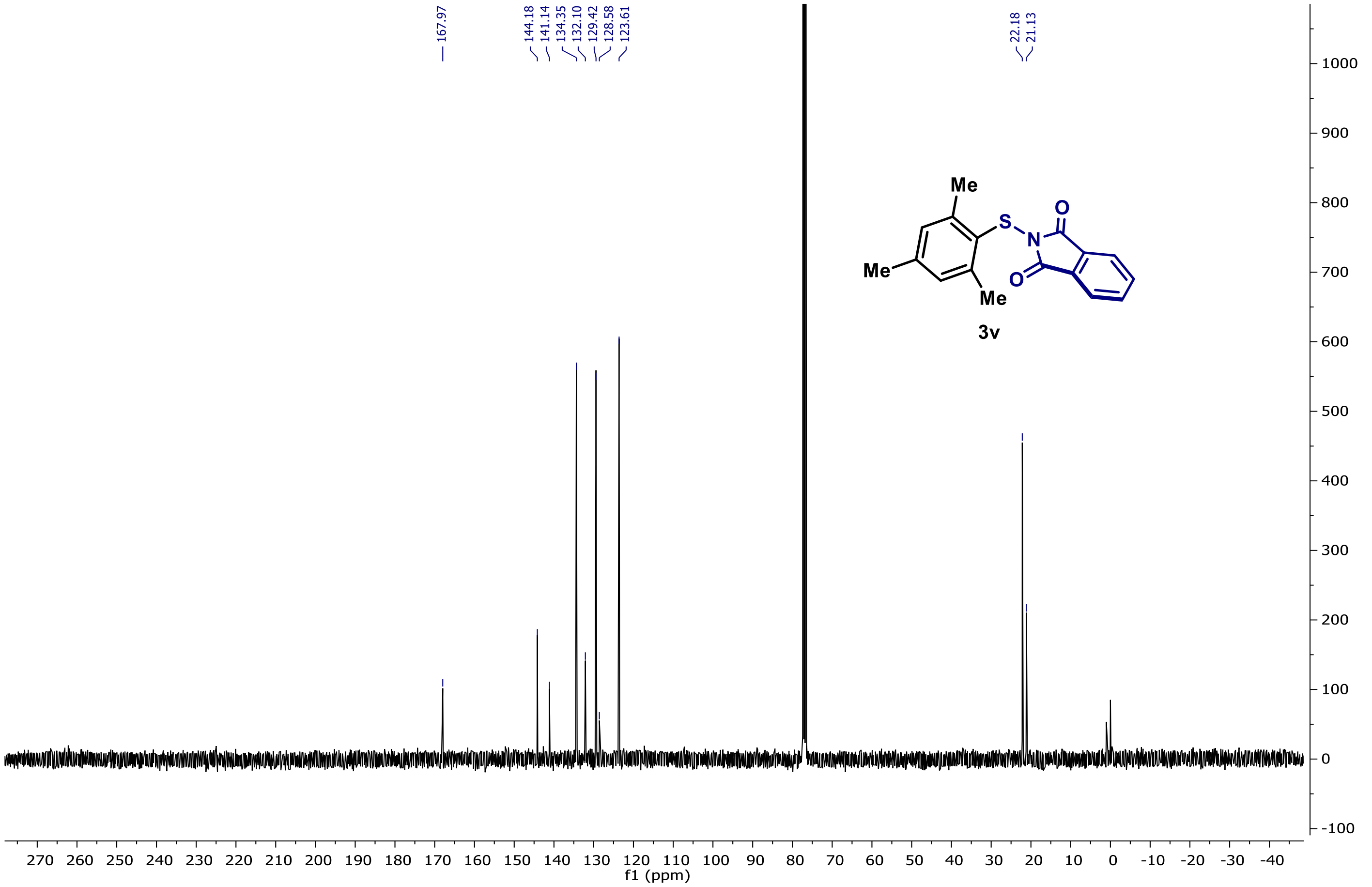

^{1}H Spectra (300 MHz, $CDCl_3$)

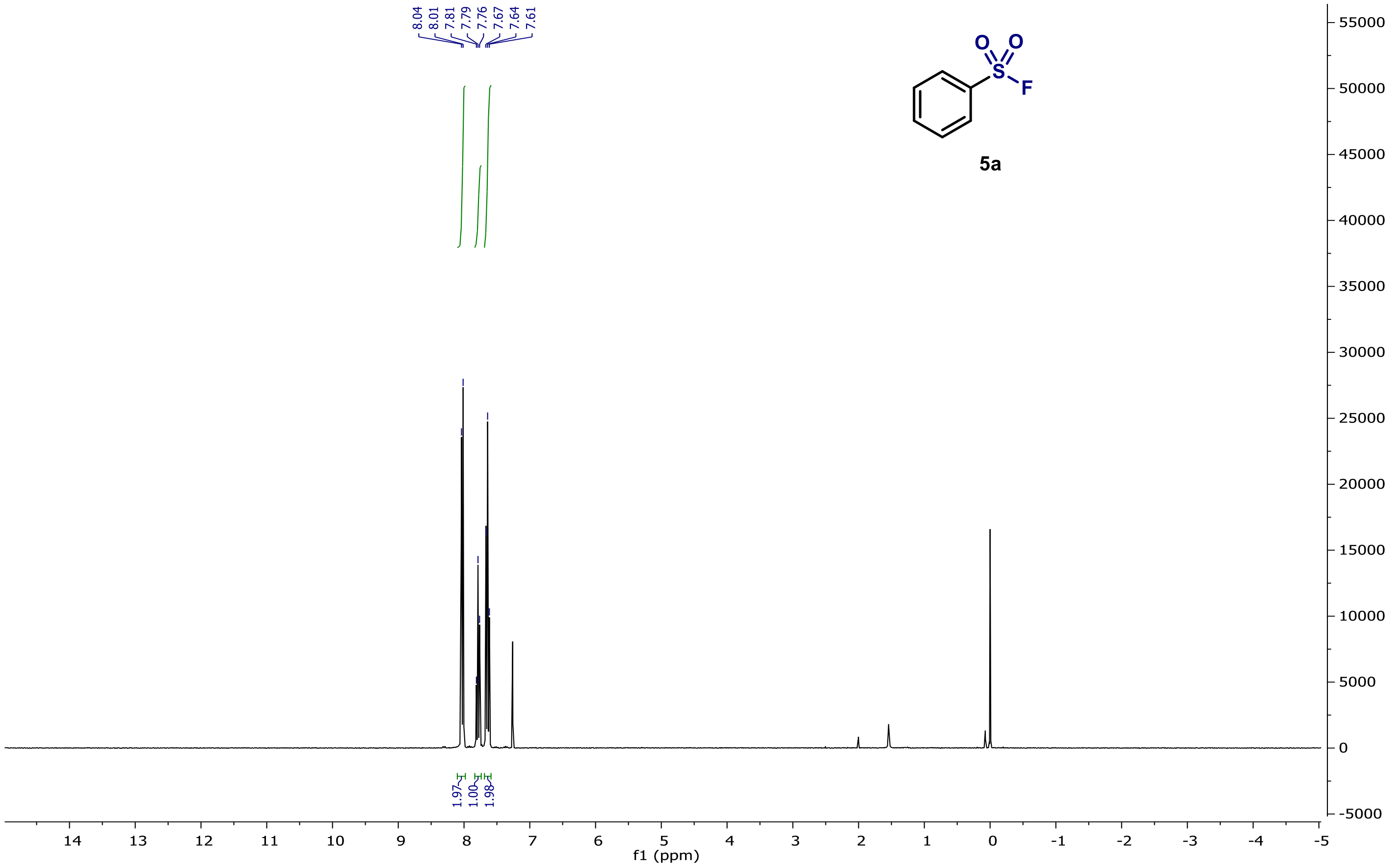

^{19}F{^{1}H} NMR (282 MHz, $CDCl_3$)

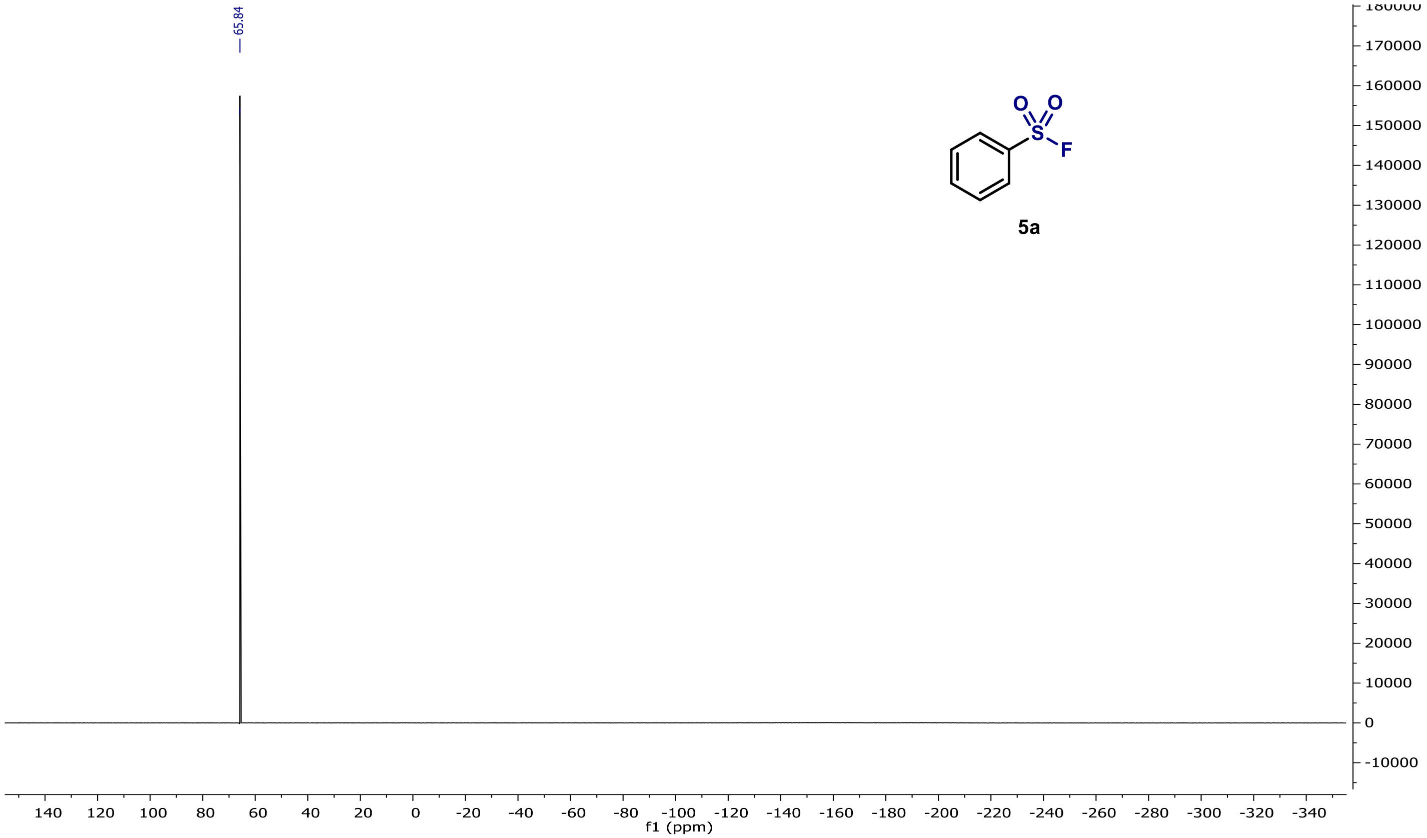

[13]C Spectra (75 MHz, $CDCl_3$)

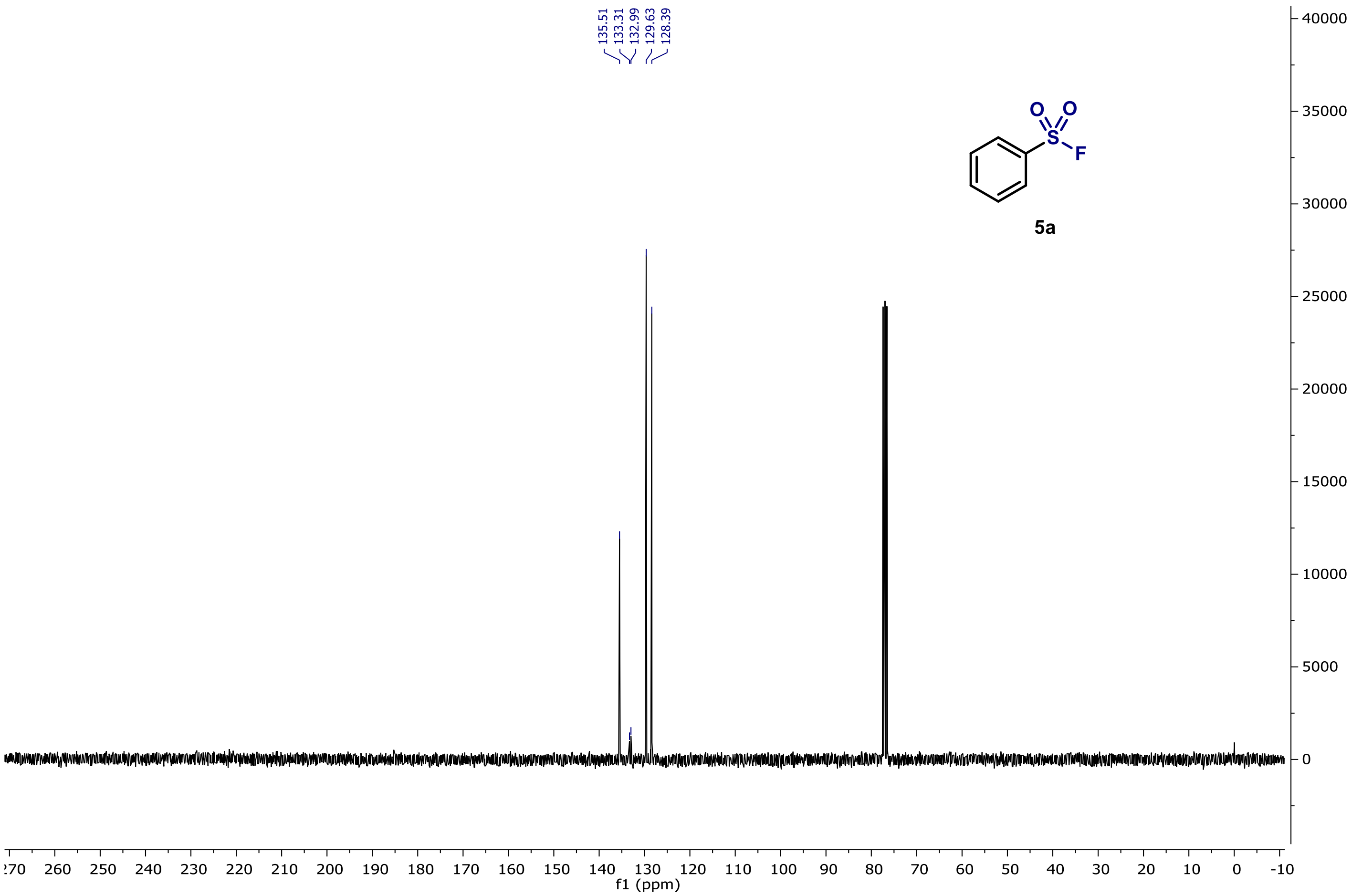

^{1}H Spectra (300 MHz, $CDCl_3$)

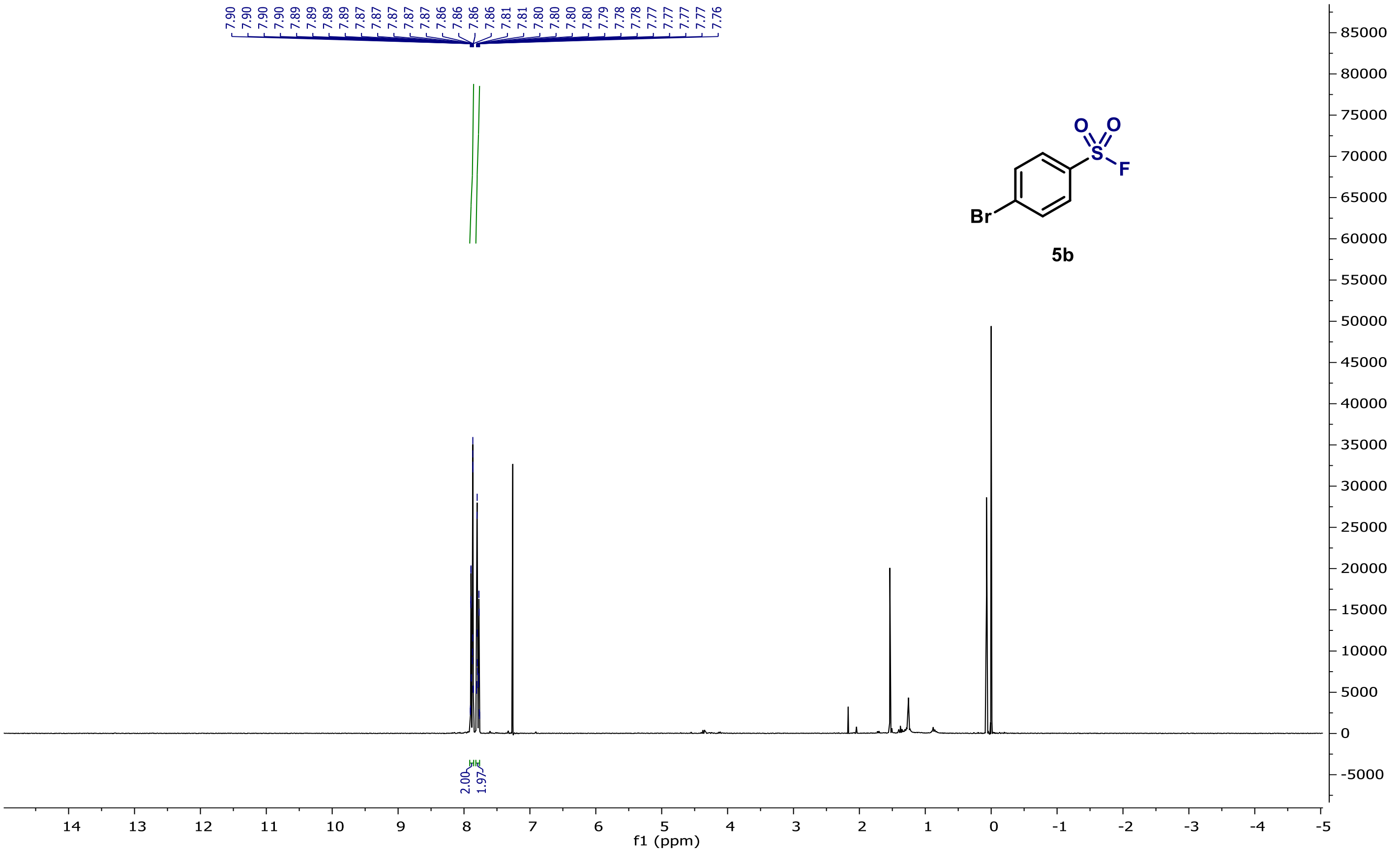

^{19}F{^{1}H} NMR (282 MHz, $CDCl_3$)

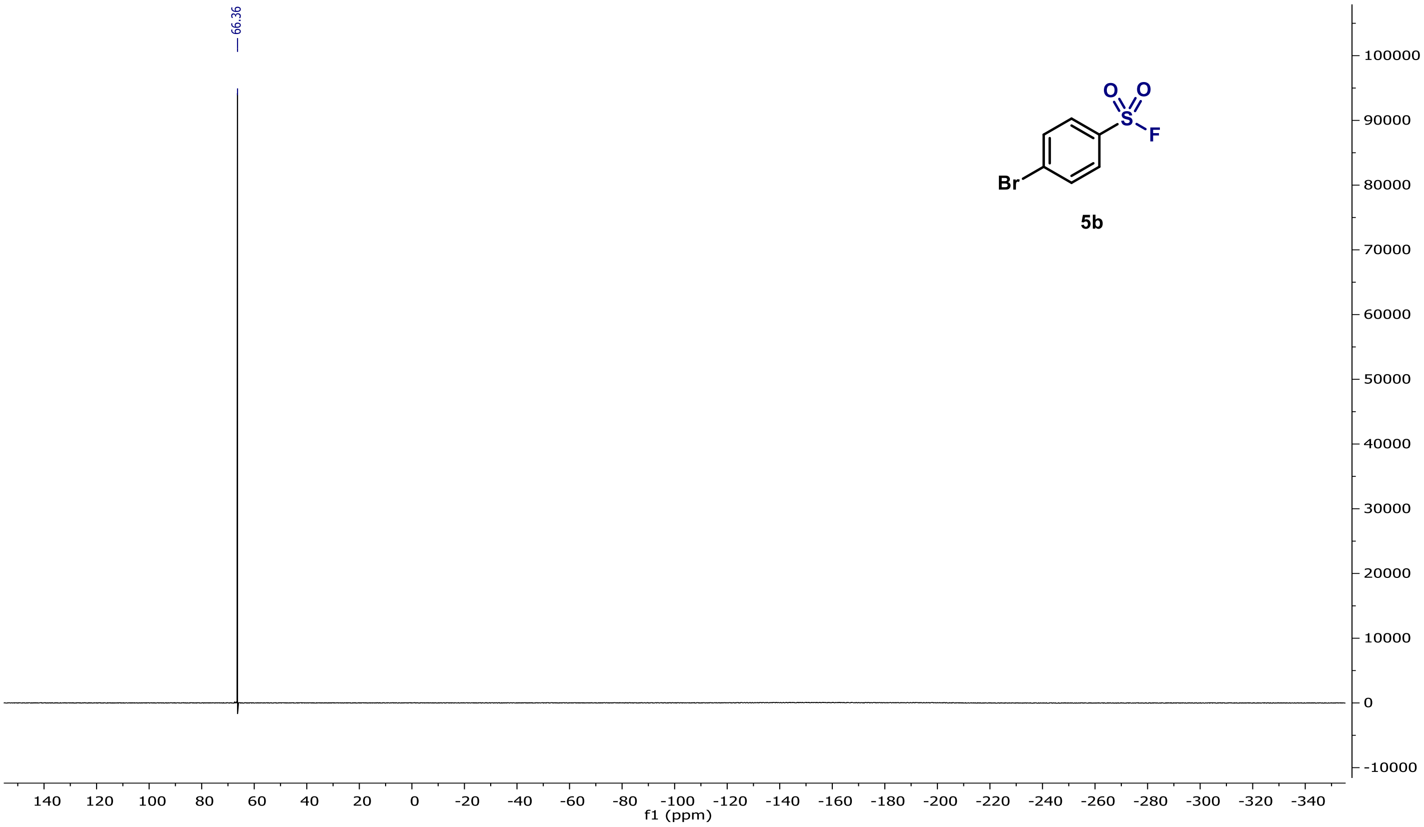

[13]C Spectra (75 MHz, $CDCl_3$)

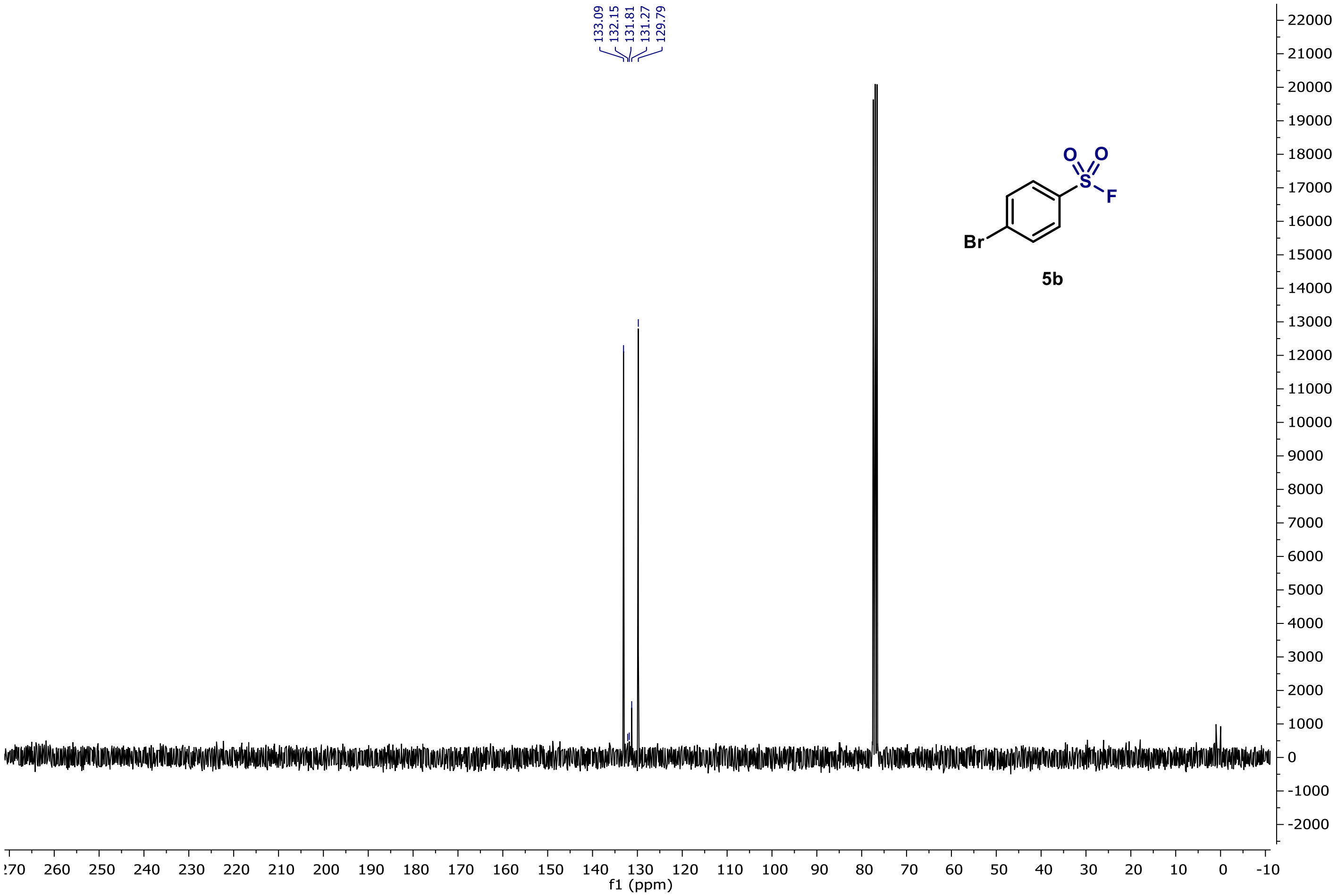

^{1}H Spectra (300 MHz, $CDCl_3$)

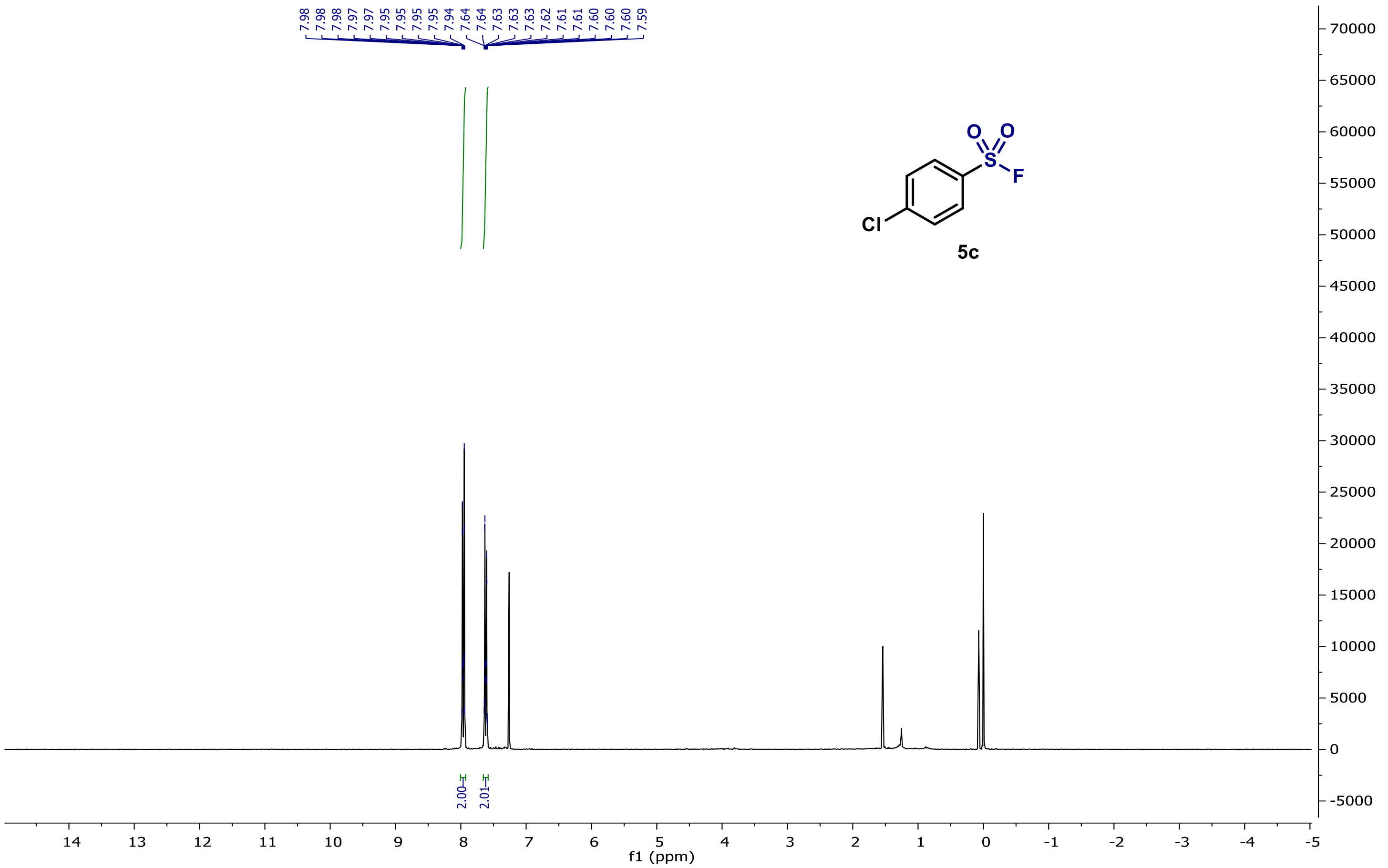

$^{19}F\{^{1}H\}$ NMR (282 MHz, $CDCl_3$)

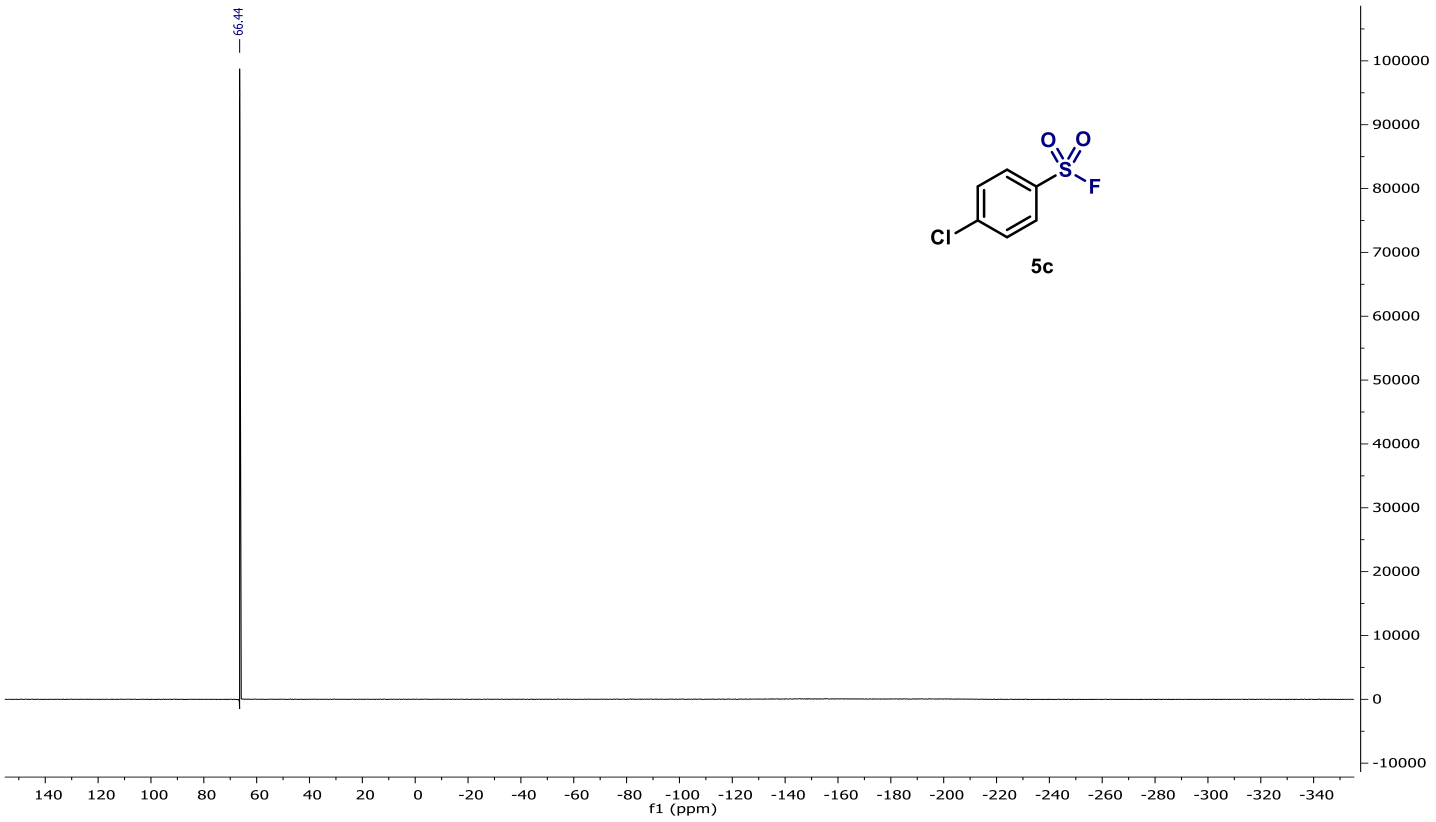

[13]C Spectra (75 MHz, $CDCl_3$)

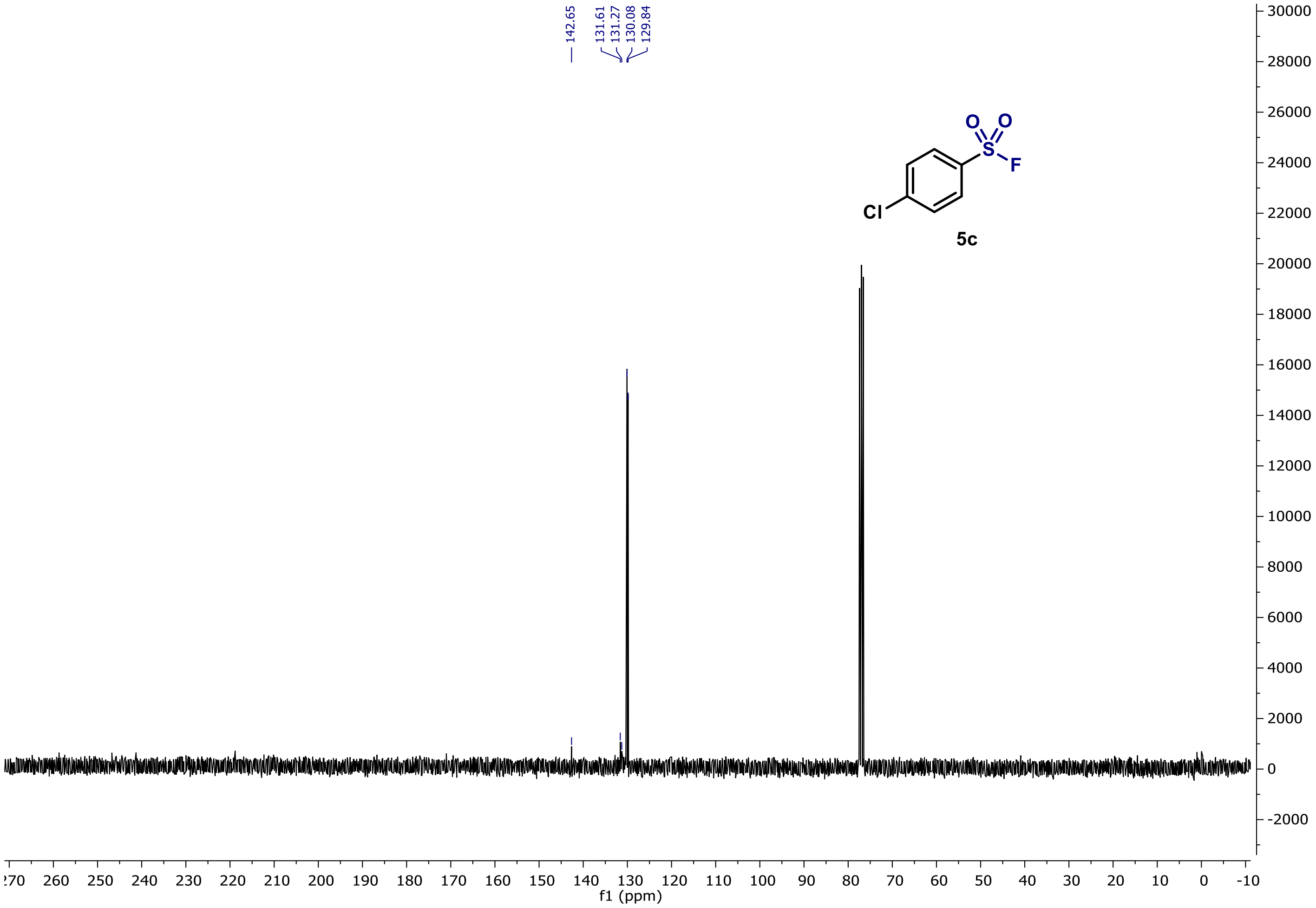

^{1}H Spectra (300 MHz, $CDCl_3$)

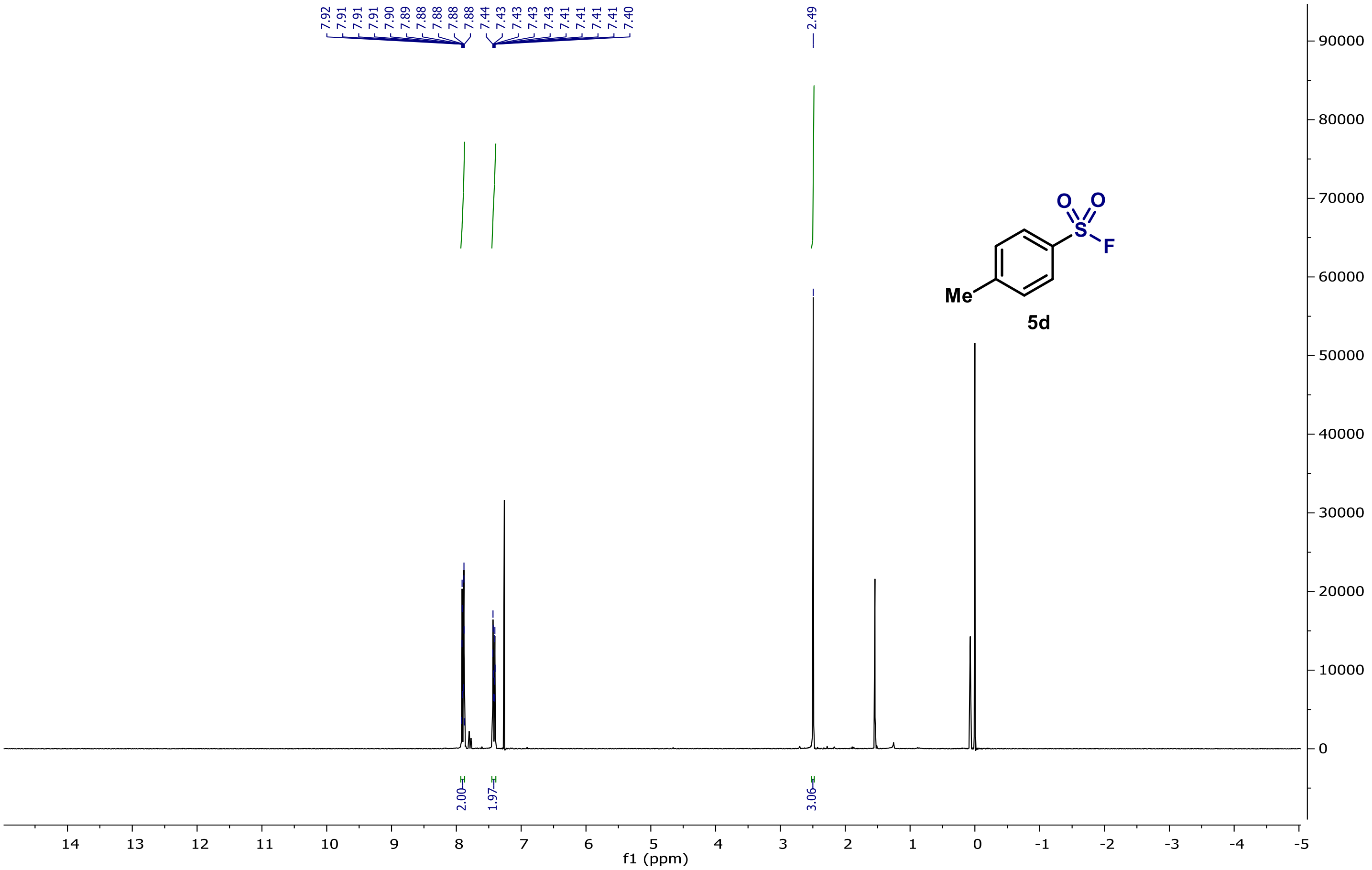

^{19}F{^{1}H} NMR (282 MHz, $CDCl_3$)

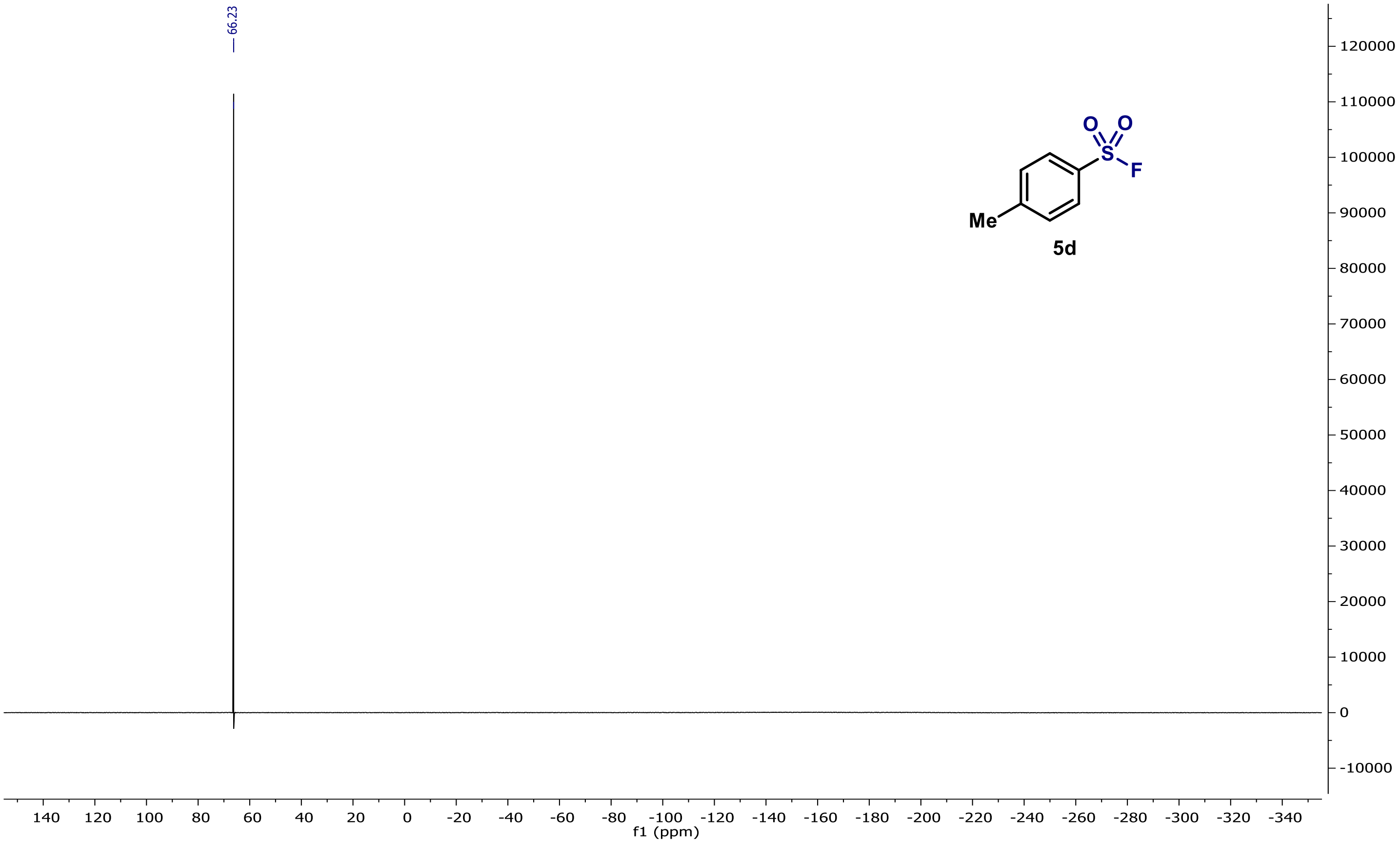

^{13}**C Spectra** (75 MHz, $CDCl_3$)

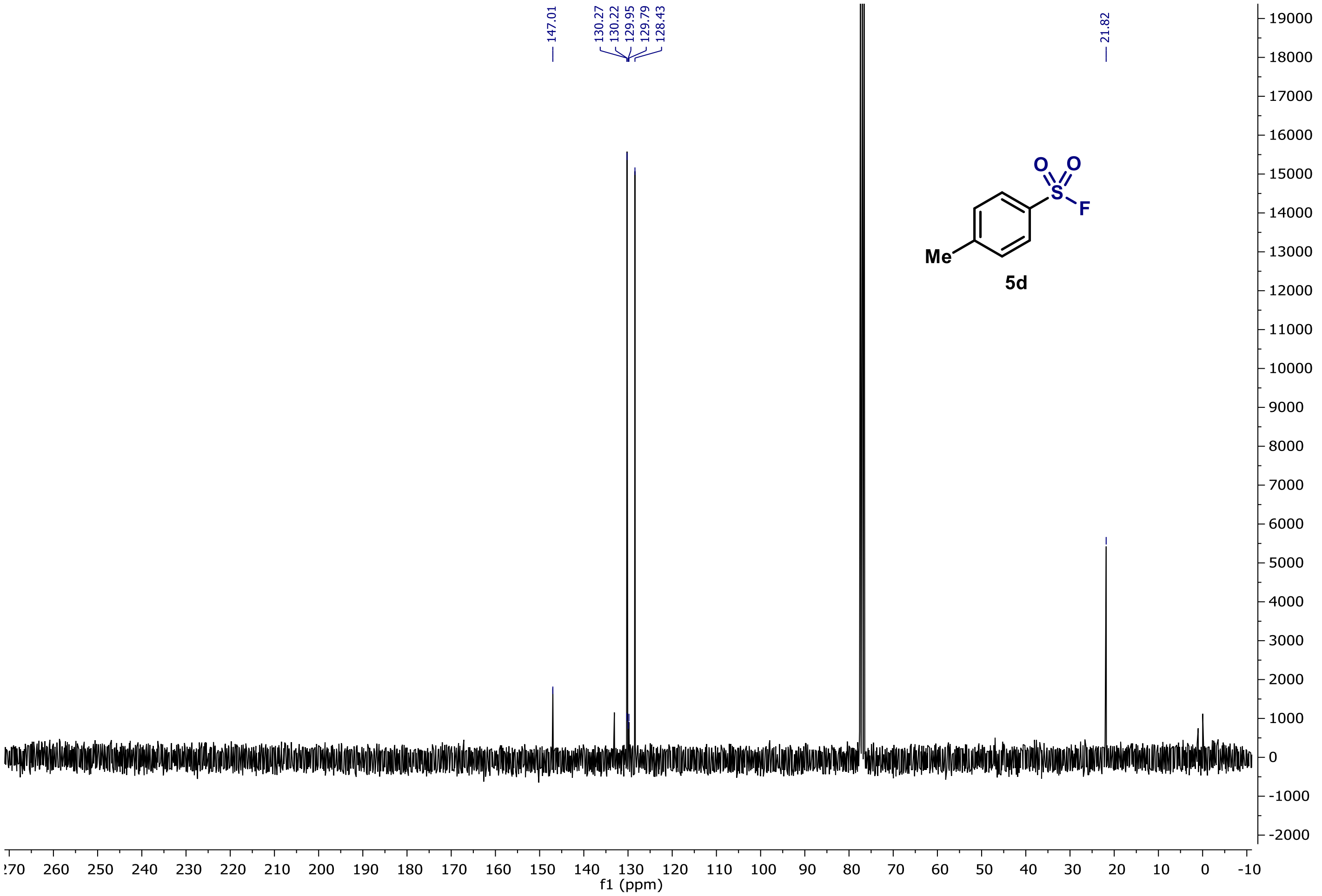

^{1}H Spectra (300 MHz, $CDCl_3$)

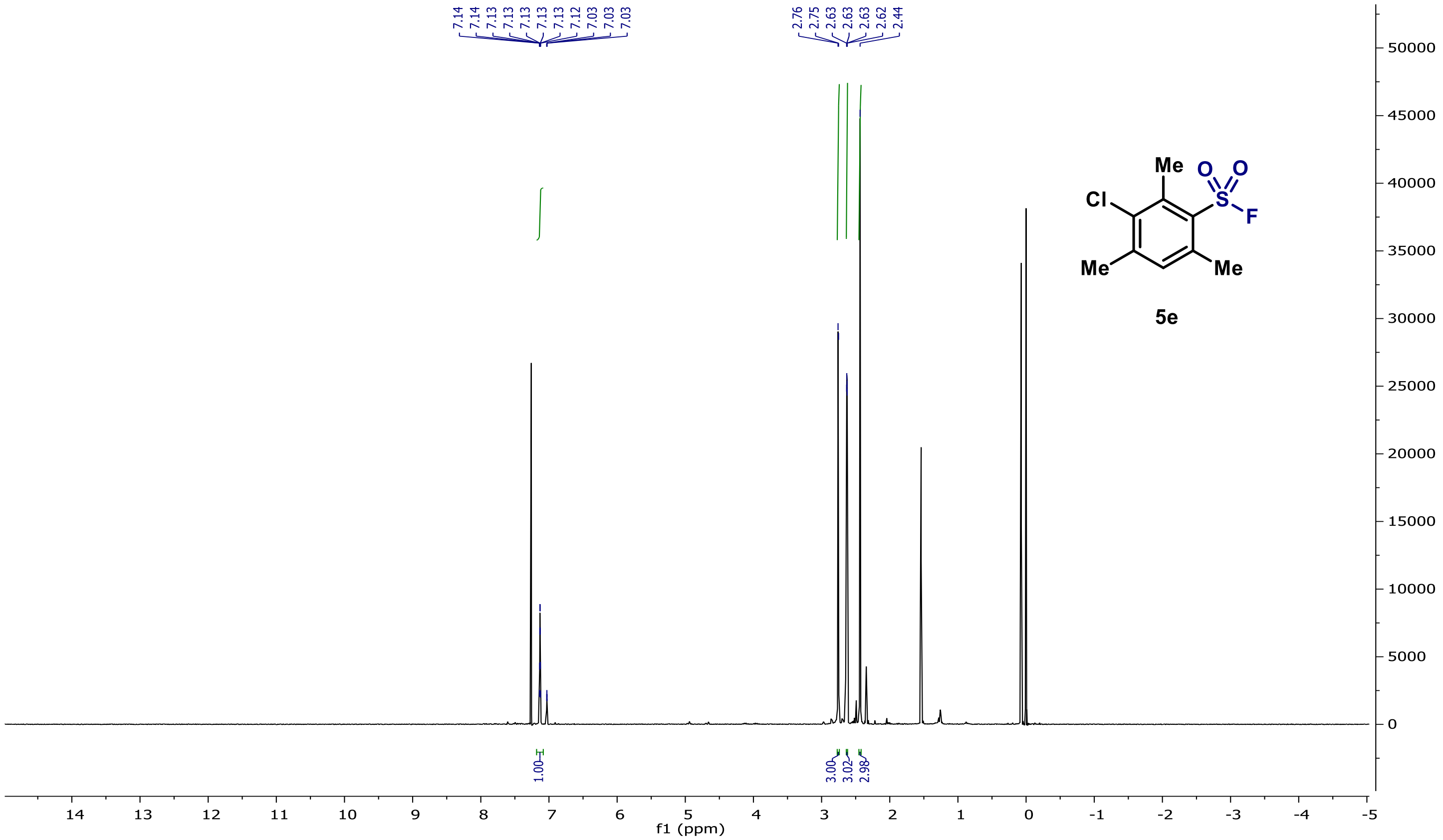

^{19}F{^{1}H} NMR (282 MHz, $CDCl_3$)

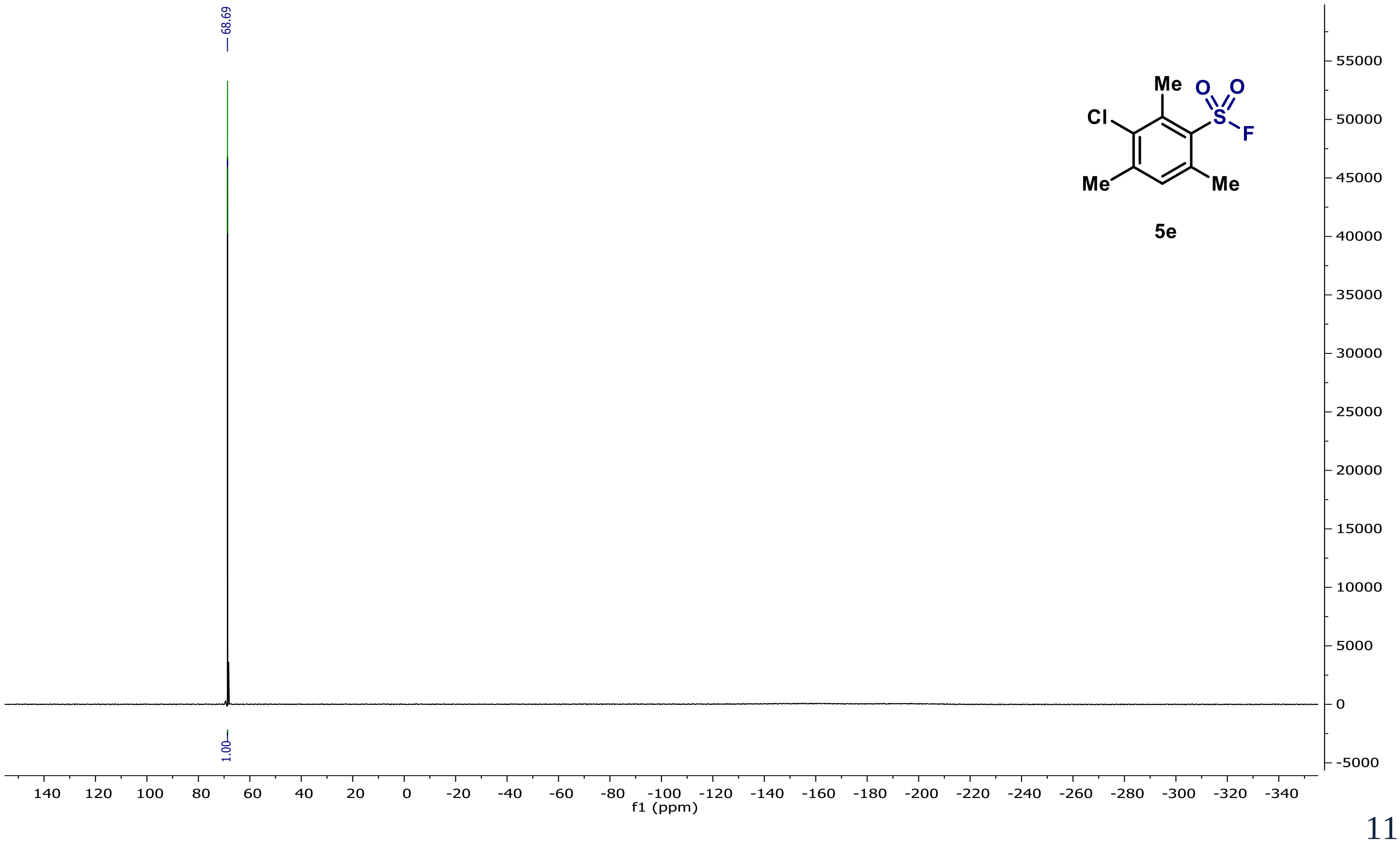

^{13}C Spectra (75 MHz, $CDCl_3$)

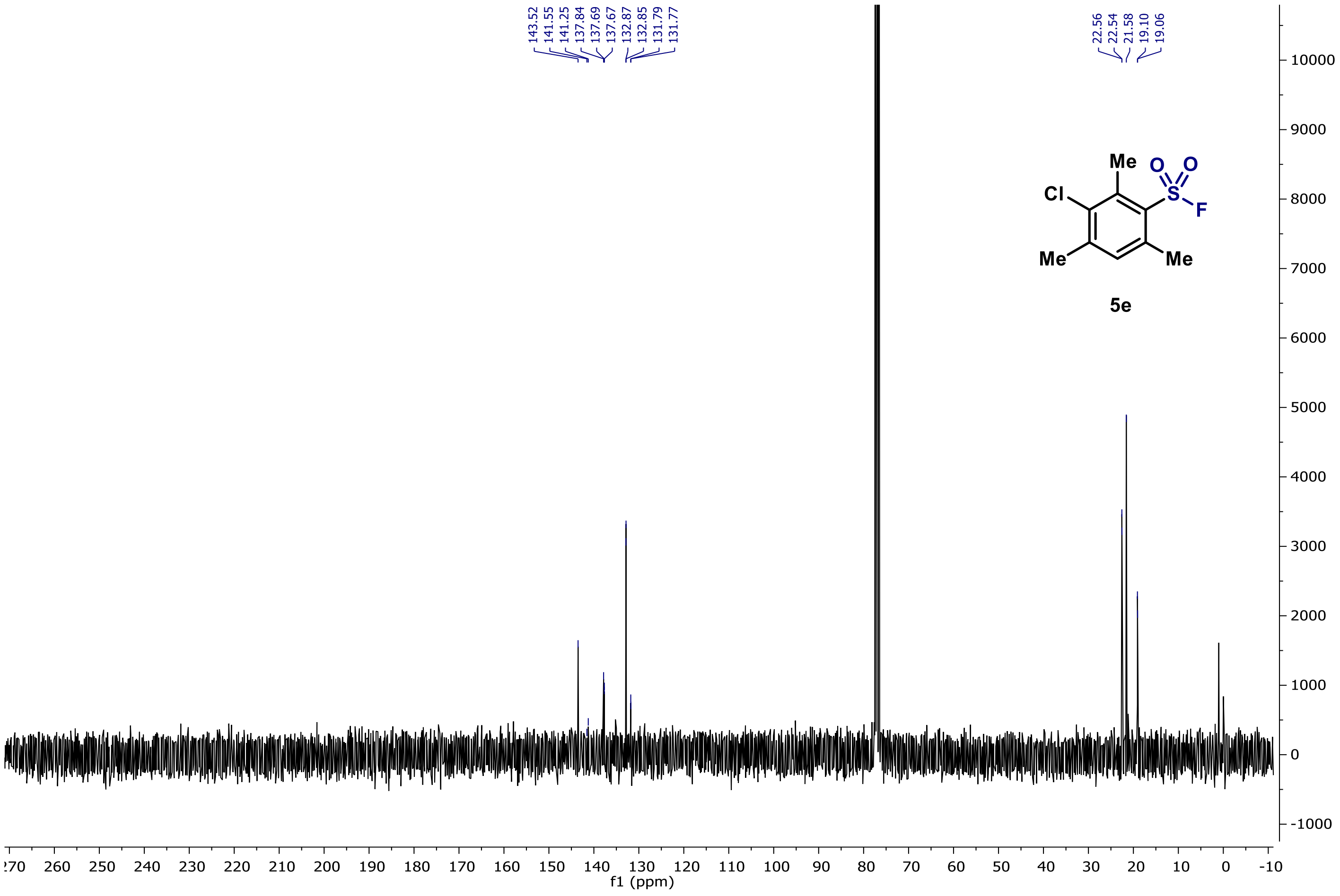

^{1}H Spectra (300 MHz, $CDCl_3$)

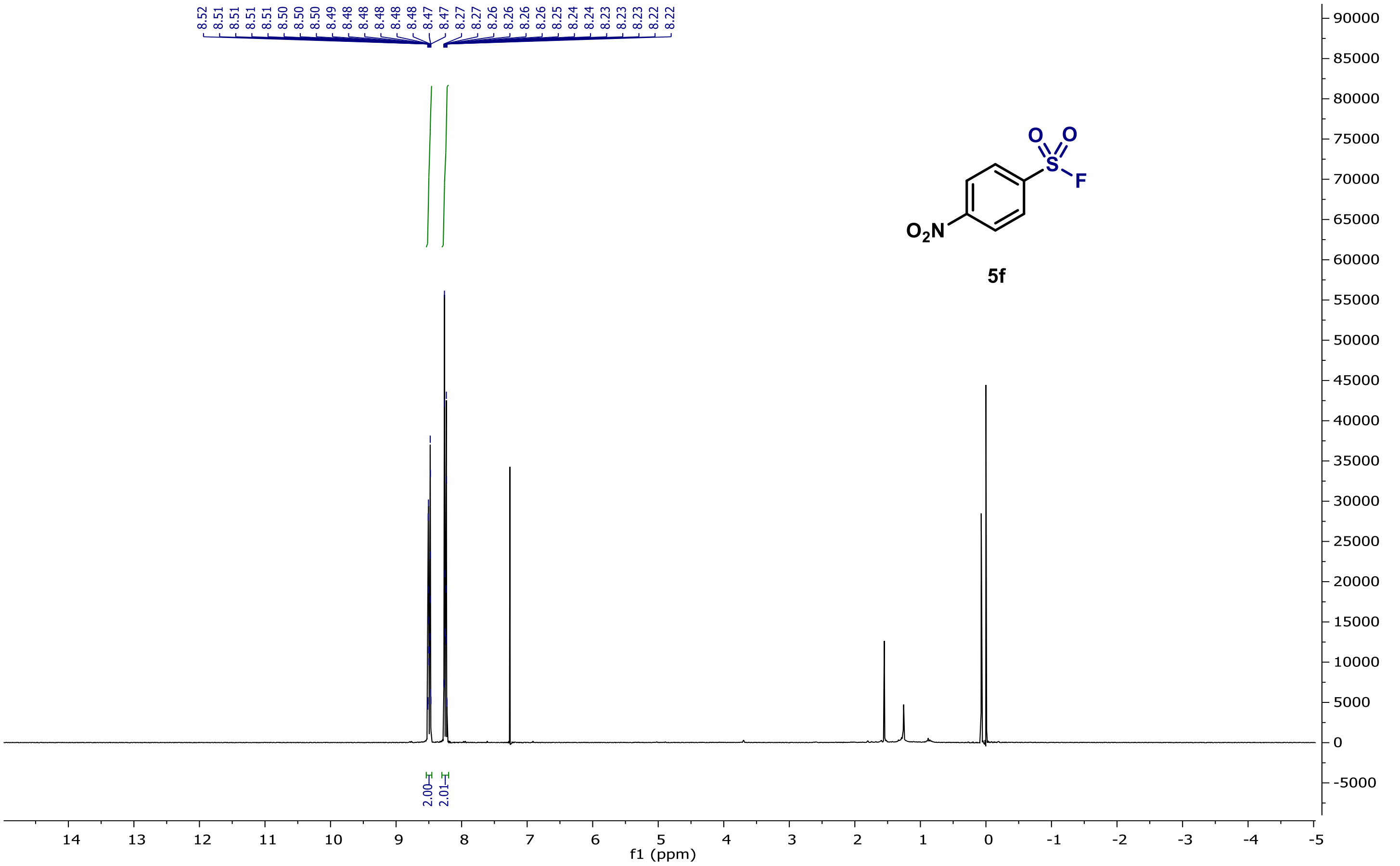

^{19}F{^{1}H} NMR (282 MHz, $CDCl_3$)

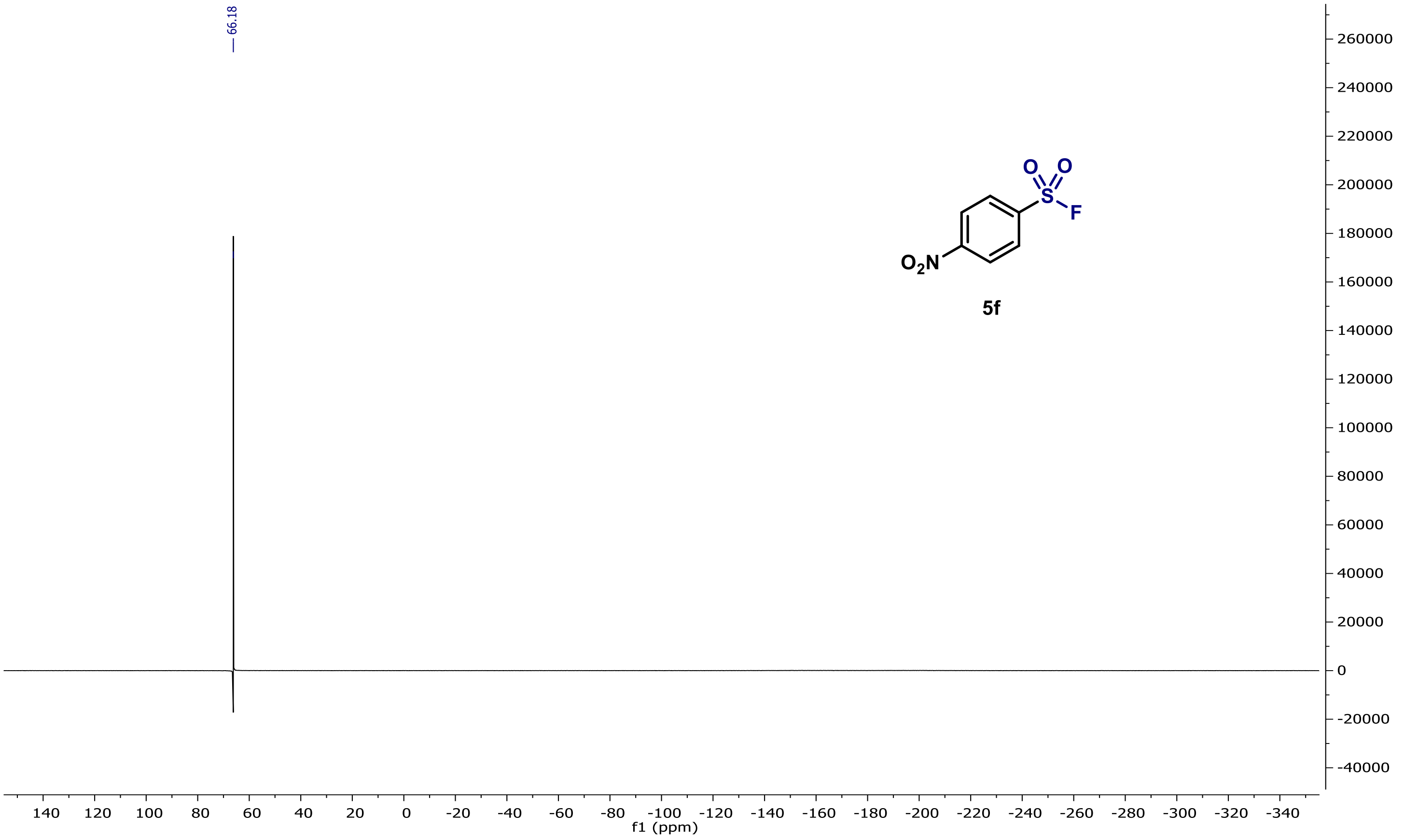

^{13}C Spectra (75 MHz, $CDCl_3$)

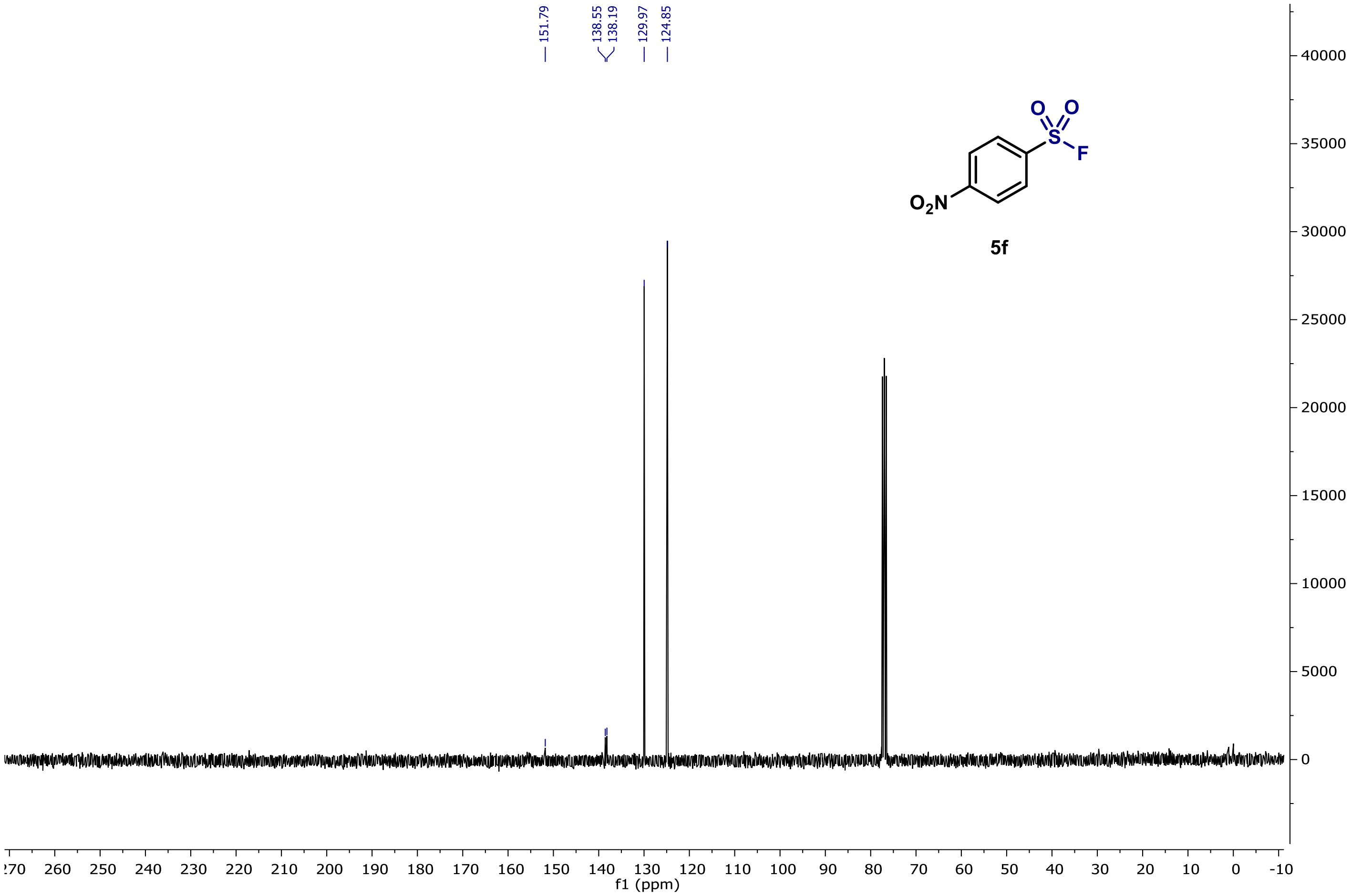

^{1}H Spectra (300 MHz, $CDCl_3$)

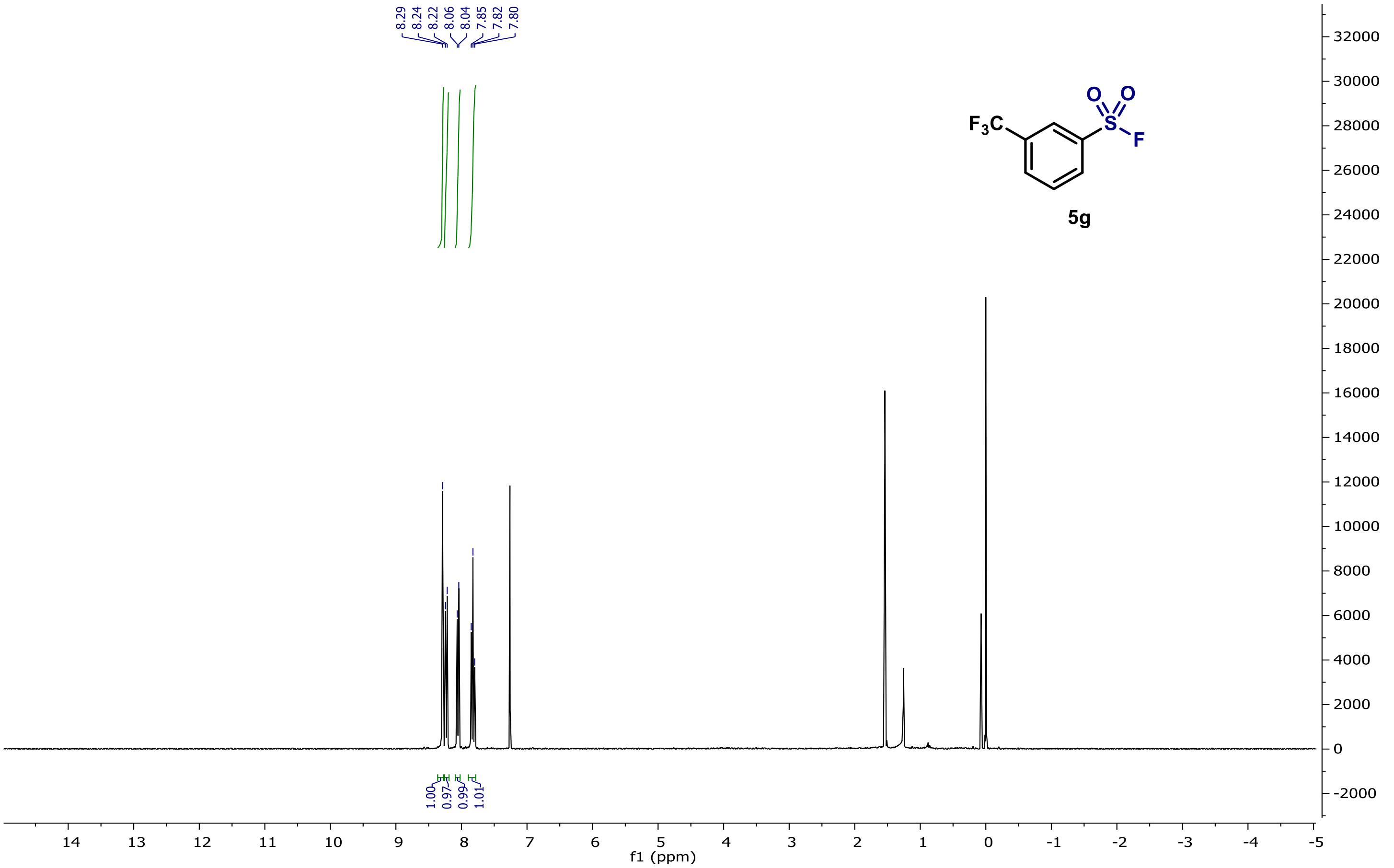

^{19}F{^{1}H} NMR (282 MHz, $CDCl_3$)

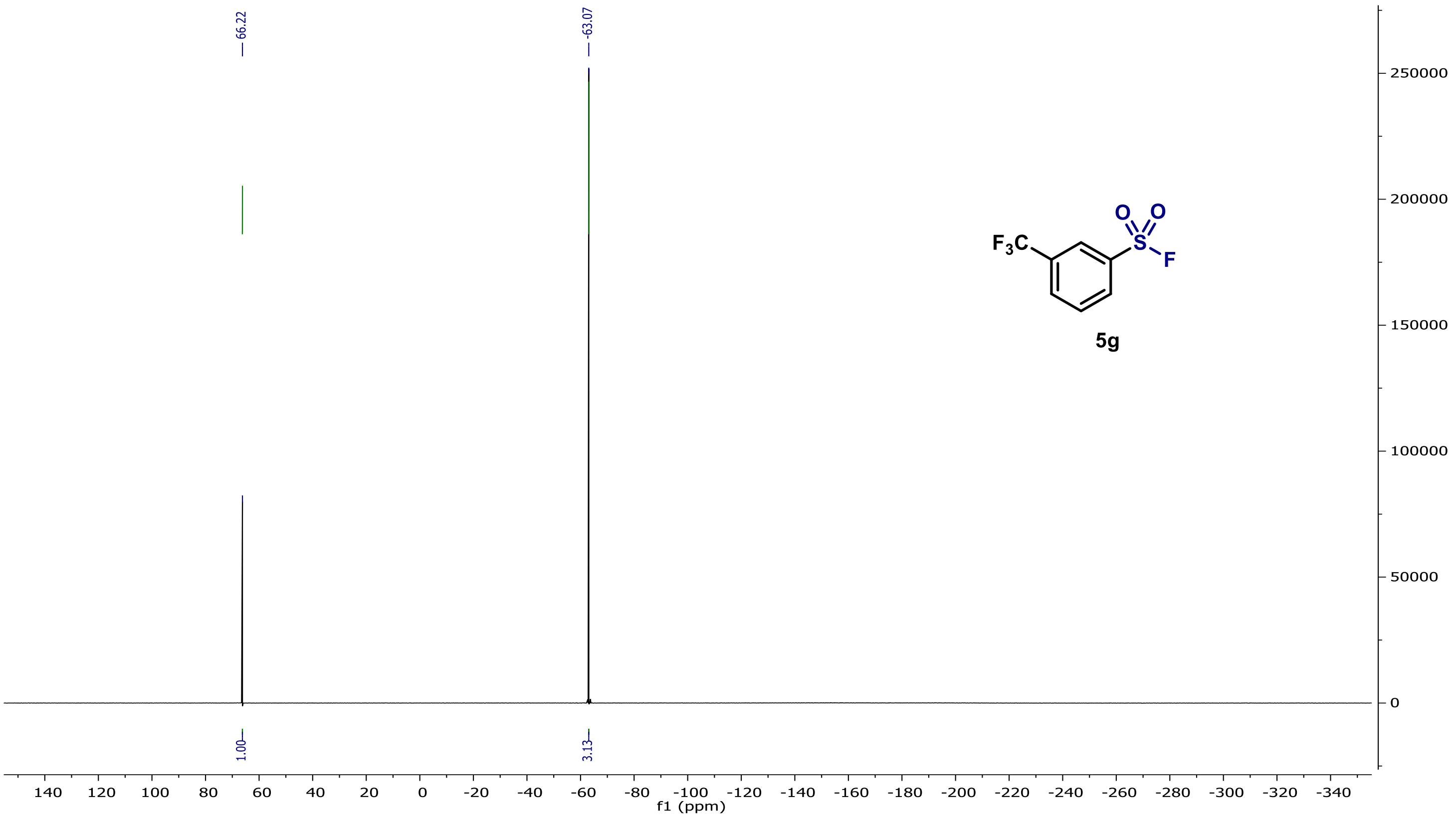

^{13}C Spectra (75 MHz, $CDCl_3$)

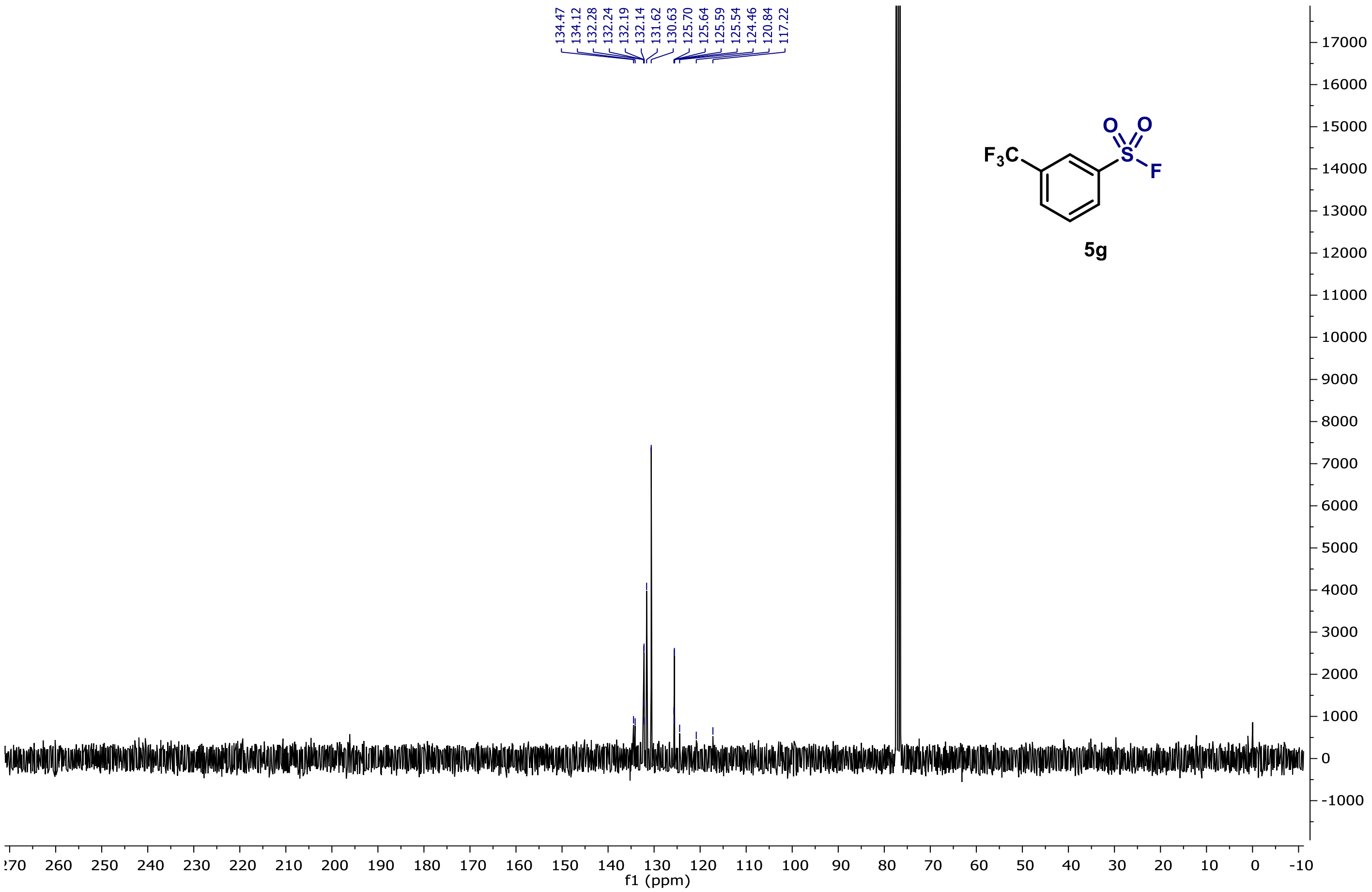

^{1}H Spectra (300 MHz, $CDCl_3$)

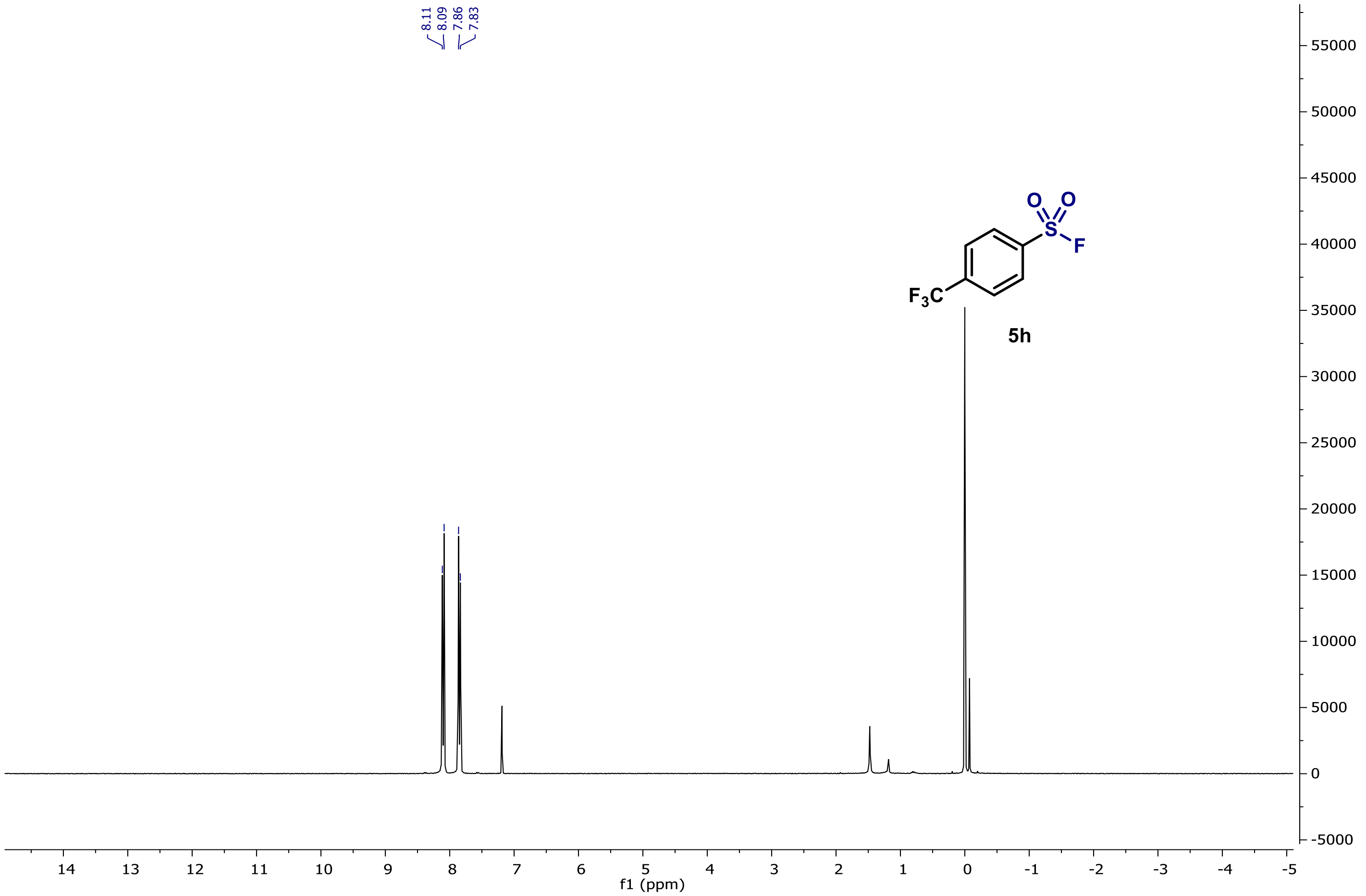

^{19}F{^{1}H} NMR (282 MHz, $CDCl_3$)

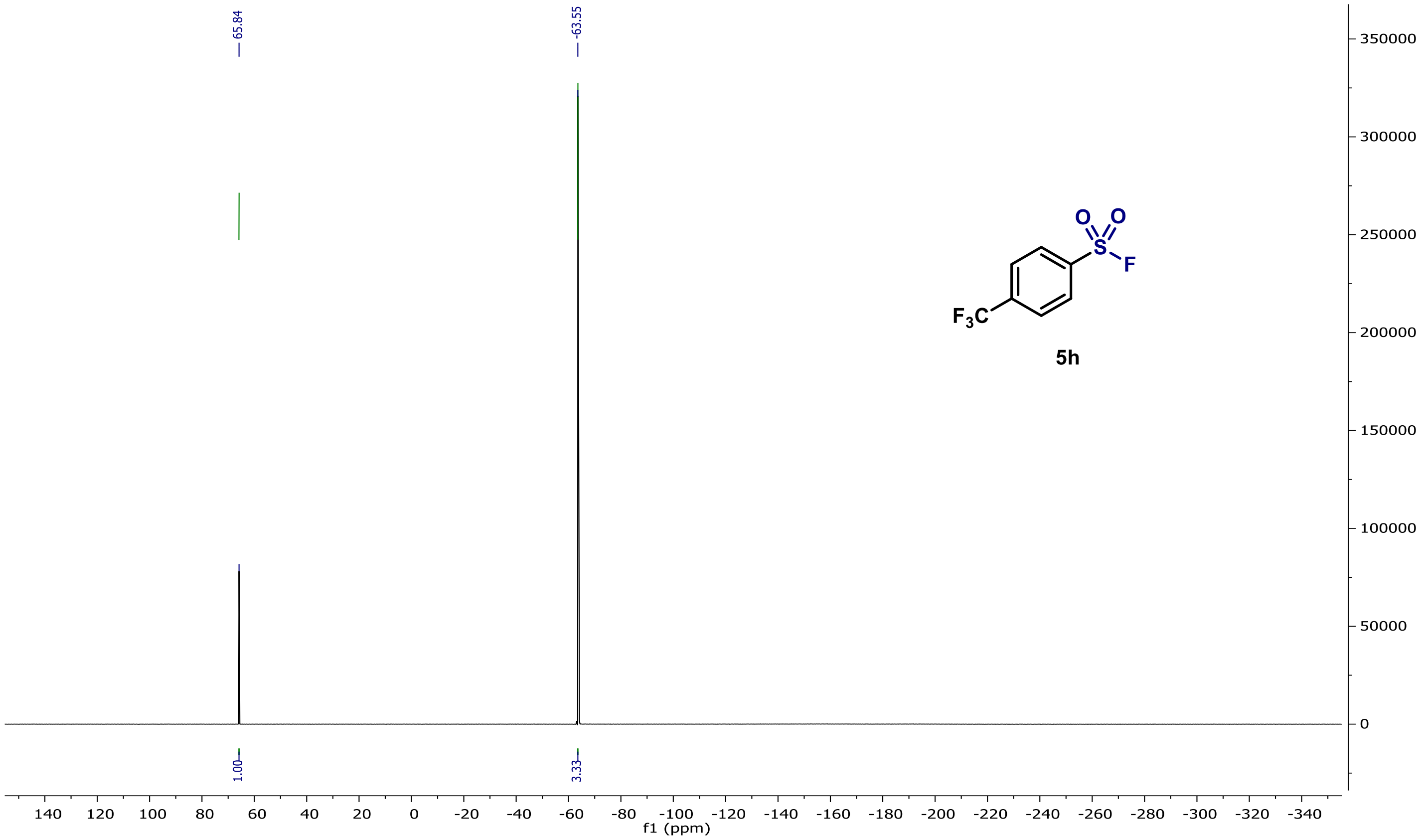

[13]C Spectra (75 MHz, $CDCl_3$)

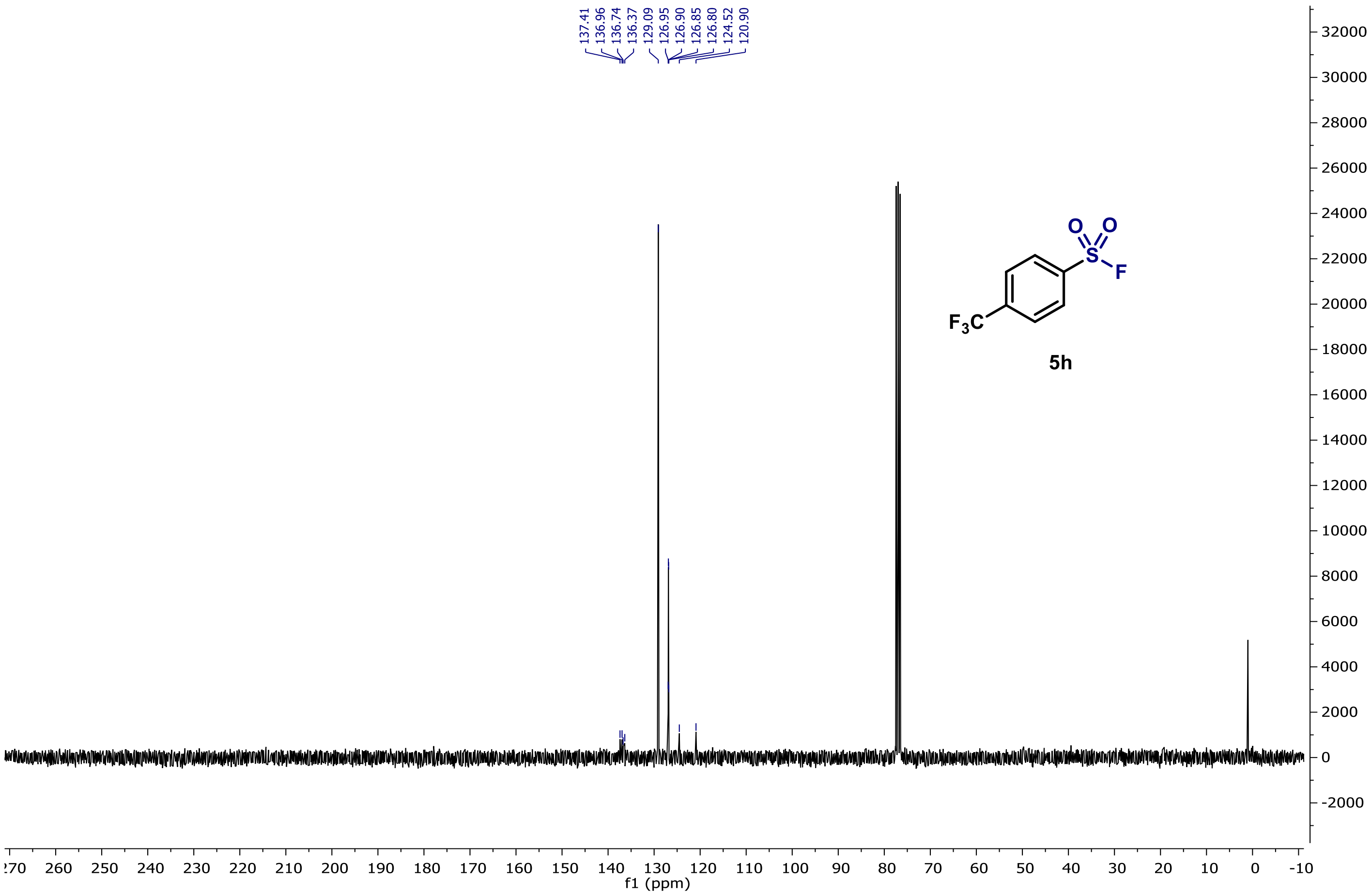

^{1}**H Spectra** (300 MHz, $CDCl_3$)

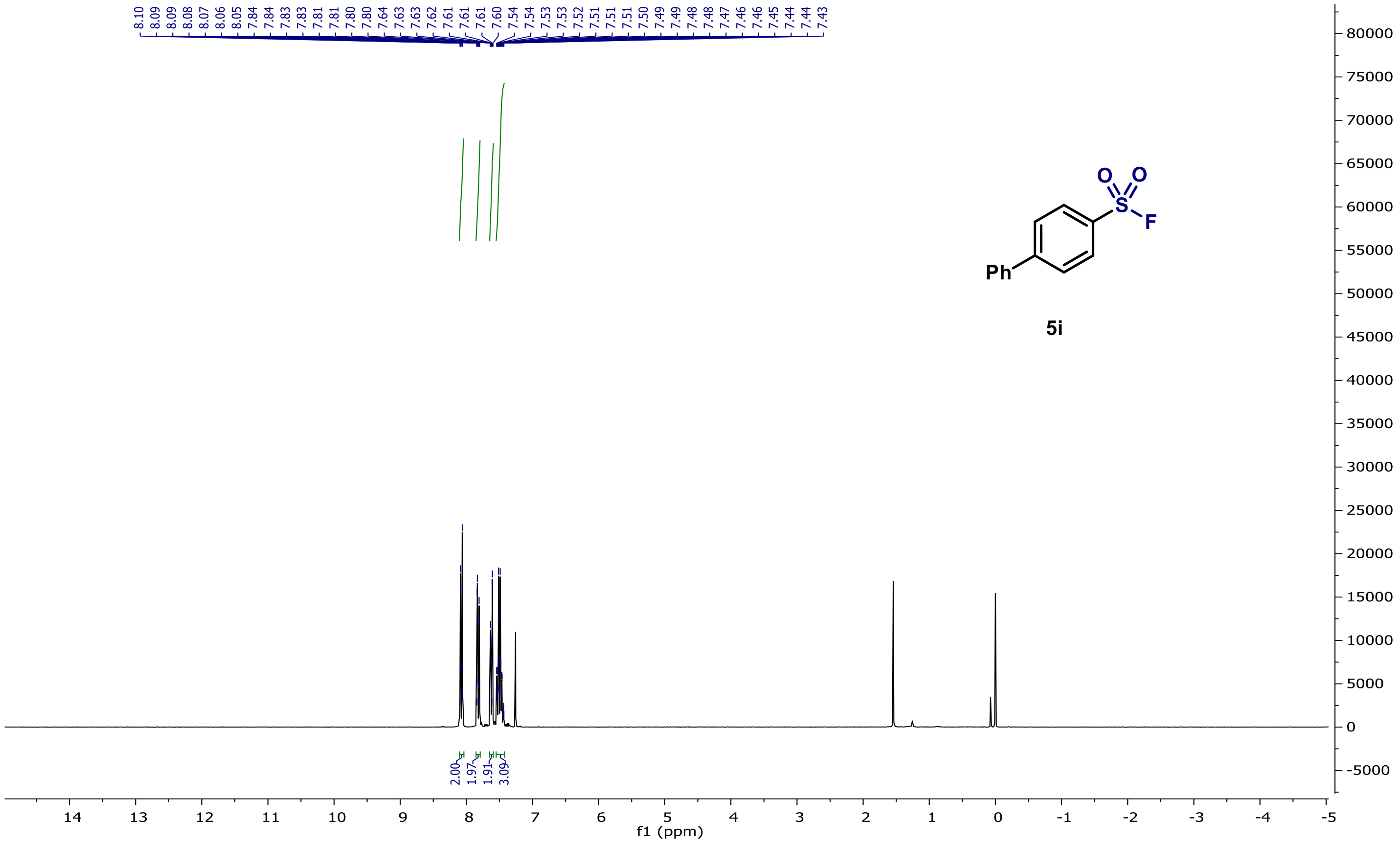

^{19}**F{^{1}H} NMR** (282 MHz, $CDCl_3$)

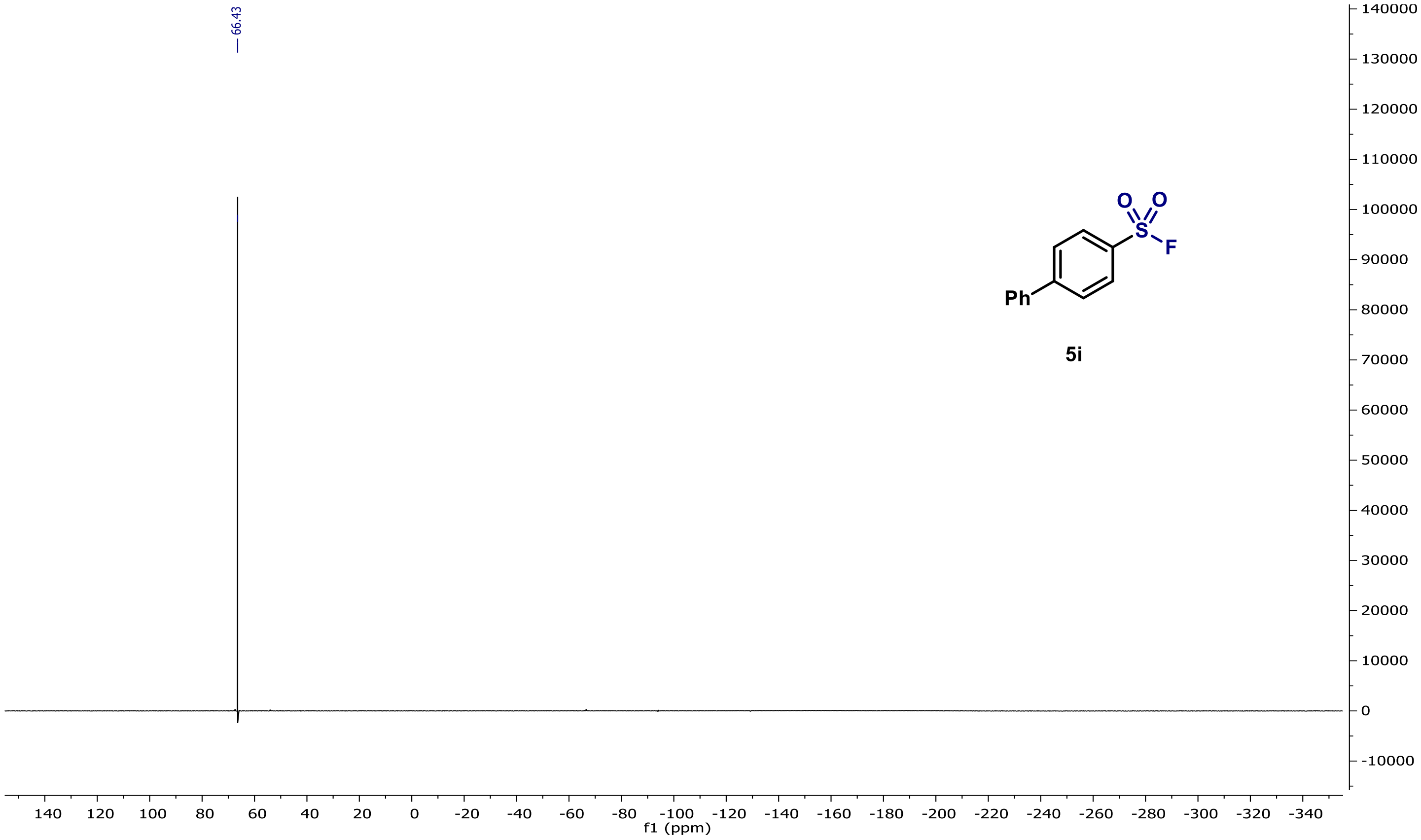

^{13}C Spectra (75 MHz, $CDCl_3$)

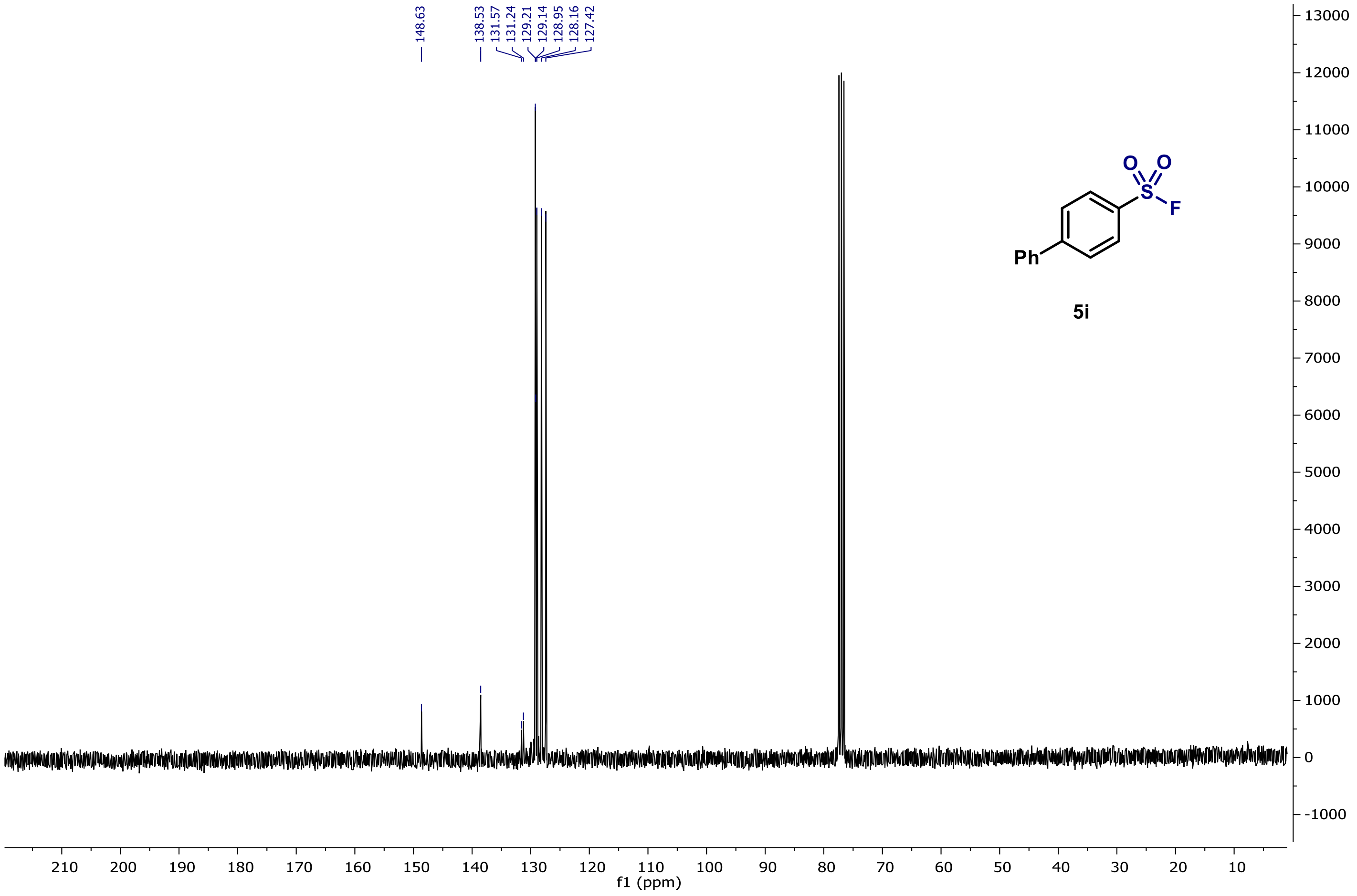

^{1}H Spectra (300 MHz, $CDCl_3$)

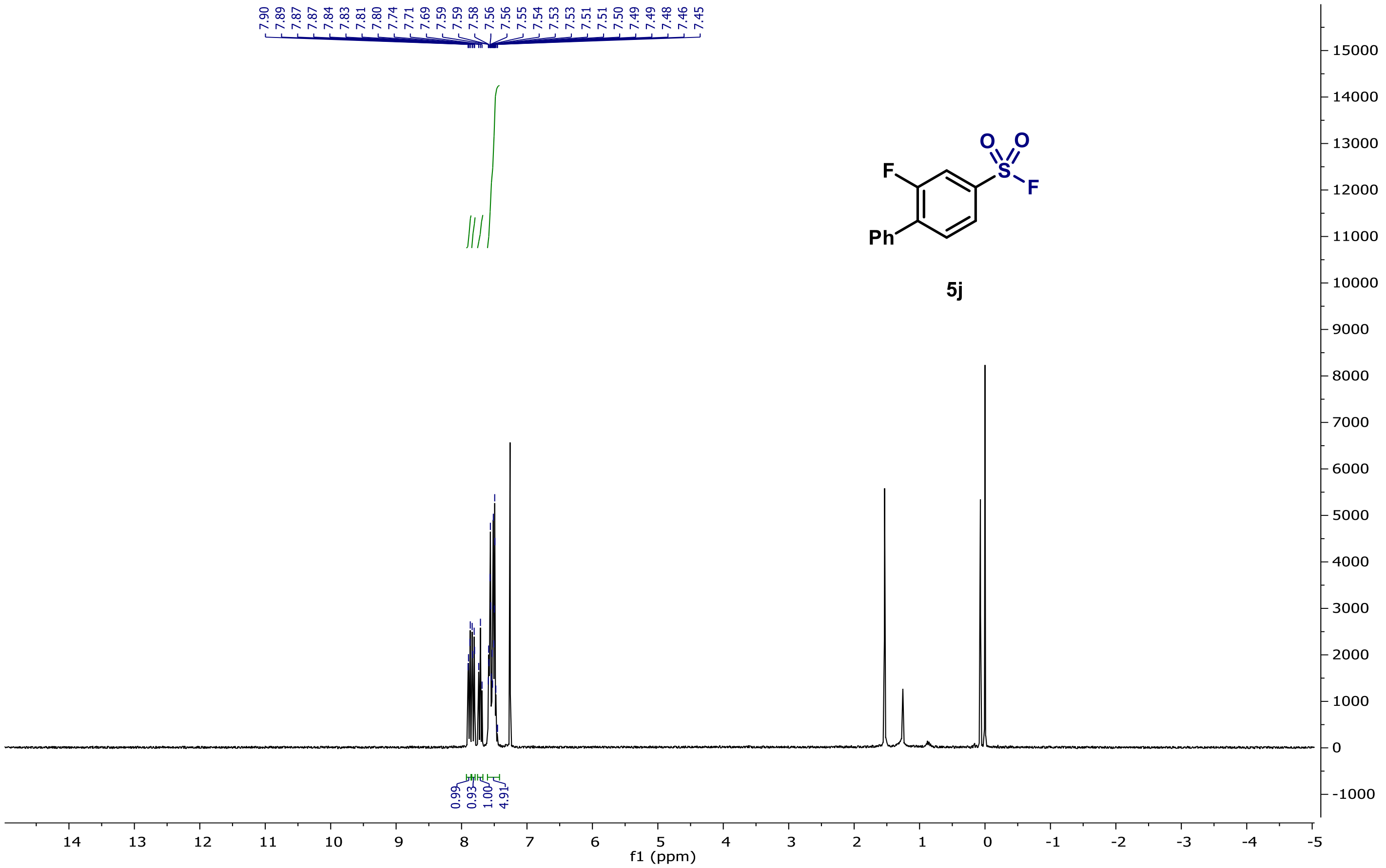

^{19}F{^{1}H} NMR (282 MHz, $CDCl_3$)

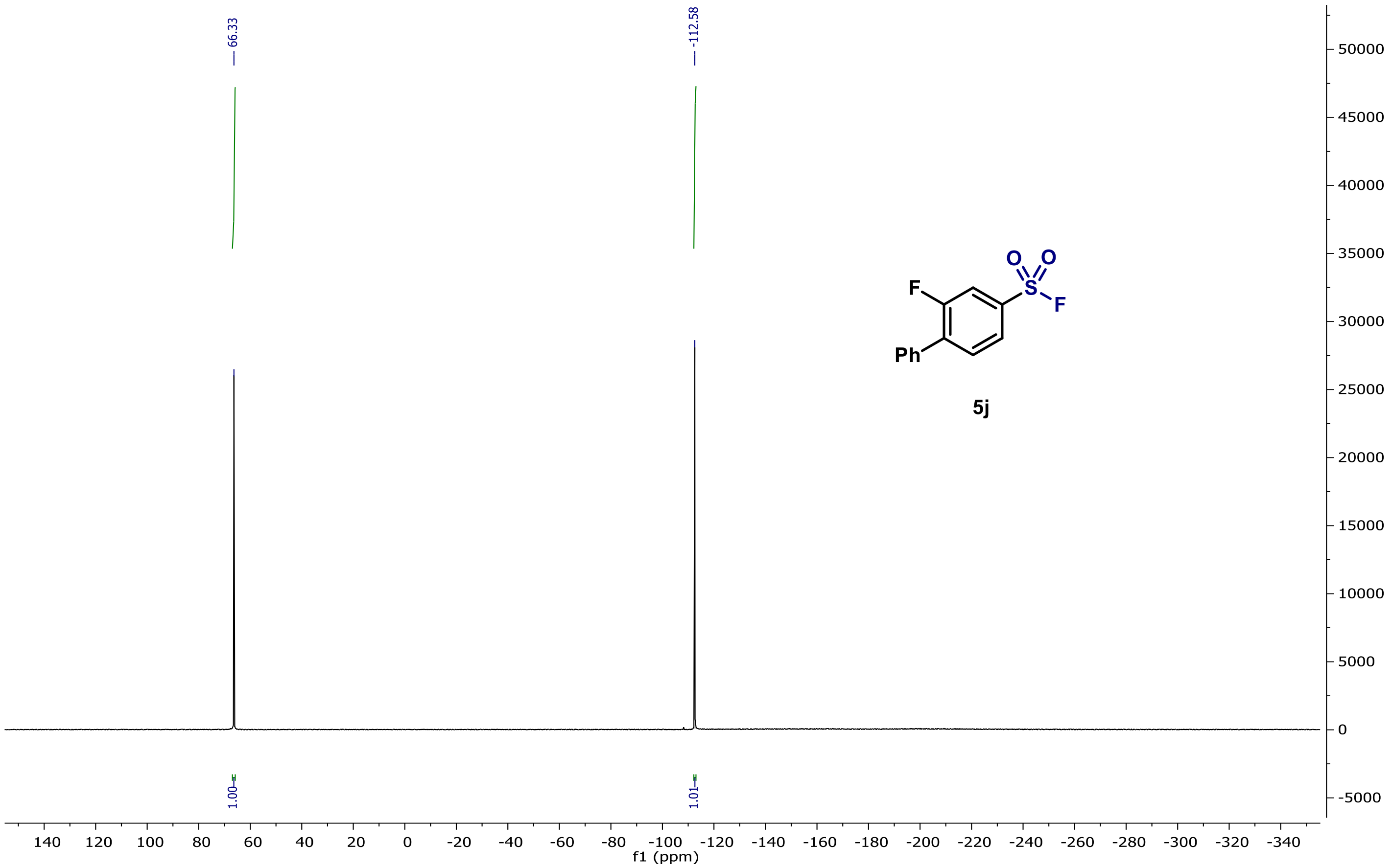

[13]C Spectra (75 MHz, $CDCl_3$)

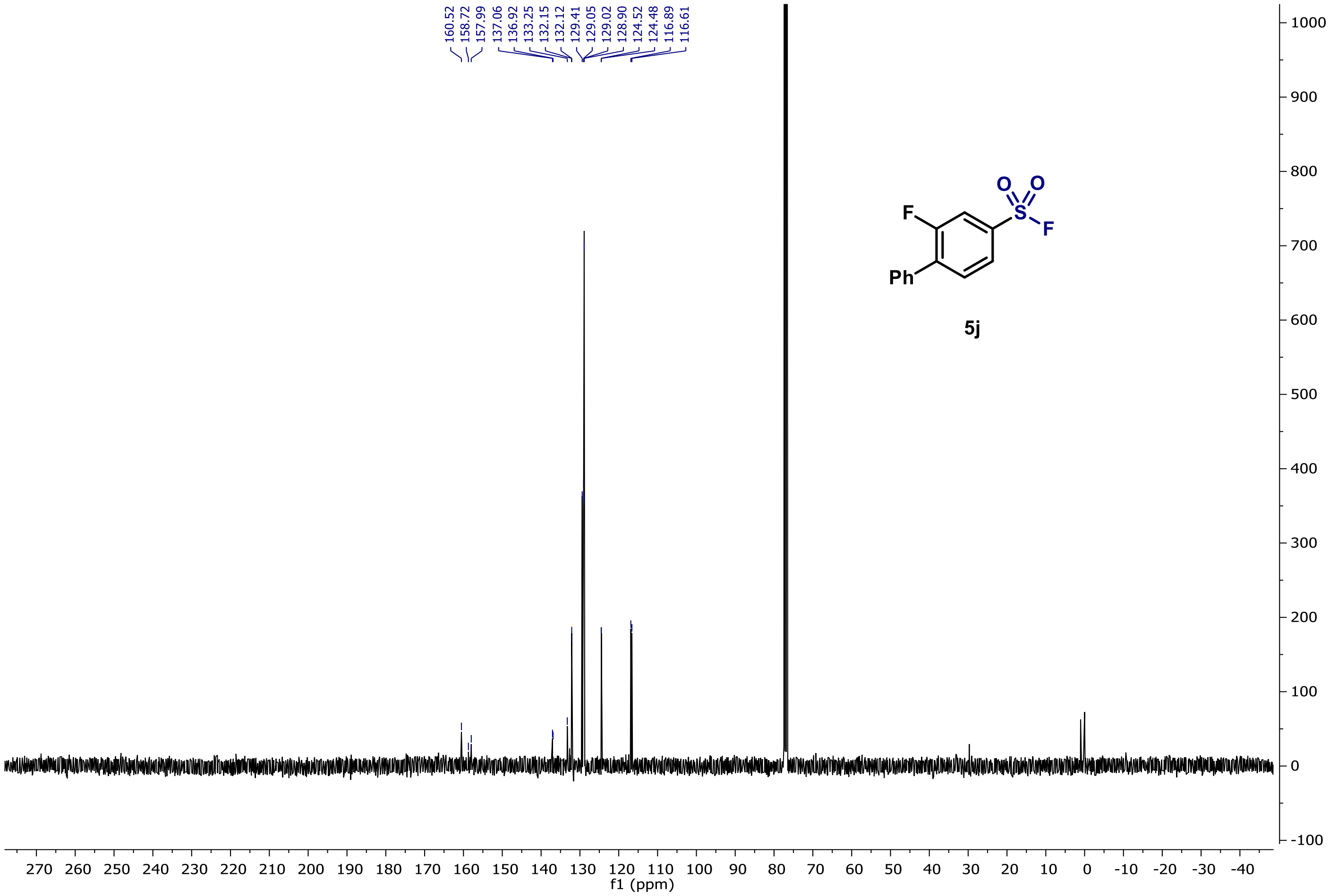

¹H Spectra (300 MHz, $CDCl_3$)

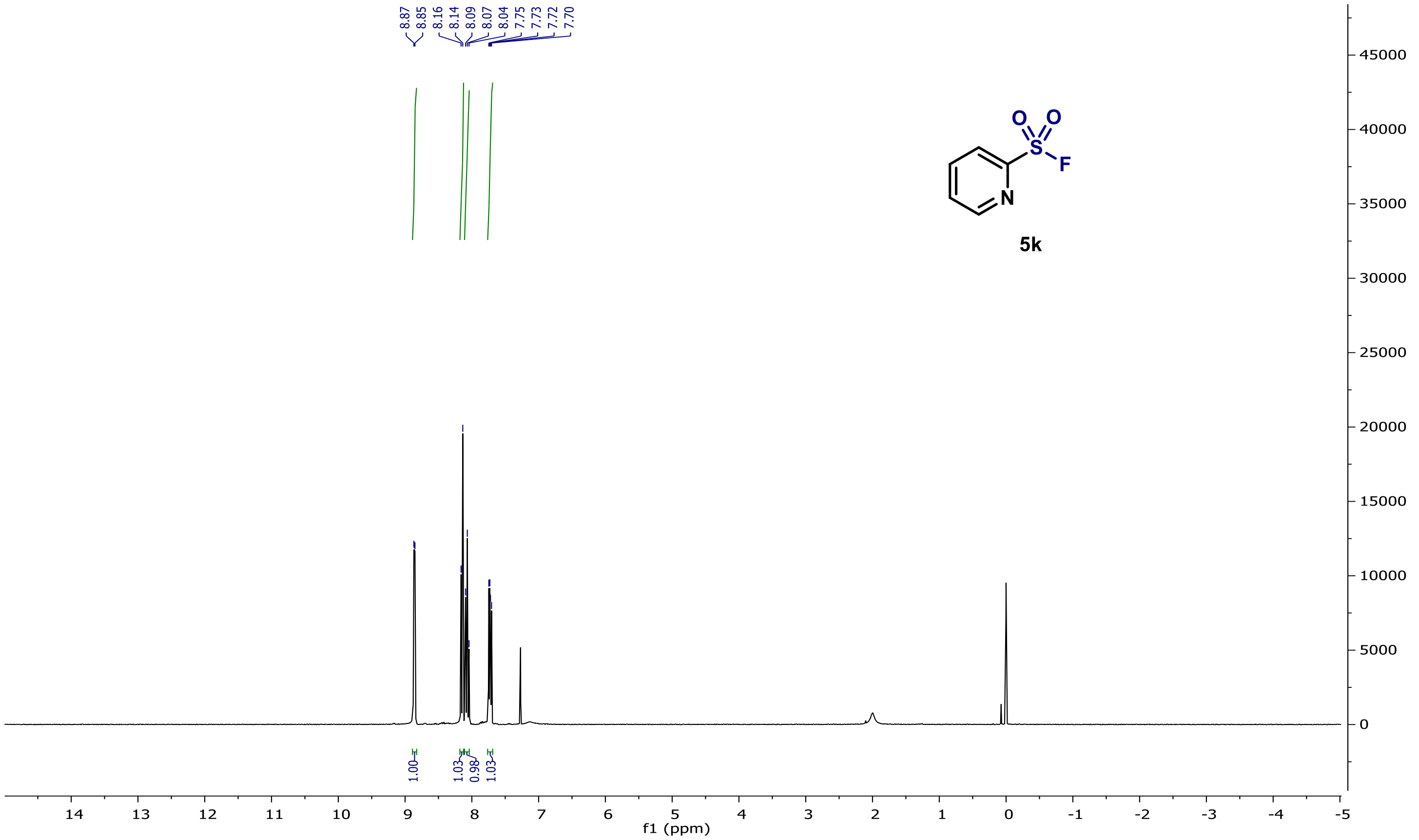

^{19}F{^{1}H} NMR (282 MHz, $CDCl_3$)

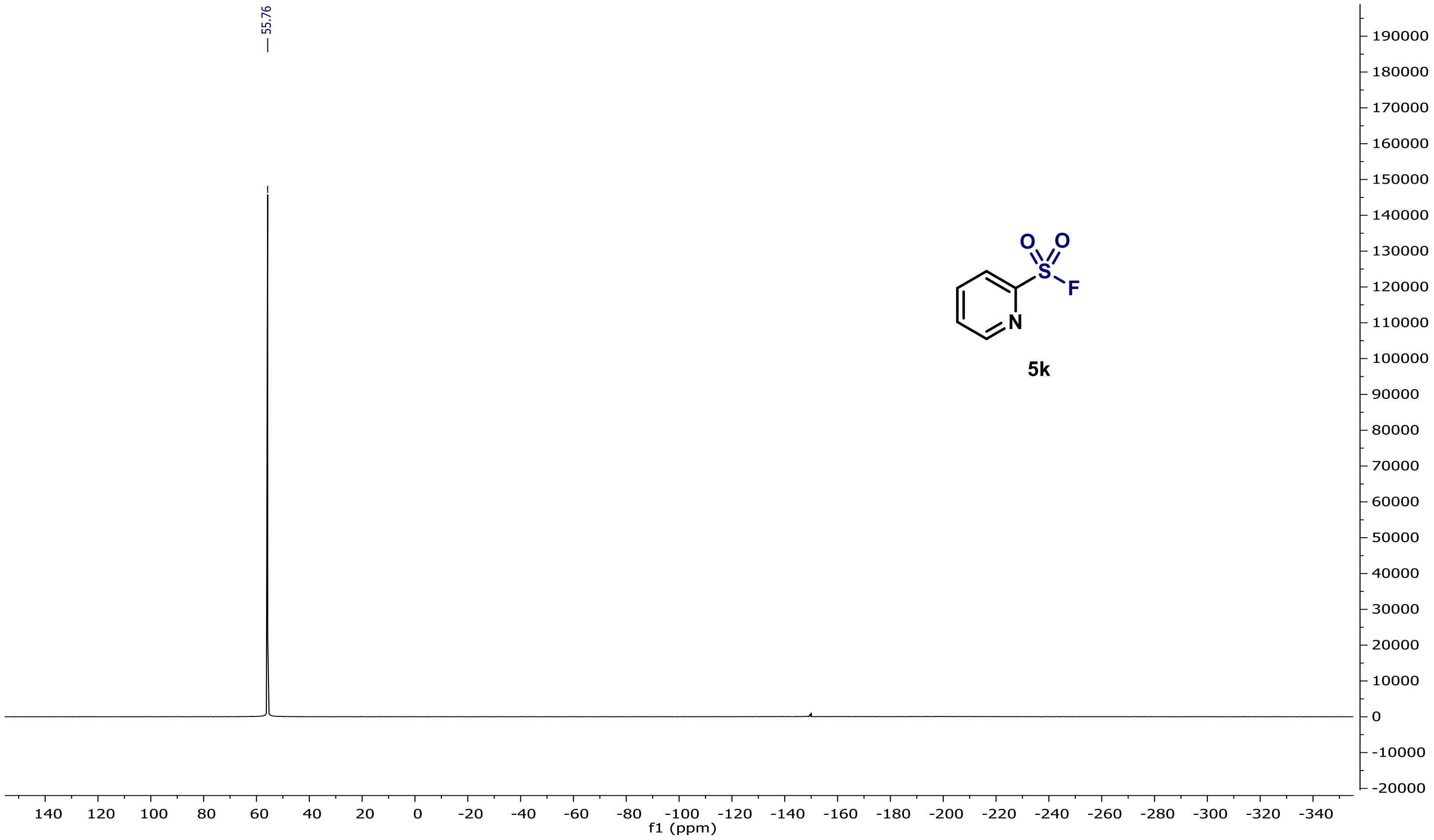

^{13}C Spectra (75 MHz, $CDCl_3$)

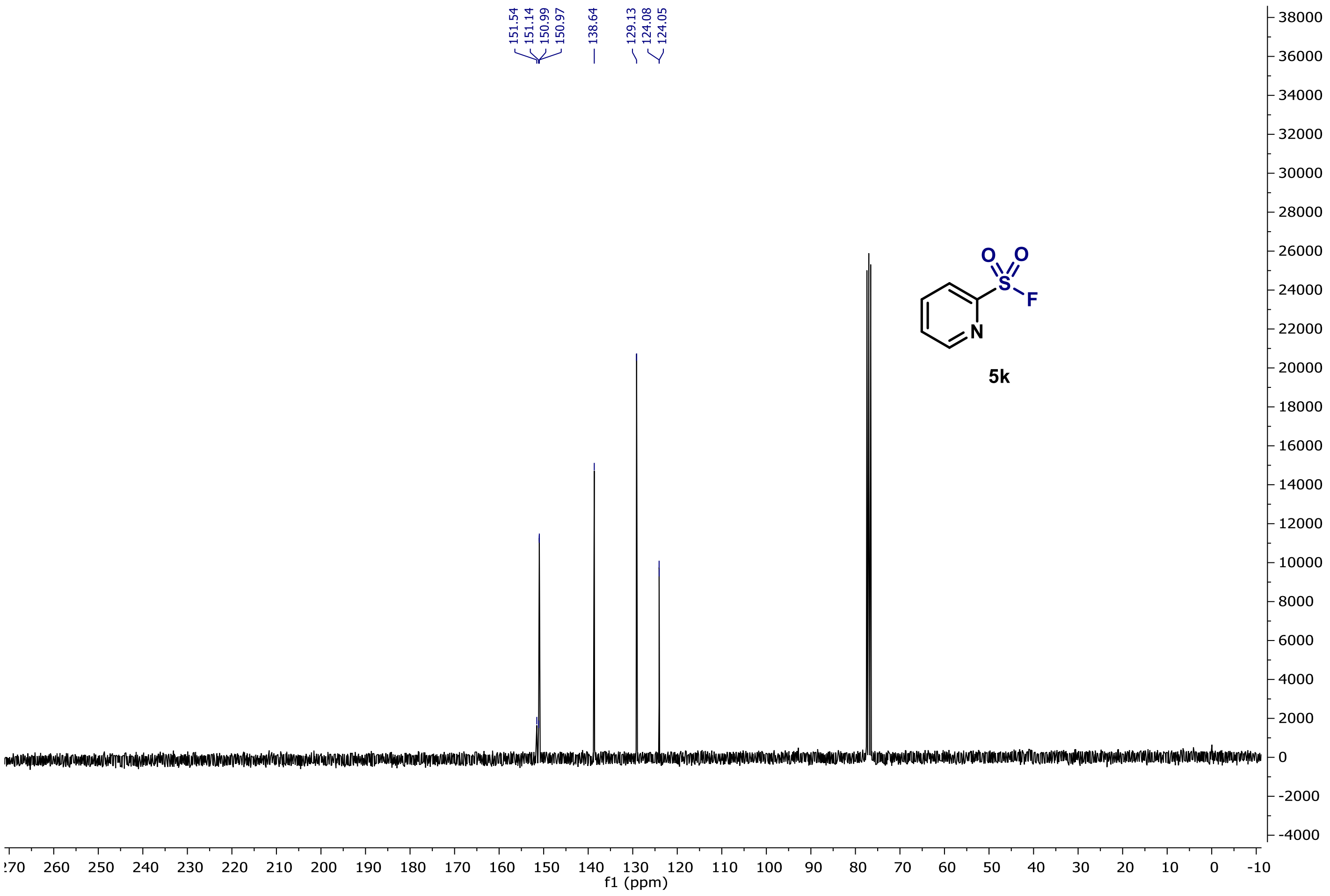

^{1}H Spectra (300 MHz, $CDCl_3$)

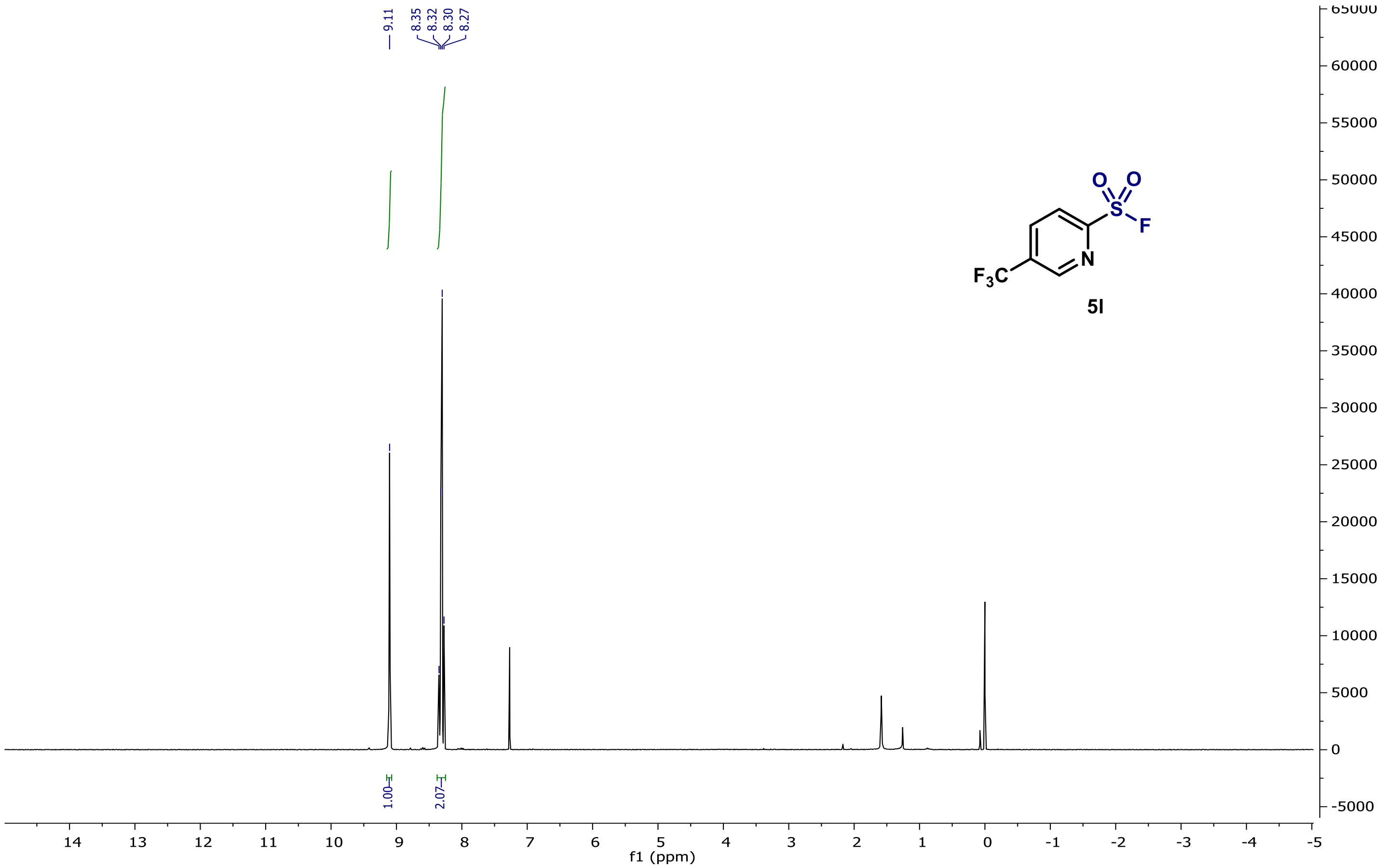

^{19}F{^{1}H} NMR (282 MHz, $CDCl_3$)

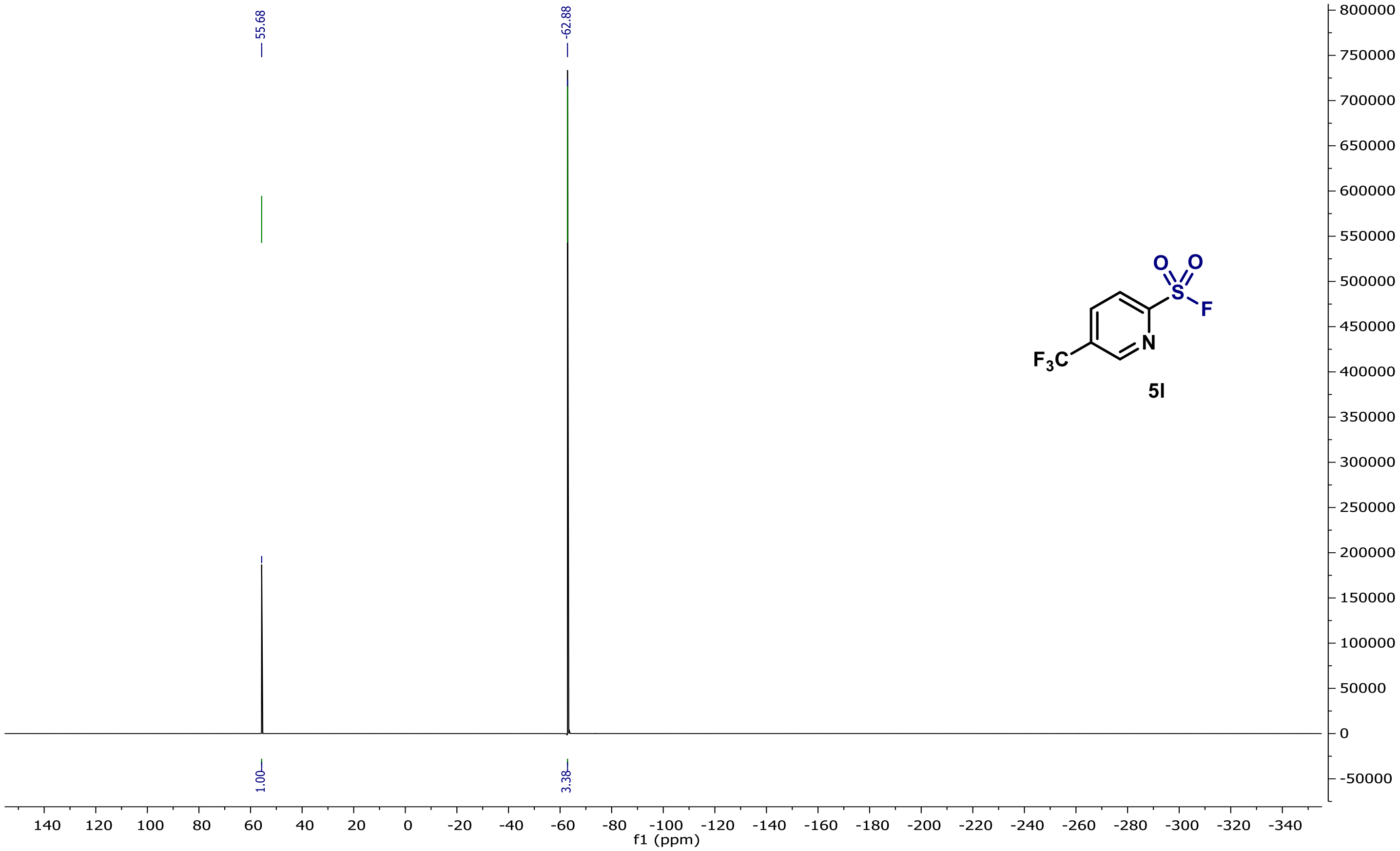

^{13}C Spectra (75 MHz, $CDCl_3$)

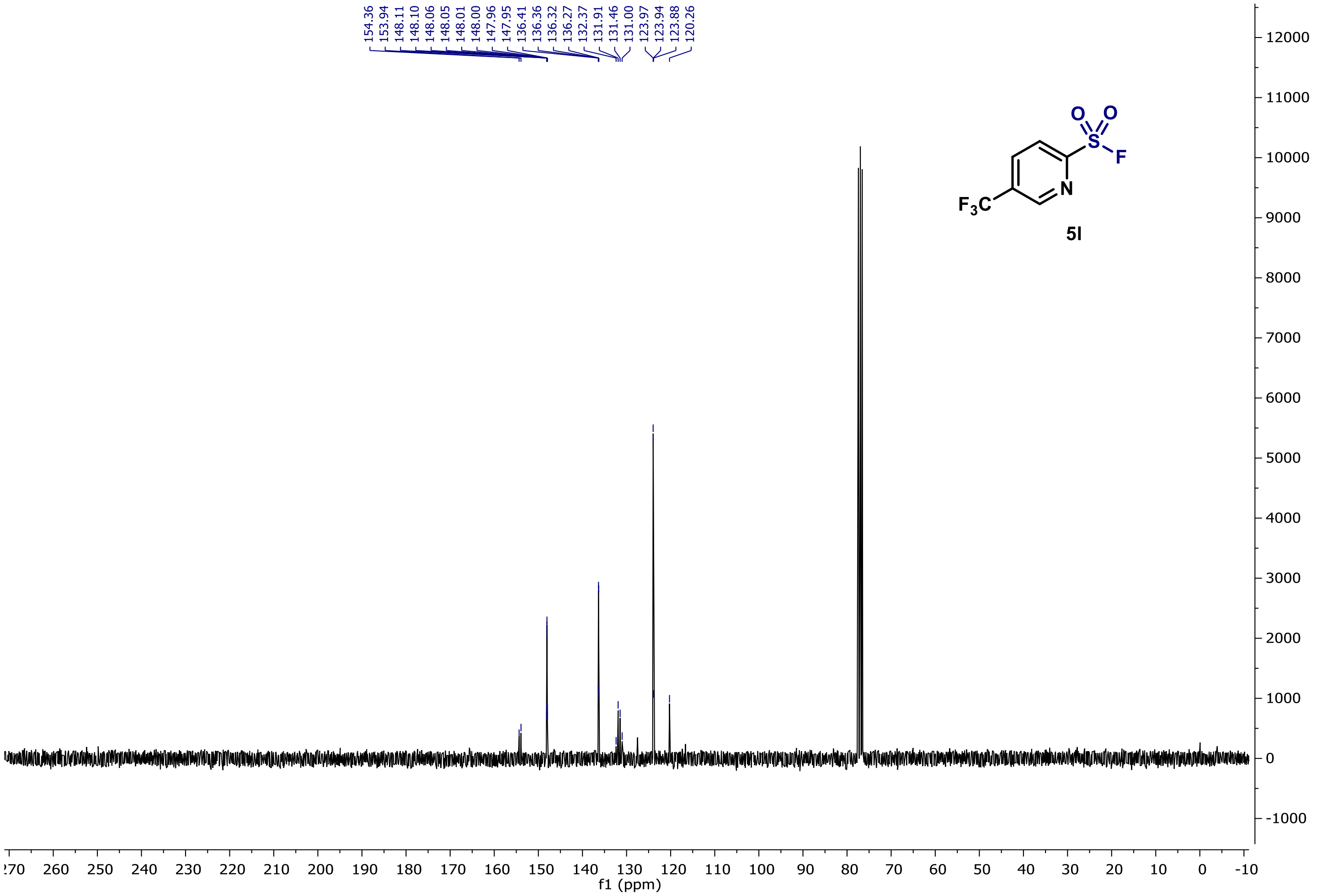

^{1}H Spectra (300 MHz, $CDCl_3$)

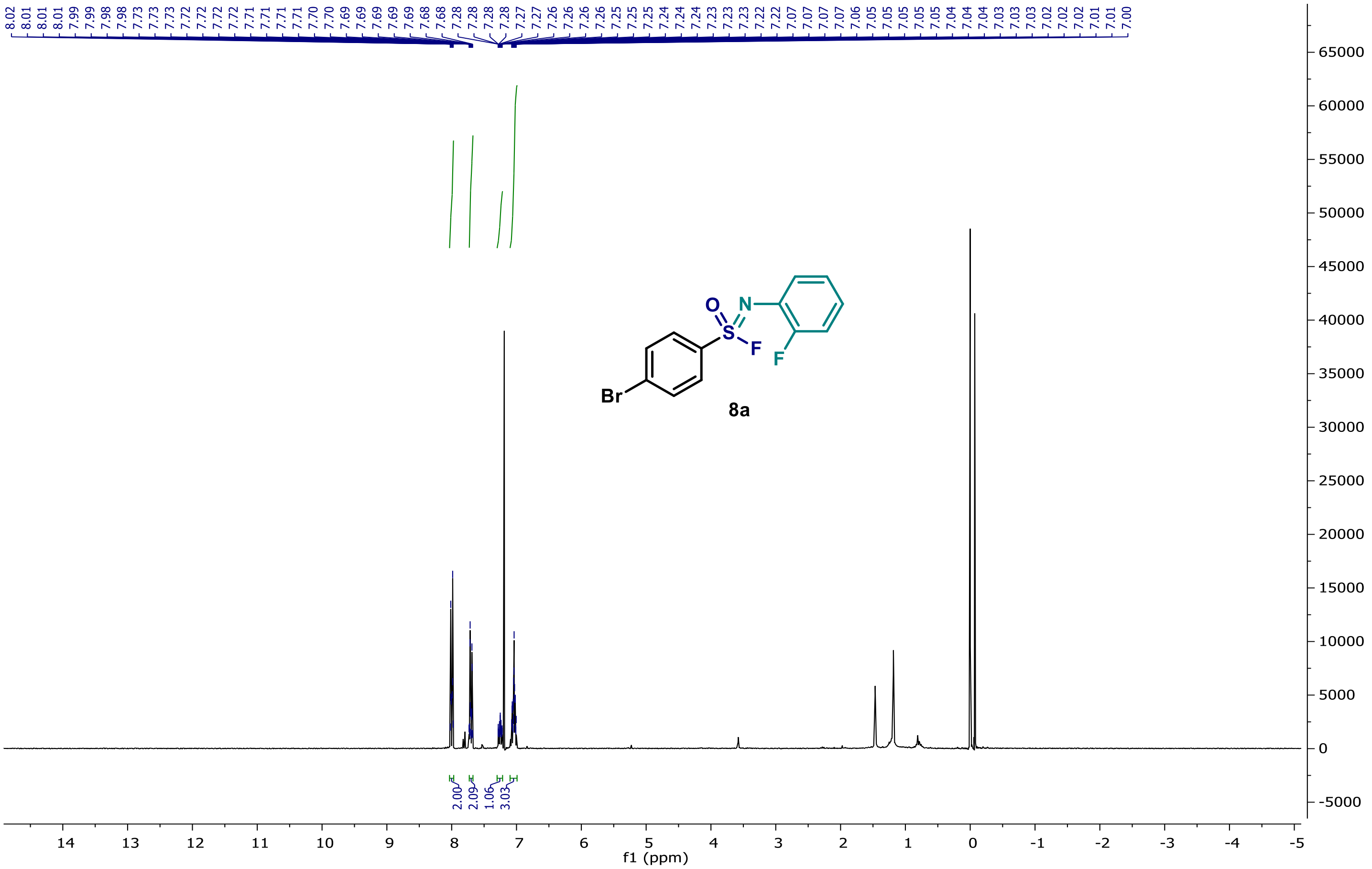

^{19}F{^{1}H} NMR (282 MHz, $CDCl_3$)

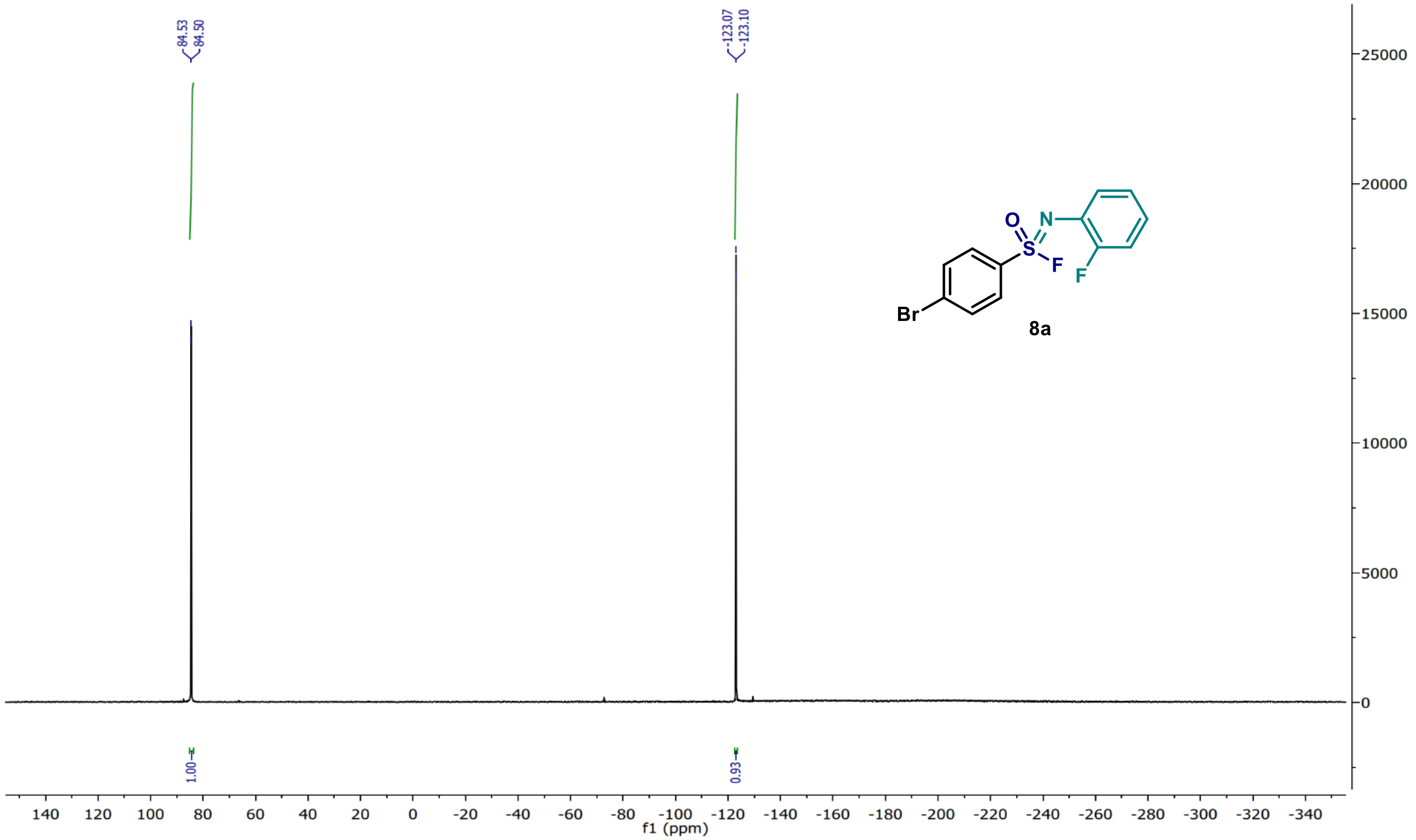

^{13}C Spectra (75 MHz, $CDCl_3$)

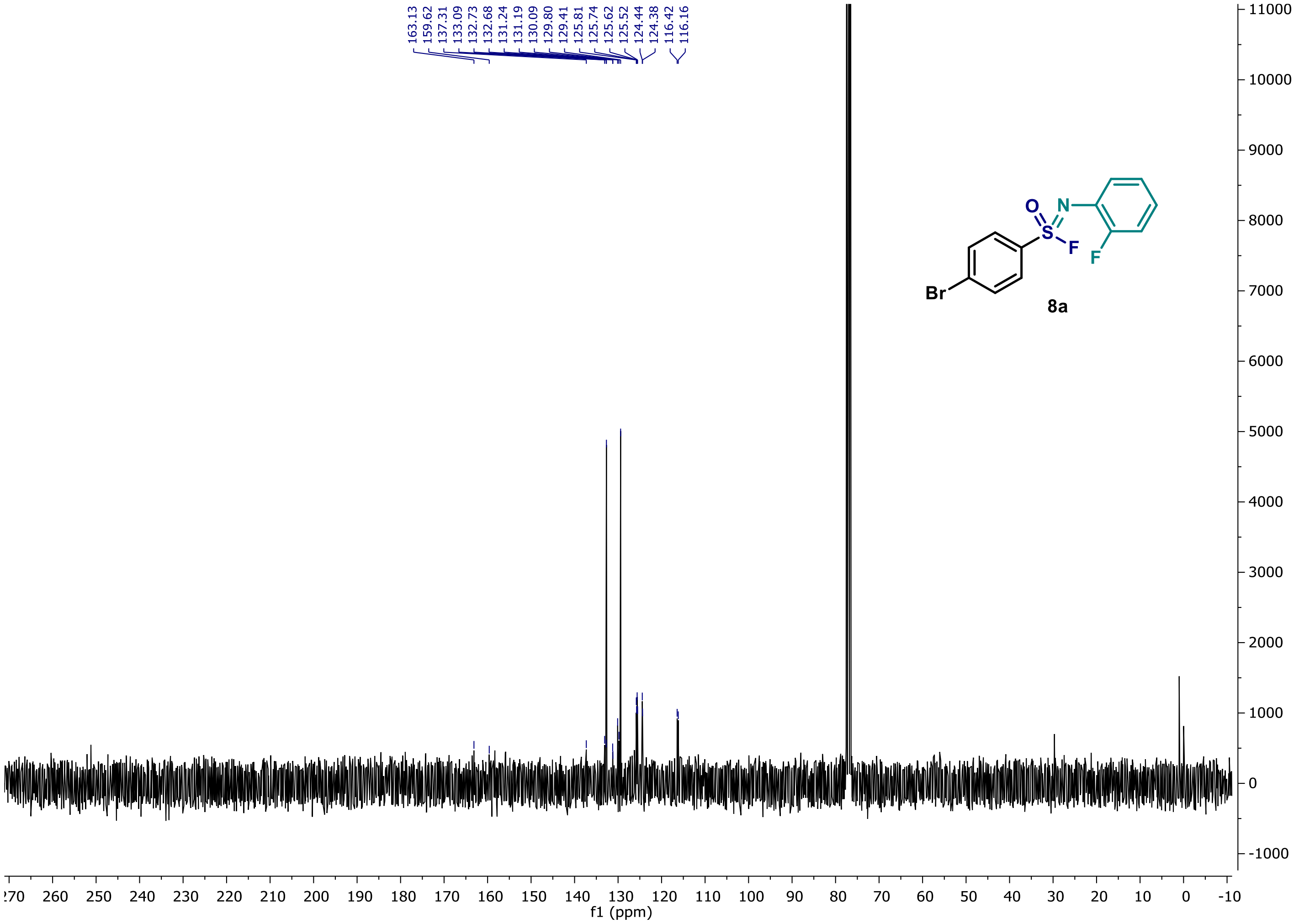

[1]H Spectra (300 MHz, $CDCl_3$)

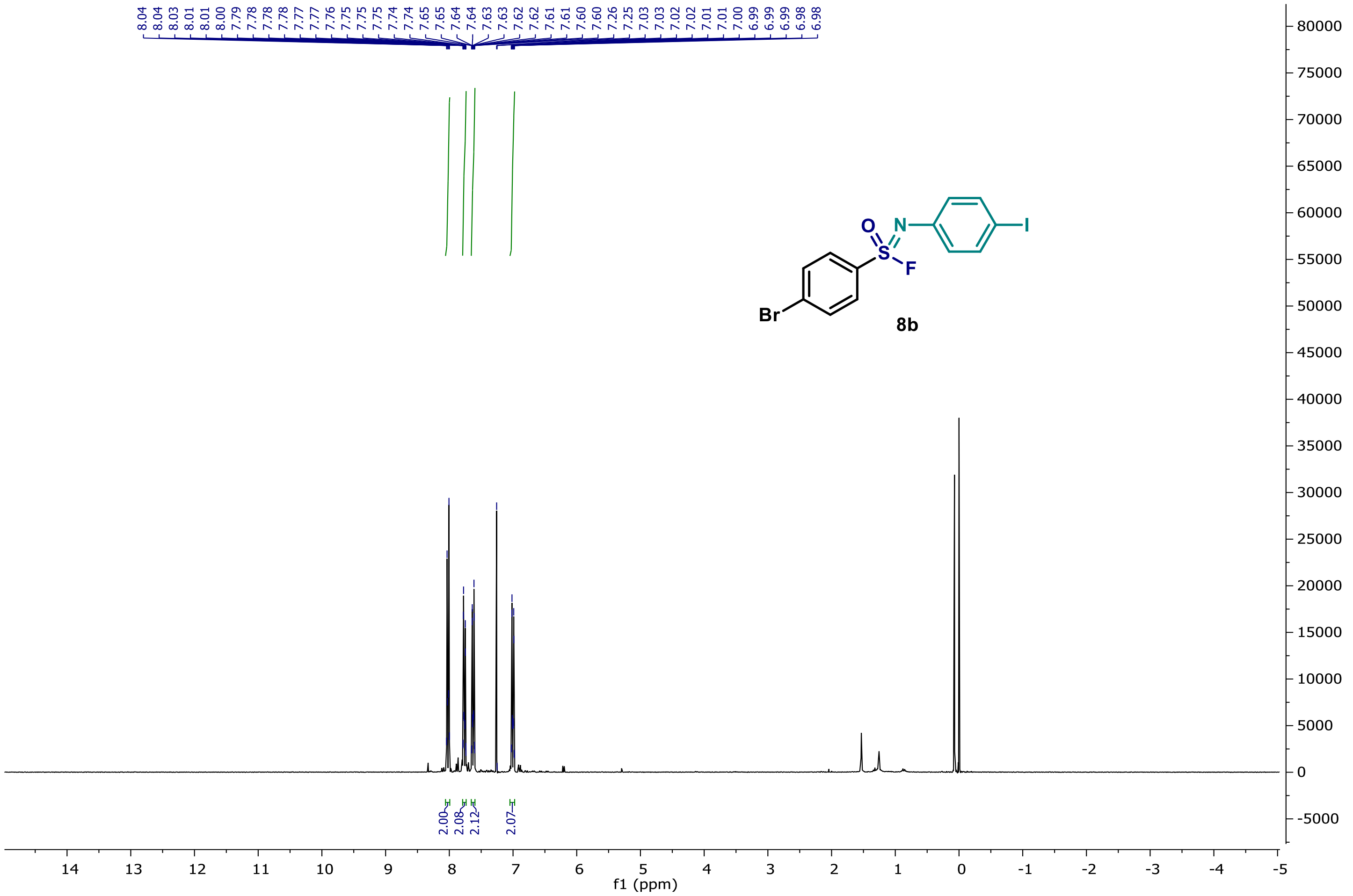

^{19}F{^{1}H} NMR (282 MHz, $CDCl_3$)

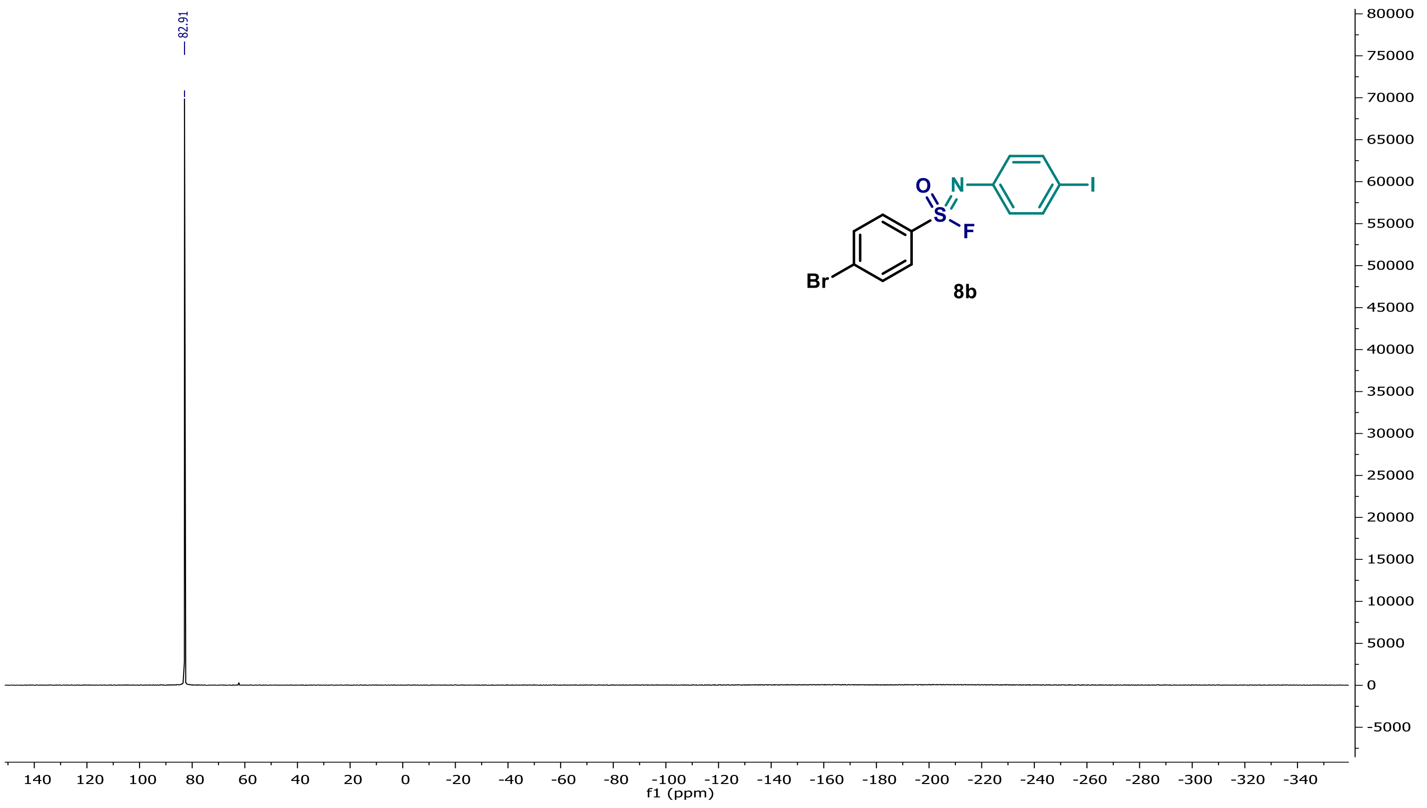

[13]C Spectra (75 MHz, $CDCl_3$)

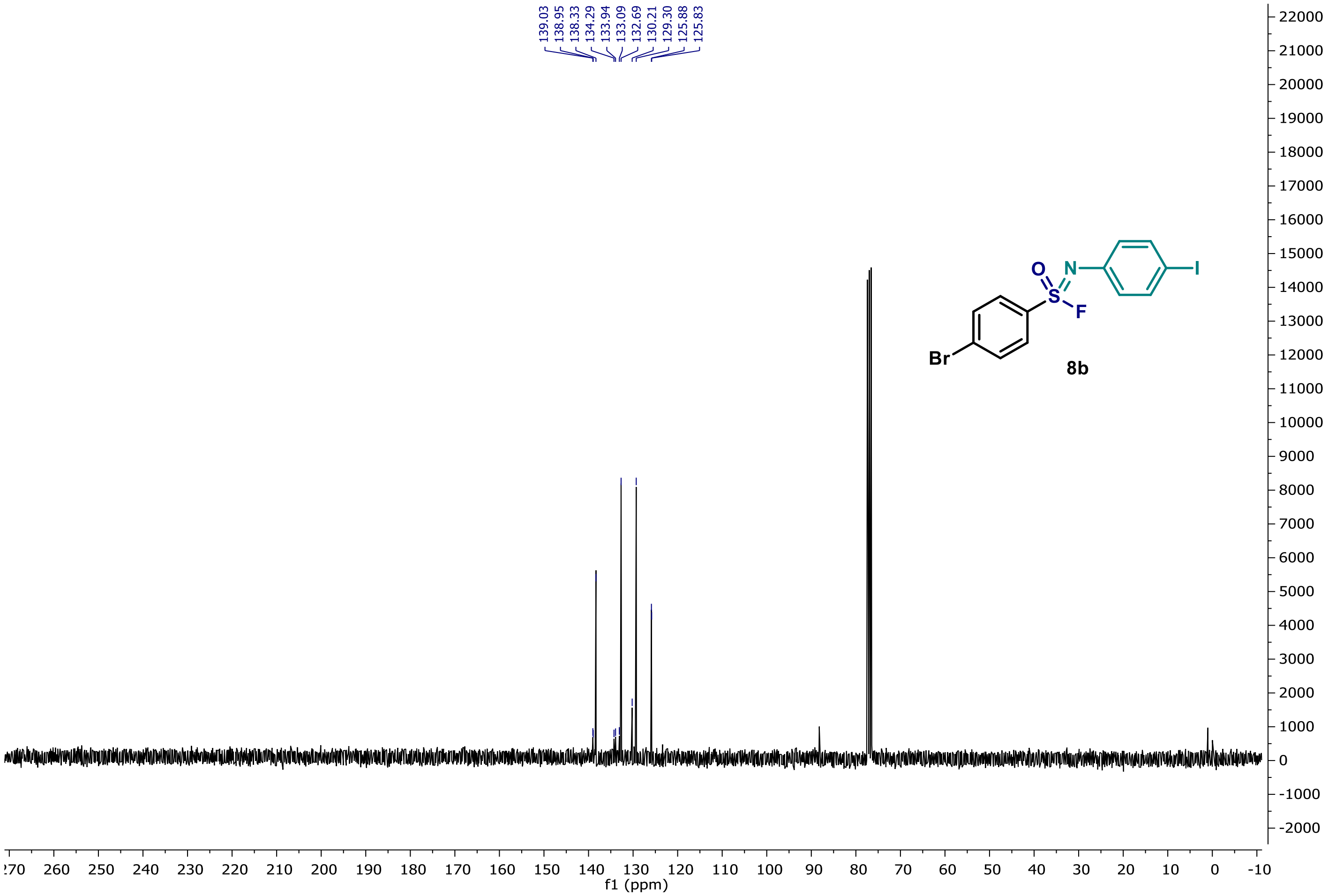

^{1}**H Spectra** (300 MHz, $CDCl_3$)

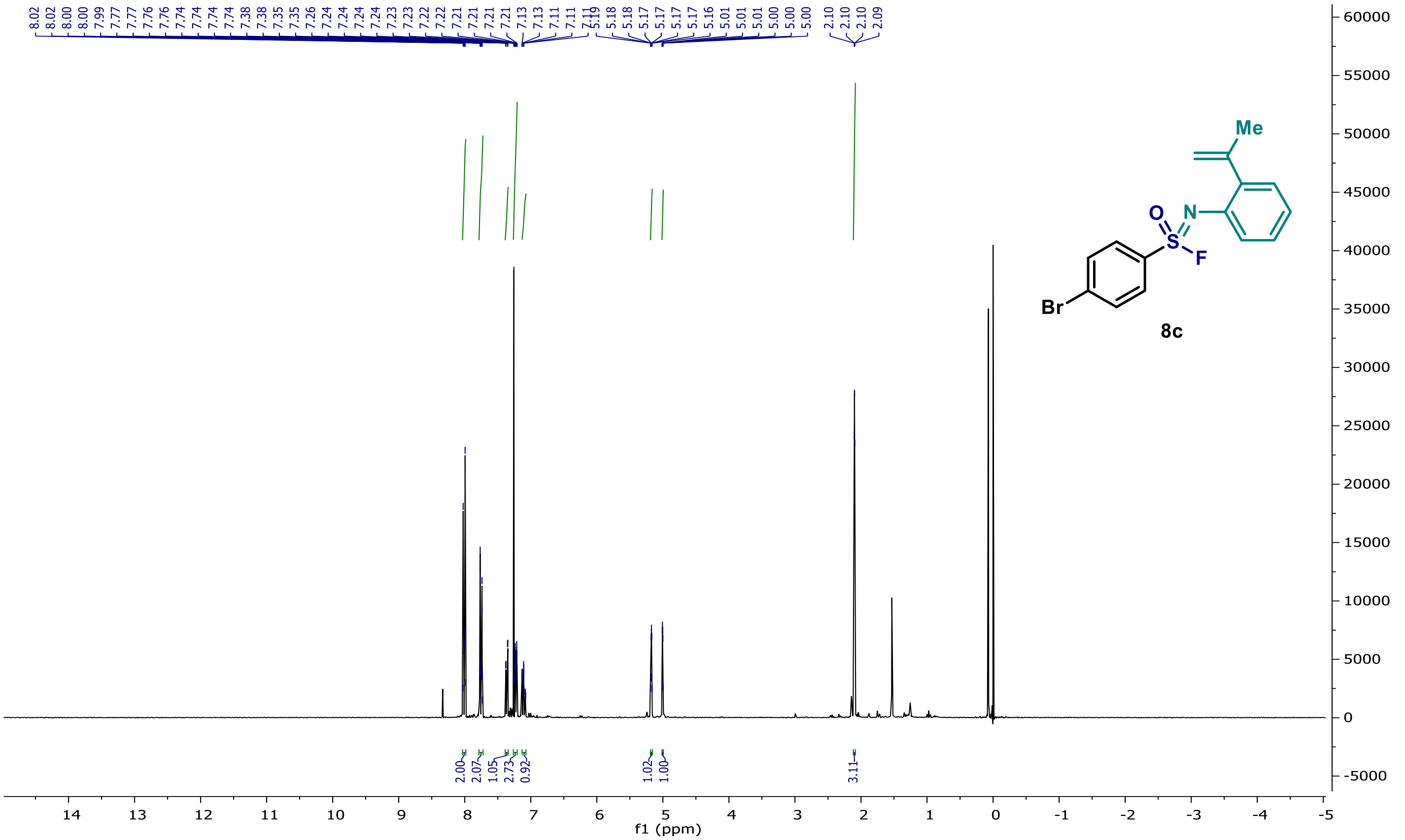

^{19}**F{**^{1}**H} NMR** (282 MHz, $CDCl_3$)

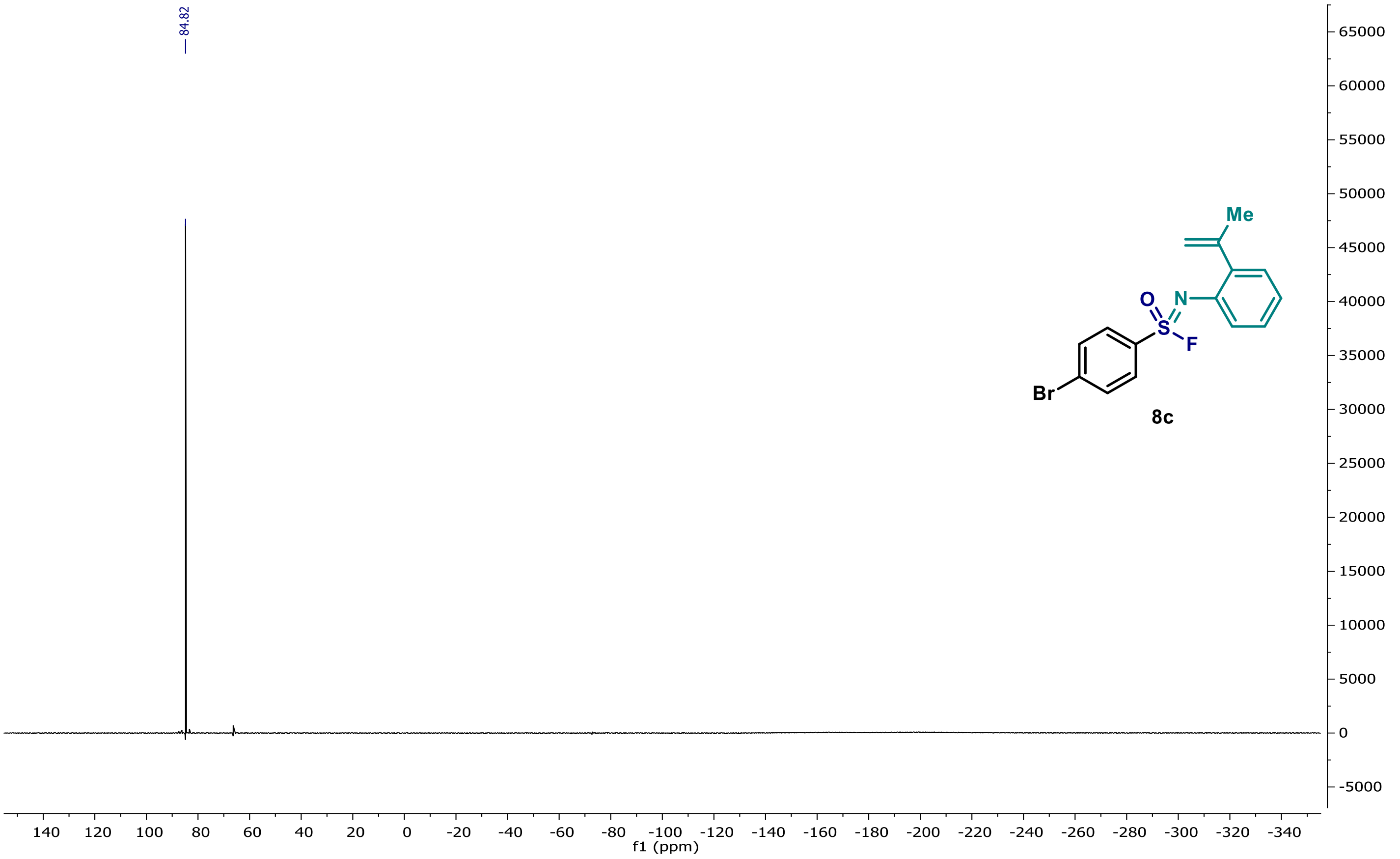

^{13}C Spectra (75 MHz, $CDCl_3$)

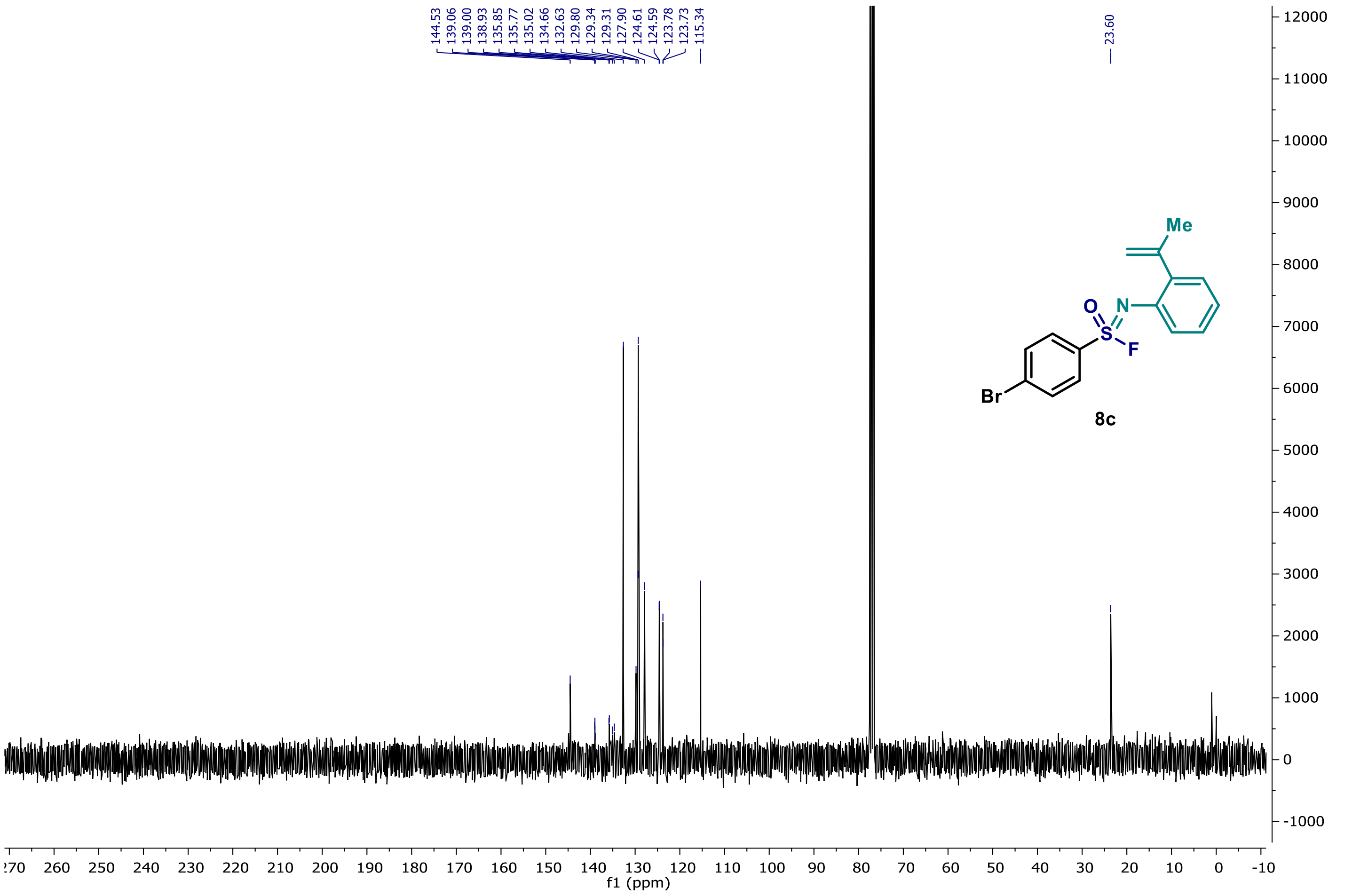

^{1}H Spectra (300 MHz, $CDCl_3$)

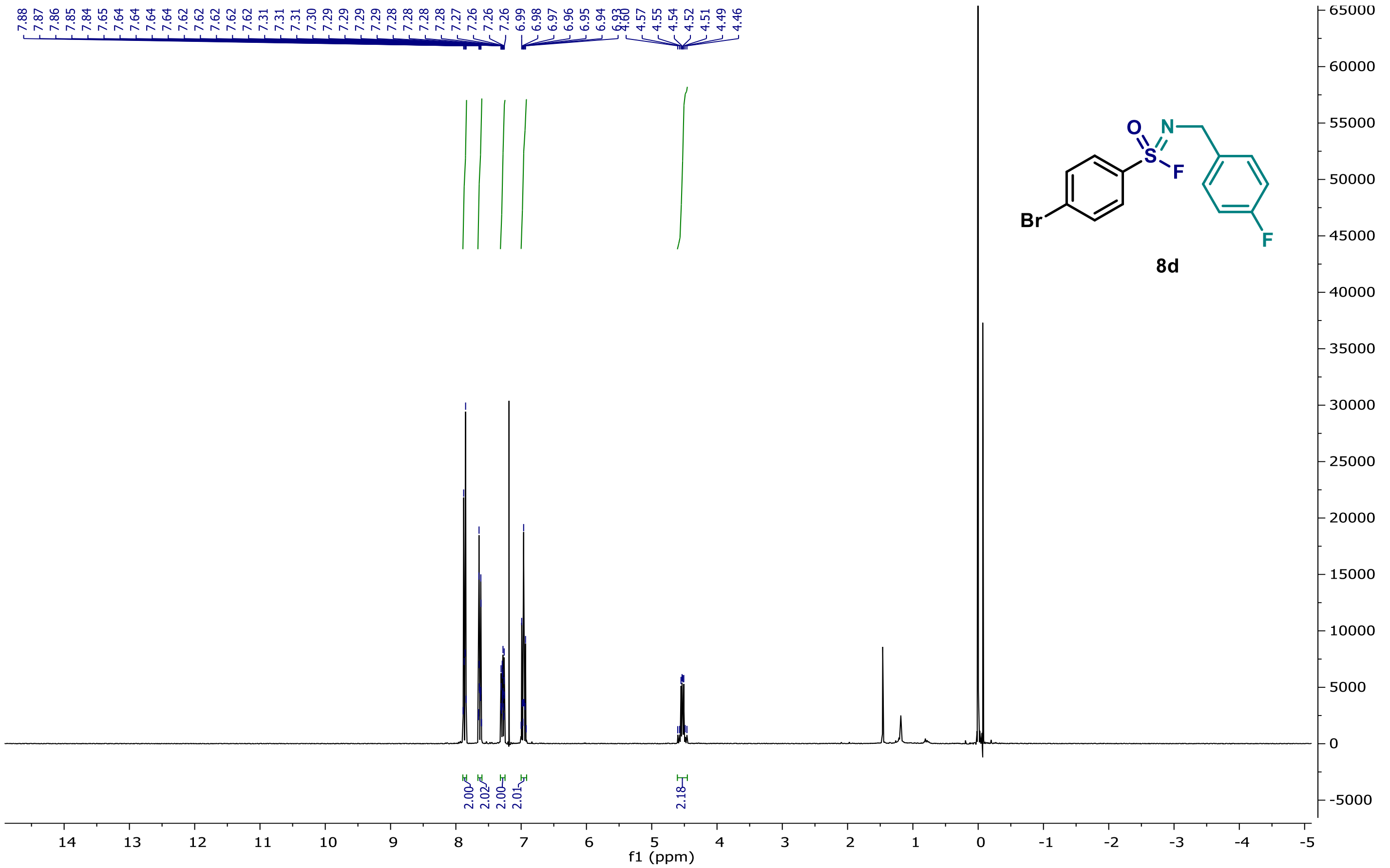

^{19}F{^{1}H} NMR (282 MHz, $CDCl_3$)

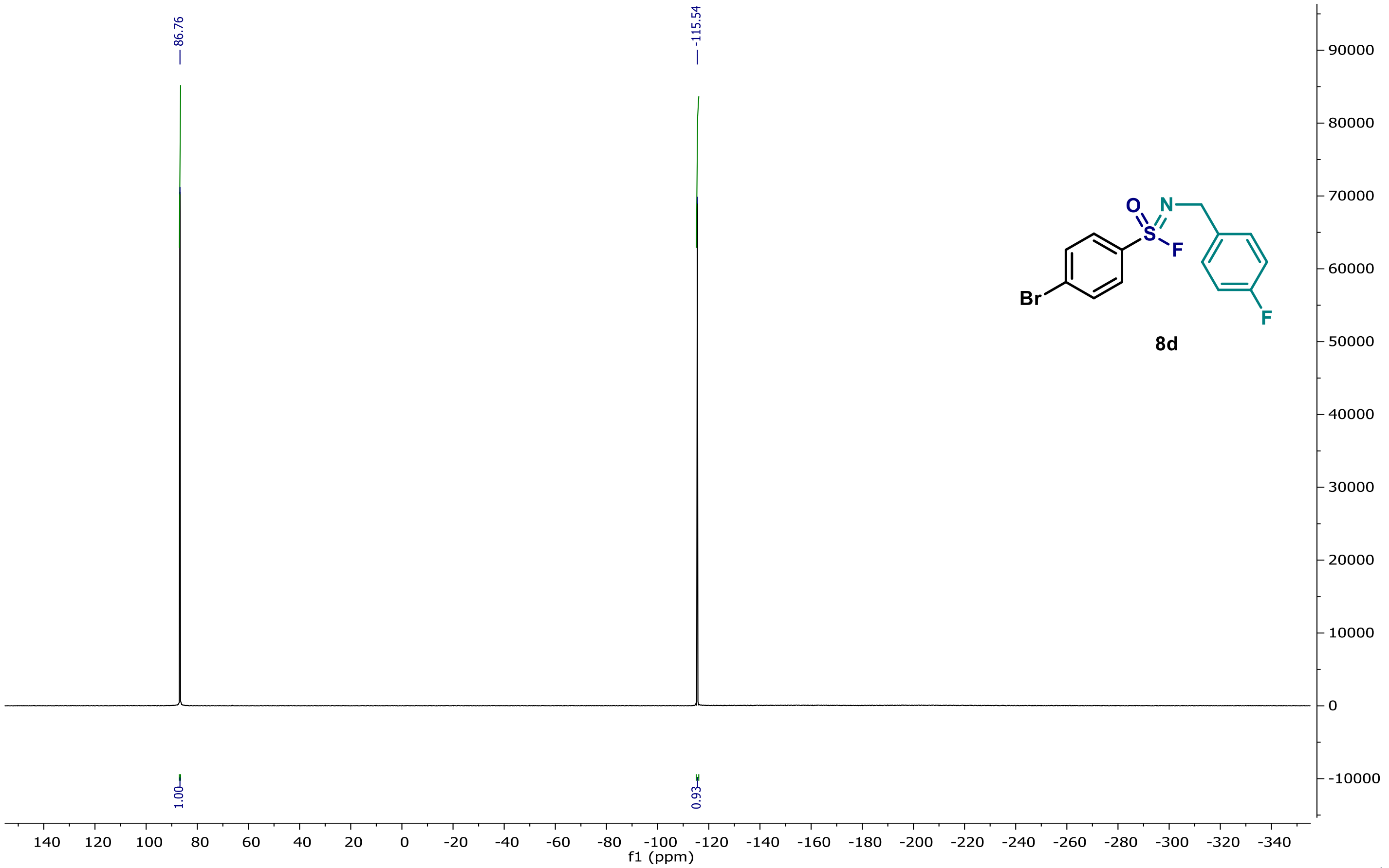

^{13}C Spectra (75 MHz, $CDCl_3$)

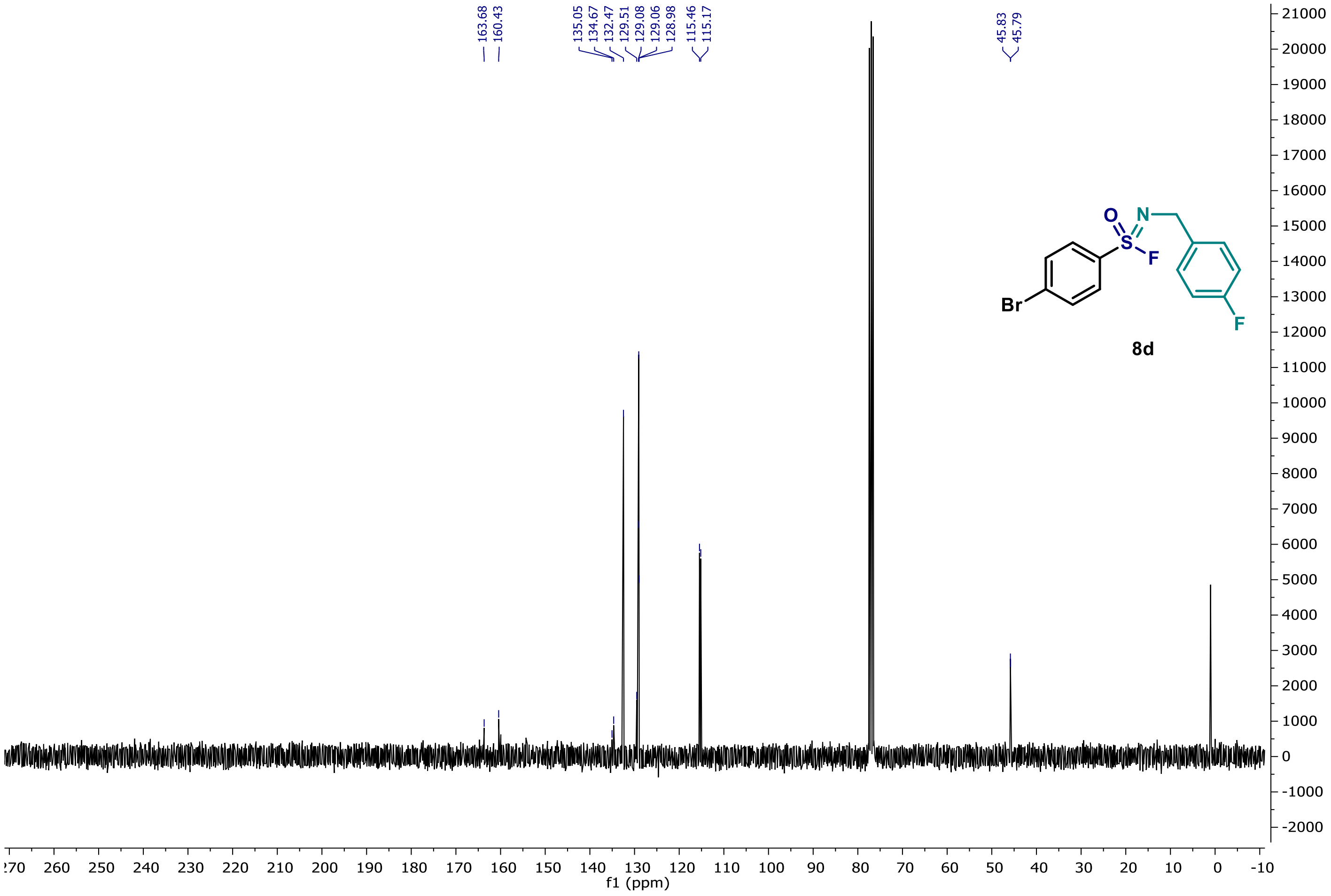

[1]H Spectra (300 MHz, $CDCl_3$)

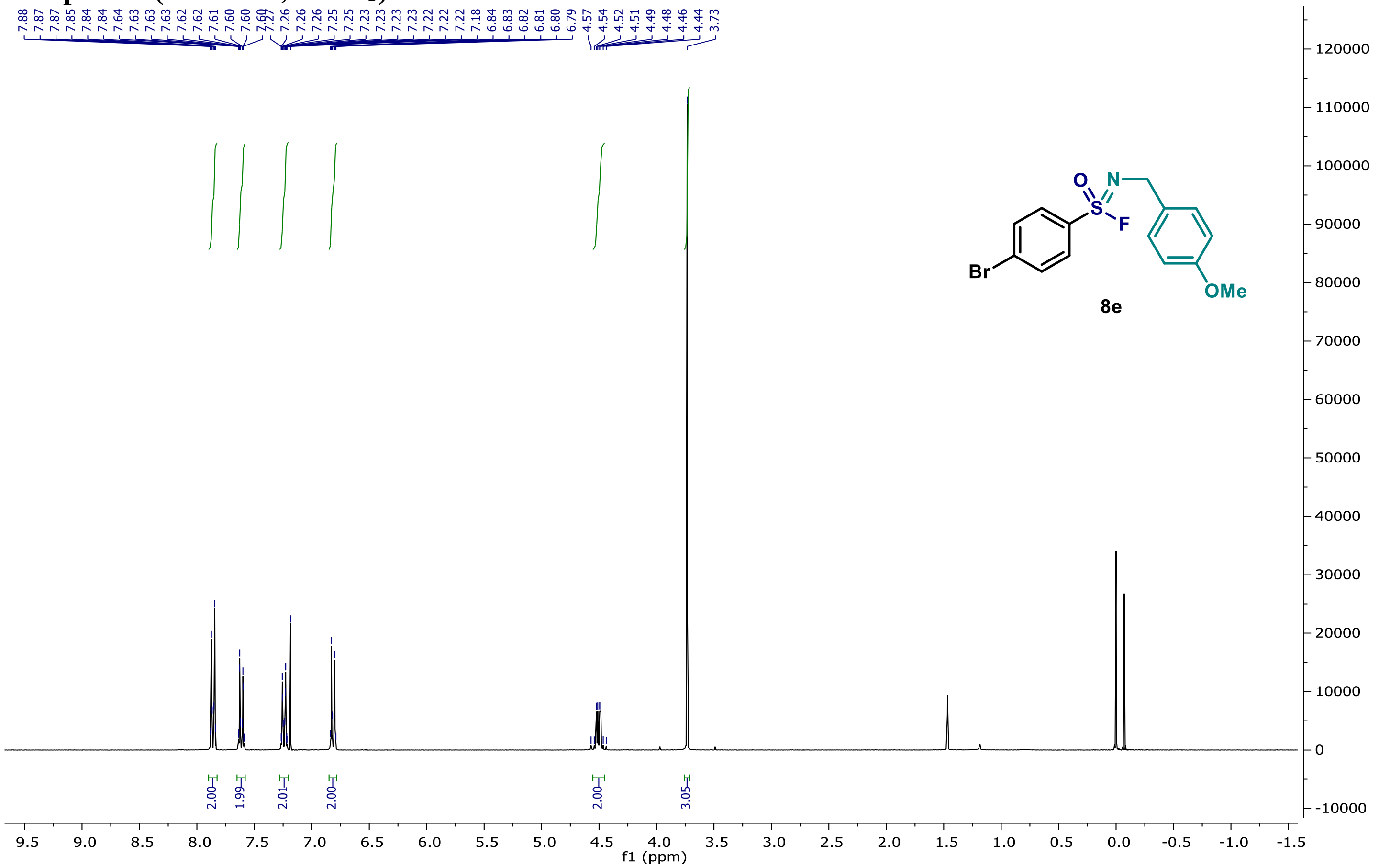

^{19}F{^{1}H} NMR (282 MHz, $CDCl_3$)

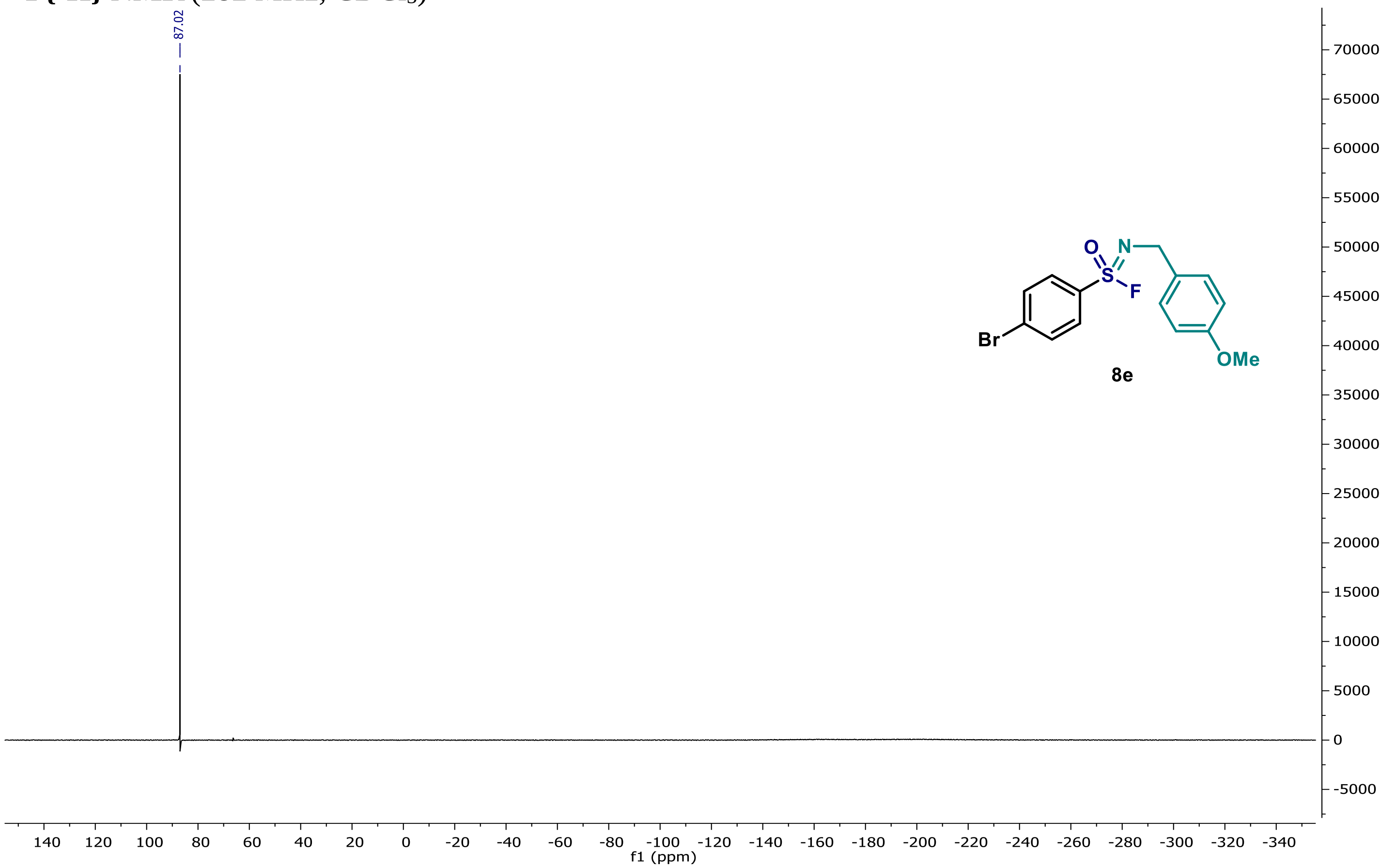

^{13}C Spectra (75 MHz, $CDCl_3$)

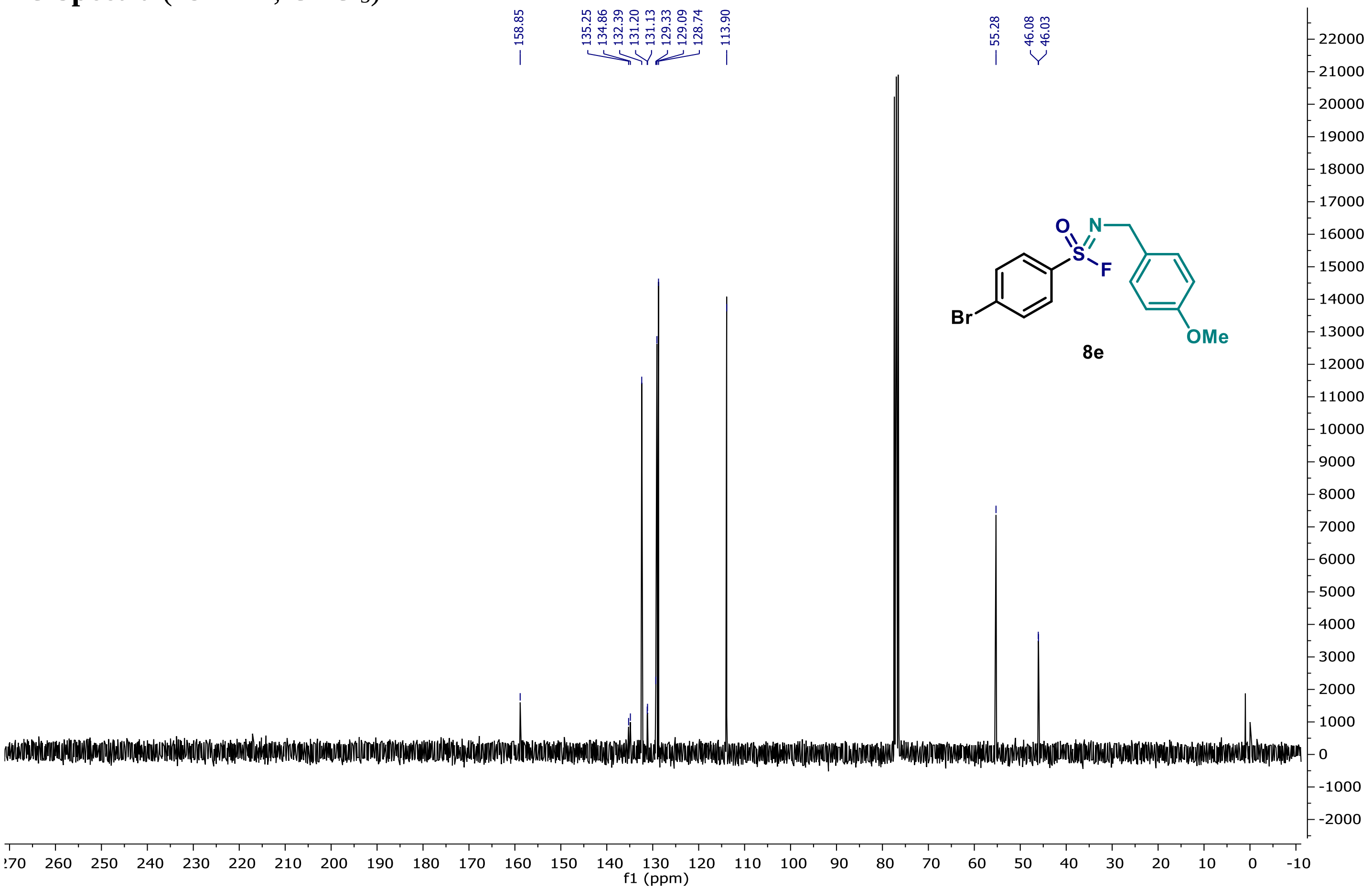

[1]H Spectra (300 MHz, $CDCl_3$)

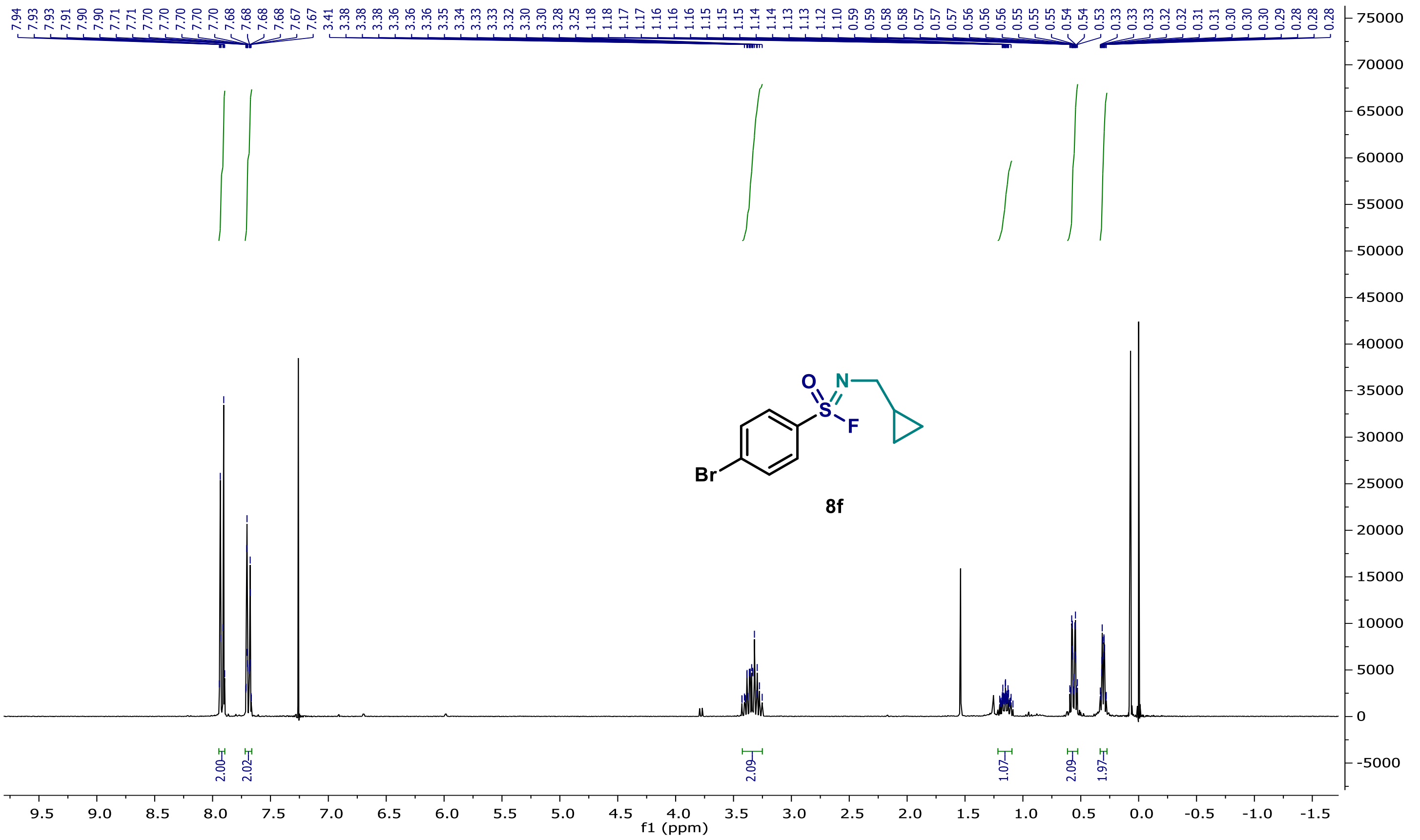

^{19}F{^{1}H} NMR (282 MHz, $CDCl_3$)

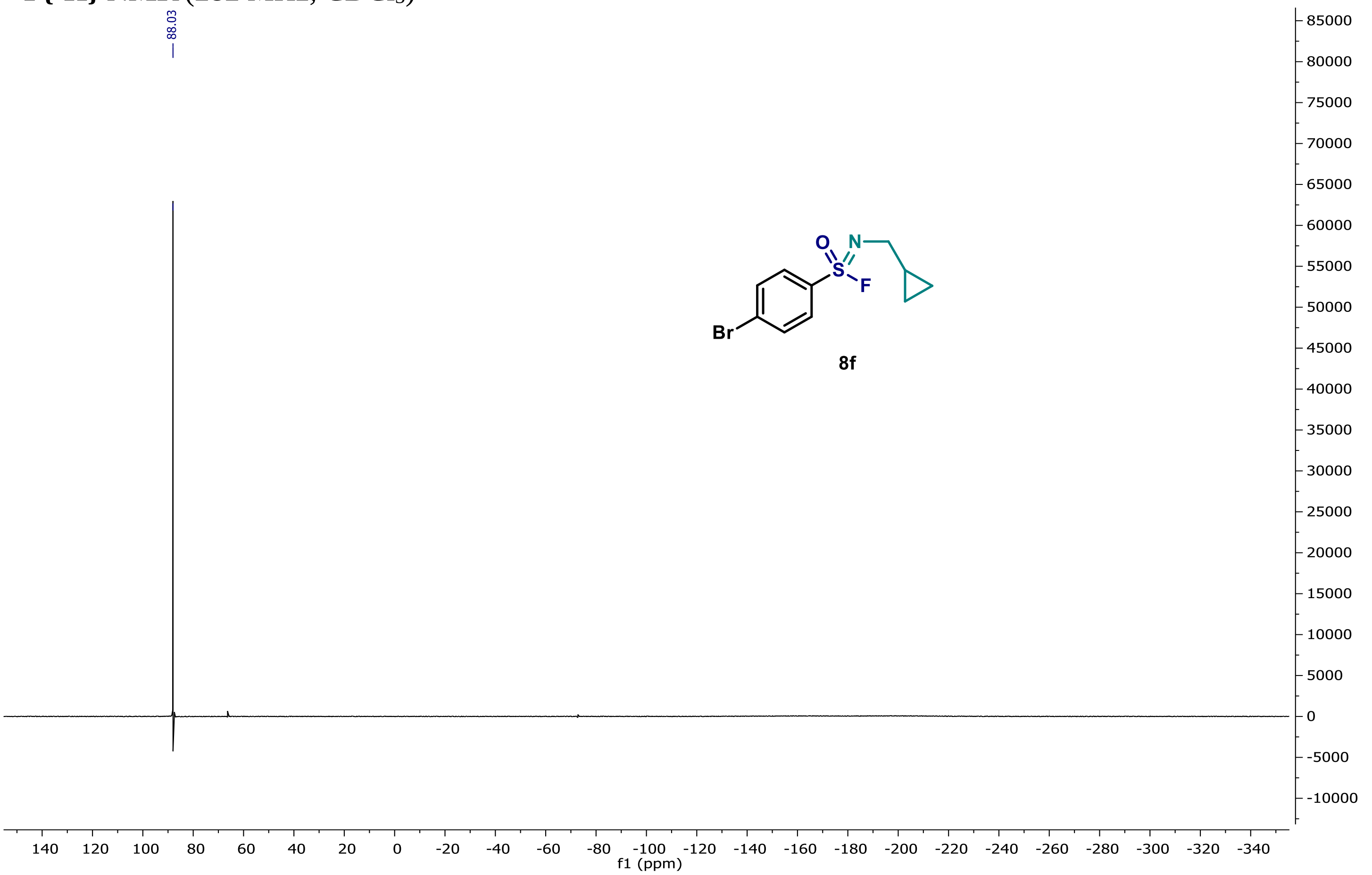

^{13}C Spectra (75 MHz, $CDCl_3$)

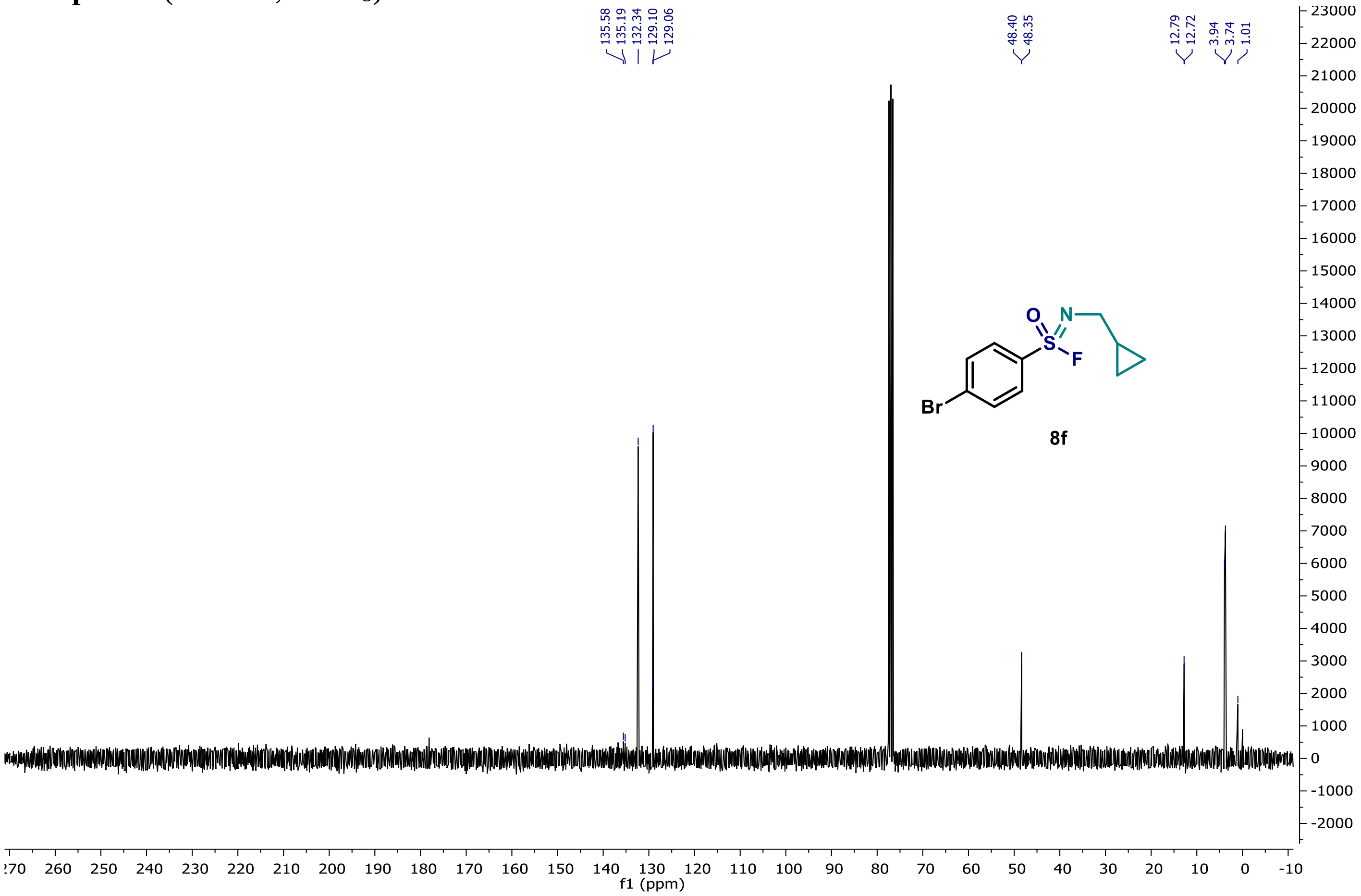

[1]H Spectra (300 MHz, $CDCl_3$)

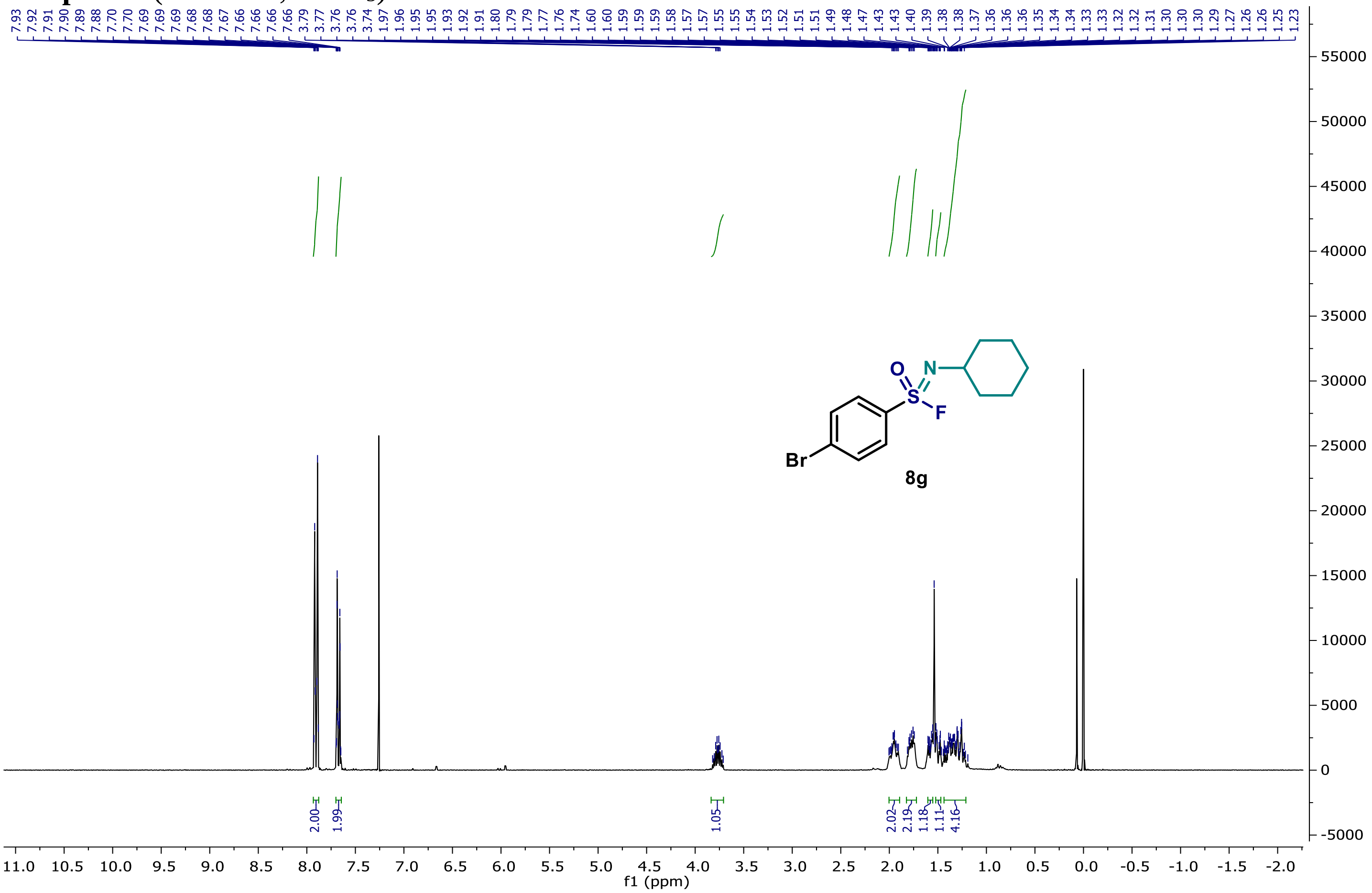

^{19}F{^{1}H} NMR (282 MHz, $CDCl_3$)

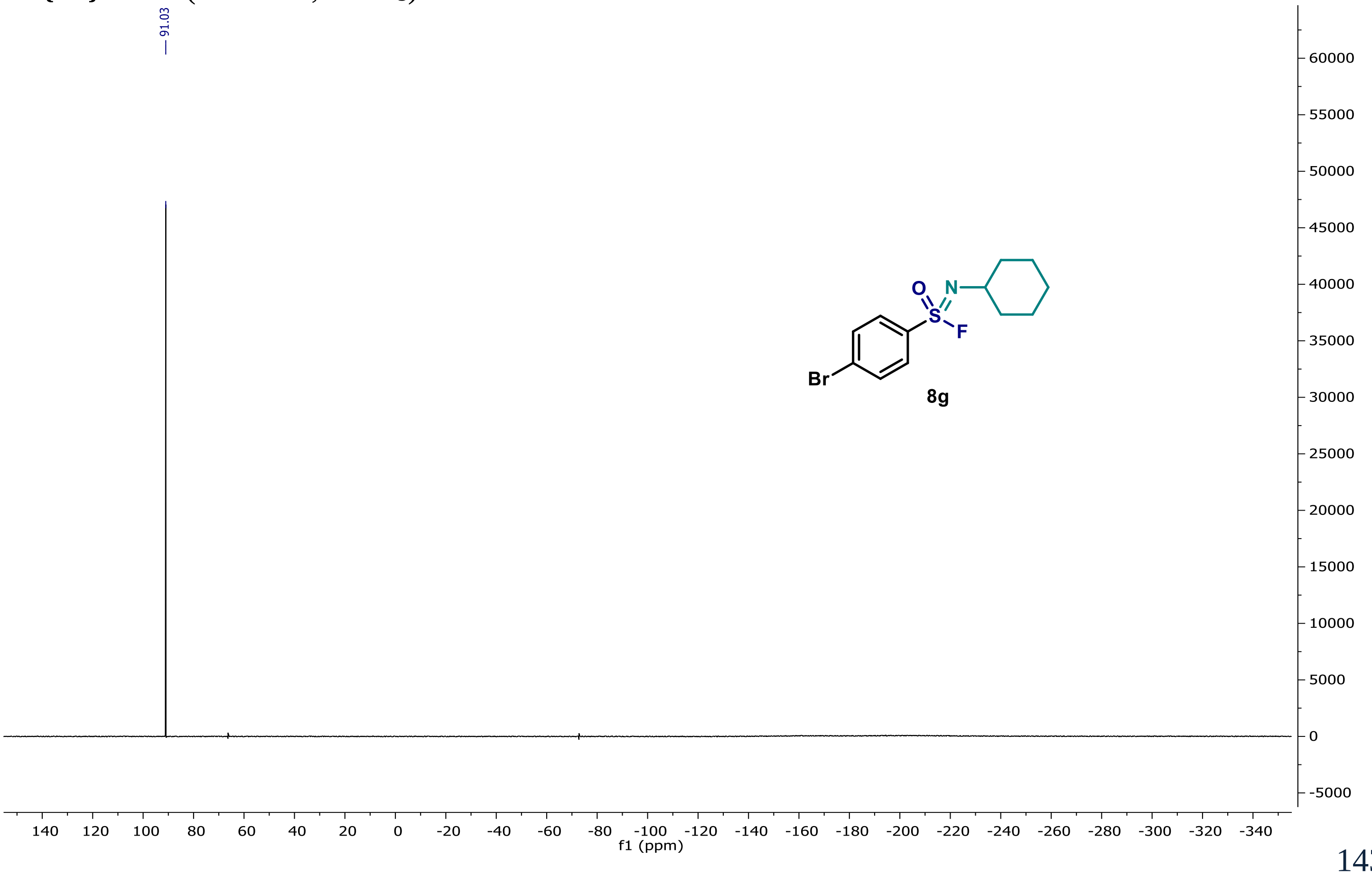

^{13}C Spectra (75 MHz, $CDCl_3$)

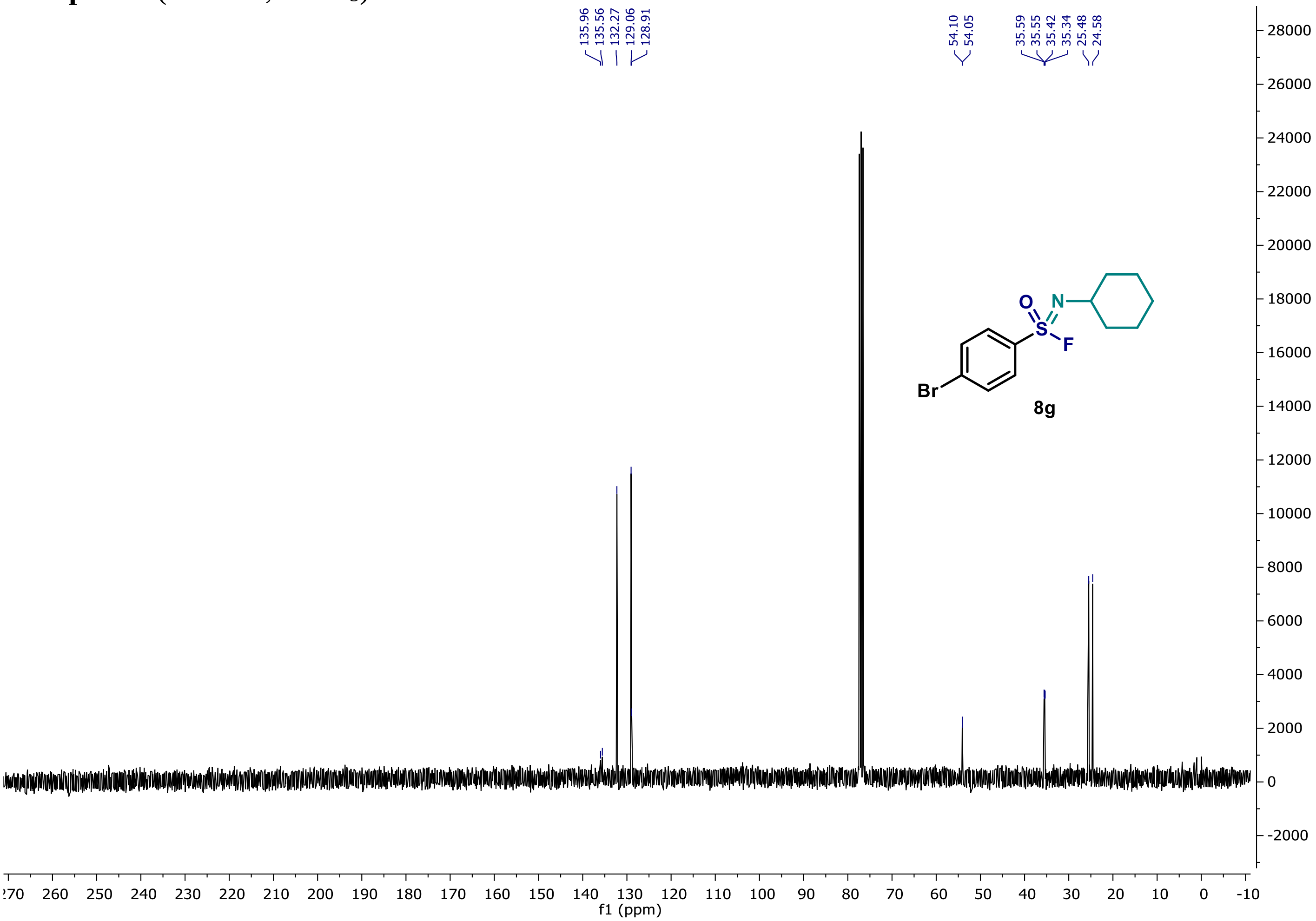

[1]H Spectra (300 MHz, $CDCl_3$)

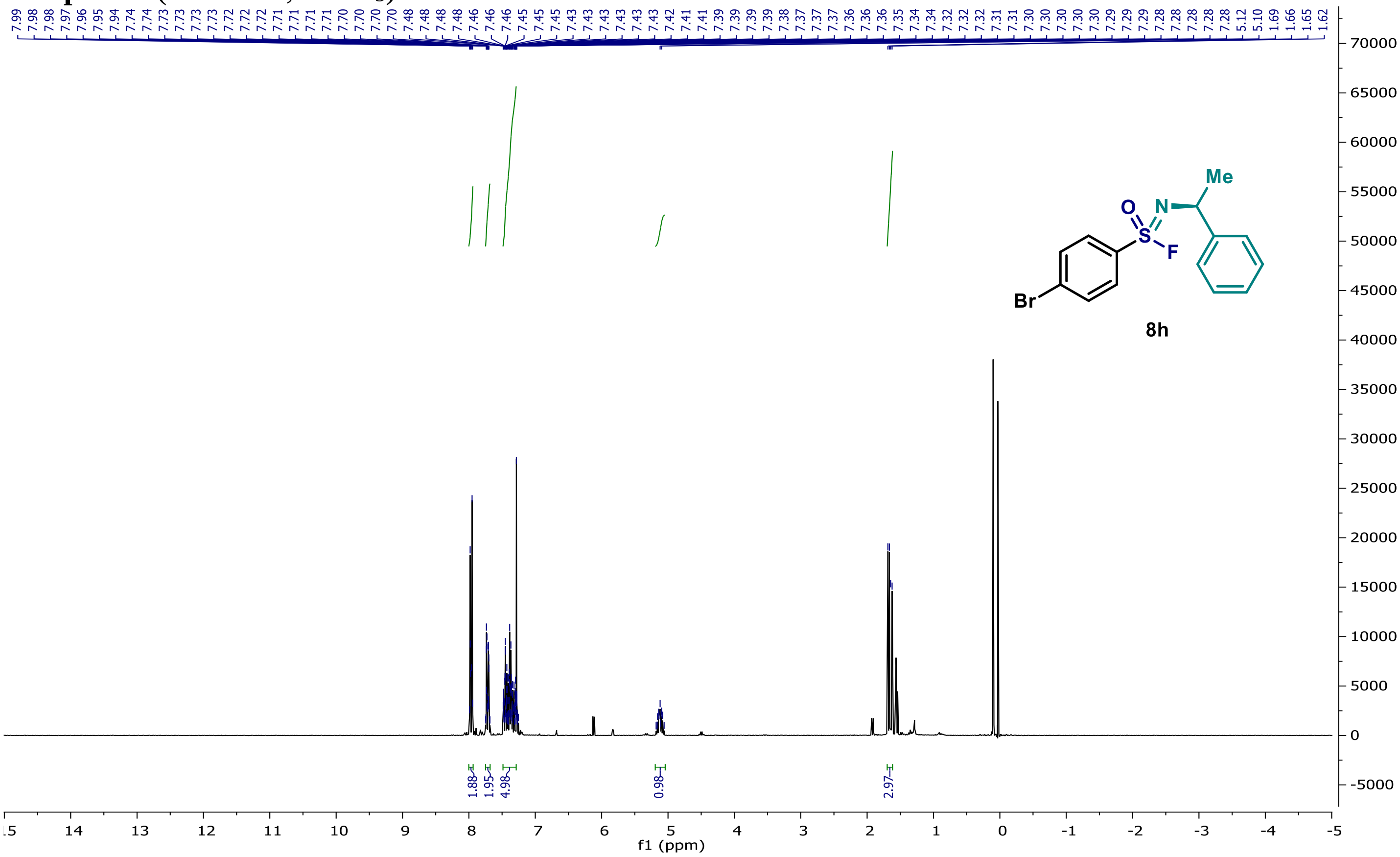

^{19}F{^{1}H} NMR (282 MHz, $CDCl_3$)

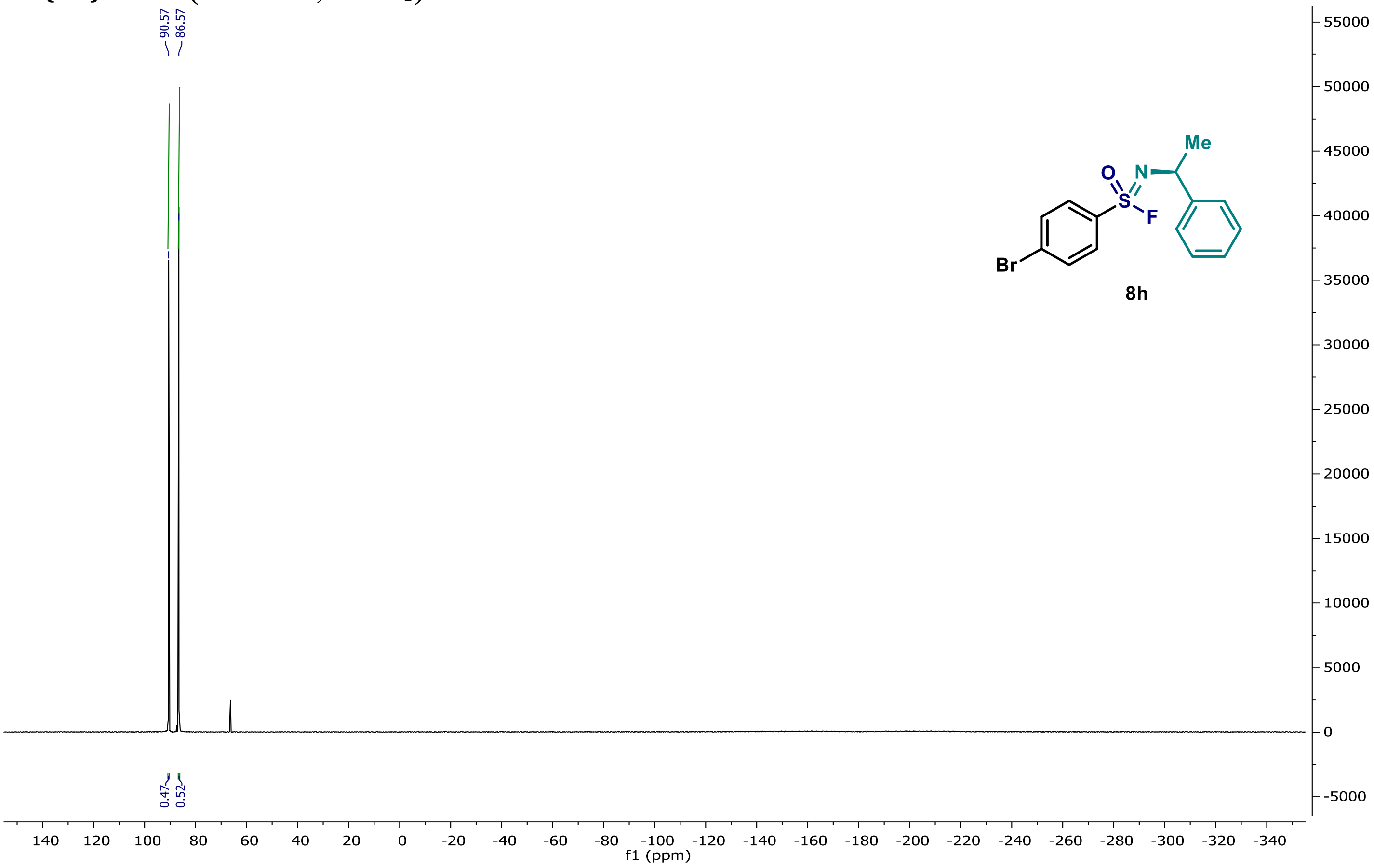

^{13}C Spectra (75 MHz, $CDCl_3$)

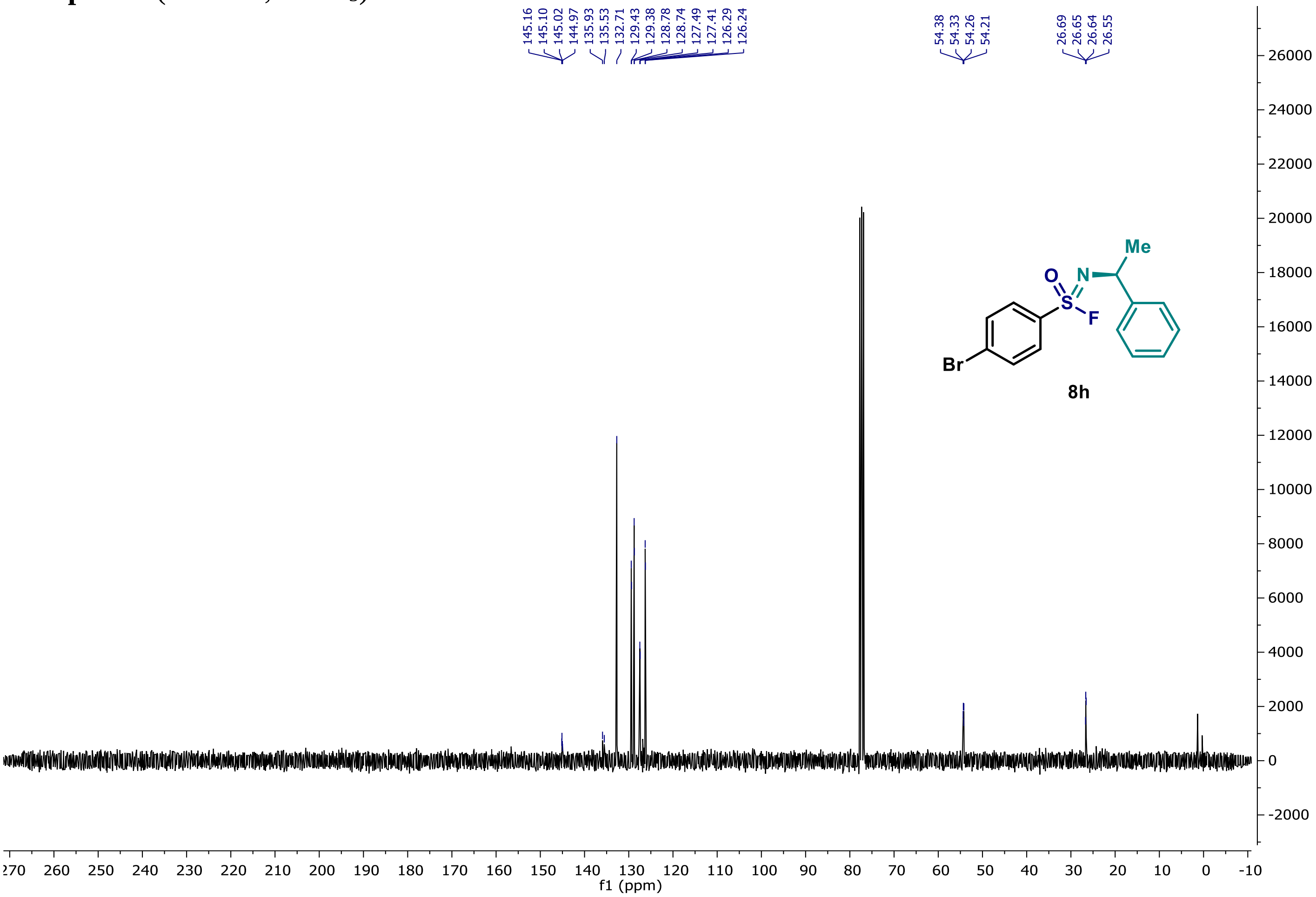

^{1}H Spectra (300 MHz, $CDCl_3$)

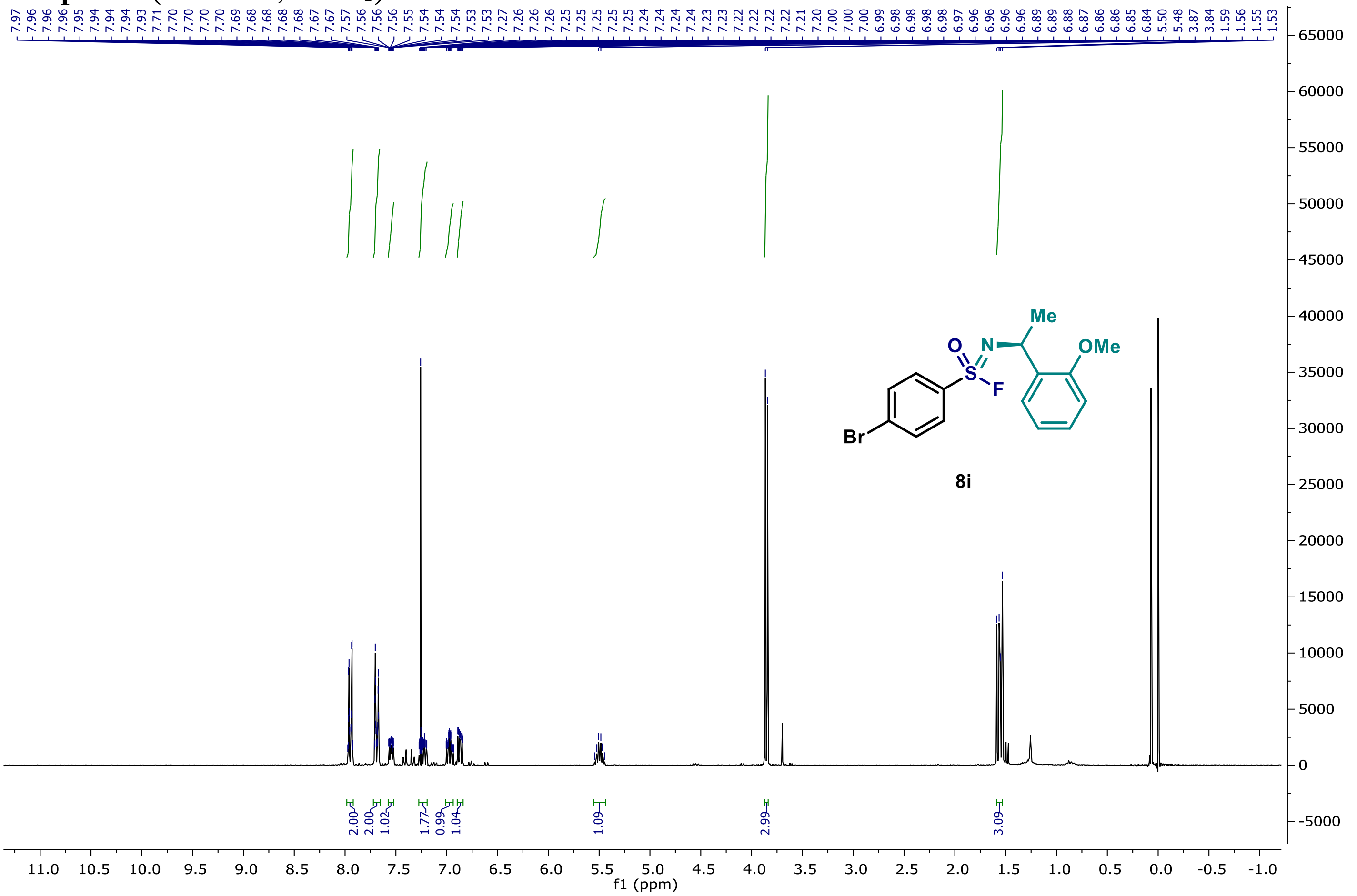

^{19}F{^{1}H} NMR (282 MHz, $CDCl_3$)

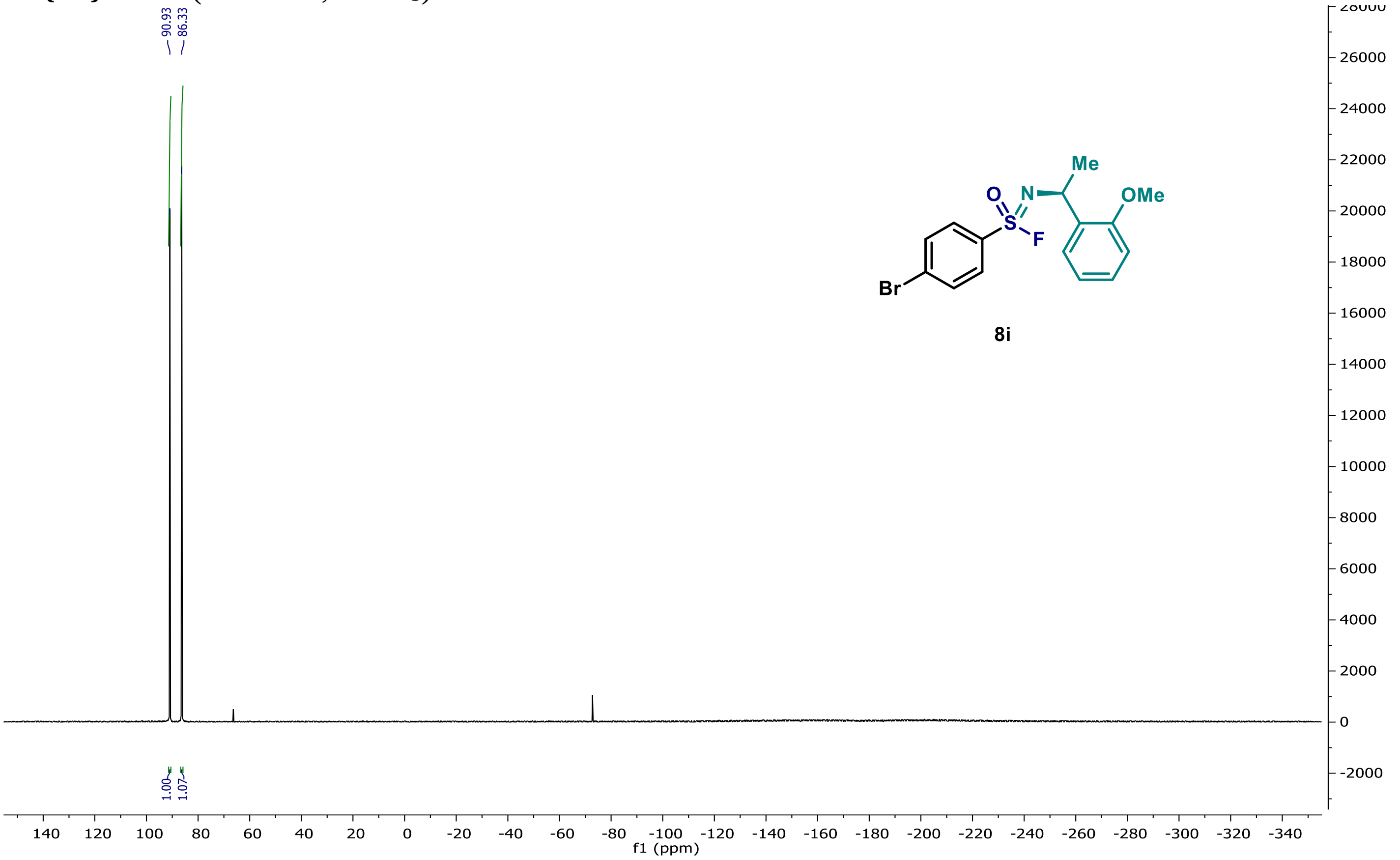

^{13}C Spectra (75 MHz, $CDCl_3$)

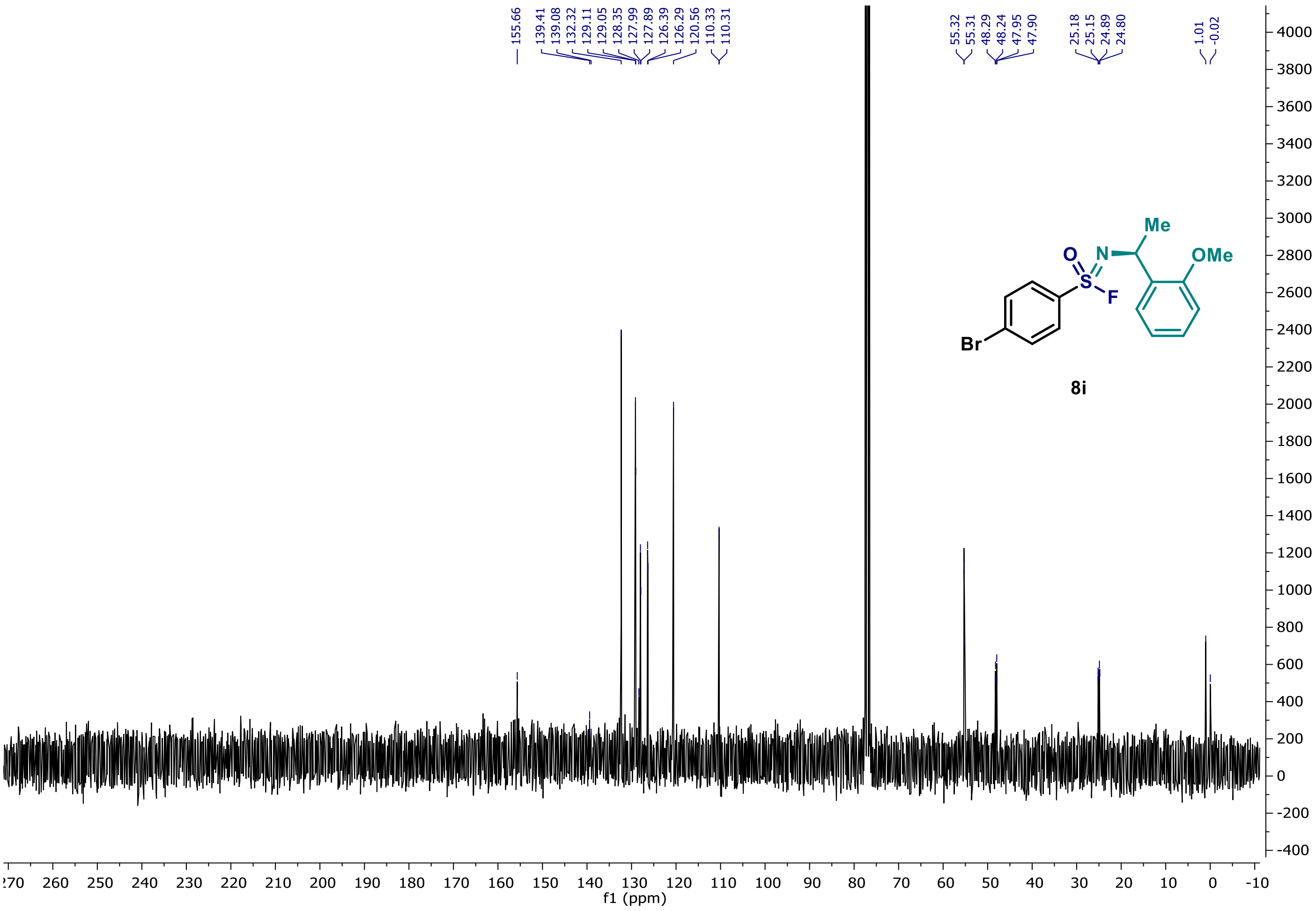

^{1}H Spectra (300 MHz, $CDCl_3$)

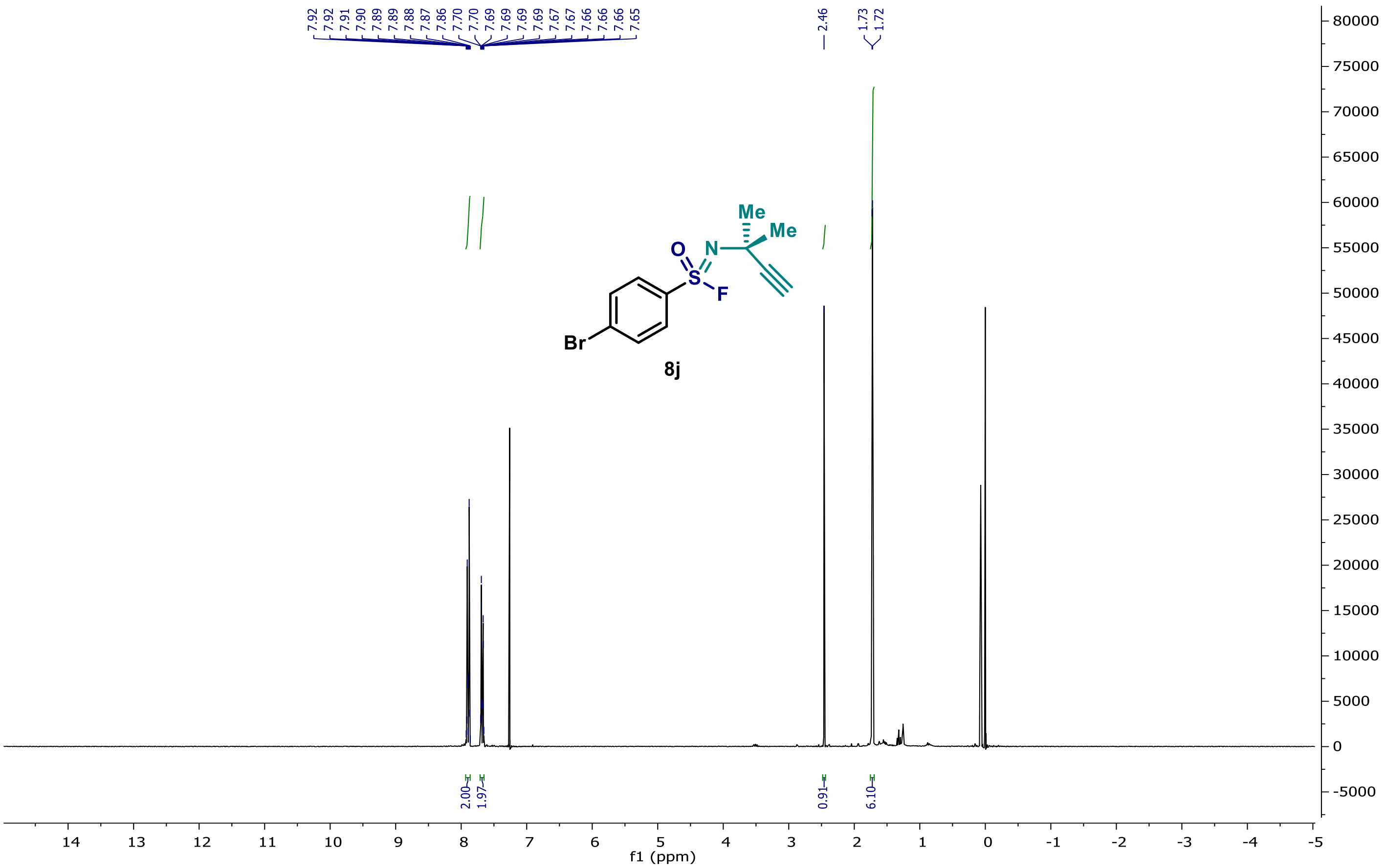

^{19}F{^{1}H} NMR (282 MHz, $CDCl_3$)

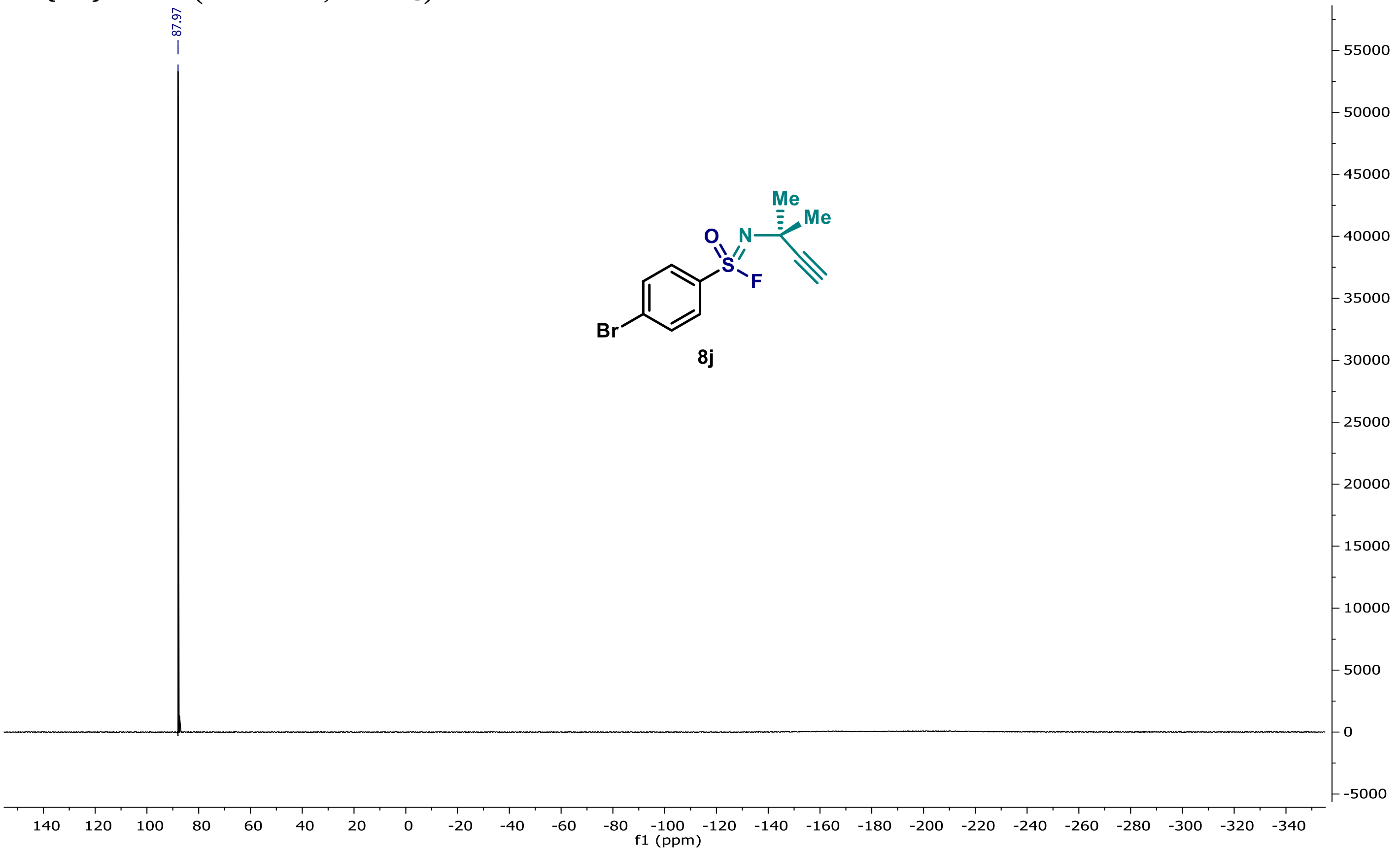

^{13}C Spectra (75 MHz, $CDCl_3$)

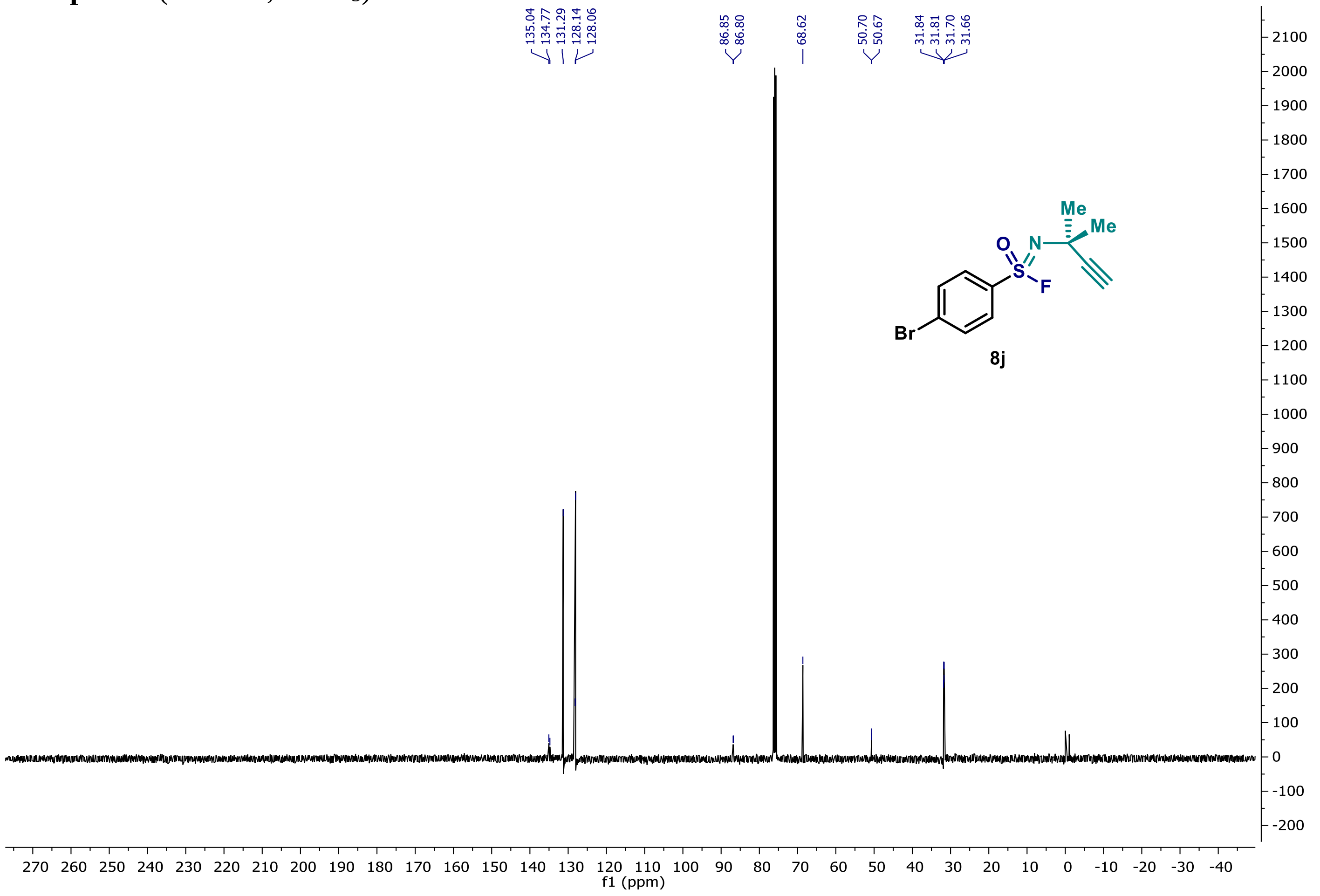

^{1}H Spectra (300 MHz, $CDCl_3$)

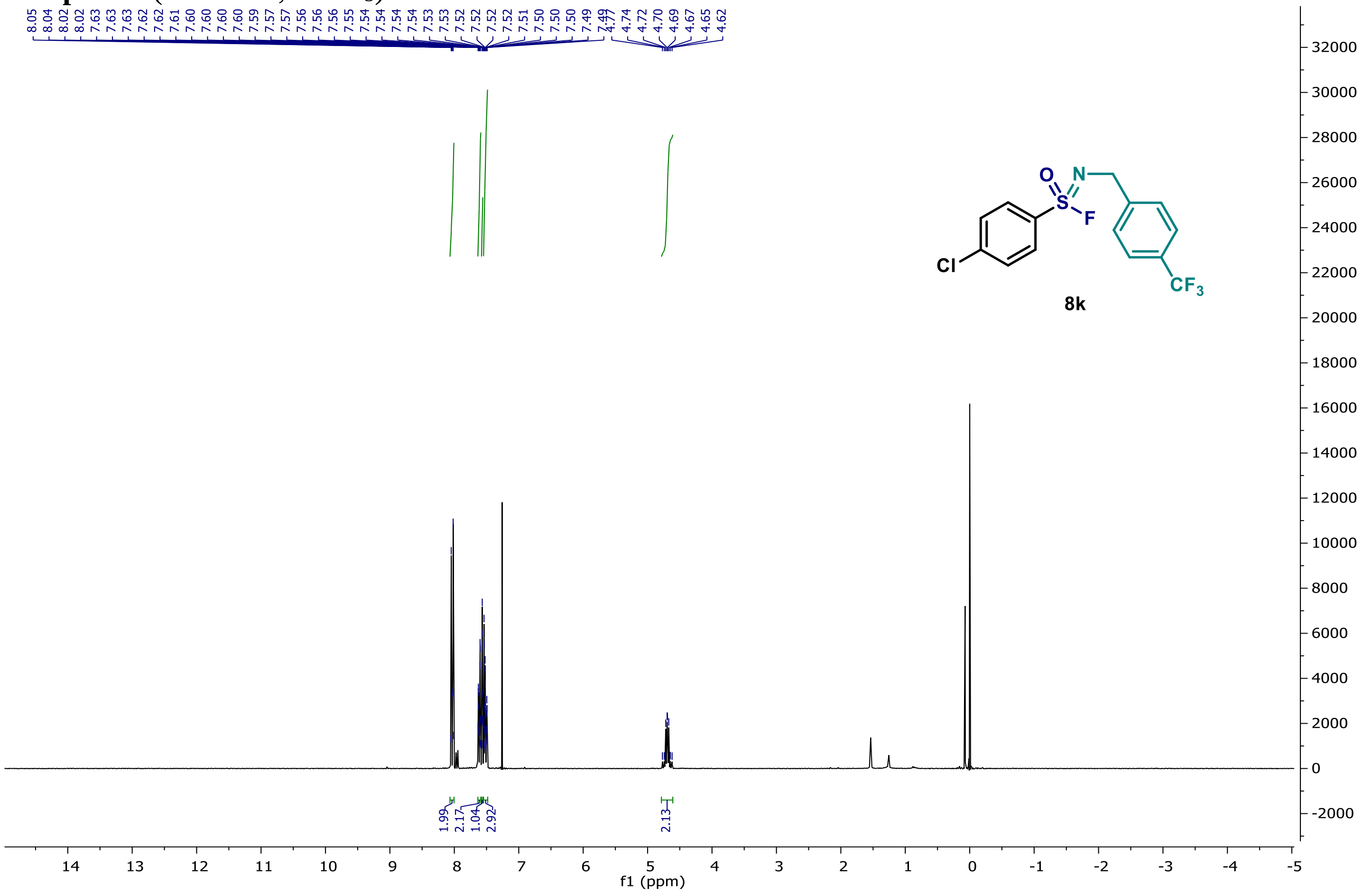

^{19}F{^{1}H} NMR (282 MHz, $CDCl_3$)

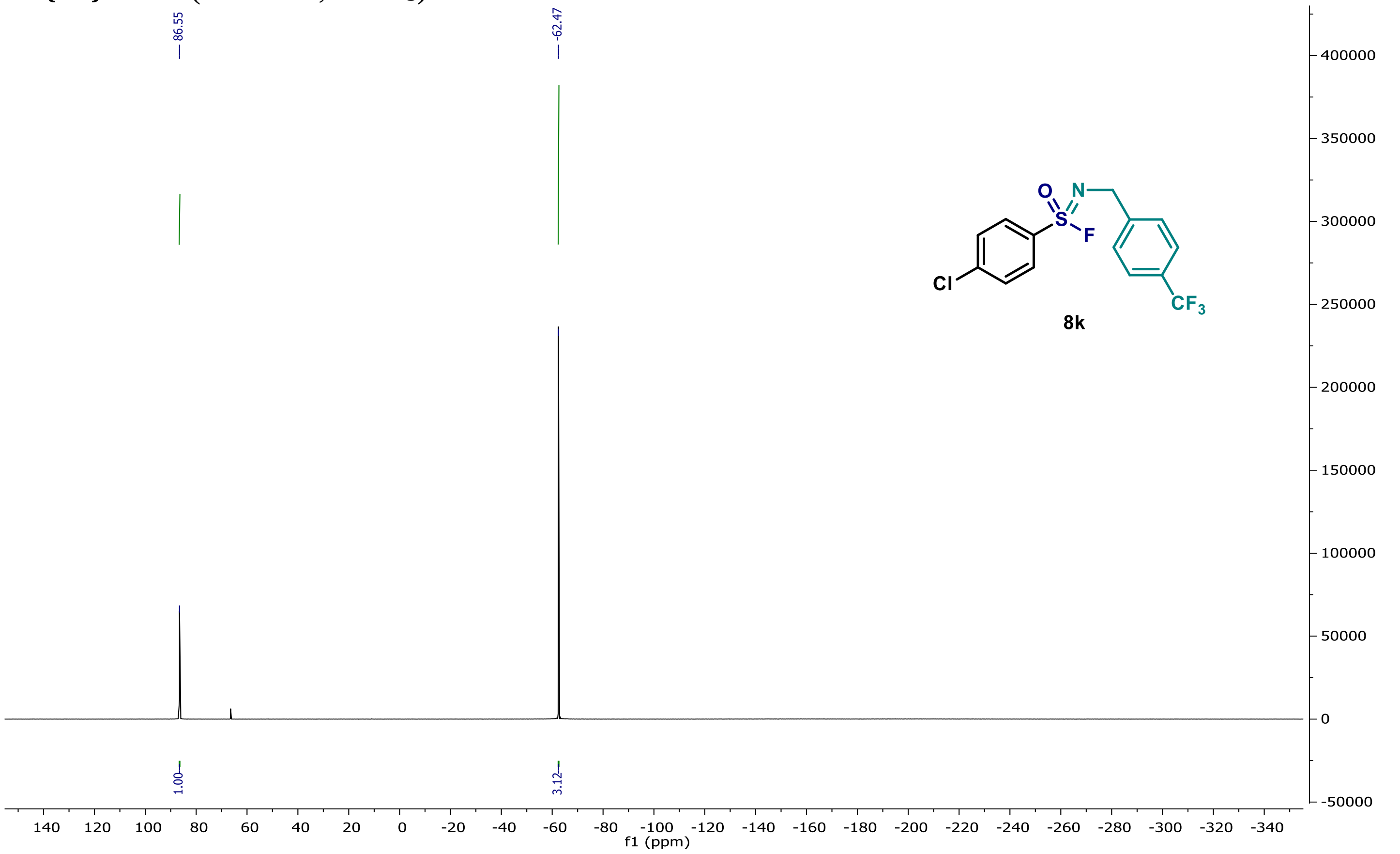

[13]C Spectra (75 MHz, $CDCl_3$)

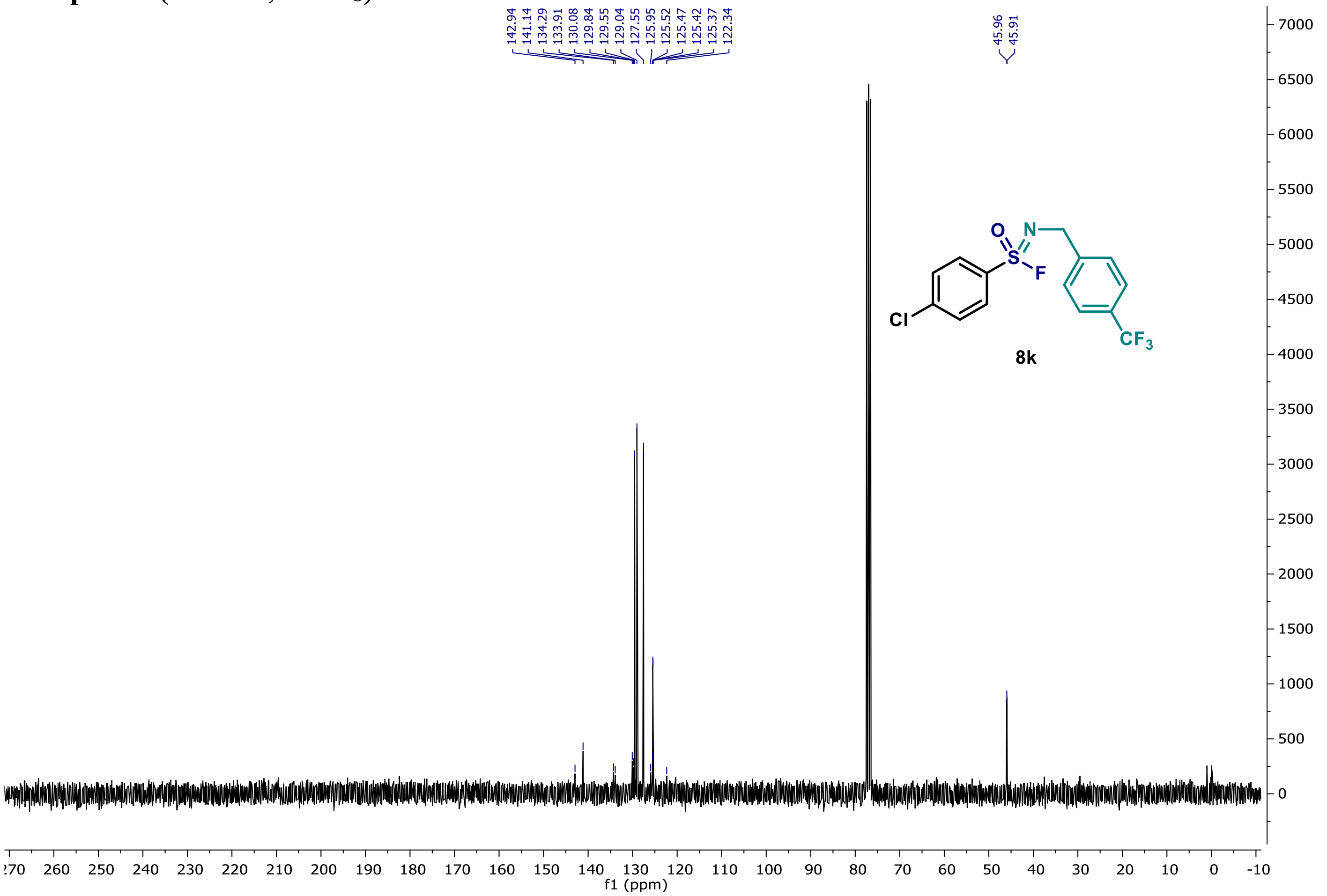

[1]H Spectra (300 MHz, $CDCl_3$)

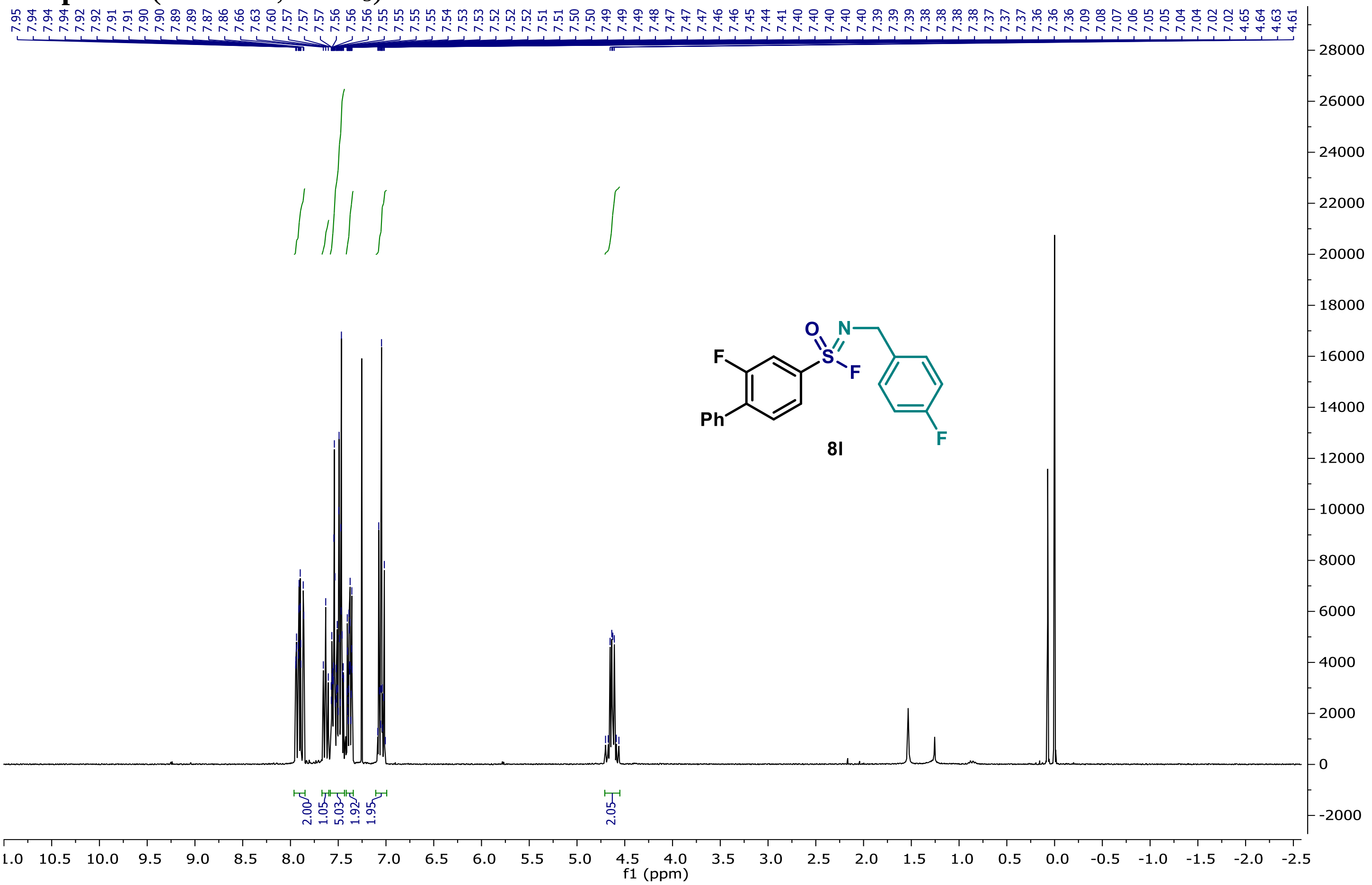

^{19}F{^{1}H} NMR (282 MHz, $CDCl_3$)

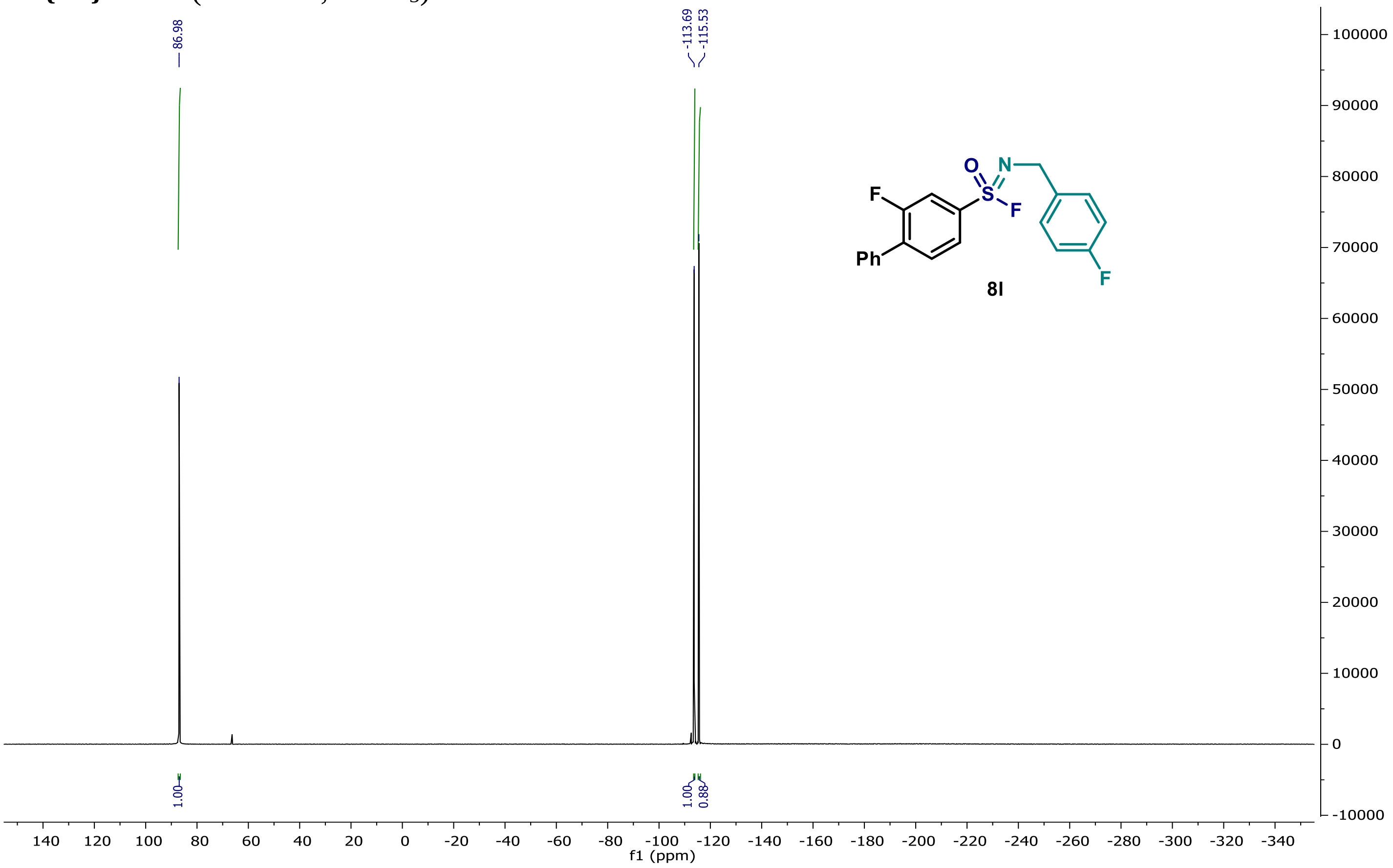

^{13}C Spectra (75 MHz, $CDCl_3$)

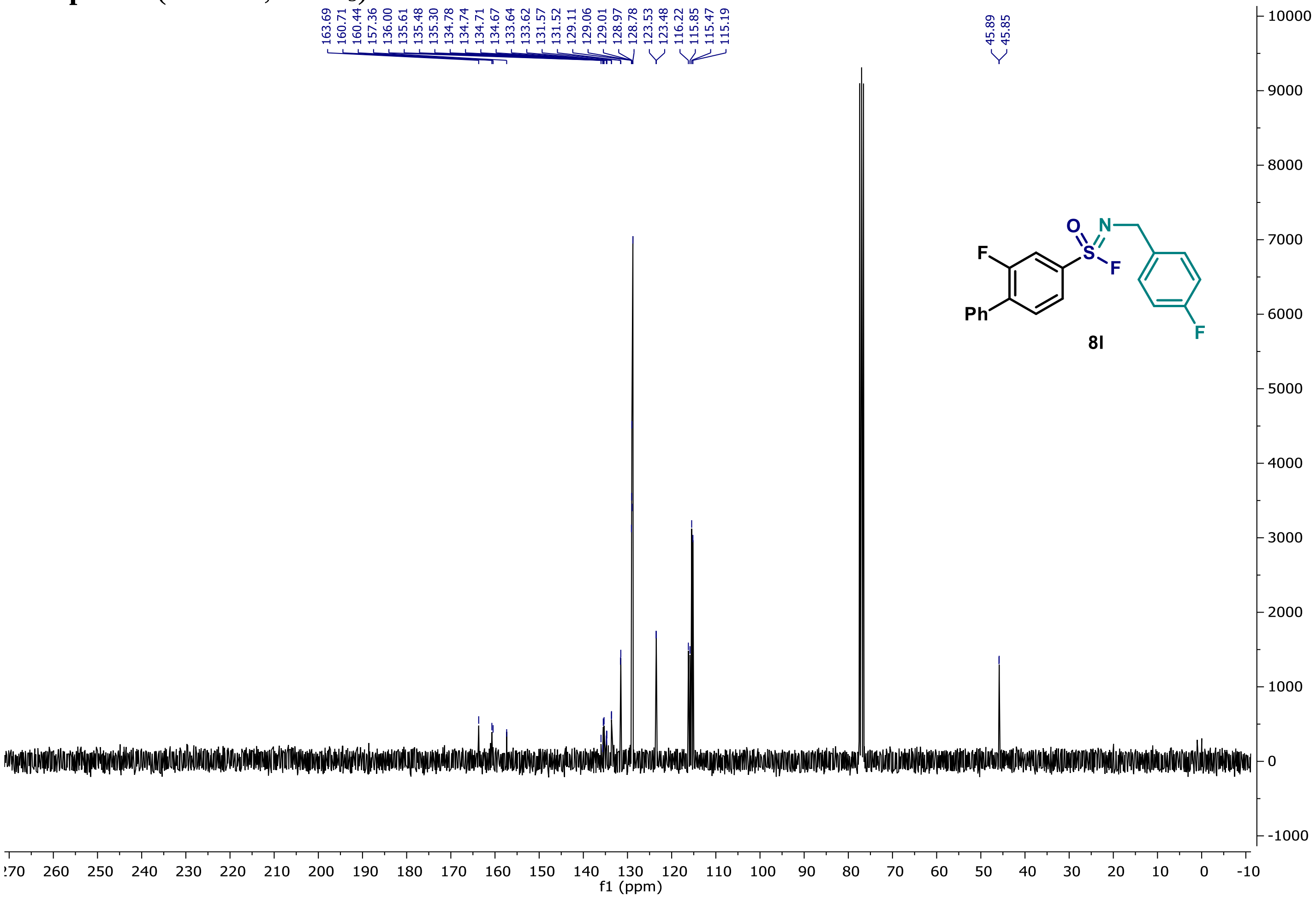

[1]H Spectra (300 MHz, $CDCl_3$)

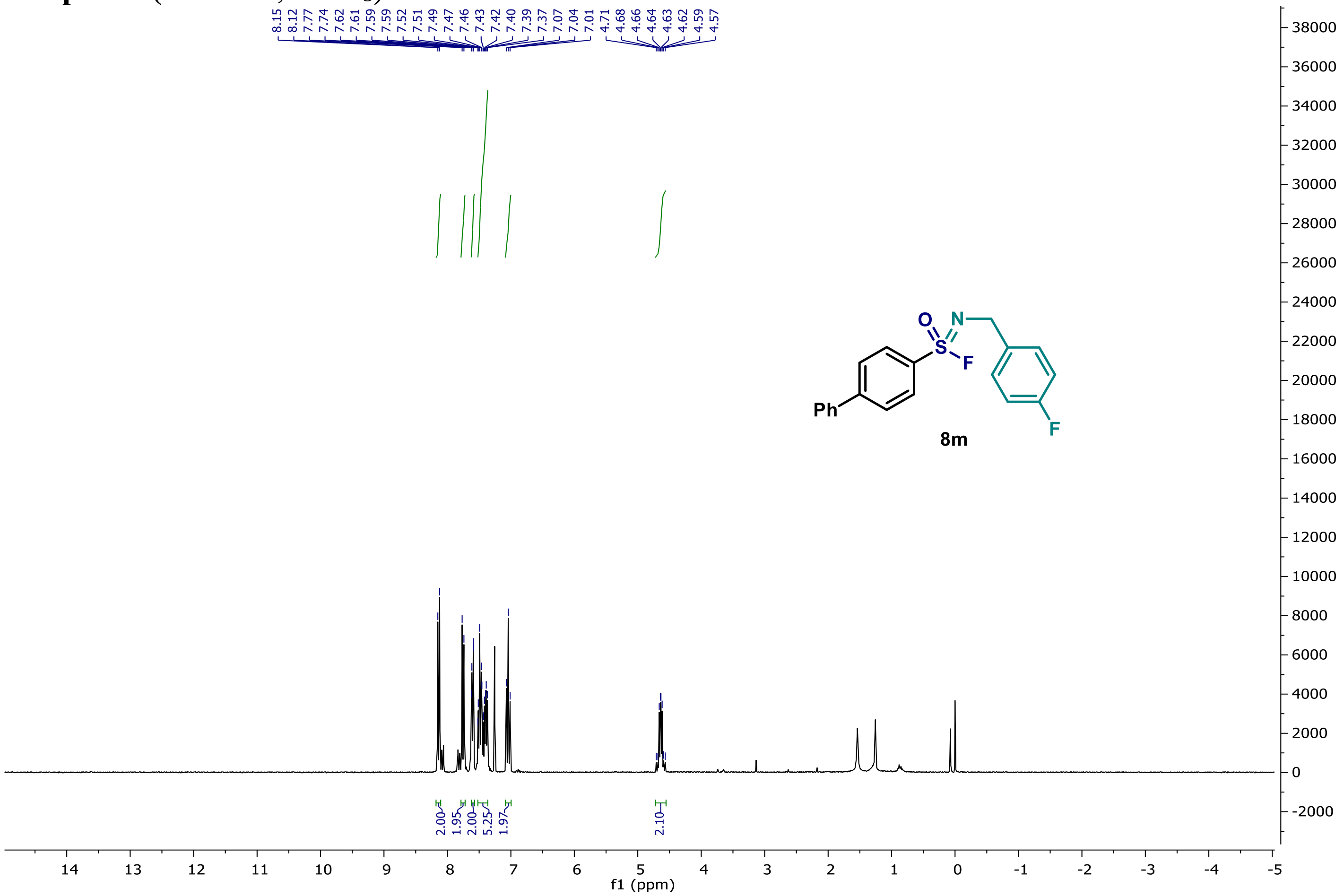

^{19}F{^{1}H} NMR (282 MHz, $CDCl_3$)

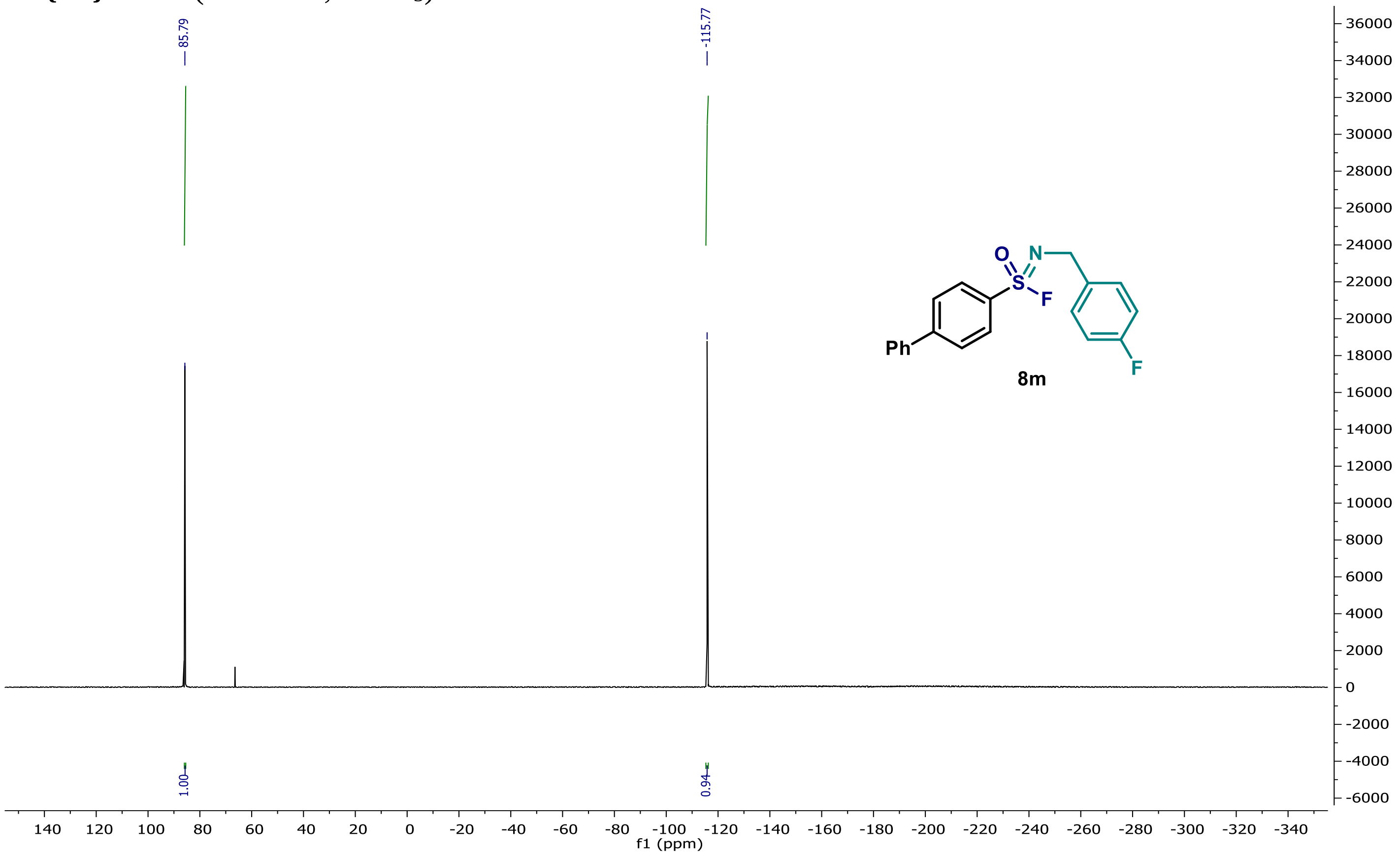

^{13}C Spectra (75 MHz, $CDCl_3$)

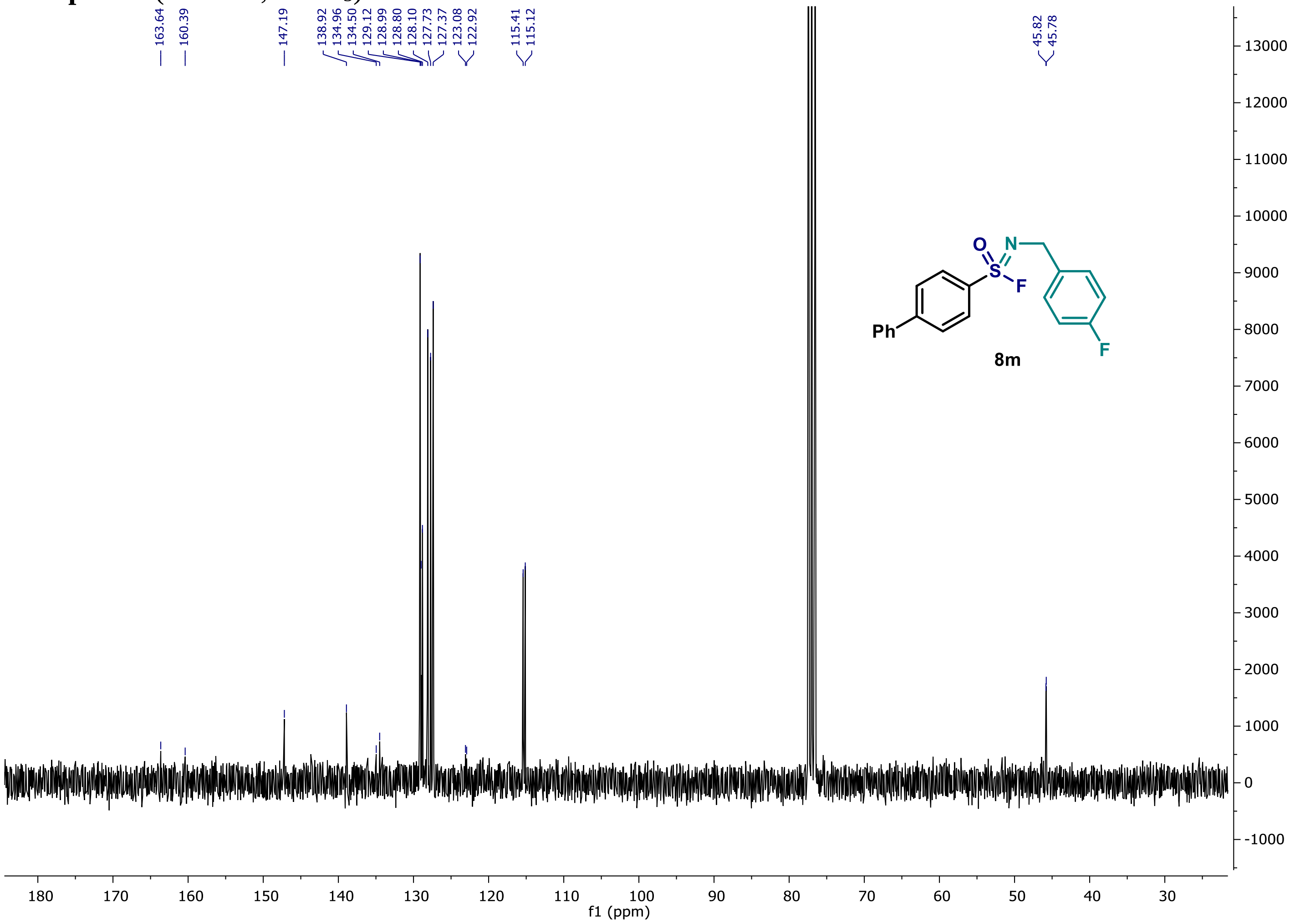

^{1}H Spectra (300 MHz, $CDCl_3$)

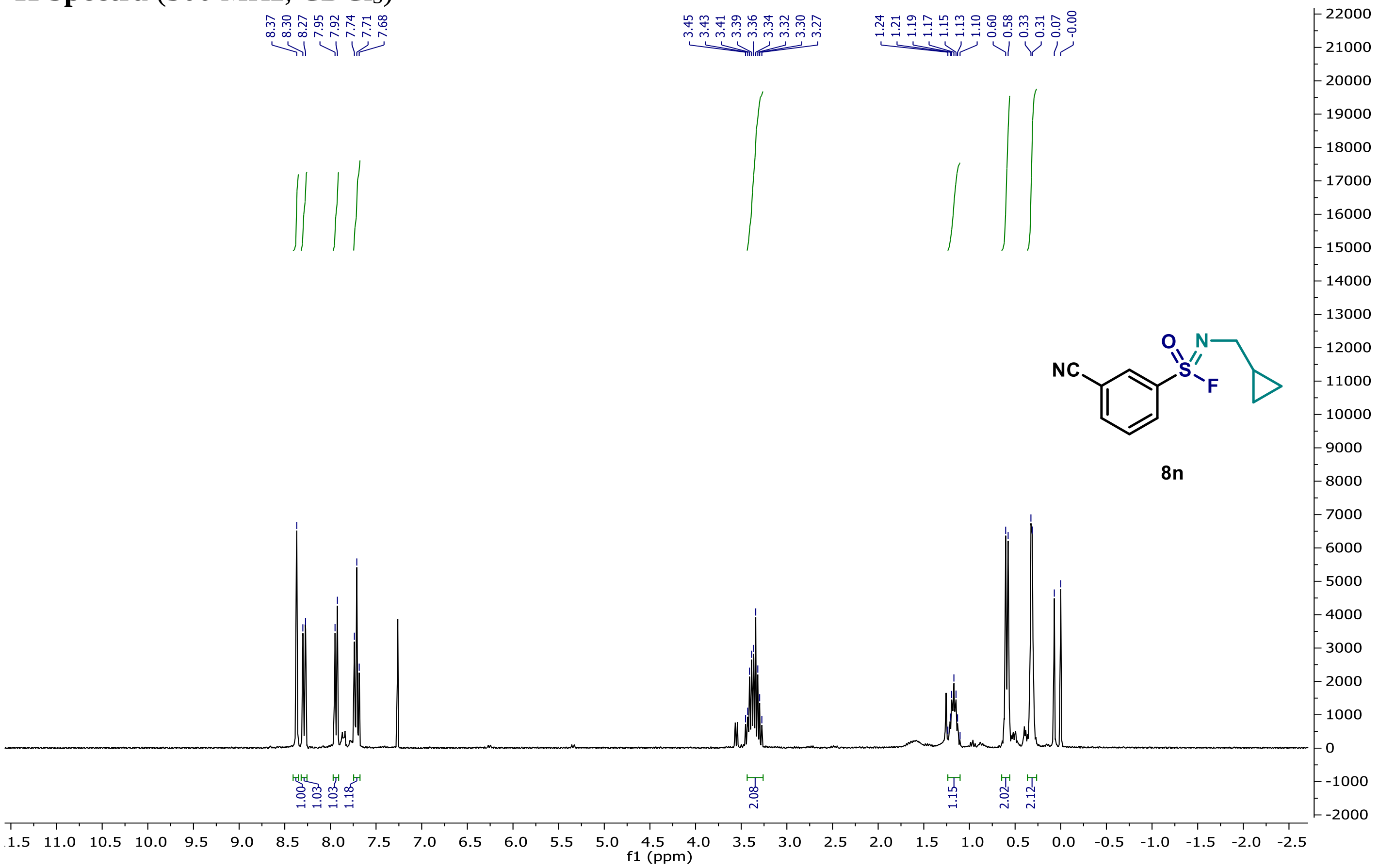

^{19}F{^{1}H} NMR (282 MHz, $CDCl_3$)

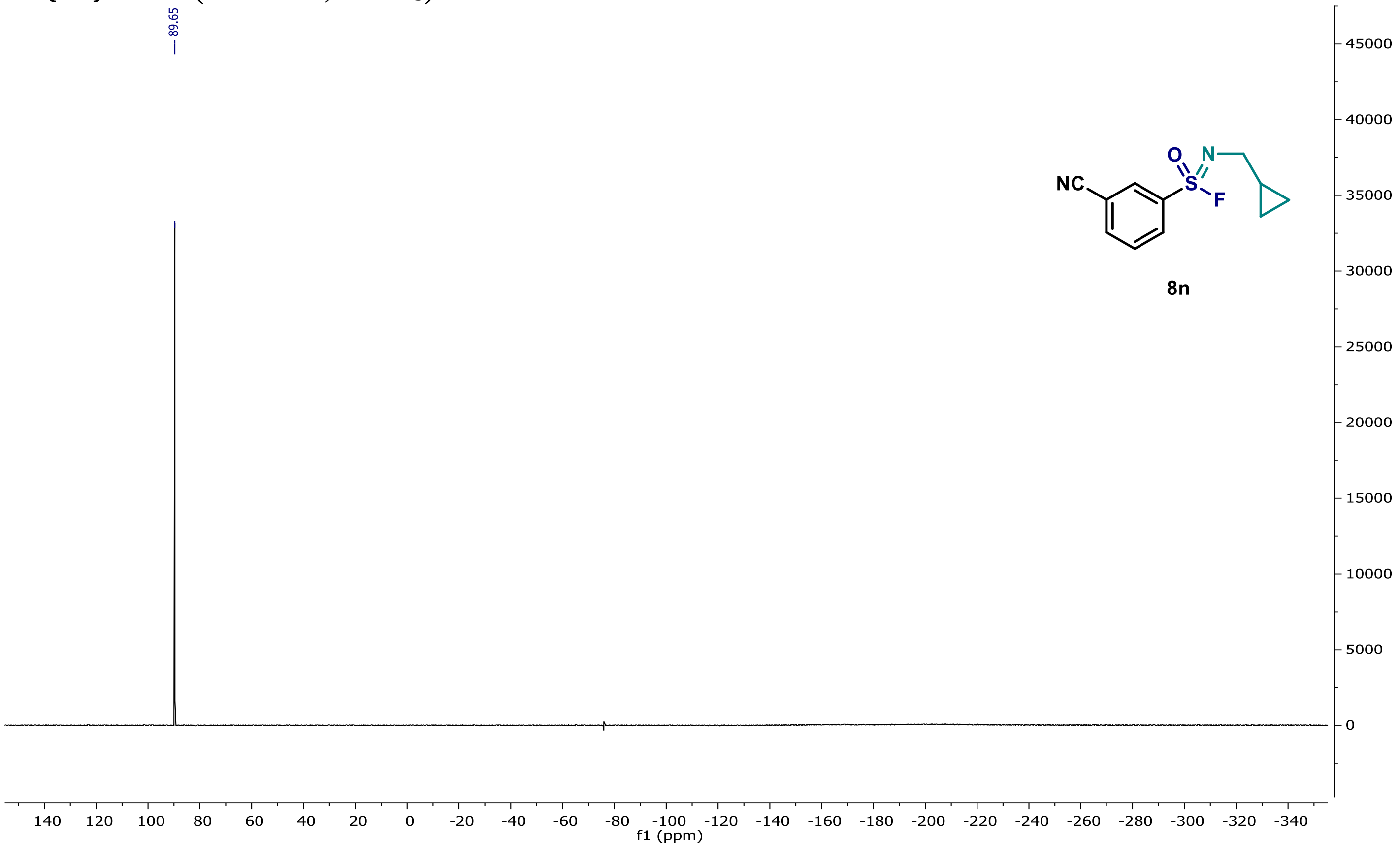

^{13}C Spectra (75 MHz, $CDCl_3$)

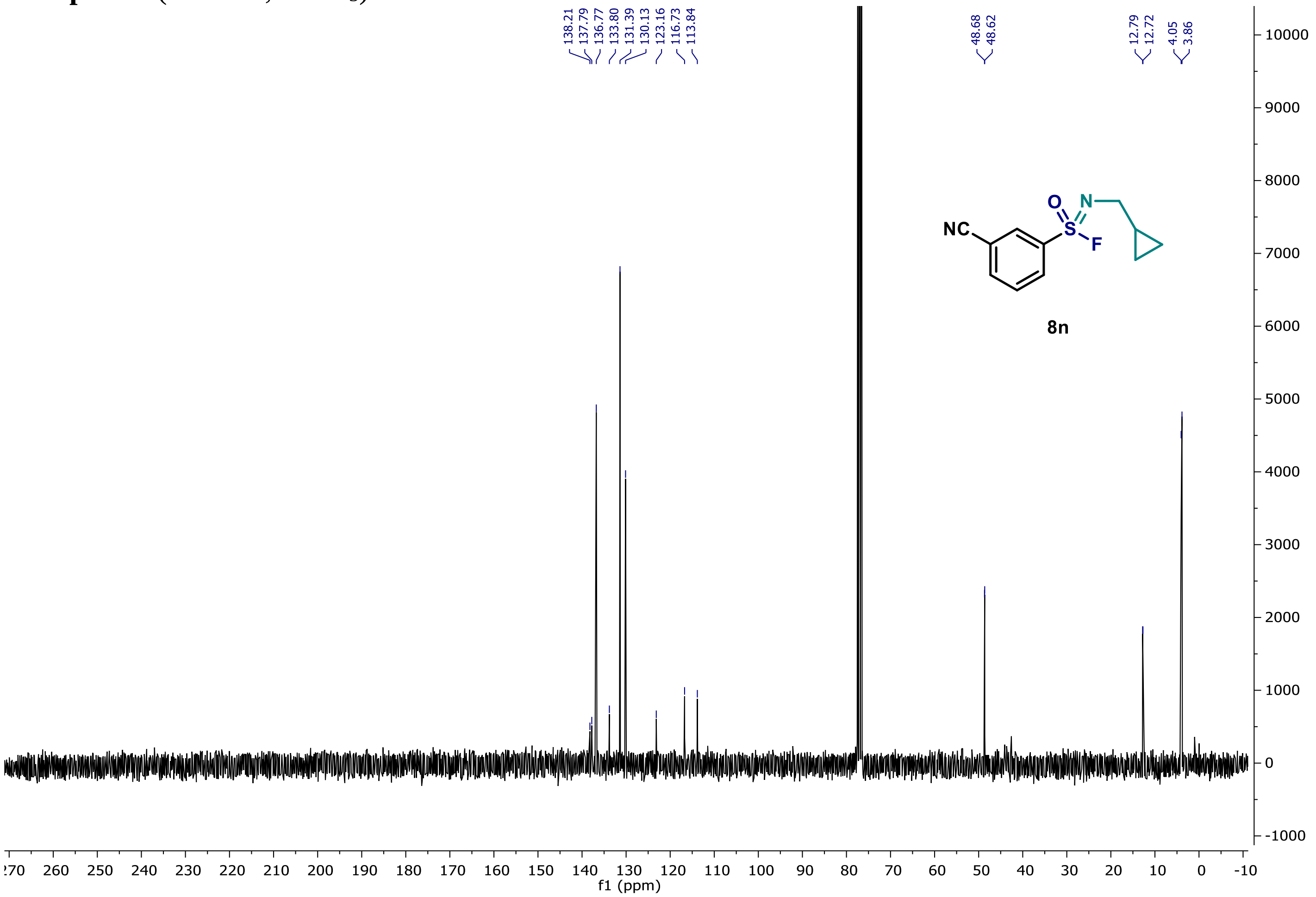

^{1}H Spectra (300 MHz, $CDCl_3$)

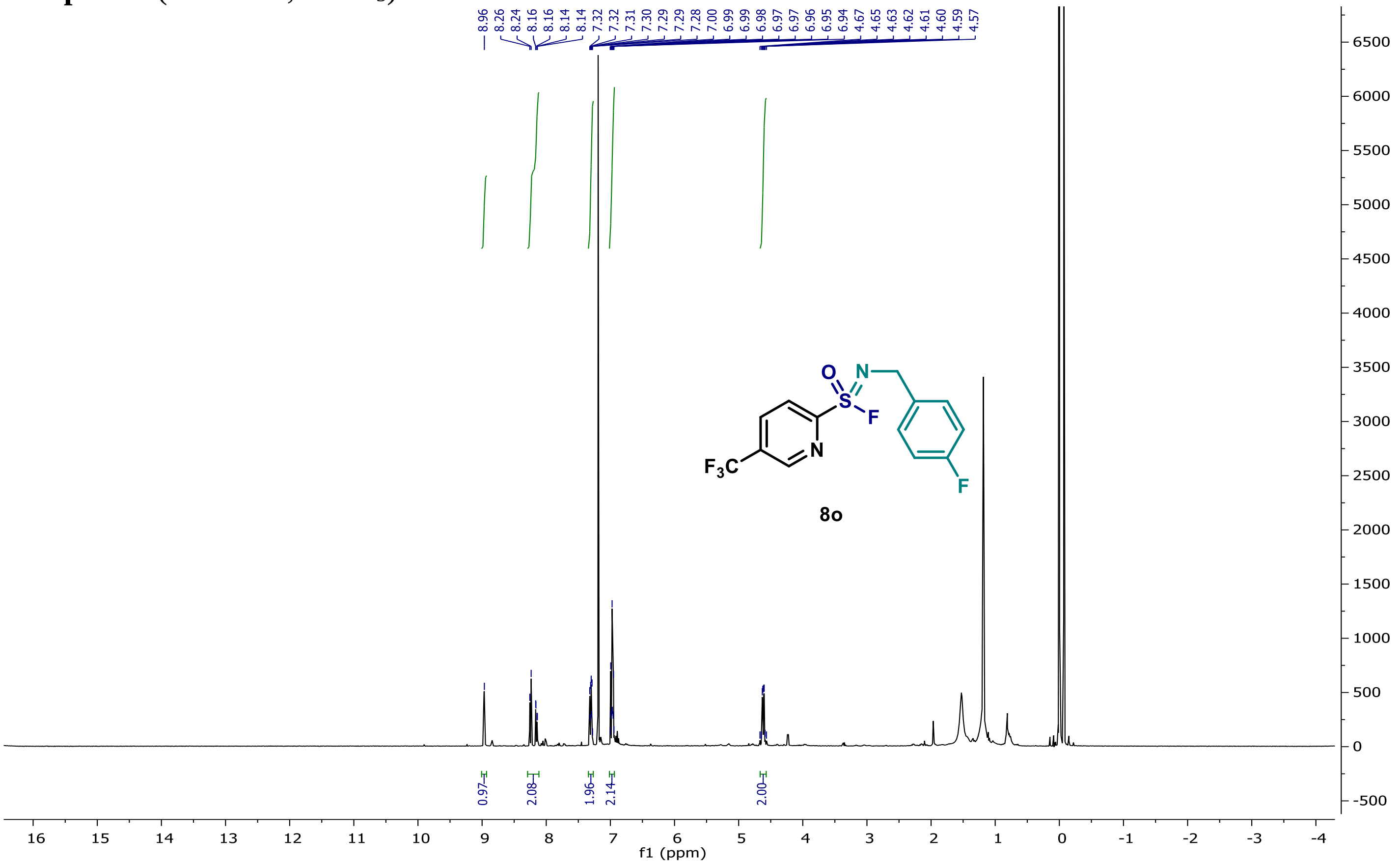

$^{19}F\{^{1}H\}$ NMR (282 MHz, $CDCl_3$)

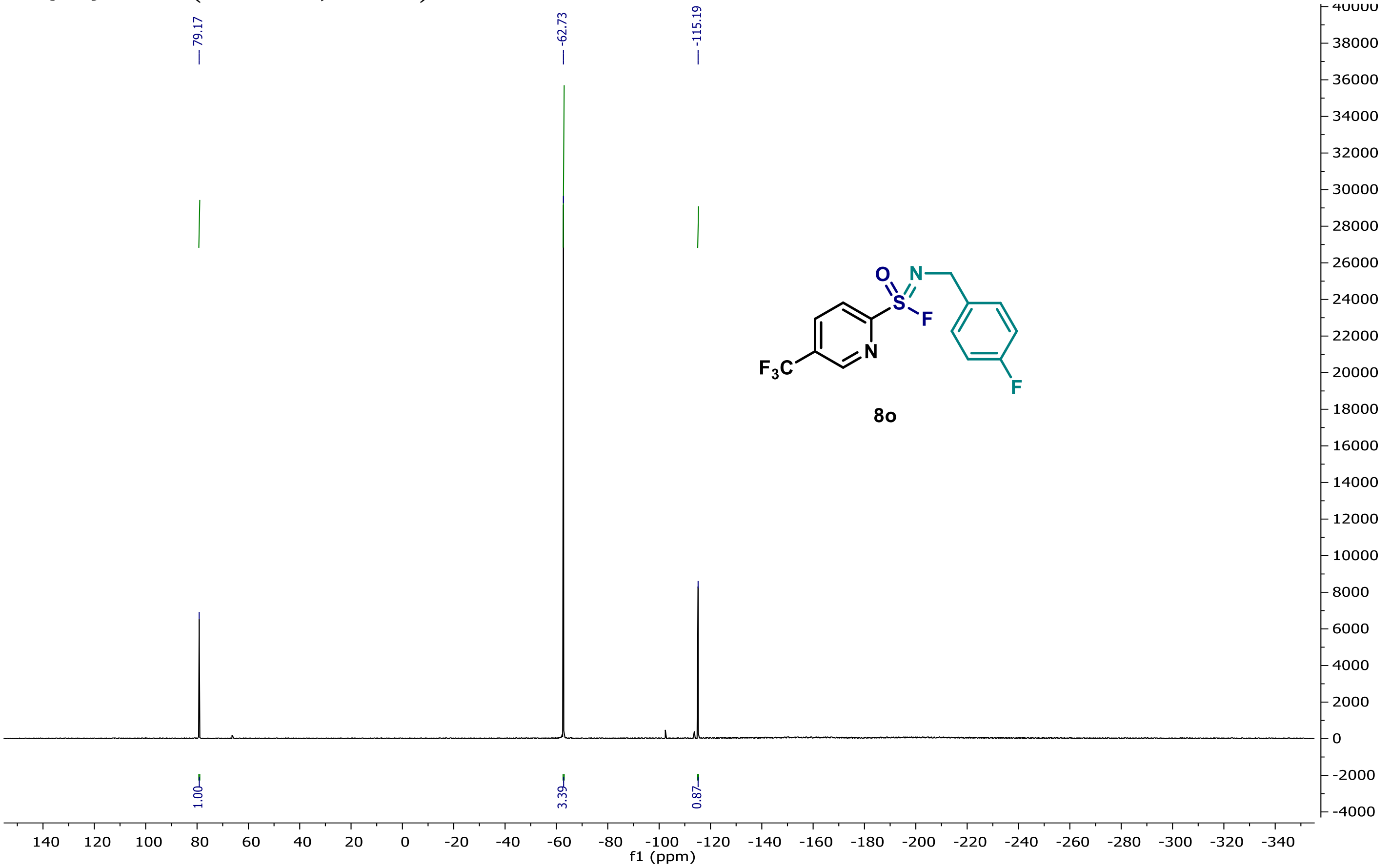

^{13}C Spectra (75 MHz, $CDCl_3$)

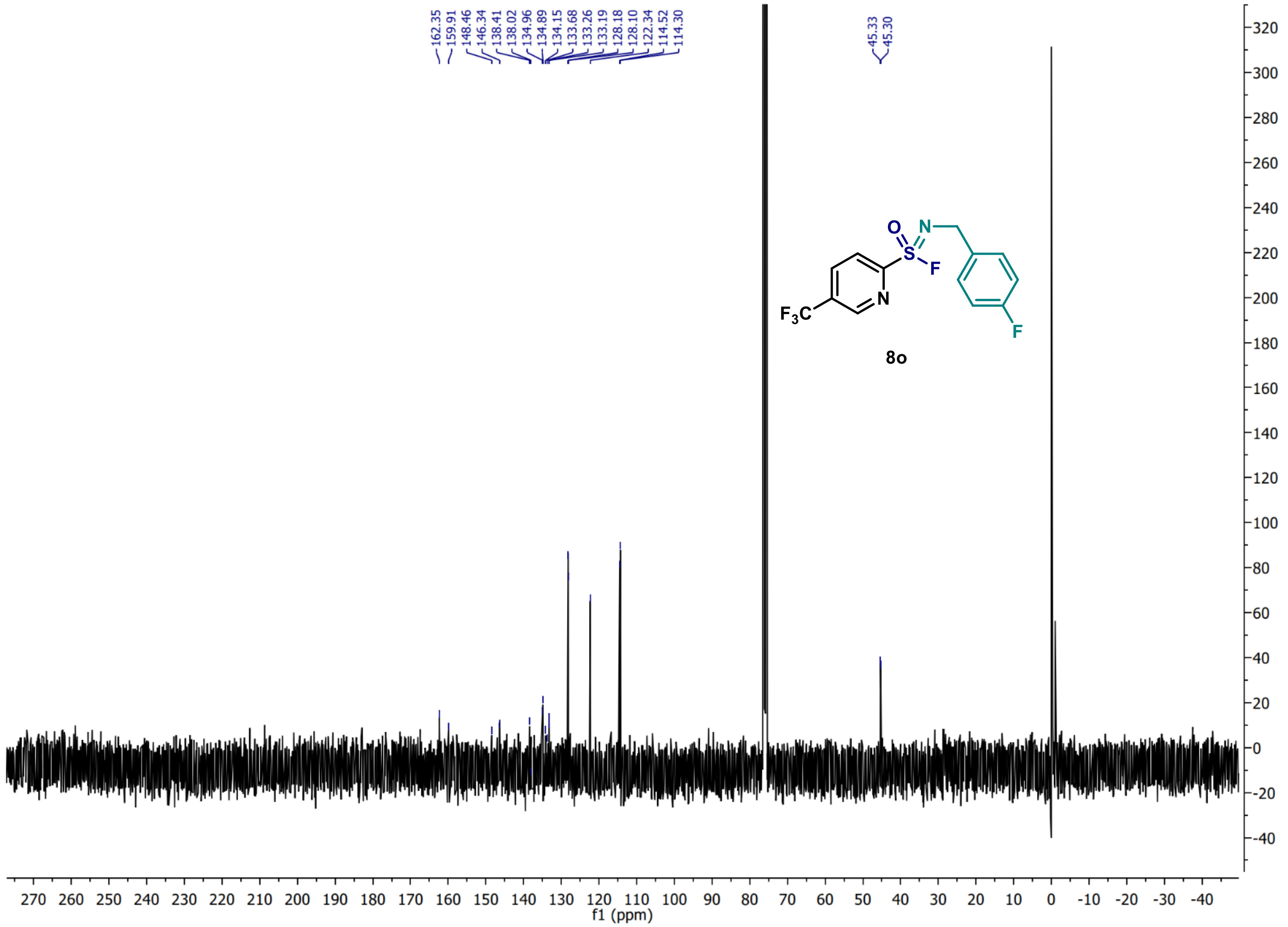

^{1}H Spectra (300 MHz, $CDCl_3$)

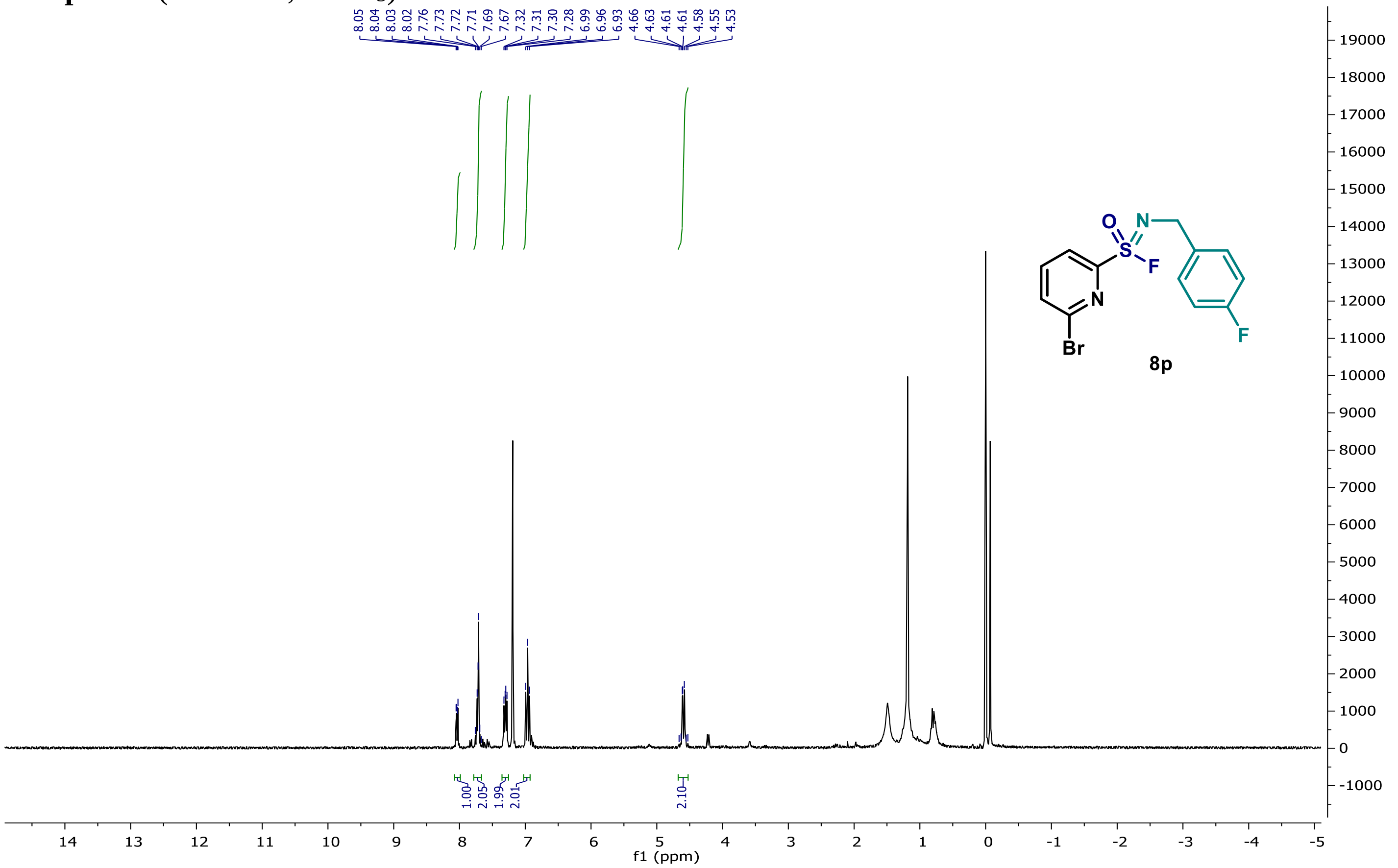

^{19}F{^{1}H} NMR (282 MHz, $CDCl_3$)

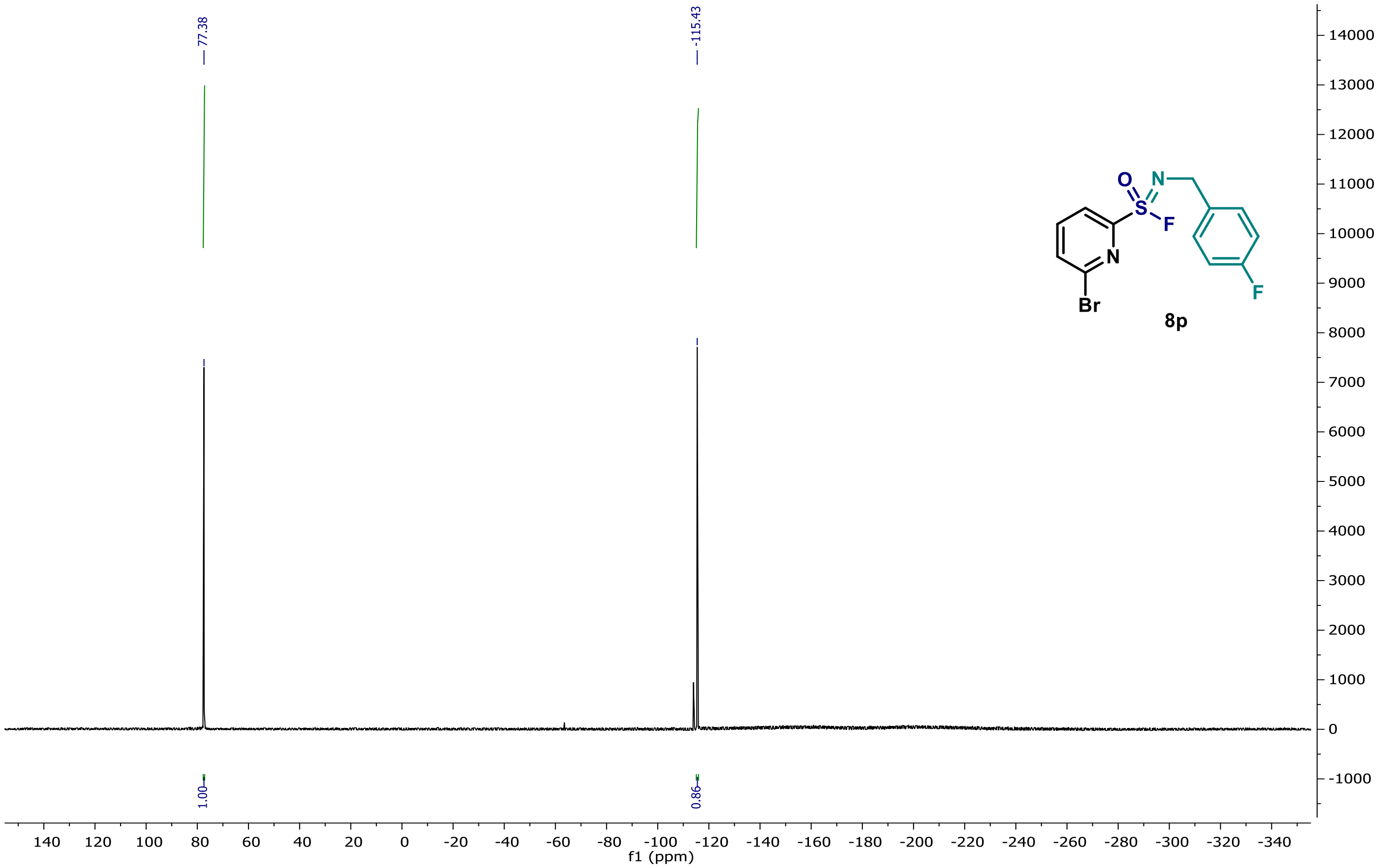

^{13}C Spectra (75 MHz, $CDCl_3$)

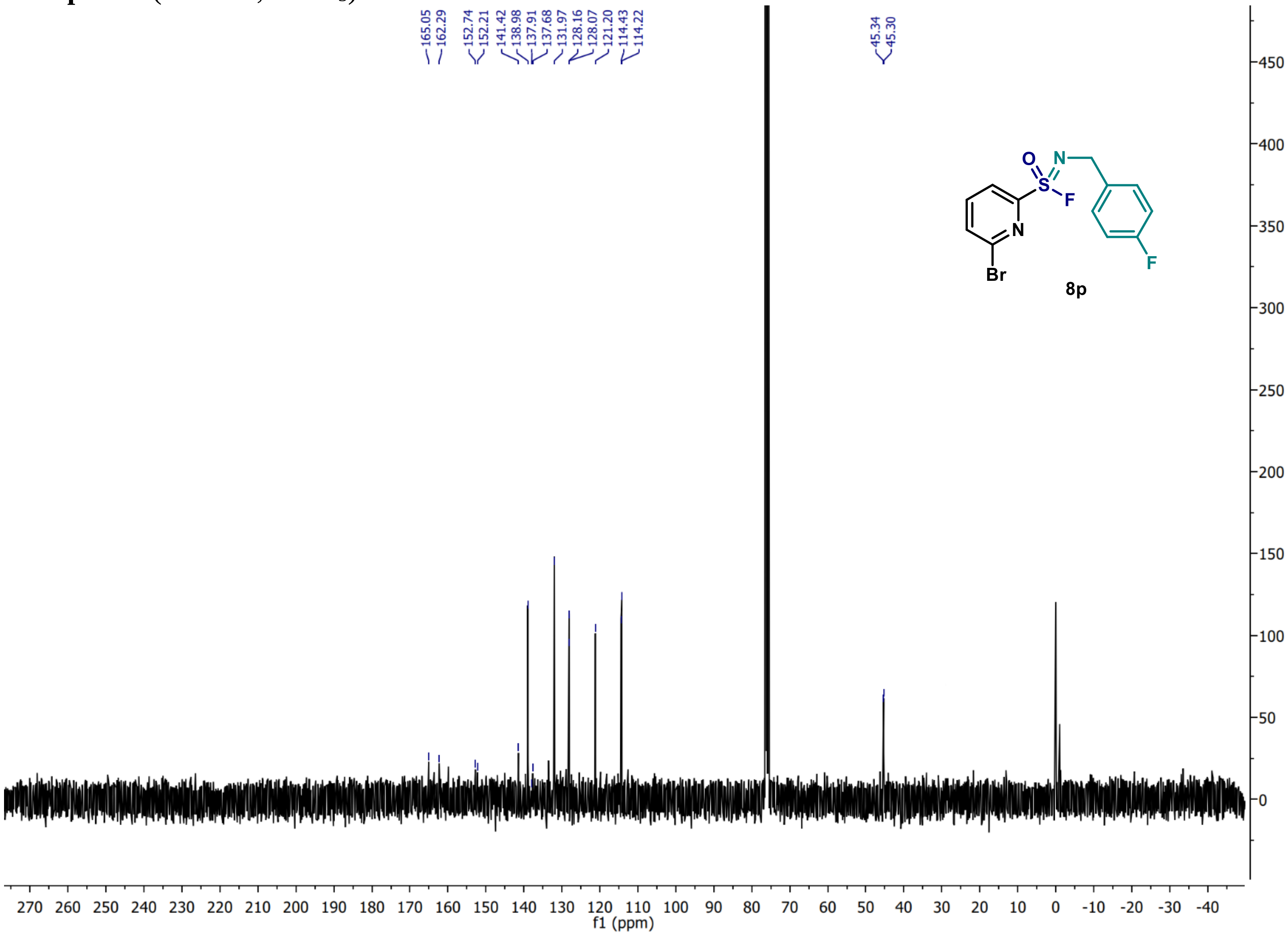

Supplement: Supplementary file 1 — Supplementary [file ANIE-59-23510-s001.pdf]
